# Genome wide identification and characterization of nodulation related genes in *Arachis hypogaea*

**DOI:** 10.1371/journal.pone.0273768

**Published:** 2022-09-09

**Authors:** Kiran Khurshid, Anum Akram, Ahmad Ali, Faiza Munir, Alvina Gul, Ghulam Haider, Zuhra Qayyum, Rabia Amir

**Affiliations:** Department of Plant Biotechnology, Atta-ur-Rahman School of Applied Biosciences (ASAB), National University of Sciences and Technology (NUST), Islamabad, Pakistan; ICAR - National Institute for Plant Biotechnology, INDIA

## Abstract

Nitrogen is an important plant nutrient that has a significant role in crop yield. Hence, to fulfill the needs of sustainable agriculture, it is necessary to improve biological nitrogen fixation in leguminous crops. *Nod* inducing gene families plays a crucial role in the interaction between rhizobia and legumes, leading to biological nitrogen fixation. However, *nod* inducing genes identification and characterization has not yet been performed in *Arachis hypogaea*. In this study, identification and genome-wide analysis of *nod* inducing genes are performed so that to explore their potential functions in the *Arachis hypogaea* for the first time. Nod genes were comprehensively analyzed by phylogenetic clustering analysis, gene structure determination, detection of conserved motifs, subcellular localization, conserved motifs, *cis*-acting elements and promoter region analysis. This study identified 42 Nod inducing genes in *Arachis hypogaea*, their sequences were submitted to NCBI and accession numbers were obtained. Potential involvement of these genes in biological nitrogen fixation has been unraveled, such as, phylogenetic analysis revealed that *nod* inducing genes evolved independently in *Arachis hypogaea*, the amino acid structures exhibited 20 highly conserved motifs, the proteins are present at different locations in cells and the gene structures revealed that all the genes are full-length genes with upstream intronic regions. Further, the promoter analysis determined a large number of *cis*-regulatory elements involved in nodulation. Moreover, this study not only provides identification and characterization of genes underlying developmental and functional stages of nodulation and biological nitrogen fixation but also lays the foundation for further revelation of *nod* inducing gene family. Besides, identification and structural analysis of these genes in *Arachis hypogaea* may provide a theoretical basis for the study of evolutionary relationships in future analysis.

## Introduction

Gene expression regulation at a transcriptional and post-transcriptional levels might influence and control important biological activities and processes such as perception of compounds, signal transduction, cellular morphogenesis, environmental stresses and beneficial interaction with microbes [1, 2]. Nodulation genes are involved in plant-microbe interaction leading to biological nitrogen fixation, an essential development for plant growth. Nodulation is a stepwise process that includes flavonoid exudation from roots and perception on rhizobia receptors, signal transduction, nod gene expression, root infection and nodule organogenesis [3]. And infection is controlled by the legume through a suite of nodulin genes which are temporally and spatially regulated [4]. After nodule formation, nitrogen is fixed by rhizobia and offer better growth to host plant in nitrogen scare soils, whereas rhizobia in return receive carbon for energy. Rhizobia uses ancient infection mode ‘crack entry’ for species of genus Arachis (counting *Arachis hypogaea*) without the development of infection threads which is easier to be established in non-legume crops [1, 5]. Plant productivity, species diversity in the natural ecosystem and agriculture, and the global nitrogen cycle is highly influenced by the nodulation process [1, 6].

Nodulation involves symbiotic signaling which initiates two parallel developmental processes i.e., bacterial infection and organogenesis of the nodule [7]. Flavonoid, a key signal in plant-microbe symbiosis, released from roots (as root-exudates) is recognized by rhizobia in the soil [8]. Upon chemotactic signal (flavonoid) by plant, rhizobia produce the signaling molecules known as nod factors (NFs) that signaled back to plant for bacterial entry into roots [7]. Rhizobia and host plant specificity is determined by NF which triggers the infection process and nodules formation [9]. Recognition of NFs is controlled by NF receptor genes such as NFP and LYK3 in *M*. *truncatula* and NFR5 and NFR1 in *L*. *japonicus*, OsCEBiP and/or LysM receptor kinases OsCERK1 in rice and LysM proteins such as AtCERK1, and AtLYK5 in Arabidopsis [10–16]. Moreover, DMI2 and receptor-like kinases SYMRK in *M*. *truncatula* and *L*. *japonicus* respectively, act as co-receptors in NF signaling [17]. The recognition process of NF activates a secondary messenger that initiates nucleus calcium oscillations and is responsible for proteins in nuclear membranes such as CASTOR, POLLUX and nuclear pore (NENA, NUP133 and NUP85) [17]. CYCLOPS and CCaMK complex decode calcium oscillations in the nucleus [17]. NSP1 and NSP2 genes in the nodulation signaling pathway encode transcription factors, that further trigger transcription factors ERN and nodule inception proteins (NIN), symbiotic signaling pathway transmits these signals for the occurrence of nodule morphogenesis [7, 17]. Furthermore, transcriptome analysis of *Arachis hypogaea* L. showed differently expressed genes (DEGs) which includes many orthologs to know genes of symbiotic signaling pathway such as ERN1, NSP2, and NFR5 [18]. Gene ontology enrichment analysis of these orthologs reveals their contribution in metabolic process, oxidative-reduction process, catalytic process, defense system and hormone biosynthesis [18]. Identification and classification of the nodulation genes in *Arachis hypogaea* are useful for future research on the expression of these genes, as to date no study has been performed on genome-wide characterization and identification of nodulation genes in *Arachis hypogaea*. In the *Arachis hypogaea*, this interaction is dependent on underground communications however, essential nutrients like nitrogen and soil organic matter may influence this relationship [19].

*Arachis hypogaea*, generally known as groundnut or peanut, is an essential food crop of subtropical and tropical areas. In the world, 84 countries grow peanut crops in an area of 27.66 million hectares having an annual production of 43 million tons of pods (nuts in-shell) i.e., with the 1590 kg ha^-1^ productivity [20]. The largest producers in the world are China, India, and the United States and exports are 1.25 million metric tons, approximately. Peanut seeds are an important contributor to human diets, as they are a rich source of lipids, proteins, and fatty acids. Oil content is about 47 to 50%, affecting its quality, flavor, and products [21]. About 55% of nitrogen required for growth and development is detained from biological nitrogen fixation in the peanut that could be enhanced (Hardarson 1993). In the present study, considering the importance of nodulation genes and lack of information about gene families in this process, we aimed to conduct a genome-wide identification and characterization of the NIG family genes in *Arachis hypogaea*.

## Methodology

### Identification and annotation of nodulation genes in *Arachis hypogaea*

To perform comprehensive analysis of Nod genes in *Arachis hypogaea*, different nodulation genes (NFP, SYMRK, NUP85, NUP133, CCaMK, CYCLOPS, NSP1, NIN, ERN1, Nod, Nol) were identified in model legumes [18]. A local BLASTp algorithm search was used to retrieve homologous FASTA sequences of these genes in NCBI (https://www.ncbi.nlm.nih.gov/). FASTA sequences retrieved from NCBI were used as queries to carry out BLASTn searches in peanut genomic database. The sequences was selected from PeanutBase (https://www.peanutbase.org/) with a cutoff e-value of 0e. To verify the reliability of results and to confirm each predicted sequences, all gene sequences were checked in the PFAM database (http://pfam.xfam.org/) [22] for domains. The full-length genes, half-length genes and genes having no domains in ORF are manually identified and then all the redundant sequences were removed. A total of 42 genes were designated as Nodulation related genes in *Arachis hypoagea* to perform further insilico analysis. The ExPASy translate tool (http://www.expasy.ch/tools/dna.html) was used to deduce the amino acid or open reading frames of *Nod* inducing genes. Then chemical and biophysical parameters of *Nod* inducing 42 genes in *Arachis hypogaea* were predicted by ProtParam (http://expasy.org/tools/protparam.html) available at ExPASy by using primary sequences of genes [23]. These properties were predicted to explore characteristics of genes e.g., protein length (aa), coding sequence (CDS), gene length (bp), molecular weight (MW), grand average of hydropathicity (GRAVY), isoelectric point (pI), instability index and aliphatic index (AI).

### Sequence alignment and phylogeny inference

The sequences of *nod* inducing genes were retrieved from PeanutBase (https://www.peanutbase.org) and multiple sequences of full gene length were aligned by using MEGA 10.2.4 tool (https://www.megasoftware.net/) at default setting by using “align by muscle”. An unrooted tree was also constructed using MEGA 10.2.4 tool with neighbor joining (NJ) algorithm. The bootstrap replicates of 1000 with 50% cutoff values were used to test the reliability of the tree and then the tree was visualized.

### Gene structure and conserved motif distribution analysis

Structural information of *Nod* inducing genes i.e., intron/exon patterns were predicted by using an online Gene Structure Display Server (http://gsds.gao-lab.org) [24, 25]. Gene structures were predicted by using coding regions (CDS) of genes in BED file format. The MEME (https://meme-suite.org/meme/) program was used for significant functional and conserved protein motifs prediction. This analysis was performed by adjustment of parameters as the optimum motif width: 3 residues, number of unique motifs: 20 and distribution of motifs: and any number of repetitions [26].

### Chromosomal distribution and gene duplication

The position information of NIG gene family and chromosomes length were acquired from PeanutBase (https://www.peanutbase.org). Relative distances and physical locations of genes was visualized on their respective 20 *Arachis hypogaea* chromosomes using the online visualization tool PhenoGram (http://visualization.ritchielab.org/phenograms/plot). To evaluate the tandem and segmental duplication events, divergence time and selective pressure of NIG-family genes, the synonymous (Ks) and non-synonymous (Ka) values were calculated by TB-tool software (https://github.com/CJ- Chen/TBtools) (Chen et al. 2020) [27]. The divergence time was calculated by using a formula T = Ks/2x*MYA (where x = 6.56 x 10^−9^ and MYA = 10^−6^) (He et al. 2016) [28].

The segmental duplication was then represented by drawing the red lines between two duplicated genes.

### Subcellular localization prediction and sequence logos analysis

To predict and better understand subcellular localization of NIG family proteins for several functions, all predicted 42 DNA FASTA sequences of *Arachis hypogaea* were translated to protein sequences via the online ExPASy translate tool (https://web.expasy.org/translate/). Plant-mPLoc (http://www.csbio.sjtu.edu.cn/bioinf/plant-multi/) was used to identify their subcellular locations [29]. Plant-mPLoc predictor allows localization of plant proteins at different 12 targets i.e., Chloroplast, Cell wall, Endoplasmic reticulum, Cytoplasm, Extracellular, Mitochondria, Golgi apparatus, Nucleus, Plasma membrane, Peroxisome, Plastid, Plasma membrane and Vacuole. Furthermore, sequence logos help to determine the amino acids that are conserved or non-conserved among all the genes. Hence for sequence logos analysis, proteins of 42 *NIG* family genes were aligned by CLUSTALW (https://www.genome.jp/tools-bin/clustalw) [30] and then sequence logos were generated by WebLogo (https://weblogo.berkeley.edu/logo.cgi) [31].

### Retrieval of promoter regions and *Cis*-acting elements analysis

Promoter sequence regions of *Arachis hypogaea*, 1.5 kb upstream of the translation site, was downloaded from the peanut database. The PlantCare tool (http://bioinformatics.psb.ugent.be/webtools/plantcare/html/) [32] was used for the investigation of *cis*-acting elements in the promotes of *NIG* genes of *Arachis hypogaea* and classified based on their known functions.

## Results

### Identification and annotation of NIG gene family in *Arachis hypogaea*

Previous studies identified many nodulation genes in legume plants [17, 18]. Genome-wide studies of nodulation genes were conducted in many legume plants by exploring its publicly available data [33]. The current study identified a total of novel 42 *NOD* inducing genes in *Arachis hypogaea* **(S1 Text)** by homology study. All the genes identified in NIG gene family, that encoded proteins having the nodulation function, were analyzed for the nodulation process according to their location on the chromosome. The ExPASy translate tool was used to deduce the amino acid or open reading frames of Nod inducing genes **(S2 Text)**. Based on PFAM analysis, the study determined whether each candidate gene contained the conserved domain, and the presence of related domains, respectively in the PFAM database was ultimately confirmed. Based on sequence identity with the functionally characterized NIG family, the individual names of all genes were given. The 42 full-length identified sequences were submitted to NCBI and their accession numbers were obtained. The gene names of the NIG family, their accession numbers, length of the coding sequences, and characteristics of these proteins are present in (Table 1).

**Table 1:** Characterization of NIG family genes in *Arachis hypogaea*.

Sr. No	Gene ID	Chromosomes	Accession numbers	Nucleotide CDS (bp)	Length (aa)	PI	Mw	II	AI	EC	GRAVY	Half life
1	**AhNMTL1**	13	MZ169526	2772	923	5.71	103395.37	42.89	91.86	107120	-0.228	>10 hours
2	**AhNMTL2**	03	MZ169527	2772	923	5.76	103503.55	41.53	91.33	110100	-0.235	>10 hours
3	**AhNKEF1**	05	MZ169528	1703	567	5.83	63918.94	34.12	82.70	58370	-0.403	>10 hours
4	**AhNKEF2**	15	MZ169529	1702	567	5.91	63952.92	35.14	82.36	59860	-0.422	>10 hours
5	**AhNKEF3**	17	MZ169530	1682	560	5.58	63090.23	44.69	81.86	49320	-0.477	>10 hours
6	**AhNKEF4**	13	MZ169531	734	559	5.58	62975.18	45.54	82.70	49320	-0.458	>10 hours
7	**AhNNLC1**	16	MZ169532	2208	735	4.82	72061.97	56.54	51.93	24980	-0.065	>10 hours
8	**AhNNLC2**	06	MZ169533	2211	736	4.82	72096.97	56.12	52.00	24980	-0.061	>10 hours
9	**AhNPR1**	17	MZ169534	2877	958	5.60	106770.63	53.07	72.35	97885	-0.558	>10 hours
10	**AhNPR2**	08	MZ169535	2895	964	5.63	107484.54	53.70	72.20	99375	-0.563	>10 hours
11	**AhNPR3**	15	MZ169536	2952	984	5.42	109253.43	58.20	76.19	86550	-0.420	>10 hours
12	**AhNPR4**	05	MZ169537	2958	986	5.48	109443.61	57.56	75.14	86550	-0.438	>10 hours
13	**AhNKLM1**	11	MZ169538	543	762	6.60	84511.47	41.55	88.39	106980	-0.183	>10 hours
14	**AhNKLM2**	01	MZ169539	1887	628	6.73	69666.50	43.30	86.43	87040	-0.211	>10 hours
15	**AhNNup1**	16	MZ170086	2151	716	6.36	81199.26	51.55	98.35	150395	-0.214	>10 hours
16	**AhNNup2**	16	MZ147089	2147	716	6.19	81118.17	51.30	98.48	150395	-0.203	>10 hours
17	**AhNNup3**	07	MZ169571	3996	1331	5.64	149014.72	43.77	84.40	202275	-0.344	>10 hours
18	**AhNNup4**	17	MZ169572	3993	1330	5.69	149018.71	43.67	84.83	200785	-0.352	>10 hours
19	**AhNKEF5**	11	MZ169573	1554	517	5.69	57794.12	46.51	92.57	42900	-0.251	>10 hours
20	**AhNKEF6**	01	MZ169574	1549	517	5.69	57820.20	45.76	93.33	42900	-0.241	>10 hours
21	**AhNPR5**	19	MZ169575	2848	969	5.63	107839.27	57.97	77.17	74060	-0.406	>10 hours
22	**AhNPR6**	09	MZ169576	2842	967	5.59	107515.96	58.36	78.35	75550	-0.384	>10 hours
23	**AhNKLM3**	05	MZ169577	1788	595	7.50	65636.76	39.83	101.04	66405	0.068	>10 hours
24	**AhNKTyr**	15	MZ169578	1538	475	8.51	52693.98	37.77	101.75	61685	0.017	>10 hours
25	**AhNSur1**	20	MZ169579	1242	413	7.88	47056.71	31.87	75.74	61365	-0.461	>10 hours
26	**AhNSur2**	10	MZ169580	1242	413	8.43	47075.70	31.66	75.71	61365	-0.462	>10 hours
27	**AhNSur3**	03	MZ169581	1188	395	6.49	44018.17	34.48	85.54	64010	-0.279	>10 hours
28	**AhNSur4**	12	MZ169582	1112	343	7.15	38749.59	39.61	66.97	50935	-0.699	>10 hours
29	**AhNSur5**	11	MZ169583	1727	548	6.33	62814.13	38.11	69.78	95535	-0.595	>10 hours
30	**AhNSur6**	02	MZ170085	1350	449	9.08	51019.07	39.19	79.15	63635	-0.456	>10 hours
31	**AhNSur7**	13	MZ170073	1314	438	6.19	48600.88	31.79	79.61	65625	-0.332	>10 hours
32	**AhNSur8**	03	MZ170074	1394	471	6.31	52449.43	34.31	80.25	76750	-0.288	>10 hours
33	**AhNSur9**	14	MZ170075	1170	390	7.53	43585.90	30.48	80.85	76625	-0.282	>10 hours
34	**AhNSur10**	04	MZ170076	1155	384	7.54	42957.07	27.62	80.60	78115	-0.315	>10 hours
35	**AhNSur11**	13	MZ170077	1359	452	8.08	50105.22	34.39	86.19	68605	-0.276	>10 hours
36	**AhNSur12**	01	MZ170078	1275	424	6.64	47951.66	38.58	78.33	58385	-0.398	>10 hours
37	**AhNSur13**	12	MZ170079	1158	384	6.47	43819.72	39.84	76.07	58385	-0.483	>10 hours
38	**AhNSur14**	02	MZ170080	1170	390	6.24	44544.47	42.63	75.67	58385	-0.494	>10 hours
39	**AhNSur15**	12	MZ170081	1245	412	6.33	46813.03	37.37	74.95	56895	-0.506	>10 hours
40	**AhNSur16**	02	MZ170082	1245	412	6.13	46837.01	37.78	75.90	56895	-0.510	>10 hours
41	**AhNSur17**	10	MZ170083	1245	412	6.13	46837.01	37.78	75.90	56895	-0.510	>10 hours
42	**AhNSur18**	20	MZ170084	1245	412	6.13	46837.01	37.78	75.90	56895	-0.510	>10 hours

The biochemical properties and physical parameters of Nod inducing 42 genes in *Arachis hypogaea* were predicted by ProtParam available at ExPASy by using primary sequences of genes. These properties were predicted to explore important characteristics of genes e.g., coding sequence (CDS), protein length (aa), isoelectric point (pI), molecular weight (MW), aliphatic index (AI), extinction coefficients (EC) by assuming all pairs of Cys residues form cystines, grand average of hydropathicity (GRAVY) and their estimated half-life. The full-length coding sequences of the Nod inducing genes ranged from 543 bp (AhNKLM1) to 3996 bp (AhNNup3) and their putative proteins contained between 343 and 1331 amino acid (aa) residues, with an average of ~631 aa. The theoretical pI ranged from 4.82 (AhNNLC1, AhNNLC2) to 9.08 (AhNSur6), and molecular weights ranged from 42957.07 (AhNSur10) to 149018.71 (AhNNup4). In congruence with the features, the genome-wide studies also detected significant variation in an aliphatic index, extinction coefficients and GRAVY inferring a high degree of complexity and functional diversification among the NIG family of *Arachis hypogea* (Table 1).

### Evolutionary analysis of NIG gene family

**Fig 1:**
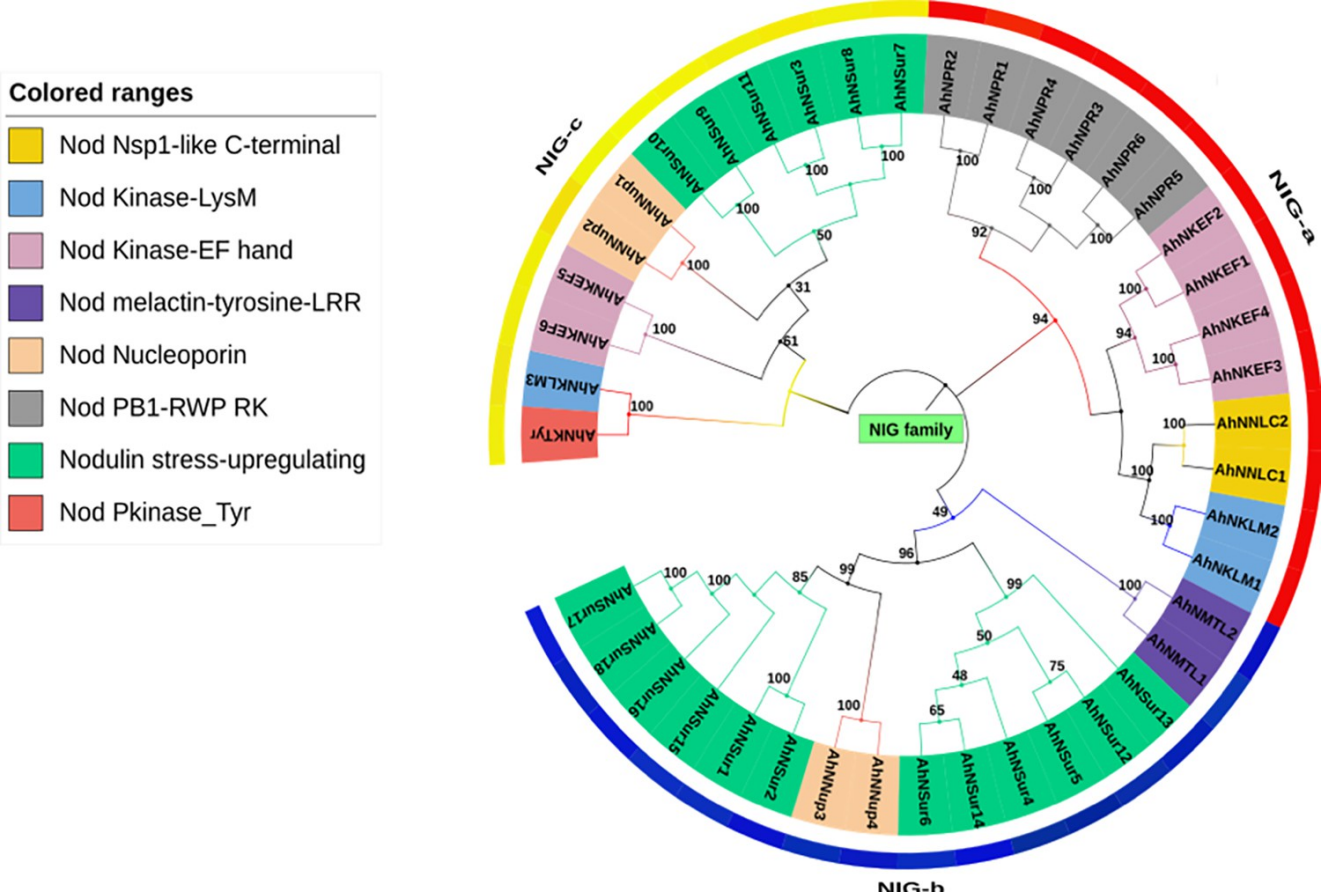
Phylogenetic analysis of NIG family genes. Full length gene sequences were aligned and phylogenetic tree was constructed using MEGA10 software by maximum Likelihood (ML) method with 1000 bootstrap replicates. Different groups or genes are highlighted in different colors according to the domains present in these genes.

To assess the evolutionary relationship, sequences of all the identified genes of NIG family (*Nod* Inducing Gene- family) were aligned and a phylogenetic tree was constructed using neighbor- joining method (Fig 1). All relationships were made by full-gene multiple sequence alignment of 42 *Nod* inducing genes and all ambiguous positions were removed for each sequence pair (pairwise deletion option). The phylogenetic analysis of these gene sequence resulted in the well-resolved tree, showing that the NIG family in *Arachis hypogaea* are classified into three major sub-families referred to as NIG-a, NIG-b and NIG-c. NIG-a has 14 members comprising of 5 domains while NIG-b having the maximum number of 16 members comprising of four domains. In addition to these families, sub-family NIG-c have the minimum number of 12 members comprising of four domains. Whereas all these three sub-families indicate that they are derived from a common ancestor with close homology. Based on their domains, NIG family represent functional similarities and have a close phylogenetic relationship. Furthermore, sub-families were divided into twelve groups, namely NIG-a1, NIG-a2, NIG-a3, NIG-a4, NIG-b1, NIG-b2, NIG-b3, NIG-b4, NIG-c1, NIG-c2, NIG-c3 and NIG-c4, on the basis of structural and functional similarities of genes. For instance, the genes that were more related to each other regarding structure and function were appeared in the same group. Thus, based on closed phylogenetic reconstruction, we speculate that these sub-families demonstrate biological nitrogen fixation. Conserved domain analysis revealed that groups of sub-family NIG-a were clustered together in a phylogenetic tree with NIG-a1, NIG-a2, NIG-a3 and NIG-a4. While groups of sub-family NIG-b contain NIG-b1, NIG-b2, NIG-b3 and NIG-b4 showed a close relationship. Meanwhile, sub-family NIG-c reported NIG-c1, NIG-c2, NIG-c3 and NIG-c4 groups have a relationship. As 6 members of NIG-a1 contains *AhNPR1*, *AhNPR2*, *AhNPR3*, *AhNPR4*, *AhNPR5 and AhNPR6* genes having RWP-RK and PB1 domain, that are reported to be involved in nodule perception as they are involved in nitrate response and nodulation. However, this insilico study provided the further characterization of 4 members (AhNKEF1, AhNKEF2, AhNKEF3 and AhNKEF4) of the same sub-family NIG-a i.e., NIG-a2 based on their domains that are predicted to function in rhizobium infection. Moreover, the 2 members within NIG-a3 i.e., *AhNNLC1* and *AhNNLC2* has Nsp1-like-C-terminal region and thereby seeming to participate in infection and normal rhizobia bacteroid formation in the nodule. Two members of NIG-a4 group consist of *AhNKLM1* and *AhNKLM2* genes which contain protein kinase domain and LysM domain. Subsequently, 2 members, *AhNMTL1* and *AhNMTL2* belongs to NIG-b1 group and we identified that they can function in root nodule symbiosis. The two members in NIG-b3 contains the A*hNNup3* and *AhNNup4* gene having Nup85 Nucleoporin domain which might be an important part of nucleopore subcomplex and has been demonstrated to be involved in rhizobia colonization. Most of the groups with the SurNod19 domain belongs to NIG-b sub-family. As NIG-b2 has 6 members *(AhNSur4*, *AhNSur5*, *AhNSur6*, *AhNSur12*, *AhNSur13* and *AhNSur14)* while 6 members of NIG-b4 contains *AhNSur1*, *AhNSur2*, *AhNSur15*, *AhNSur16*, *AhNSur17* and *AhNSur18* genes. Besides these two, 6 members of NIG-c1 *(AhNSur3*, *AhNSur7*, *AhNSur8*, *AhNSur9*, *AhNSur10*, *AhNSur11)* has the same domains and it was interesting to study that the NIG-b2, NIG-b4 and NIG-c1 are functionally same, which has been documented to participate in nodule development. The NIG-c has functionally characterized groups suggesting that most of the groups of this sub-family have similar functions as groups of NIG-a and NIG-b. While the NIG-c2 has functionally similar 2 members (*AhNNup1* and *AhNNup2*) as NIG-c3. Members as *AhNKEF5* and *AhNKEF6* belongs to NIG-c3 and has functional similarity to NIG-a2 i.e., according to their domains they are characterized as rhizobial infection and nodulation causing genes. The 2 members of NIG-c4 *(AhNKLM3* and *AhNKTyr*) include LysM, protein kinase and Pkinase_Tyr domains which have been reported to be involved in rhizobial nodule symbiosis. Hence, these studies revealed evolutionary relationships of *Nod* inducing genes in NIG family and the domains of these genes are implicated in causing rhizobial infection and nodulation which leads to biological nitrogen.

### Conserved motif analysis and intron/exon organization of NIG gene family

**Fig 2:**
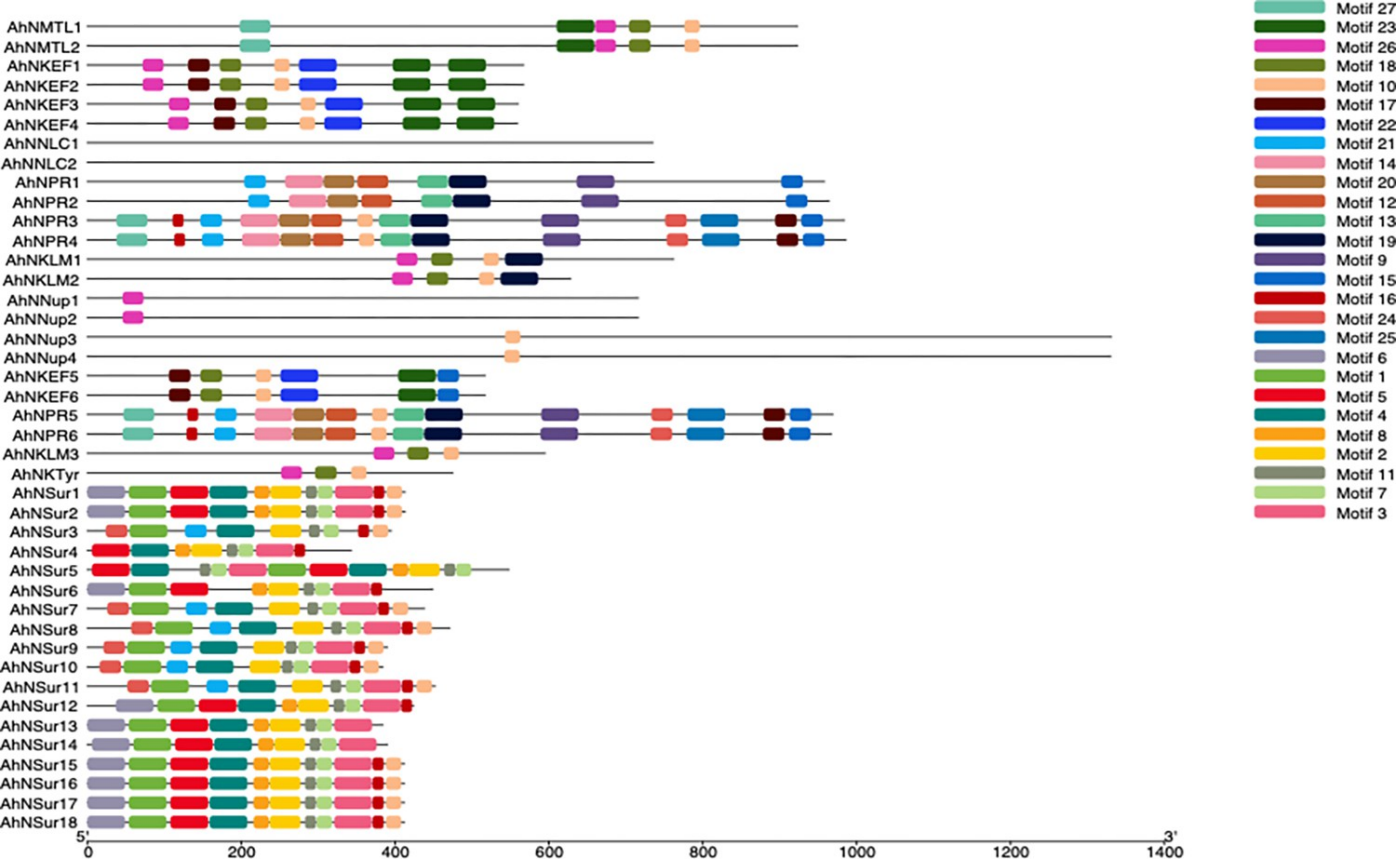
Conserved protein motifs of *NIG* gene family. The protein motifs of *NIG* genes are shown as colored boxes. The proteins or motifs are ordered according with the order of sub-family NIG-a, NIG-b and NIG-c. Twenty seven predicted motifs were presented by different colored boxes and schematically arranged in accordance with the pattern of phylogenetic tree.

Conserved motifs of NIG family genes were predicted by utilizing the online MEME tool. However, the conserved motif prediction is essential to further gain an understanding of diversification and structural characteristics of genes. The detailed information about the 42 genes of NIG family including name, width and best possible matches is presented by this study. The current study found 27 conserved motifs with different amino acids ranges from 15–50. Identified motifs and their schematic distribution in all sub-groups (Fig 2). In addition, the sequence, sites and width for each conserved motif (Table 2).

**Table 2:** Conserved sequences, sites, width and e-values of *NIG* family genes motifs.

Motif	Conserved amino acid sequences	e-values	Sites	Width
1.	YFDIEFPRGHIGIKNFQAELVDEHGNSIPLYEAYLHHYFVLRYFENITMS	2.7e-591	17	50
2.	YNNKQKRKVYLKYTVTWVDWDQYQVPLKFYILDVTDQVTYN	2.5e-476	18	41
3.	AHVHSGIVNATLYGEBGRVLCEVKPTYGTGEEAGNEKGYVVGMSGCYPKP	1.0e-566	17	50
4.	ENVPKEYDEEKWLINILVIDTRGTEDKKGCTECRCDLYNVKSEDFGNPTG	2.8e-537	17	50
5.	ZSQPIYGKYFRRNDGVCQGSVNSYSWGLGVDARKTSLELPDPFRIEVGTH	1.8e-453	12	50
6.	MKFICEVVJLSLSIIVJQSSIIFSRZLENPNHIKTATFYTKTFVLEPGKV	4.5e-306	10	50
7.	EGHYHIKRTKIPMKKGGNLIY	1.4e-200	18	21
8.	SDYKGGIFCCEKKSQCKLQKG	1.3e-188	12	21
9.	QRYFAGSLKDAAKSLGVCPTTMKRICRQHGISRWPSRKINKVNRSLSKLK	8.6e-169	6	50
10.	EKKDVTGLGVVFYILLAGDLP	4.7e-145	30	21
11.	HNCAVEYSITPQNTD	1.4e-140	18	15
12.	QICNEGRQNALAEILEILTVVCETHNLPLAQTWVPCRHRSV	1.4e-113	6	41
13.	HHLQQGQGVAGRAFLSHNMSFCPNITRFCKTDYPLVHYALM	2.3e-125	6	41
14.	GRVYQQKVPEWTPDVQYYSSKEYPRRDHAQHYNVRGTLALPVFEPPGQSC	2.8e-150	6	50
15.	FDIKYLDDDHEWVLITCDADLQECIDVLR	4.5e-088	8	29
16.	GSIKIKDGEILTLEF	2.1e-084	17	15
17.	AYEDDJIVHIVMELCGGGELFDRIVKRGK	4.4e-096	10	29
18.	ILAYLHEHHKPGVVHRDJKPENILLDNKM	3.0e-107	12	29
19.	FGLTAAFAICLRSSHTGSDDYVLEFFLPPEJTDFNEQKKLLGSJLLIIKQ	3.6e-113	8	50
20.	VGVLELIMTSQKINYAPEVDKVCKALEAVNLKSSEILEHQY	1.1e-093	6	41
21.	AIVRNSGRCQLNTLGQYFGLGSETRGLHT	1.4e-093	10	29
22.	AILKGHIDFKEEPWPGISDSAKDLIRKMLTPDPSKRLTAQEVLSHPWIIE	1.3e-092	6	50
23.	MDTDKDGRVTYEELKAGLRKLGSTLADNEJRLLMEVADVDRSGLJDYGEF	3.7e-079	8	50
24.	SSYDKTNPKSKTAVHLSPKIEIGPGKVSN	4.8e-070	10	29
25.	QPPTNALNYPTAYTAPDVERTEPQEPFGGMLLEGVGSSKDLRNLCPLEDZ	4.0e-058	4	50
26.	RGNFGLVYLYTENGSLEEWLCKEISKRK	2.7e-053	12	28
27.	EHSPSAPNPMSPFIFPTSSEQPYSPLWLFSDVEDEKHNVTL	2.2e-048	6	41

The number of motifs were comparatively greater in NIG-b sub-family than NIG-a and NIG-c sub-families. Motif 1 was found in all Nodulin stress up-regulating genes of groups NIG-b2, NIG-b4 and NIG-c1 except for *AhNSur4*. Similarly, motif 4 was exclusively present in all Nodulin stress up-regulating genes except for *AhNSur6*. Motif 2, 7 and 11 were unique to all genes of NIG-b2, NIG-b4 and NIG-c1 groups. Motif 5 and 8 were encountered in NIG-b2 and NIG-b4 groups. Motifs 6 is present in 10 (AhNSur1,2,6,12,13,14,15,16,17 and 18) out of 18 genes of *AhNSur* group, suggesting that these motifs might have conserved functions. Motif 3, 16, 21, 24 were detected only within some genes of *AhNSur* and *AhNPR* groups. Motif 9, 12, 20 and 14 were found in NIG-a1 group. Motif 25 was also encountered in NIG-a1 group except for AhNPR1 and AhNPR2 genes. Motif 13 and 27 were documented for all genes of NIG-a1 and NIG-b1. Motif 15, 17, 19 and 23 were unique to some genes of NIG-a1, NIG-a2, NIG-a4 and NIG-b1 groups, suggesting that these genes may be derived from a common ancestor. Motif 22 was only encountered in all *AhNKEF* genes. Motif 18 and 26 were observed within most genes of groups NIG-a2, NIGa-4, NIG-b1, and NIG-c2, NIG-c3 and NIG-c4. The analysis also suggests that motif 10 is conserved to most of the NIG family (present at 30 different sites; having 21 amino acids sequence), suggesting that it could be a signature motif associated with NIG family genes. While the non-conservation between motifs of NIG family predicts groups specificity, which could be related to diversifications in their functions. Furthermore, analysis revealed the conserved domains in genes of NIG family are positioned at similar locations and the results demonstrate that majority of the genes of this family are closely related and they have common compositions of motifs which represents that the gene structures are highly conserved.

**Fig 3:**
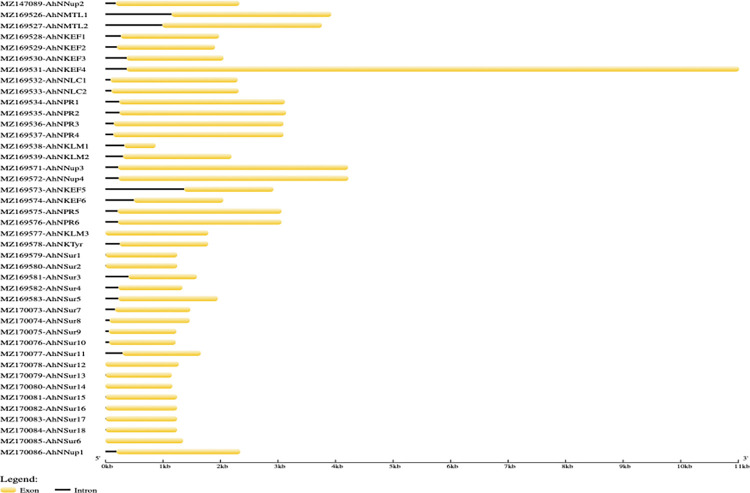
The gene structure of *NIG* family genes. The coding sequence and intronic regions are plotted using yellow boxes and black lines respectively. The genes are ordered with their accession numbers.

Structural diversity and functional characterization of *NIG* family genes are important to gain information about the evolution of this gene family. Hence to analyze the exon/intron structure of *NIG* family genes BED files were used, the analysis suggested that all are full-length genes i.e., the domains for nodulation lie inside the CDS regions. However, there are intronic regions in these genes and these intronic regions are upstream of the CDS regions. While the genes *AhNSur6*, *AhNSur12*, *AhNSur14* and *AhNKLM3* contained no intronic region (Fig 3). These observations indicated that each group in NIG family, shows maximum common gene structural conservation.

### Chromosomal location and gene duplication of NIG genes

**Fig 4:**
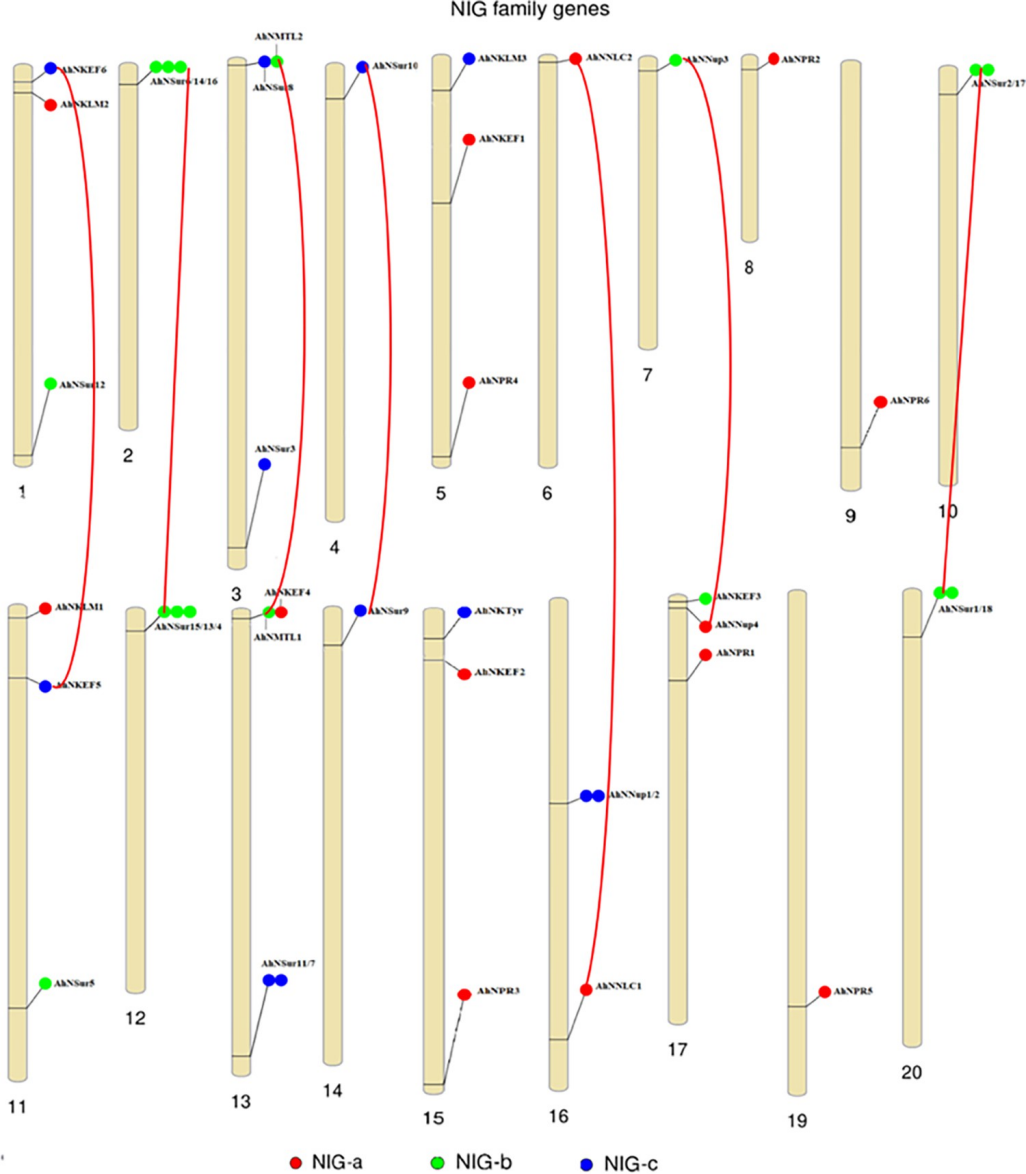
Chromosomal localization of *NIG* family genes on *Arachis* hypogaea chromosomes. The candidate *NIG* genes were designated on respective chromosomes from top to bottom according to their order.

Chromosomal locations of 42 *NIG* gene family were investigated on their corresponding 20 *Arachis hypogaea* chromosomes. The genes were widely distributed throughout the *Arachis hypogaea* genome. Genes were mapped on chromosomes according to their order (Fig 4). However, th-e genes show unequal distribution on the chromosomes. Uneven distribution of NIG gene family on chromosomes indicates that the evolutionary process leads to genetic variation. Among 20 chromosomes, 9 chromosomes harbored maximum number of 3 genes (Chr01: *AhKEF6*, *AhNKLM2*, *AhNSur12*; Chr02: *AhNSur6*, *AhNSur14*, *AhNSur16*; Chr03: *AhNSur8*, *AhNMTL2*, *AhNSur3*; Chr05: *AhNKLM3*, *AhNKEF1*, *AhNPR4*; Chr11: *AhNKLM1*, *AhNKEF5*, *AhNSur5*; Chr12: *AhNSur15*, *AhNSur13*, *AhNSur4*; Chr15: *AhNKTyr*, *AhNKEF2*, *AhNPR3*; Chr16: *AhNNup1*, *AhNNup2*, *AhNNLC1*; Chr17: *AhNKEF3*, *AhNNup4*, *AhNpr1*). Chr13 has a higher number of *NIG* genes as compared to others. While there was no *NIG* gene located on Chr18. Chr10 and Chr20 hosted two genes. *AhNsur10*, *AhNNLC2*, *AhNNup3*, *AhNPR2*, *AhNPR6*, *AhNSur9* and *AhNPR5* are located on Chr04, Chr06, Chr07, Chr08, Chr09, Chr14 and Chr15 respectively. In plants, presumably, duplication mechanisms occur during gene families expansion; usually, these mechanisms involve segmental duplication, tandem duplication and whole-genome duplication [34]. Hence to understand the possible relationship between *NIG* gene family and potential gene duplication within the *Arachis hypogaea* genome, we analyzed the duplication mechanisms during the evolution of this gene family here. By analyzing the sequence coverage and similarities of 42 *NIG* genes, we identified 7 pairs of genes (A*hNKEF5/AhNKEF6*, *AhNSur9/AhNSur10*, *AhNNCL1/AhNNCL2*, *AhNMTL1/AhNMTL2*, *AhNNup3/AhNNup4*, *AhNSur1/AhNSur2* and *AhNSur15/AhNSur16*) experienced segmental duplication. While there was no tandem and whole-genome duplication event. These observations were suggested that segmental duplication contributed largely to the expansion of NIG family members in *Arachis hypogaea*. These segmental duplication events were presented by constructing red lines on the Fig 4.

The *Arachis hypogaea* chromosomes (Chr02, Chr10, Chr12, Chr13, Chr16, and Chr20) also have nodulation inducing overlapping genes. Ka and Ks values for these duplicated gene pairs were calculated by using the KaKs calculator and then obtained information was used to determine selective evolutionary pressure. 6 out of 7 gene pairs had Ka/Ks<1 which indicated purification selection while one gene pair had Ka/Ka>1 which implies positive selection during evolution. In addition, segmental duplication events of 7 gene pairs were predicted to occurred between 0.286 and 41.607 million years ago (Table 3).

**Table 3:** *Ka*, *Ks* and *Ka_Ks* calculation and divergent time of duplicated *Arachis hypogaea NIG* family genes.

S. No	Paralogous Pairs	Ka	Ks	Ka_Ks	Duplication	Time (Mya *)
1	AhNKEF5- AhNKEF6	0.54589071	0.52131698	1.0471378	SD	41.607524
2	AhNSur9- AhNSur10	0.0113413	0.06329949	0.17916888	SD	0.86442829
3	AhNNLC1- AhNNLC2	0.00376216	0.03565028	0.10552952	SD	0.28674973
4	AhNMTL1- AhNMTL2	0.00615291	0.01876564	0.32788171	SD	0.46897178
5	AhNNup3- AhNNup4	0.04099033	0.05346009	0.76674648	SD	3.12426328
6	AhNSur1- AhNSur2	0.02139904	0.06607038	0.32388251	SD	1.63102454
7	AhNSur15- AhNSur16	0.00522741	0.02578523	0.20272897	SD	0.39843085

### NIG family protein subcellular localization and sequence logos analysis

**Fig 5:**
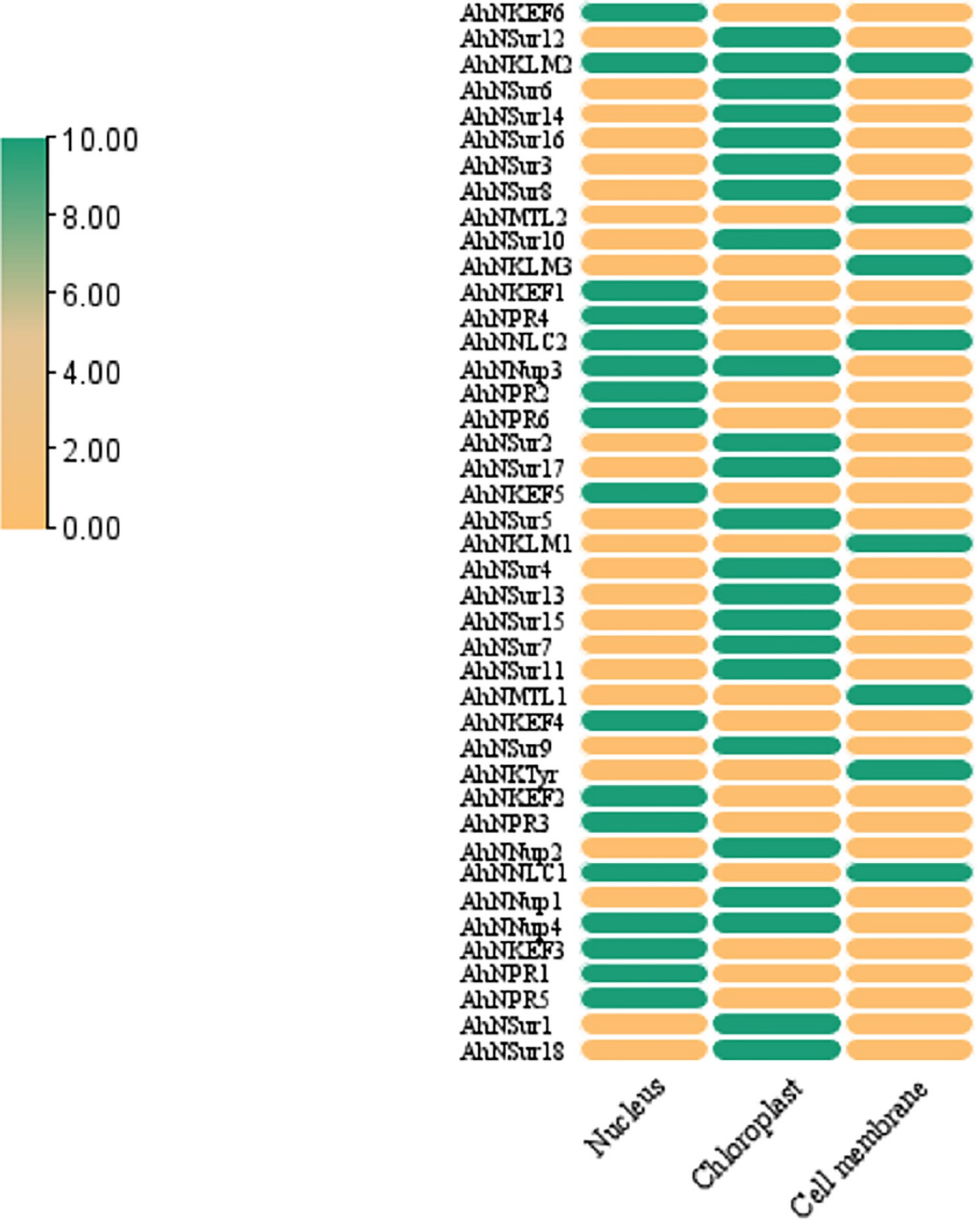
Protein locations of *NIG* family genes. The predicted locations of the proteins are nucleus, chloroplast and cell-membrane. The presence of protein on its respective location is represented by yellow colored boxes while their absence is indicated by green colored boxes.

The protein localization by Plant-mPLoc analysis predicted that 37 proteins of *NIG* family are located at single positions. For instance, these single location proteins are present in the nucleus, chloroplast and cytoplasm. While there are 5 proteins found in multiple positions. For instance, the proteins encoded by *AhNNup3* and *AhNNup4* genes are located in chloroplast-nucleus, while *AhNNLC1* and *AhNNLC2* encoded proteins are located in cell membrane-nucleus and the protein encoded by *AhNKLM2* gene is present in cell membrane-cytoplasm-nucleus (Fig 5).

The sequence logos of *NIG* family proteins could help to evaluate and discover the pattern of amino acid conservation in all 42 genes. Sequence logos of aligned amino acid residues of nodulation genes in *Arachis hypogaea* were generated to determine whether the *NIG* family proteins were conserved in all 42 genes throughout evolution (**S1 Fig**). The analysis showed that the protein sequences had moderate to high-level conservation at many different positions across N to C terminal.

### Retrieval of promoter regions and cis-acting elements analysis

**Fig 6:**
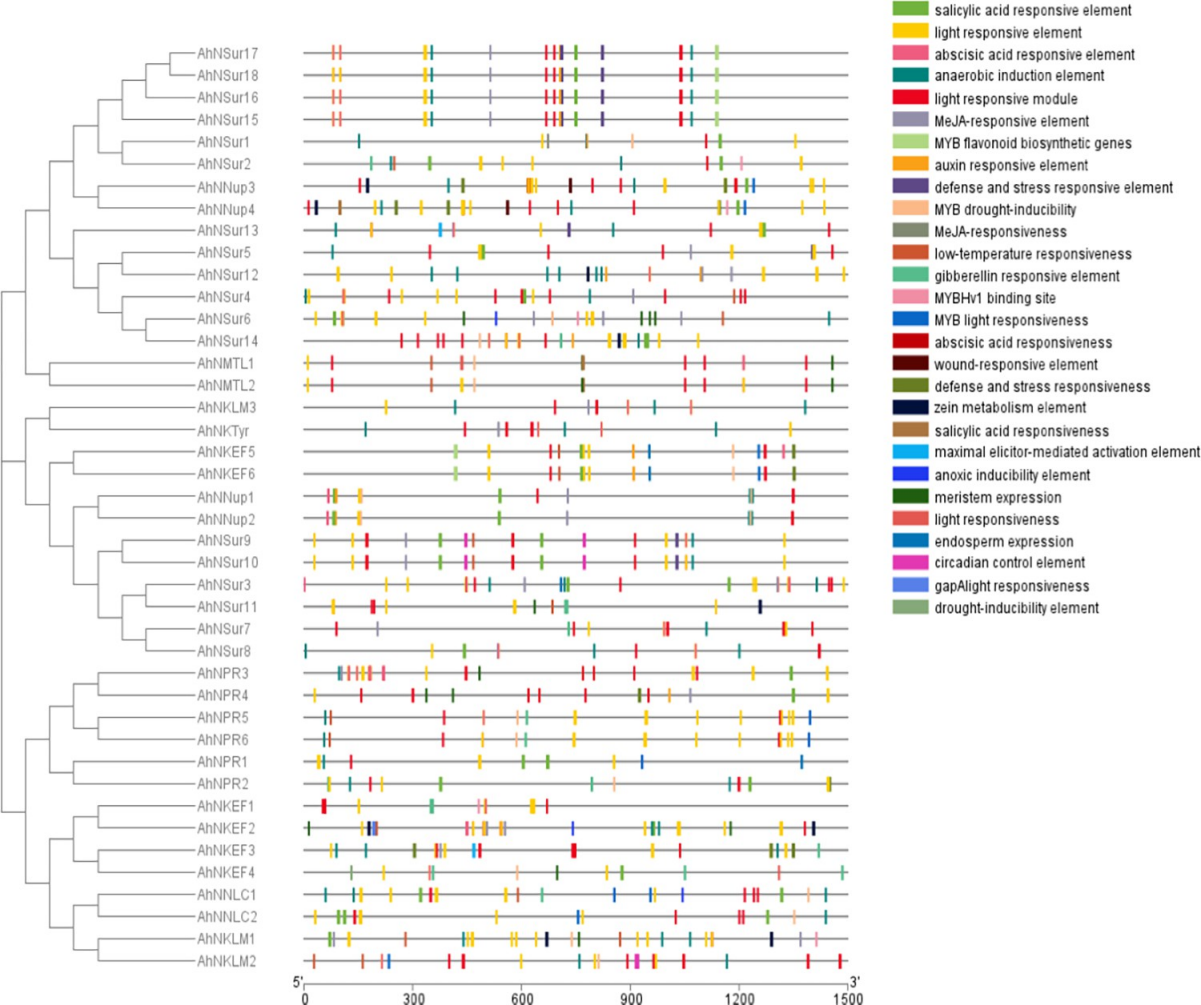
Analysis and frequency of occurrence of cis-acting elements related to stress responses, hormonal regulations and cellular development in *NIG* promoter regions of *Arachis hypogaea*.

To evaluate the transcriptional regulation of NIG gene family in response to different environmental conditions, promoters and cis-acting regulatory elements of candidate genes have been identified. Hence, 1500 bp upstream of the start codon was selected for identification of putative cis-acting regulatory elements (CAREs) **(S3 Text)**. The study revealed a total of 55 kinds of cis-acting regulatory elements across all *Arachis hypogaea* nodulation related genes. However, the CAREs length varies from 5–13 bp. Fig 6 represents the frequency of occurrence of each cis-acting element in each gene.

**Fig 7:**
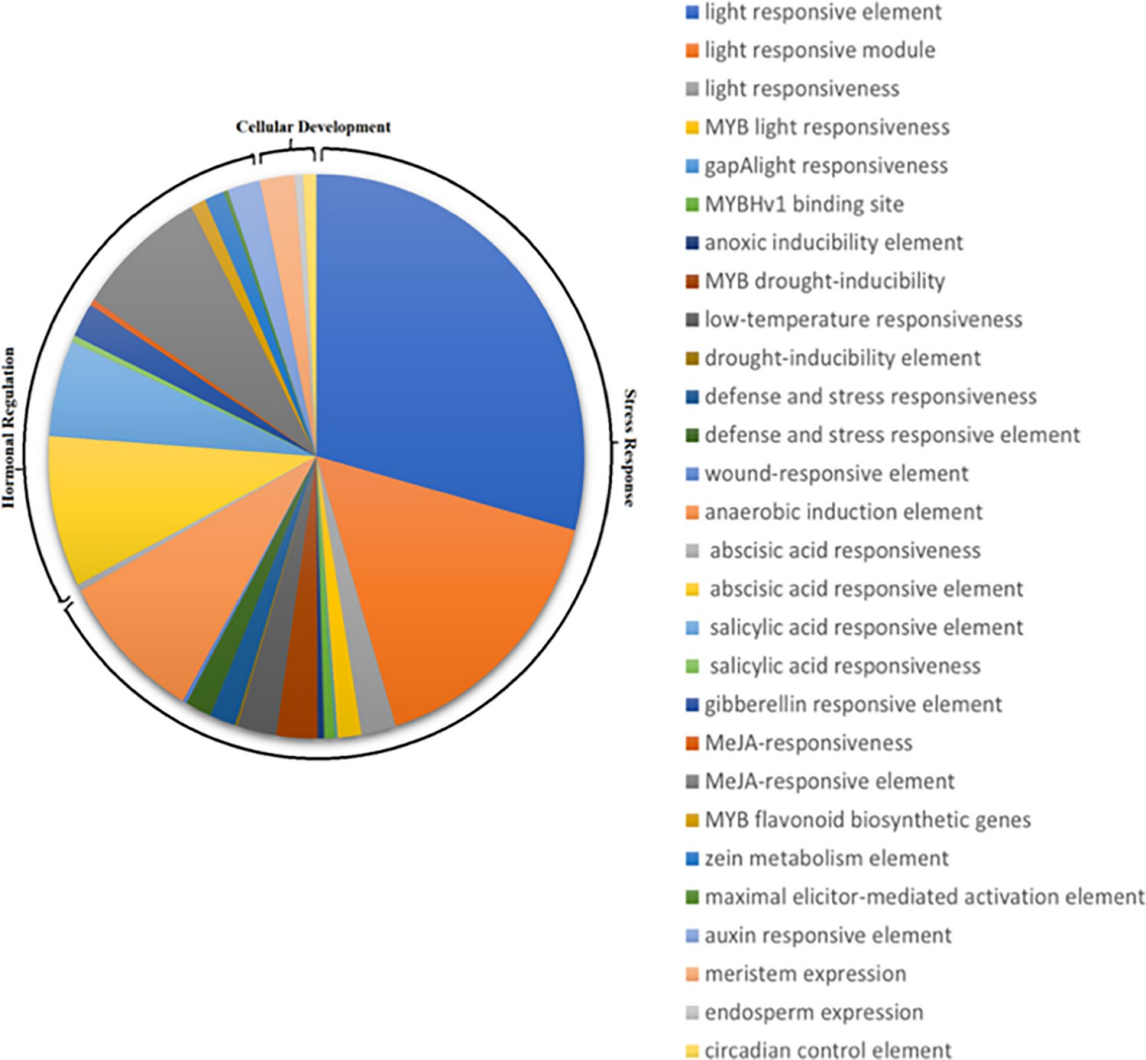
Pie distribution of identified cis-elements of *Arachis hypogaea NIG* family genes from PlantCARE, on the basis of their biological functions.

**Fig 8:**
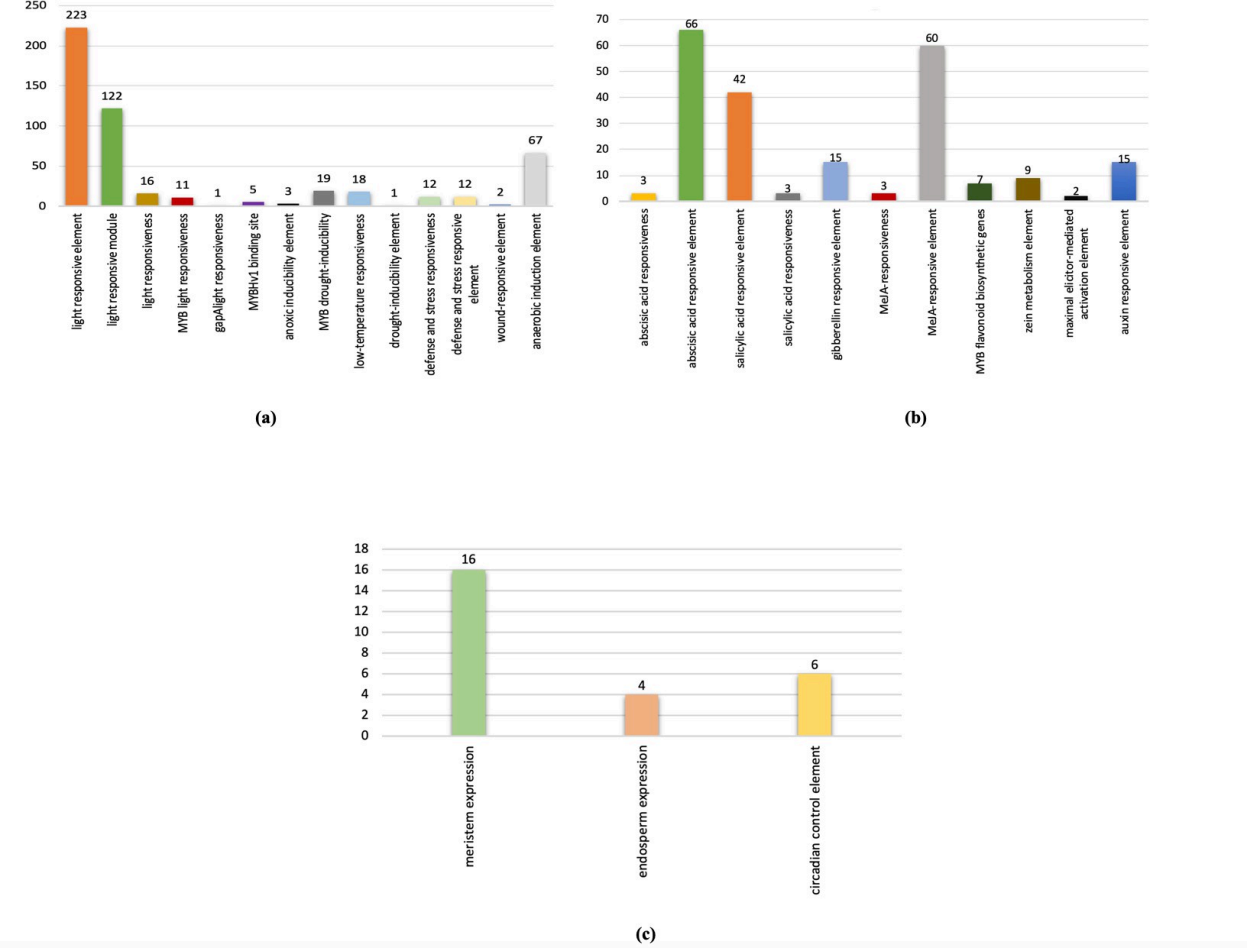
Histogram showing the number of cis-elements in *NIG* family genes, (a) is representing the number of cis-elements of cellular development, (b) is representing the number of cis-elements of hormonal regulation and (c) is representing the number of cis-elements of cellular development, (c) is representing the number of cis-elements of stress responses.

The cis-acting elements of all the candidate genes were grouped into functional categories as show in Fig 7. Among these identified cis-acting elements, common elements, such as TATA-box and CAAT-box, were present in abundance and were shared by all *NIG* genes. In addition, other identified cis-acting regulatory elements were associated with environmental stress-related factors, hormonal regulation and cellular development. For instance, among all the identified cis-acting elements, the motifs such as ACE, GT1-motif, AE-box, GCC-box, ATC-motif, I-box, Box II, Box III, Box 4, chs-CMA1a, chs-CMA2a, CCAAT-box, MBS, LTR, ABRE, O_2_ site, G-box, GATA motif, GA motif and LAMP-element etc. are associated with stress responses like low-temperature, light, oxidation, defense, wound, drought, anaerobic induction and anoxic response respectively. After, the stress-responsive motifs, motifs involved in hormonal regulation were found to be the second largest in number. Motifs such as, CGTCA and TGACG (methyl-jasmonate), ABRE (abscisic acid), TCA element (salicylic acid), AuxRR-core (auxin responsiveness element) were considered as hormonal responsive elements in *NIG* genes. Similarly, the elements involved in cellular development are relatively fewer in number than hormonal and stress-sensitive elements. These cis-acting elements include CAT box, O_2_ site, GCN4_motif, circadian, MBSI and ARE etc. CAT box is involved in meristem expression responses. Zein metabolism is regulated by O_2_ site. GCN4_motif is involved in endosperm expression. The circadian motif plays an important role in controlling circadian rhythms. MBSI has a role in the regulation of flavonoid biosynthetic genes. And anaerobic induction is carried out by ARE motif. Furthermore, the analysis revealed that most of the *NIG* genes belonging to different groups contain a number of binding sites for a major class of plant transcription factor genes. For instance, CCAAT-box has a MYBHv1 binding site. The detailed information about the cis-elements, their sequences and functions are shown in **S1 Table**. However, the number of occurrences of each cis-element was predicted. All *NIG* genes contained 512 stress-responsive elements, among them light-responsive elements made up 29%, which represents that light-responsive elements are most abundantly found in *NIG* genes. Similarly, hormonal regulation is coordinated by 237 motifs and there are at least 26 element to control cellular as shown in Fig 8.

## Discussion

Many legume plants respond to rhizobium to form a symbiotic relationship and develop a structure on their roots, known as a nodule. A nodule is an organ in which rhizobium converts atmospheric N_2_ into ammonia by a unique process [35]. However, this type of symbiotic relationship is triggered by the release of flavonoids from plants which might act as chemo-attractant to rhizobium spp., the Nod genes and a signal which is generated in plants after the perception of Nod-Factors [36]. Nod factors activate several nodulation genes in the nitrogen-fixing plants, however, the characterization of Nod genes was reported in many legumes [7, 12, 17]. For instance, nodulation factor receptor genes like NFR1 and NFR5 are discovered in L. japonicus [12, 13] and LYK3 and NFP were discovered in M. truncatula1 [10]. Furthermore, in *L*. *japonicus* and *M*. *truncatula*, the receptor-like kinases SYMRK and DMI2 serve as co-receptors for NF signaling [17]. It was crucial to completely predict and understand the role of nodulation genes in *Arachis hypogaea*. *Arachis hypogaea* is 3^rd^ largest class of legumes and it plays an important role in the world agriculture economy [37]. In this study, we analyzed the evolutionary relationship of nodulation inducing genes (*NIG*) family in *Arachis hypogaea* and categorized the physiochemical properties, gene structure, motif analysis, protein localization, chromosomal location, duplication of genes and their selection pressure of *NIG* family genes. The nodulation genes in allotetraploid *Arachis hypogaea* [38] were analyzed to understand the function of *NIG* family genes in flavonoid exudation, nodule development and biological nitrogen fixation.

The phylogenetic tree divided NIG family genes into three sub-families NIG-a, NIG-b and NIG-c, where sub-family NIG-b was the largest with 16 members while the sub-family NIG-c was the smallest with 12 members while previously identified MtGRAS family divided nodulation genes into eight subfamilies [39] and *A*.*evenia* LysM-RLK nodulation gene family contained 18 members [40]. The role of these genes in nodulation was also supported by conserved amino acid residue analysis of *Arachis hypogaea* genes. These results showed that sequence logos were conserved at many regions among all the genes, exhibiting that *NIG* family genes remained conserved throughout the process of evolution. Moreover, in the phylogenetic tree the NIG genes which have a very close evolutionary relationship was clustered together, suggesting that they may play related functions in plant nodulation.

Further analysis revealed that the gene length of *NIG* family genes ranges from 543 bp to 3996 bp. It has been predicted that genes belonging to *NIG* family share similar gene structures and protein motif distribution as well as few conserved motifs which indicated that *NIG* gene family is more conserved. Here we also predicted that the proteins encoded by these genes have almost similar motifs hence they are associated with the specific functions of symbiotic relationships. For instance *L*. *japonicus* and *M*. *truncatula* has Nucleoporin [41, 42], RWP-RK in *Arachis duranensis* and *Arachis ipaensis* [43], EF-hand domain in soybean [44], LRR in L. japonicus [45] were found to be involved in nodulation.

*AhNup3* is the largest assumed protein (149014.72 Da) while *AhNSur4* is the gene whose molecular weight was the smallest (38749.59 Da). In addition, the proteins were found to be located at different cell organelles i.e., nucleus, chloroplast and cell-membrane. These proteins were either single or multilocus proteins.

Genes derived from the same ancestral genes are called orthologs they have same biological functions, while the genes resulted from single genes by duplication event are called paralogs. Paralogs encode proteins with dissimilar functions [25, 46, 47]. The duplicated genes are mainly participating in paralogous genes formation of families. Besides, the uneven distribution of *NIG* family genes on 20 *Arachis hypogaea* chromosomes indicated that during evolution *NIG* genes experienced duplication. Further, in MtGRAS gene family of model legume M. truncatula 17 genes were duplicated [39] while our investigations predicted seven duplicated gene pairs in *NIG* family. Furthermore, these genes are originated from segmental duplication events and none of the genes originated from tandem duplication. Hence segmental duplication contributed to functional divergence and gene family expansion.

Cis-regulatory elements present in the promoter regions of genes are considered to be responsible for controlling the environmental and developmental regulation of gene expression. We determined different types and numbers of cis-acting elements in NIG family genes promoters. Many genes contained MBS, ACE, Box 4, MRE, CCAAT-box, GATA-motif, AT1-motif, I-box, ACA-motif, ATC-motif, Box II, AAAC-motif, WUN-motif, GA-motif, GC-motif etc. And these elements might responsible to perform different functions of the gene families under this study. For instance, stress-related elements were in abundance which might play an important role in adapting to external environmental stresses such as, low temperature, drought, light and defense. Cis-elements controlling hormonal regulation was second the largest in number. In addition, few elements might play role in cellular development. Furthermore, different numbers of such cis-acting elements were also discovered in wild species (*A*. *duranensis* and *A*. *ipaensis*), representing, distinct functions of these genes, vital for plant growth and development [43].

## Conclusion

The function of many nodulation related genes of legumes has been determined, but the functions of nodulation genes in *Arachis hypogaea* are still not demonstrated. NIG family genes were identified and divided according to evolutionary lineages into three sub-families. The expansion of *NIG* family might be the result of segmental duplication. Whereas, biophysical properties depict that NIG family proteins are located at distinctive cellular compartments, which represents that the proteins or enzymes are correlated with specific functions. In addition, these genes have conserved motifs and gene structures which suggests that the genes have functional similarities. All 42 genes are unevenly mapped on the chromosome. Cis-element analysis revealed the role of *NIG* family genes display their pivotal role in stress responses, hormonal regulation and cellular development. Hence, the results provided by genomic and bioinformatics analysis of *NIG* family genes provides a piece of valuable information about the phylogenetic relationship, structure, and function of these family members. These results could help in developmental research and genetic improvement of nodulation genes in *Arachis hypogaea* and other valuable nitrogen-fixing plants to improve biological nitrogen fixation. However, functional characterization of genes is needed to confirm their role in the nitrogen fixation and their use in future research programs.

## Supporting information

S1 Text Sequences of 42 *Nod* inducing genes of *Arachis hypogaea*. (DOCX)

S2 Text Open reading frames or amino acid sequences of 42 Nod inducing genes of *Arachis hypogaea*. (DOCX)

S3 Text Putative promoter sequences of 42 *Nod* inducing genes of *Arachis hypogaea*. (DOCX)

S1 Table Cis-elements and their respective functions. (DOCX)

S1 Fig Sequence logos of *Arachis hypogaea* NIG genes. The C-terminal and N-terminal pf NIG gene domain are represented by using ‘N’ and ‘C’.
(TIF)
