# Supplementary material for: Genome wide identification and characterization of nodulation related genes in Arachis hypogaea
Source: PLoS One. 2022 Sep 9;17(9):e0273768. doi: 10.1371/journal.pone.0273768 (PMC9462762; doi:10.1371/journal.pone.0273768)
Supplement: S1 Text — (DOCX) [file pone.0273768.s001.docx]

**1.AhNMTL1**

CACCGAAATGCATGCGTCTCAGTAAATTGAGAGAATGTGGTGGTGGCAGC

GTAATCGTGTACGTAGAAGAATACGACACTTTGTTAATACTTTAGTGCAC

CTCAAAATAACAATAAGAAATCAACTGAGAATGCCGTTTCCAATTAGAAA

TGGAATTTGTGATGATCTCAGGTTGATGAATATGTATATAACAAATGTTA

CATTGCCCAACTTTAATTACTTGCAAGTTTAGAATAACGGAAGCTAGGGA

CAAGTTTTGATGAAGCTCAAAGTATAGCGTCTTCGTTTTAATTTTTAGAC

CAATTATATACTATACAAAAATACTAACGTAGCTCTTTTTTTTTTTTAAG

TTTTATTTACTGGTAACTCCTACTGTCCAAGTCAATGGGGGGTGCATGCT

CTTTCTGCCTCTGATTGAGCCACTTCTGATCATCGTGTTGATTTCTCATC

AGTTTCAAAGTTGAAAATCTGAAAGGGAATTACTGTTAAGCATAATATAA

GGATAGTTGCATTGACACTCTTGACAATACAATTATTTATTCATTTTATA

TTATTTAACTCTATCTCCACAAAAATACGTACAGAAGAAATAGTTCCTTT

TTTTTTGTCTTATGTTTAATATCTTATTTTCAATCATGTTGCATCCATCG

TTAGAGTGAAAGGGAGGGGGGAATGAAGGAAGAACAGGACCAATCAAGGA

CATTAATTACTCTCTCTAATTATATTAAATCACATAATCAAGTAAAGCAA

CAACAATTAATAAATATGAACCGAAGAAAACATATTTAACTAAGGATTTT

GTACATAGTGCAAGCTCCCTAGCTAGCTAACTCCTCTTCTATCTCCTTGT

TAGGAAGTTCCTACACGTTTCAAGTCAAACCCTTTATTCTTAATATCCAT

TCGAGTTCTATGATCTTCCATCTAACATTTGGATAAGTAATGATTTCAAA

TTCTTCCAATAGAAGAATTTTTATTGAGGTTCATGGTAGTAGTATACTGT

AGGGTTGACCAAGGTGCCTTAGTTTTTGTTATCTGAATTAATAGTAGGAC

TAGTGAGTGTTGGTTGCATAAGGCAGAGAAAGAAGTGAGAAATTCAAGAG

ATTTGTATAGTGGCATGGCTTCATGTTTAGCAACCCCATGGGGTAACCTA

TAATGATGGAGAAATCAGATAATTTGGTATTATTAAGACAGGTTGTGCCA

TATGTCTTATGTTTGTGCATATTTATCAGATCAGCTTCTGCAAGTGAAGG

ATTTGAGAGCATAGCATGCTGTGCTGATTCAAACTATAAAGATCCATTGA

CCAACTTAGATTACAAAACAGATTATGCTTGGTTCTCTGATACAAGAAGT

TGCAGGCCAATAACTAGTGTGTTAAAACATTCAGCCTATGGAAGATTAAG

AGTGTTTGACATAGAAAAGGGAAAGAGATGTTACAATTTGGCCACAACTA

AGGATCAAGTGTATCTGATAAGGGGCACATTTCCATCTGAAAATGAACCA

GGTAAAGGTTCCTTTGGTGTTTCTATAGGGGTAACAGTGTTAGGTACAGT

GAGATCATCATCGCAGGACTTGAGAATTGAAGGAGTTTTCAGAGCCACAA

AGAACAACACAGACTTTTGCCTAGTGACAGAAGAGGGTAACCCTTATATA

TCTCAGCTTGAGCTAAGATCAGTGTCTGAAGAGTATCTGCAGGGTTTGAA

TTCTAGTGTCCTGAAGCTGATCAACAGAAGTAATCTTGGGGGAAAAGAAG

ATGACATAAGGTACCCAATTGATCAAAGTGATAGAATCTGGAAAAGAACT

ACTACAAGTCCATACACTCCTATATCATTCAATATTAGCATTTTGGACCA

CAAATCTAATGTGACACCTCCTCTGAAAGTTCTACAAACAGCTTTGACTC

ACCCTGAAAGGTTGGAATTCAATCACAATGGCCTTGAGGTTAAGGAGGAT

TACGAGTACCTTGTGTTTCTCTACTTCCTTGAATTGAATAACAGTGTTAG

AGAAGGCCTAAGAGTGTTTGACATCTATGTTAACAGTGAGATTAAGAAGG

CTAGTTTTGATGTATTAAGTGAAGGGTCAAATTATAGACACATTGTGTTG

AATGCTTCTTCAGCAAATGGATCACTTAATCTAACATTGGTCAAGGCATC

TGGATCTGTGTTTGGACCCTCTTGTAATGCTTATGAGATCATGCAGGTGC

GACCATGGAAGCAAGAAACCAACCAAACAGATTTGGTGGCGATTTTGAAG

ATAAGAGAAGAACTAATCACTGAAAACCATGATAACAAAGTCCTACAAAG

CTGGACCGGTGACCCATGCATGCTTTTTGCATGGCAAGGAATATCATGTG

ATTTTTCCAATGATGCACCTGTTATCATTAAGCTGGATCTTTCCTTAAGT

AATCTGAAAGGACCACTTCCATCCAGTGTCACAGAGTTGACTAACTTACA

AATAATGAACCTTAGCCACAACAACTTCAATGGCCATATCCCCTCATTTC

CAAGTTCCTCAATGCTCACTTCACTAGATCTGAGATACAATGATCTTATG

GGGTCACTTCCACCGTCATATAACTCACTGCCACATTTAAAGTCAATATA

TTATGGCTGCAATGATCGCATGGACCATAAGCTTTCAGGAAACTTGAACA

TTTCAATCAATACAGATAATGGAAGTTGTCAGAAAGGGAATGATTTTCCA

CAACAAGTAGCAATTATTTCAGCCGTTGCATGTGGCTCTTTCTTGATTGC

TATGGCTGTTGGAACAATTTTCATTTATCGTTATAGACAGAGATTAAATC

CATTGGAAGCATTTGGAAGAAAAAGCAACCCAATGATAACAAATGTGATA

TTCTCATTGAACAGCATAGACGATTTCTTGATAAAATCTATTTCAATTCA

AGCATTCACTTTGGAATATATAGAGGTTGCAACCGAAAAGTACAACATTT

TGATAGGTGAAGGAGGCTTTGGTTCTGTCTACCGTGGCACCCTAGAAAAT

GGTCAAGTTGTGGCTGTGAAGGTTCGGTCAGCCACATCAACGCAAGGCAC

TCGAGAATTTGATAATGAGCTAAACCTGCTTTCTGCAATAAGGCATGAGA

ACCTGGTGCCTCTTCTTGGTTATTGTAATGAAAATGACCAACAAATTCTG

GTTTATCCTTTTATGTCCAATGGCTCTCTGCAAGATAGACTATATGGAGA

ACCTGCAAAGAGAAAAATACTAGACTGGCCAACCAGACTGTCTATTGCTC

TTGGTGCCGCCCGAGGTTTGGCATATTTGCACTCGTTCCCTGAGCGATCT

GTAATTCACAGGGATGTGAAATCAAGCAATATACTTTTGGATCACAGCAT

GTGTGCTAAGGTTGCAGATTTTGGTTTTTCAAAATATGCTCCACAGGAAG

GAGACAGTGCTGTTTCGCTTGAAGTAAGAGGAACTGCAGGATATCTTGAT

CCTGAGTACTACACAACCCAGCAATTATCTGCAAAGAGTGATGTCTTCAG

CTTTGGTGTGGTTCTTCTTGAAATTGTGAGTGGCCGGGAGCCTCTCAACA

TCCATAGACCGCGGAATGAGTGGAGTTTGGTTGAATGGGCTAAACCGTAC

ATAAGGGCATCAAAGATTGAGGAAATTGTGGATCCTGGCATCAAGGGAGG

GTACCATGCAGAAGCAATGTGGAGAGTGTTGGAAGTAGCACTGCAATGTG

TTGAACCTTTCTCAACAAATAGACCGGCCATGGATGACGTCATGCGCGAG

TTGGAGGATGCTCTGATCATAGAAAACAATGCATCGGAGTACATGAAGTC

CATAGAGAGCCTTGGATCCAACCGTTACTCTATTGTCATAGAGAAAAGGG

TGCCACCCTCGACCTCGTCAATGGTAGAATCAACCATCATACCTCAATCC

TTGAACCATCCGCAGCCGAGGTAGTATAAGGAAAAAACACTTGAGATGCA

CTTTCAGGGACTCCATAACGCATCTGGAAACTAAAATAATAGTTGTCTAG

GACACTGGCATTCAAAGCTCCAGTTTTATTGTGACTCAATGTTTTCATCA

ATGCATTCAAATGGATAAACAAGTGGTAGGGTTGCCACATACAACTAGGA

AAAGATGCACCAACTTGCAAGTTTGACAACAGAATGCTCACTGTTTAACA

CAACTGCATTGACACCCCCTTCGTCAATTGTTTATGGGAGAGTACTAGTA

GCAATATAGTTTGTTGAATTGAGCTAAAAGTTCAGACTCGAATTGTTTTC

TTACACTTAGAATAGAGGCTGGTATACCCTTAAGTAAATCACGTTCTTGG

GTATTGAATTTTTATTTGATCTGAGTAGTAAATAATTGTATGGTAGGTGA

ATACAGATTAAAAATATGACTAATTTTTTTATATAGATTAAATATGCATT

TAATACATTGTTATTAGAAGTGTGGGTGTTTTTCTTTGATATGGAGTTTT

TTTTTA

**2.AhNMTL2**

ATCTCAGGTTGATGAATATGTATATAACAAATGTTACATTGCCCAACTTT

AATTACTTGCAAGTTTAGAATAACGGAAGCTAGGGACAAGGTTTGATGAA

GCTCAAAGTATAGCGTCTTCCTTTTAATTTTTGGACCTTCAATTATATAC

TATAAGAAAATACTAATGTAGCTCTTTTTTTTTTTGAGTTTTATTTACTG

GTAACTCCTACTGTCCAAGTCAATGGGGGGTGCATGCTCTTTCTGCCTCT

CATTGAGCCACTTCTGATTATCGTGTTGATTTCTCATCAGTTTCAAAGTT

GAAAATCTGAAAGGGAATTACTGTTAAGCATAACATAAGGATAGTTGCAT

TGACACTCTTGACAATACAATTATTTATTCATTTTATATTATTTAACTCT

ATCTCCACAAAAATACGTACAGAACAAATAGTTCCTTTTTTTTTTGTCTT

ATGTTTAATATCTTATTTTCAATCATGTTGCATCCATCGTTAAAGTGAAA

GGGAAGGGGGAATGAAGGAAGAACAGGACCAATCAAGGACATTAATTACT

CTCTAATTATATTAAATCACATAATCAAGTAAAGCAACAAACAATTAATA

AATAAACCGAAGAAAACATATTTAACTATGGATTTTGTACATAGTGCAAG

CTCCCTAGCTAGCTAACTCCTCTTCTATCTCCTTGTTAGGAAGTTCCTAC

ACGTTTCAAGTCAAACCCCTTTATTCTTAACATCCGTTCGAGTTCTATGA

TCTTCCATCTAACATTTGGATAAGTAATGATTTCAAATTCTTCCAAATAG

AAGAATTTTTATTGAGGTTCATGGTAGTAGTATACTGTAGGGTTGACCAA

GGTGCCTTAGTTTTTGTTATCTGAATTAATAGTAGGACTAGTGAATGTTG

GTTACATAAGGCAGAGAAAGAAGTGAGAAATTCAAGAGATTTGTATAGTG

GCATGGCTTCATGTTTAGCAACCTCATGGGGTAACCTATAATGATGGAGA

AATCAGATAATTTGGTATTATTAAGACAGGTTGTGCCATATGTCTTATGT

TTGTGCATATTTATCAGATCAGCTTCTGCAATTGAAGGATTTGAGAGCAT

AGCATGCTGTGCTGATTCAAACTATAAAGATCCATTGACCAACTTAGATT

ACAAAACAGATTATGCTTGGTTCTCTGATACAAGAAGTTGCAGGCCAATA

ACTAGTGTGTTAAAACATTCAACCTATGGAAGATTAAGAGTGTTTGAAAT

AGAAAAGGGAAAGAGATGTTACAAATTGGCCACAACTAAGGATCAAGTGT

ATCTGATAAGGGGCACATTTCCATCTGAAAATGCACCAGGTAAAGGTTCC

TTTGGTGTTTCTATAGGGGTAACAGTGTTAGGTACAGTGAGATCATCATC

GCAGGACTTGAGAATTGAAGGAGTTTTCAGAGCCACAAAGAACAACACAG

ACTTTTGCCTAGTGACAGAAGAGGGTAACCCTTATATATCTCAGCTTGAG

CTAAGATCAGTGTCTGAAGAGTATCTGCAGGGTTTGAATTCTAGTGTCCT

GAAGCTGATCAACAGAAGTAATCTTGGGGGAAAAGAAGATGACATAAGGT

ACCCAATTGATCAAAGTGATAGAATCTGGAAAAGAACTACTACAAGTCCA

TACACTCCTATATCATTCAATATTAGCATTTTGGACCACAAATCTAATGT

GACACCTCCTCTGAAAGTTCTACAAACAGCTTTGACTCACCCTGAAAGGT

TGGAATTCAATAACAATGGCCTTGAGGTTAAGGAGGATTACGAGTACCTT

GTGTTTCTCTACTTCCTTGAATTGAATAACAGTGTTAGAGAAGGCCAAAG

AGTGTTTGACATCTATGTTAACAGTGAGATTAAGAAGGCTAGTTTTGATG

TATTAAGTGAAGGGTCAAATTATAGACACATTGTGTTGAATGCTTCTTCA

GCAAATGGATCACTTAATCTAACATTGGTCAAGGCATCTGGATCTGTGTT

TGGACCCTCTTGTAATGCTTATGAGATCATGCAGGTGCGACCATGGAAGC

AAGAAACCAACCAAACAGATTTGGAGGTGATTTTGAAGATAAGAGAAGAA

CTAATCACTGAAAACCATGATAACAAAGTCCTACAAAGCTGGACCGGTGA

CCCATGCATGCTTTTTGCATGGCAAGGAATATCATGTGATTTTTCCAATG

GTGCACCTGTTATCACTAAGCTGGATCTTTCCTTAAGTAATCTGAAAGGA

CCACTTCCATCCAGTGTCACAGAGTTGACTAACTTACAAATTATGAACCT

TAGCCACAACAACTTCAATGGCTATATCCCCTCATTTCCAAGTTACTCAA

TGCTCACTTCACTAGATCTGAGATACAATGATCTTATGGGGTCACTTCCA

CCGTCATATAACTCACTGCCACATTTAAAGTCAATATATTATGGCTGCAA

TGATCGCATGGACCATAAGCTTTCAGGAAACTTGAACATTTCAATCAATA

CAGATAATGGAAGTTGTCAGAAAGGGAATGATTTTCCACAACAAGTAGCA

ATTATTTCAGCCGTTGCATGTGGCTCTTTCTTGATTGCTATGGCTGTTGG

AACAATTTTCATTTATCGTTATAGACAGAGATTAAATCCATTGGAAGCAT

TTGGAAGAAAAAGCAACCCAATGATAACAAATGTGATATTCTCATTGAAC

AGCATAGACGATTTCTTGATAAAATCTATTTCAATTCAAGCATTCACTTT

GGAATATATAGAGGTTGCAACCGAAAAGTACAACATTTTGATAGGTGAAG

GAGGCTTTGGTTCTGTCTACCGTGGCACCCTAGAAAATGGTCAAGTTGTG

GCTGTGAAGGTTCGGTCAGCCACATCAACGCAAGGCACCCGAGAATTTGA

TAATGAGCTAAACCTGCTTTCTGCAATAAGGCATGAGAACCTGGTGCCTC

TTCTTGGATATTGTAATGAAAATGATCAGCAAATTCTGGTATATCCTTTT

ATGTCCAATGGCTCTCTACAAGATAGACTATATGGGGAACCTGCAAAGAG

AAAAATACTAGATTGGCCAACCAGACTGTCTATTGCTCTTGGTGCCGCTC

GAGGTTTGGCATATTTGCACTCGTTCCCTGAGCGATCTGTAATTCACAGG

GATGTGAAATCAAGCAATATACTTTTGGATCACAGCATGTGTGCTAAGGT

TGCAGATTTTGGTTTTTCAAAATATGCTCCACAGGAAGGAGACAGTGCTG

TTTCGCTTGAAGTAAGAGGAACTGCAGGATATCTTGATCCTGAGTACTAC

ACAACCCAGCAATTATCTGCAAAGAGTGATGTCTTCAGCTTTGGTGTGGT

TCTTCTTGAAATTGTGAGTGGCCGGGAGCCTCTCAACATCCACAGACCGC

GGAATGAGTGGAGTTTGGTTGAATGGGCTAAACCGTACATAAGGGCATCA

AAGATTGAGGAAATTGTGGATCCTGGCATCAAGGGAGGGTACCATGCAGA

AGCAATGTGGAGAGTGTTGGAAGTAGCACTGCAATGTGTTGAACCTTTCT

CAACAAATAGACCGGCCATGGATGACGTCATGCGCGAGTTGGAGGATGCT

CTGATCATAGAAAACAATGCATCAGAGTACATGAAGTCCATAGAGAGCCT

TGGATCCAACCGTTACTCTATTGTCATAGAGAAAAGGGTGCCACCCTCGA

CCTCGTCAATGGTAGAATCAACCATCATACCTCAATCCTTGAACCATCCG

CAGCCGAGGTAGTATAAGGAAAAAACACTTGAGATGCACTTTCAGGGACT

CCATAACGCATCTGGAAACTAAAATAACAGTTGTCTAGGACACTGGCATT

CAAAGCTCCAGTTTTATTGTGACTCAATGTTTTCATCAATGCATTCAAAT

GGATAAACAAGTGGTAGAATTGCCACATACAACTAGGAAAAGATGCACCA

ACTTGCAAGTTTGATGACAGAATGCTCACTGTTTAACACAACTGCATTGA

CACCCCCTTCGTCAATTGTTTATGGGAGAGTACTACTGTACTAGTAGCAA

TATAGTTTGTTGAATTGAGCTAAAAGTTCAGACTCGGATTGTTTTCTTAC

ACTTAGAATAGAGGCTGGTATACCCTTAAGTAAATCACGTTCTTGGGTAT

TGAATTTTTATTTGATCTGAGTAGTAAATAATTGTATGGTAGGTGAATAC

AGATTAAAAATATGACTAATTTTTTTATATGGATTAAATATGCGTTTAAT

ACATTGTTATTAGAAGTGTGGGTGTTTTTCTTTGATATGGAGTTTTTTTT

AA

**3.AhNKEF1**

TTTTTCCTAGTCTTTTTCTGTTATTATTATATTATTATAAGAGGTAAGGT

GTACTTAATACCAAAAGGTGATTTACTTTTTCTCAACTAAACTTACCAAC

GTTCACACAAACTGCCCCCTTTTGTCTCGCTAGCCTCGCTTCCCCGCCGG

GAAAACCCAACTGGCGGGACAAACCCGCCGCTCTGGTCTGTATTCAGTGG

TCACTCCCAACTCTAAATTAAACCTCCAAAACCGAATAAAACAAGAAAAT

AAACAAATAAAAAGAAAAAGGATGGGAAACTGCAACGCTTGCGCCAGAGC

AGATGTAGTAGACAGCACAACAACCACAATCACAAAGCCTACCACCAACA

ACCGAAAACCGAACCCCTTTTCCTCCACTCCGGCCCGGCCAGCCAACCCG

ATCCGCGTCCTCAACAACGACATCCCCGCGGGCCCGCAGCACCGGGCCCG

AATCAGCGACGATTACATACTTGGCCGTGAGCTGGGCCGCGGCGAATTCG

GCATAACCTACCTCTGCACTGACCGCGAGACCAAAGAAGCTCTCGCATGT

AAGTCCATCTCGAAGCGGAAGCTCCGCACCGCTGTCGACGTAGACGACGT

GCGGCGCGAGGTGAAGATCATGTCCACGCTGCCGCCGCATCCGAACGTGG

TGCAGCTGAAGGGAGCTTACGAGGACGACGAGAACGTTCACATAGTTATG

GAGCTCTGTGAGGGCGGCGAGCTCTTCGACAGGATCGTTGCCAGGGGACA

CTACAGCGAGCGCGCCGCCGCCGGAGTGTTCCGGACGATCGCGGAGGTTG

TTAGGATGTGCCACGCTAACGGTGTCATGCATAGAGACCTCAAGCCTGAG

AATTTCTTGTTTGCTAACAAGAAGGAGAATTCGCCCCTCAAGGCCATTGA

TTTTGGCTTGTCTGTGTTCTTCAAGCCAGGGGAGAGGTTTTCGGAGATTG

TGGGGAGTCCTTACTACATGGCGCCGGAGGTATTGAAGAGGAATTATGGG

CCGGAGGTAGATGTGTGGAGTGCTGGTGTGATCCTTTATATTTTGCTCTG

TGGGGTTCCTCCGTTTTGGGCAGAGACGGAGCAAGGTGTGGCTCTGGCAA

TATTGAGGGGGGTGATTGATTTCAAGAGGGAACCTTGGCCACATATATCA

GATAGCGCTAAAAGCCTTGTCCGGCAGATGTTGGAACCAGATCCTAAAAA

GCGCTTGACTGCTGAACAGGTGCTTGGTGGGAACTGGGAAGGAGATTGTG

GAAAATTTTGGAATGAAATCACCTTAGACTTTCTTGCACATCCATGGCTA

CAAAATGCCAAGAAAGCTCCAAATGTTCCATTAGGTGATCTTGTGAGGTC

AAGGCTTAAGCAGTTTTCTATGATGAATAGATTCAAAAAGAAAGCTCTTC

GGGTAATTGCAGATCATTTATCTGTTGAAGAGGTAGAAATAATCAAAGAT

ATGTTTACATTGATGGACACCGACAAAGACGGACGAGTAACATACGAAGA

ACTAAAGGCTGGGTTGAGGAAAGTTGGTTCACAATTGGCTGAGCCAGAGA

TAAAGTTGCTGATGGAAGTGGCCGATGTCGATGGGAATGGAATACTTGAC

TATGGGGAGTTTGTAGCTGTTACCATTCACTTGCAAAAAATGGAGAACGA

TGAGCATTTCCGCAAAGCATTCAAGTATTTTGACAAAGACAGTAGTGGTT

ATATTGAGTTTGGTGAACTACAGGAAGCATTAGCAGATGAGTCAGGAGAA

ACTGATCATGATGTTTTGAATGACATCATGCGTGAAGTTGACACTGATAA

GGATGGTCGCATCAGTTTTGAGGAGTTTGTTGCCATGATGAAAACTGGAA

CTGACTGGAGAAAAGCATCTAGGCAATATTCAAGGGAGAGATTCAAGAGT

TTGAGCCTAAACTTGATGAAAGACGGCTCTCTTCAGCTTCATGACGGTAT

CAGTGGTCAAGCTGTAGTGGTTTAATTATATTATTGTTGTTAATATTATT

GTTTTATATAACCTCAGTGCTCTCTAGGTTTTTCTTATTCCTTTCCACTG

CTTACACTGCCTTAAGATATGGCTTATTTAATCTCGCCGTGAAATCAGCT

TTTGGGATGGTTTACCTATGAACATTTGAACCATACTAATGAGCTTGAGT

TGGTTTTGCGTAGCTTAGGTTTCTTACATGCCTGAAGCATGCTTTGGGGG

AATACAATTGCATACTAATGATGGTTTATAGTTTGGCAGCAAAAATGTCG

GGGCAGATAGTATAGGCCTAAAAGATGTTACATATTCTTTGTATATCACA

CTGTGCTGTTATATTTGTATTTTTAATTCAATTGGTAAGTGTGATTTTCC

TTACTTGTATGCAGAAAACGACTTAAAGCTTGTCTCATTGGTTAGATGAA

GTTATAACTTTTTATAGAACTAGTCGAACAATCTGCGCAGAGCGCAAGGT

TGTGTGATTAATTTGTGTTGATAAGATAATCATAACTCAGTTCTAGCAAA

TACGGGATATTTTGATTTTTGTGTTTTGATTTATGCATGCATGGGTCACC

ATGGTATCTTAGAGCATATTAGTTGTGTATAATTATGCATGATCATGCAT

AAGGAAGAAGAACAAAGCATAGTTATCACAACCTAACCAAACTAGTGGTT

TGACCAGGTCGATTGTGGGTTTGGTTTTGATAACTATGAAAGAAAGTCAT

GCAGCTTTTTGCTTGCTCACTCAAGGAAGAGAACACAAATGTTATCCCAC

ATTTGTTTAATAAAATGTTTCTCGGATTTGTTTTCTCTGCCATCATCTCC

TCCAAGTTGGCAACAGGTCATTGGTTGACGATTTGTTCGCACTTGACGTA

CGTGTGAAGGTTTGCAGAGCTTGAAGCTAAGGGAGTTGCAGCCTATAGAA

CTGTAAATTATCCAACTTGTTTTGAGTCACACCCGCCGAGTCACTTTGTC

CTTCCAACCAACGTCGTTTGCCTGGTCGTCCTTTGCTGCATGACTATTGC

CCCCTTGTCTTCGTGCGATTGCCGACAATGCTTTCAATATGTAATCCTAT

CTGTAGTTGTTAGTTTCATTCCTTGTTTATTTACATTAAAGAATAAGAGA

GAGAGTTGAATGTTTTTATTAGAAGAAAAGGGAAGACTTTGTTTTTTTTT

TTTTAAACAAGAAGGCGACGTATCTCCTTATTGGTTAGAGTGGTAGATTC

TTTCTCCTAAATTAGTGGTTTGAATTCAAGATTTGGATATCGAAAATTCA

TGTTCGGAGAGTCATGTCTCTTGTAGAATATCAAATTGTTCAAATATGAT

TGCATCTAACGAGAATATTTATGGTTTCATAGAAAATAGAGTAAATACTC

AGTTTGGTCTTGGTCTTTAAAATATTCTAGAAGACTTTAATCTCTAACAT

TATTTCACGAGTCTCCAACATCTCCGCTACTTTGCTTTGCCAAGTCCTAA

CAAATCTGTGCGATGTGTACTTTTTTGGTGTTTTCGATGTTAAAGGGACA

CATGATTAAGATGGTAAACTAGTTTAATGTCCCGTATTTGCAC

**4.AhNKEF2**

GATTTACTTTTTCTCAACTAAACTTACCAACGTTCACACAAACTGCTCCC

TTTTGTCTCGCTAGCCCCGCTTCCCCGCCGGGAAAACCCAACTGCCGGGA

CAAACCCACCGCTCTGGTCTGTATTCAGTGTTCACTCCTAACTCTAAATT

AAACCTCCAAAACCGAACAAAACAACAAAATAAACAAATAAAAAGAAAAA

AGGATGGGAAACTGCAACGCTTGCGCCAGAGCAGATGTAGTAGACAGCAC

AACAACCACAACCACAAAGCCTAACACCAACAACCGTAAATCGAACCCCT

TTTCTTCCACTCCGGCCCGGCCAGCCAACCCGATCCGCGTCCTCAACAAC

GACATCCCCGCGGGCCCGCAGCACCGGGCCCGAATCAGTGACGATTACAT

ACTTGGCCGTGAGCTGGGCCGCGGCGAATTCGGCATAACCTACCTCTGCA

CTGACCGCGAGACCAAAGAAGCTCTGGCATGTAAGTCCATCTCGAAGCGG

AAGCTCCGCACCGCCGTCGACATAGACGACGTGCGGCGCGAGGTGAAGAT

CATGTCCACGCTGCCGCCGCATCCGAACGTGGTGCAGCTGAAGGGAGCTT

ACGAGGACGACGAGAACGTTCACATAGTTATGGAGCTCTGTGAGGGCGGC

GAGCTCTTCGATAGGATCGTTGCCAGGGGACACTACAGCGAGCGCGCCGC

CGCCGGAGTGTTCCGGACGATCGCGGAGGTTGTTAGGATGTGCCACGCTA

ACGGTGTCATGCATAGAGACCTCAAGCCTGAGAATTTCTTGTTTGCTAAC

AAGAAGGAGAATTCGCCCCTCAAGGCCATTGATTTTGGCTTGTCTGTGTT

CTTCAAGCCAGGGGAGAGGTTTTCGGAGATTGTGGGGAGTCCTTACTACA

TGGCGCCGGAGGTATTGAAGAGGAATTATGGACCGGAGATAGATGTGTGG

AGTGCTGGTGTGATCCTTTATATTTTGCTCTGTGGGGTTCCTCCGTTTTG

GGCAGAGACGGAGCAAGGTGTGGCTCTGGCAATATTGAGGGGGGTGATTG

ATTTCAAGAGGGAACCTTGGCCGCATATATCAGATAGCGCTAAAAGCCTT

GTCCGGCAGATGTTGGAACCAGATCCTAAAAAGCGCTTGACTGCTGAACA

GGTGCTTGGTGGGAACTGGGAAGGAGATTGTGGAAAATTTTGGAATGAAA

TCACCTTAGACTTTCTTGCACATCCATGGCTACAAAATGCCAAGAAAGCT

CCAAATGTTCCATTAGGTGATCTTGTGAGGTCAAGGCTTAAGCAGTTTTC

TATGATGAATAGATTCAAAAAGAAAGCTCTTCGGGTAATTGCAGATCATT

TATCTGTTGAAGAGGTAGAAATAATCAAAGATATGTTTACATTGATGGAC

ACCAACAAAGACGGAAGAGTAACATACGAAGAACTAAAGGCTGGGTTGAG

GAAAGTAGGTTCACAATTGGCTGAGCCAGAGATAAAGTTGCTGATGGAAG

TGGCCGATGTCGATGGGAATGGAATACTTGACTATGGGGAGTTTGTAGCT

GTTACCATTCACTTGCAAAAAATGGAGAACGATGAGCATTTCCGCAAAGC

ATTCAAGTATTTTGACAAAGACAGTAGTGGTTATATTGAGTTTGGTGAAC

TACAGGAAGCATTAGCAGATGAGTCAGGAGAAACTGATCATGATGTTTTG

AATGACATCATGCGTGAAGTTGACACTGATAAGGATGGTCGCATCAGTTA

TGAGGAGTTTGTTGCCATGATGAAAACTGGAACTGACTGGAGAAAAGCAT

CTAGGCAATATTCAAGGGAGAGATTCAAGAGTTTGAGCCTAAACTTGATG

AAAGACGGCTCTCTTCAGCTTCATGACGGTATCAGTGGTCAAGCTGTAGT

GGTTTAATTATATTATTATTGTTAATATTATTATTTTATATTACCTCAGT

GCTCTCTAGGTTTTTCTTATTCCTTTCCACTGCTTACACTGCCTTAAGAT

ATGGCTTATTTAATCTCGCCGTGAAATCAGCTTTTGGGAAGGTTTAGCTA

TGAACATTTGAACCATACTAATGAGCTTGACTTGGTTTTGCGTAGCTTAG

GTTTCTTACATGCCTGAAGCATGCTTTGGGGGAATACAATTGCATACTAA

TGATGGTTTATAGTTTGGCAGCAAAAATGTCGGGGCACACAGTATAGGCC

TAAAAGATGTTACATATTCTTTGTATATCACTGTGCTGTTATATTTGTAT

TTTTAATTCGATTGGTAAGTGTGATTTTCCTTACTTGTATGCAGAAAACG

ACTTAAAGCTTGTCTCATTGGTTAGATGAAGTTATAACTTTTTCTAGAAC

TAGTCGAACAATCTGCGCAGAGCGCAAGGTTGTGTGATTAATTTGTGTTG

ATACTTGATAAGATAACCATAACTCTAGCAAATACGGGATATTTTGATTT

TTGTGTTTTGATATATGCATGCATGGGTCACCATGGTATCTTAGAGCATA

TTACTTGTGTATAATTATGCATGATCATGCATAAGGAAGAAGAACAAAGC

ATAGTTATCACAACCTAACCAAACCAGTGGTTTGACCAGGTTGATTGTGG

GTTTGGTTTTGATAACTATGAAAGAAAGTCATGCAGCTTTTTGCTTGCTC

ACTCAAGGAAGAGAACACAAATGTTATCCCACATTTGGTTAATAAAATGT

TTCTCGGATTTGGTTTCTCTTCCATCATCTCCTCCAAGTTGGCGACAGGT

CATTGGTTGACGATTTGTTCTCACTTGACGTACGTGTGGAGGTTTGCAGA

GCTTGAAGCTAAGGGAGTTGCAGCCTATAGAACTGTAAATTATCCAACTT

TGTTTCGAGTCACACCCGCCGAGTCACTTTGTCCTTCCAACCAACATCGT

TTGCCTGGTCGTCCTTTGCTGCATGACTATTGCCCCCTTGTCTTCGTGCG

ATTGCCGACAATGCTTTCAATATGTAATCCTATCTGCAGTTGTTAGTTTC

ATTCTTTGTTTATTTACATTAAAGAATGAGAGAGAGAGTTGAATGTTTTT

ATTAGAAGAAAAGGGAAGACTTTGTTTTTGTTTTTTTACAAGAAGGCGAC

GTATCTCCTTATTGGTTAGAGTGGTAGATTCTTTCTCCTAAATTAGTGGT

TTTGAATTCAAGATTTGGATATCGAAAATTCATGTTCGGAGAGTCATGTC

TCCTGTAGAATATCAAATTGTTCAAATATGATTGCATCTAACGAGAATAT

TTATGGTTTCATAGAAAATAGAATAAATACTCAGTTTGGTATTTAAAATA

TTCTAGAAGACTTTAACATTATTTCACAAGTCTCCAACATCTCCGCTACT

TTGCTTTGTCAAGTCCTAACAAATCTGCGCGATGTGTACTTTTTTGGTGT

TTTCGATGTTAAAGTGACACATGATTAAGATGGTGAACTAGTTTAATGTC

CCGTATTTGCAC

**5.AhNKEF3**

ACAGTAGACGCAGCTGGTCAGCACTGACCACCTCCGATGTTTCTCGGCAG

GAACCGCTACAAATATACAAATAGTCAAATACCATATCCATCCTAAAATT

AACAAATAACCCAAATATAAAAAGAGAAAAAGAAAAACTTATTTTCCGAA

GATCTGTCGGAGCTGCAGGAGAGAAATGAATTGAATTGAATGGAAAACAA

AGCATGAGAGGTTAATATTATCCATTCGTCCTCTTTCCTTTTCCATCCTC

ACCTTCTGCCTGTTCTGGATTTTGATCGTGACTCGTGAGCACAGCAGCTT

AGCGCATAAGAGAAACTGATGAGAGATTGTTGGATGAGGAAACGGGCGTA

ATACAGAATCGTATTGTTTGATGGGCAACACATGCCGTGGATCTTTGAGA

GGAAAATATTTTCAGGGCTTCAGCCAGCCCGAAGACCCCTCCAGGCGCAG

CAATCCTTCTGACCCCTCCGACTCAGAGCACCTCCCCAAGCAGAATCCCA

ATTCAGCAGACAACAACAACAGCAACAACAACAACAACAACAACAACAAC

ATCAACAACAACAAGAGAAATCTACCCTTCAAGAAGGACACCATCATGCG

CAGAGGCCCCGACAACCAAGCTTATTATGTCCTGGGTCATAAGACTCACA

ACATTCGTGATCTCTATACTCTTGGCCGCAAACTGGGTCAGGGCCAATTT

GGCACCACTTATTTATGCACCGAGAATTCCACCAACATCGAATACGCATG

TAAATCCATCTCCAAGAGGAAGTTGATTTCCAAGGAGGACGTGGAAGACG

TTAGGAGAGAAATTCAAATAATGCACCATTTAGCTGGTCACAAGAACATC

GTCACCATCAAGGGTGCTTATGAGGATCCTCTCTACGTCCATATTGTCAT

GGAGCTTTGTTCTGGGGGTGAGTTGTTTGATCGCATCATCCAAAGGGGCC

ACTACACCGAGAGGAAGGCTGCTGAGTTGACCAAAATCATTGTTGGAGTT

GTTGAGACTTGCCATTCCCTCGGGGTTATGCATAGAGATCTTAAGCCTGA

AAACTTCTTGTTGGTTAATAAGGATGATGATTTCTCTCTTAAAGCAATTG

ACTTTGGCCTCTCTGTTTTCTTCAAACCAGGTCAAGTATTCACTGACGTG

GTTGGCAGTCCGTACTATGTTGCTCCTGAGGTTCTCCTCAAGCATTATGG

CCCCGAAGCAGATGTGTGGACAGCGGGCGTCATTCTATACATATTACTTA

GTGGAGTGCCGCCGTTTTGGGCAGAAACCCAGCAGGGTATATTTGATGCT

GTTTTGAAGGGGCATATAGATTTCGACTCTGATCCATGGCCTTTAATTTC

TGATAGTGCTAAAGATCTGATTAGAAAGATGCTGTGTTCTCGGCCTTCAG

AACGGTTGACAGCTCATGAAGTGTTATGTCATCCATGGATATGTGAAAAT

GGAGTTGCCCCTGACAGAGCATTGGACCCTGCTGTTCTTTCCCGCCTTAA

ACAGTTCTCTGCAATGAACAAACTAAAGAAGATGGCTTTGCGGGTAATTG

CTGAAAGTTTGTCCGAAGAGGAGATTGCTGGTTTGAGAGAGATGTTTCAA

GCTATGGATACTGATAACAGTGGTGCAATTACTTTTGATGAGCTTAAAGC

TGGTCTAAGAAGATATGGGTCAACCCTTAAGGATACAGAAATACGTGATC

TTATGGAGGCGGCTGATGTGGACAATAGTGGGACTATAGATTATGGAGAG

TTTATCGCTGCAACAATTCATCTCAACAAGCTAGAGCGCGAGGAACATCT

CATTGCAGCATTTCGATATTTTGACAAGGATGGGAGTGGCTATATTACAG

TTGACGAACTTCAACAAGCTTGTATAGAACATAACATGACTGACGTTTTT

CTTGAAGATATTATTAGGGAAGTTGATCAAGATAATGATGGAAGGATTGA

TTATGGTGAATTTGCTGCCATGATGCAAGGCAATGCTGGAATTGGAAGGA

GGACTATGCGCAACAGCCTGAATTTAAGCATGAGAGATGCACCAGGTGTC

TGATTCTCATCGGTGCAGGTTGTACAGCAAAATCCAGAAAGAGAAAAATA

GAGCATGAATCTTTTATAAAGAAAAAGATTTGTTGCTATCCTGCACAGGG

ATCCACTCGCATGCAAGAAGAATGGGGGATCATAGATGCTGTCCCATGAA

CACTCCTCAATTGTTTGCATTTATGTGCTGTATGAATGATTGCGAAAGTT

ATTTTGATCTTTGTAAACTCCAGTTTCAACGTAACAGCTGTTTCAAGTTA

GTCGTTATTTTTCTGTACTTTCATGTAGGGGAATTCTGTTAATAGTCGAA

GTAGAAGGATTTGAAAGGTTTTGATTTTACGGATGTAGAGTTTTACACGA

GTGAAGTTGATGGTTTGGGCGAACTATGAAGCCTGTTTGGATGTGTTGAC

TGTTGATGTGAAAGTAGACACATAATATATCTCAGCTGGCTTGTTTTTCA

ATTAAATTTATGCCTTTATTAATAATAGTTAATTTTCCATACGTAATTTT

GAAATAGTCATCTAATCTATATTACGAACTACTGTATGGTGTTAAACTGG

ACTTAAAATTTATAATTTTTTATAAAAATTTGTATGGAGTAATGTATATG

CTGACTAAGCAACTTGACAACGAGTAATGAGGAGAAACTTCGATTTACTA

GAAATAATACAAATTTACGGTGCAATGAGTTGAGTTTATTCAAATTTAAA

TTTGACTCATTTATTATATGAGTTTAAGTTTGGTCACTAGCTCACGAATT

TAGCTTATCGAGCTATTAACGAGTCAAGTTCGAGCTAGTTCATGAGTTGG

CTTGACTCACTTCCAGTCTTAGTGATAGCTCTACCTAATTTCTTTGGCGG

GAGGAGTTTGGAAGCGACTATGCAAGATGCTACTGACGAAGGGAAGGGGA

TTAGGGTTCAGGGGTTCACCGTT

**6.AhNKEF4**

ACAGTAGACGCAGCTGGTCAGCACTGACCACCTCCGATGTTTCTCGGCAG

GAACCGCTACAAATATACAAATAGTCAAATACCATATCCATCCTAAAATT

AACAAATAACCCAAATATAAAAAGAGAAAAAGAAAAACTTATTTTCCGAA

GATCTGTCGGAGCTGCAGGAGAGAAATGAATTGAATTGAATGGAAAACAA

AGCATGAGAGGTTAATATTATCCATTCGTCCTCTTTCCTTTTCCATCCTC

ACCTTCTGCCTGTTCTGGATTTTGATCGTGACTCGTGAGCACAGCAGCTT

AGCGCATAAGAGAAACTGATGAGAGATTGTTGGATGAGGAAACGGGCGTA

ATACAGAATCGTATTGTTTGATGGGCAACACATGCCGTGGATCTTTGAGA

GGAAAATATTTTCAGGGCTTCAGCCAGCCCGAAGACCCCTCCAGGCGCAG

CAATCCTTCTGACCCCTCCGACTCAGAGCACCTCCCCAAGCAGAATCCCA

ATTCAGCAGACAACAACAACAACAGCAACAACAACAACAACATCAACATC

AACAACAACAAGAGAAATCTACCCTTCAAGAAGGACACCATCATGCGCAG

AGGCCCCGACAACCAAGCTTATTATGTCCTGGGTCATAAGACTCACAACA

TTCGTGATCTCTACACTCTTGGCCGCAAACTGGGTCAGGGCCAATTTGGC

ACCACTTATTTATGCACCGAGAATTCCACCAACATCGAATACGCATGTAA

ATCCATCTCCAAGAGGAAGTTGATTTCCAAGGAGGACGTGGAAGACGTTA

GGAGAGAAATTCAAATAATGCACCATTTAGCTGGTCACAAGAACATCGTC

ACCATCAAGGGTGCTTATGAGGATCCTCTCTACGTCCATATTGTCATGGA

GCTTTGTTCTGGGGGTGAGTTGTTTGATCGCATCATCCAAAGGGGCCACT

ACACCGAGAGGAAGGCTGCTGAGTTGACCAAAATCATTGTTGGAGTTGTT

GAGACTTGCCATTCCCTCGGGGTTATGCATAGAGATCTTAAGCCTGAAAA

CTTCTTGTTGGTTAATAAGGATGATGATTTCTCTCTTAAAGCAATTGACT

TTGGCCTCTCTGTTTTCTTCAAACCAGGTCAAGTATTCACTGACGTGGTT

GGCAGTCCGTACTATGTTGCTCCTGAGGTTCTCCTCAAGCATTATGGCCC

CGAAGCAGATGTGTGGACAGCGGGCGTCATTCTATACATACTACTTAGTG

GAGTGCCGCCGTTTTGGGCAGAAACCCAGCAGGGTATATTTGATGCTGTT

TTGAAGGGGCATATAGATTTCGACTCTGATCCATGGCCTTTAATTTCTGA

TAGTGCTAAAGATCTGATTAGAAAGATGCTGTGTTCTCGGCCTTCAGAAC

GGTTGACAGCTCATGAAGTGTTATGTCATCCATGGATATGTGAAAATGGA

GTTGCCCCTGACAGAGCATTGGACCCTGCTGTTCTTTCCCGCCTTAAACA

GTTCTCTGCAATGAACAAACTAAAGAAGATGGCTTTGCGGGTAATTGCTG

AAAGTTTGTCCGAAGAGGAGATTGCTGGTTTGAGAGAGATGTTTCAAGCT

ATGGATACTGATAACAGTGGTGCAATTACTTTTGATGAGCTTAAAGCTGG

TCTAAGAAGATATGGGTCAACCCTTAAGGATACAGAAATACGTGATCTTA

TGGAGGCGGCTGATGTGGACAATAGTGGGACTATAGATTATGGAGAGTTT

ATCGCTGCAACAATTCATCTCAACAAGCTAGAGCGCGAGGAACATCTCAT

TGCAGCATTTCGATATTTTGACAAGGATGGGAGTGGCTATATTACAGTTG

ACGAACTTCAACAAGCTTGTATAGAACATAACATGACTGATGTTTTTCTT

GAAGATATTATTAGGGAAGTTGATCAAGATAATGATGGAAGGATTGATTA

TGGTGAATTTGCTGCCATGATGCAAGGCAATGCTGGAATTGGAAGGAGGA

CTATGCGCAACAGCCTGAATTTAAGCATGAGAGATGCACCAGGTGTCTGA

TTCTCATCGGTGCAGGTTGTACAGCAAAATCCAGAAAGAGAAAAATAGAG

CATGAATCTTTTATAAAGAAAAAGATTTGTTGCTATCCTGCACAGGGATC

CACTCGCATGCAAGAAGAATGGGGGATCATAGATGCTGTCCCATGAACAC

TCCTCAATTGTTTGCATTTATGTGCTGTATGAATGATTGCAAAAGTTATT

TTGATCTTTGTAAACTCCAGTTTCAACGTAACAGCTGTTTCAAGTTAGTC

GTTATTTTTCTGTACTTTCATGTAGGGGAATTCTGTTAATAGTCGAAGTA

GAAGGATTTGAAAGGTTTTGATTTTACGGATGTAGAGTTTTACACGAGTG

AAGTTGATGGTTTGGGCGAACTATGAAGCCTATTTGGATGTGTTGACTGT

TGAAGTGAAAGTAGACACATAATATATCTCAGCTGGCTTGTTTTACAATT

AAATTTATGCTTTTATTAATAATAGTTAATTTTCCATACGTAATTTTGAA

ATAGTCATCTAATCTATATTACGAACTACTGTACGGTGTTAAACTGGACT

TAAAAATTTATAATTTTTTATAAAAATTTGTATGGAGTAATGTGTATGCT

ACTATGGTCTACGCGACTAAGCAACGAGACAACGAGTAATGAGGAGAAAC

ATCAATTTACTAGAAATAATACAAATCTGTGATGCAATGGGATGAGTTTA

TTTAAATTTAAATTTGACTCATTTAATTTATGAACTTAATTTCAGGTTCA

AGTTTGGCTTATCAGTTCACGAGTTTAGCTTATCGAACTATTAACAAATC

AAATTCGAGCTAGTTTATGAGCTGATTTGACTCACTTCCAGTCCTAGCGG

TAGCACTGGAGGAGCTTGGAGGCGAGTATGTGAGATGCTACTGATGAAAG

GGAAGGGGATTAGGGTTCAGGAGTTCACCGTT

**7. AhNKEF5**

TTTAAAAATTATAAAATAAAAAATCACAATTTACAAAATAATTGAAAAAA

AATTAGAAAGATCCATCTATTATTTATATTTGTCCGAAAAGCAGTATTAT

CAAACCGAGCTCAACTTGATCGGTTGGACTGAAAAAACCGGTCATTCGGA

TCTTAAACCAGGTCGAGCCACCAATTAGACCAGACATGCAGATGCAATAG

ACCTACTTTGACCCGCTTCAACCCGGCTGGTTTTTAAAACTCGGACCGGA

TCAAGGTTTGTGTTTTTTGTTAATCTTTCTCTAAACTCAAAAAACGATGT

TACGTTTTGCTATTTACCCTGAAAGTCAAACTGTTCCTCCCTCCCTCCCT

CCTGGCCTCATCCTCCCCCAATTTCTCAGTCTCTTCGACTGAGTCTCACT

TCCCTCACCCTTCCAGTCTCTCGTCGTCCTTTGGTTCATCACTGTCGTCG

TCACAGGTGTGGGCATCCGTTTCTTCTTTTTTCTGTCTTCATCCACTCGT

ACTTCTTACCTTTGTCTCACCATCTGCCGCCTCTGCGTAAGTGCGTATCC

TGGTTTGTCGTGGCTGTCCTTTTCTCTGCCGTCGTCGTGCTTGGGCTGGC

CGTCTGCCTCTCCTACTCCTCTGAATCTACCGCTACCTGTGCTAGAAAGC

TTTGGGTCCTGTGTGTTGTGAACTATTTAATAATTGGTTTACAATTAAAT

TGTCTTCCAATTCATTCTTGAAGAGGCTATCAGCCTCTTGGAAGTGATGC

GGTGTATTAACGATGTGAAGGGATCACATATTGGAGGCAAAAGAATTTGA

TGTTGAATTTGTTGCATGAGATCGGACCTCTTATATTAAATTTTGAAAAT

GCAAAAATCTTCAAATTATGGTGTTCTTAGTGTTGAGTCCAGGTGCTCAG

TTTCTACCTTAGCTTACTGATGCATCTGCCGTAGGAATGCCATGTGGAGG

TTTGGTGTCTTGCTTCAGGAAGGGACAGATCCGGGTTTTCTCAAGTTTGT

CGCATCTCACGCACTCACTTGTGCCGGCCACCTTAAGTCAACTCTCTCTG

GCCTCTCGCAGTCTGCCTTCCTGTGTCTCACACAACGGTACTGCTGTCTT

CAGTTAACGCTGACGCTCTCTTCGCACTCTTCCTCTCTCTCGCTCGTTCC

ATCTGCCAACACTCAAACCTCCGTGGCTCTTTTCTGCCTCCACACTCATC

CTGCTCGAGGAAGAAACAAAGACACCGTCGCAGGCGCAGCATCGACACCG

CTGCAGGTTGAGTTCAGTTGACCCTCCTCTTTTGCTCCTCCATTCCAGAC

CCTTCTTTGTTCGATTTCAAGAAGGACGGGCCTTGTTTTGTGCAACACAT

ACATCAAAACAACTACCATGGGACATGAATCCCGAAAGCTCTTGGATGAA

TATGAGGTGTCAGAGATTCTAGGAAGAGGTGGATTCTCTGTTGTTAGGAA

AGGCATAAAAAAATCAAGCAGTGATGAGAAAACTCATGTTGCCATAAAGA

CACTAAGAAGAGTAAGTGCCTCTACTACAACCCCTGGTTGTTTACCAAGA

GAGAGGAGCAACATGGGGTTTCCCACATGGAGACAGGTTTCAGTATCAGA

TGCTCTTCTCACCAATGAGATCCTTGTGATGAGGAAGATAGTCGAAAATG

TGTCGCCACATCCGAATGTGGTTGACCTCTATGATGTTTATGAGGACTCG

AATGGTGTTCATCTTGTTTTGGAGCTGTGTTCTGGCGGTGAGCTGTTTGA

TCGCATTGTGGCACAGGATAGGTACTCAGAGACTGAGGCTGCGACAGTTA

TTCGCCAGATTGCGGCGGGCTTAGAGGCTATTCATAAAGCAAACATTGTC

CATAGAGACTTGAAGCCTGAGAATTGCTTGTTCTTGGACAAGAGGAAGGA

TTCTCCTCTAAAGATCATGGATTTCGGTTTGAGCTCTGTTGAAGAGTTTA

CTGATCCAGTTGTTGGTTTGTTTGGTTCCATTGATTATGTTTCACCGGAG

GCACTTTCTCAAGGAAAGATAACAGCTAAGAGTGACATGTGGTCTCTAGG

GGTAATTTTGTACATCTTATTATCTGGATATCCGCCTTTCATTGCTCAGT

CTAATCGCCAAAAACAACAAATGATAATGAATGGGAACTTCAGCTTCTAT

GAGAAGACATGGAAGGGCATTTCTCAATCAGCAAAGCAATTGATTTCGAG

TCTTCTGACAGTTGATCCTAGTAGGAGACCTAGTGCGCAGGAGCTCCTGA

GTCATCCATGGGTCATAGGTGATGTAGCGAAAGATGATCAGATGGACCCT

GAGATTGTCTCAAGGTTGCAAAGCTTCAATGCTCGTCGCAAGCTCCGGGC

AGCTGCAATTGCAAGCGTATGGAGCACCACAGTGTTCTTGAGAACTAAGA

AACTGAAATCCTTGATAGGATCCTATGATCTTACAGAAGAGGAAATTGAA

AATCTTAGGATACACTTCAAGAAGATATGTGGAAATGGGGACAATGCCAC

GCTCTCTAAGTTTGAGGAGGTACTGAAAGCAATAAATATGCCATCACTAA

TTCCTCTAGCACCACGCATATTTGACTTGTTCGACAACAACCGTGATGGA

ACGGTTGACATGCGAGAGATTTTATGTGGGCTTTCCAGCCTCAAGAATTC

CAAAGGAGATGATGCCCTCCGTTTGTGCTTCCAGATGTATGATGCAGATC

GATCCGGGTGTATCACAAAGGAAGAAGTAGCATCCATGCTTAGAGCTTTG

CCGGATGACTGTCTTCCAGTTGATATCACGGAACCTGGCAAATTGGACGA

GATTTTCGACAGAATGGATGCCAACAGTGATGGAAAAGTCACCTTTGAGG

AATTCAAAGCTGCTATGCAGAGAGATAGCTCCCTCCAAGATGTAGTCCTT

TCTTCCCTTCGCCCACTATAGTTTTTCCATTTTTCATCCAATCAATCACA

ATGTTTGGGGCTTTCCTTTTTCTTATGTTCATACTGATGGTCTCTTTTGA

GTCCACTATGTCTCGCTTTTATTCTCCAACCAAACATATCCTTGGAGAAA

AGAGAGGGAATGCAGTCTATGACAATTAGGAACCTTTGCATTCTCAAGTT

ATGTTATTGAGTACCTGAAAGGCAGAAAGCTAACATGTTCATCATAAATA

CTGTAATTAAACTAGAAAAGCCAACACTTTTCAGTCATTACATCCAATGT

GTGATATATGTAACAGATACTGTAATGGAGATGCAAAGTTTGGTTTGAGA

GAAGTGAGAACATTTAAGTGACTATATAGATACTACCGTAATCCTATCCC

ACAAAAAGACCACCTAAT

**8.AhNKEF6**

AGTTAGTCGGTTTAATCAATAATTCAGTGGTTGAATTAATAATTCGGTTT

GATTGTCGGTTCGATTATGGTAACTACGCCAAAACCAAGTTAACAATTAA

AAAACTTGCCTTGTTCAGGAAGCTCAAGTTTGTCGCATCTCACGCACTCA

CTTCTGCCGGCCACCTTAAGTCAACTCTCTCTGGCCTCTCGCCGTCTGCC

TTCCTGTGTCTCACACAACGGTACTGCTGTCTTCAGTTAACGCTCACGCT

CTCTTCGCACTCTTCCTCTCACTCAATGACGTGTGCGTCTCCGTGATATC

TGTCTCTCTCGCTCGTTCCATCTGCCAACACTCATCCTGCTCGAGGAAGA

AACAAAGACGCCGTCGCAGGCGCAGCATCGACACCGCTGCAGGTTGAATT

CAGTTGATCCTCCTCTTTTGCTCCTCCATTCCAGACTTCGATTTCAAGAA

GGACGGACCTTGTTTTGTGCAACACATACATCACGAACAACTACCATGGG

ACATGAATCCCGAAAGCTCTTGGATGAATATGAGGTGTCAGAGATTCTAG

GAAGAGGTGGATTCTCTGTTGTTAGGAAAGGCATAAAAAAATCAAGCAGT

GATGAGAAAACTCATGTTGCCATAAAGACACTAAGAAGAGTAAGTGCCTC

TACTACGACCCCTGGTTGTTTACCAAGAGAGAGGAGCAACATGGGGTTTC

CCACATGGAGACAGGTTTCAGTATCAGATGCTCTTCTCACCAATGAGATC

CTTGTGATGAGGAAGATAGTCGAAAATGTGTCGCCACATCCGAATGTGGT

TGACCTCTATGATGTTTATGAGGACTCGAATGGTGTTCATCTTGTTTTGG

AGCTGTGTTCTGGCGGTGAGCTGTTTGATCGCATTGTGGCACAGGATAGG

TACTCAGAGACTGAGGCTGCGACAGTTATTCGCCAGATTGCGGCGGGCTT

AGAGGCTATTCATAAAGCAAACATTGTCCATAGAGACTTGAAGCCTGAGA

ATTGCTTGTTCTTGGACAAGAGGAAGGATTCTCCTCTAAAGATCATGGAT

TTCGGTTTGAGCTCTGTTGAAGAGTTTACTGATCCAGTTGTTGGTTTGTT

TGGTTCCATTGATTATGTTTCACCGGAGGCACTTTCTCAAGGAAAGATAA

CAGCTAAGAGTGACATGTGGTCTCTAGGGGTAATTTTGTACATCTTATTA

TCTGGATATCCGCCTTTCATTGCTCAGTCTAATCGCCAAAAACAACAAAT

GATAATGAATGGGAACTTTAGCTTCTATGAGAAGACATGGAAGGGCATTT

CTCAATCAGCAAAGCAATTGATTTCGAGTCTTCTGACAGTTGATCCTATT

AGGAGACCTAGTGCGCAGGAGCTCCTGAGTCATCCATGGGTCATAGGTGA

TGTAGCGAAAGATGATCAGATGGACCCTGAGATTGTCTCAAGGTTGCAAA

GCTTCAATGCTCGTCGCAAGCTCCGGGCAGCTGCAATTGCAAGCGTATGG

AGCACCACAGTGTTCTTGAGAACCAAGAAACTGAAATCCTTGATAGGATC

CTATGATCTTACAGAAGAGGAAATTGAAAATCTTAGGATACACTTCAAGA

AGATATGTGGAAATGGGGACAATGCCACGCTCTCTAAGTTTGAGGAGGTA

CTGAAAGCAATAAATATGCCATCACTAATTCCTCTAGCACCACGCATATT

TGACTTGTTCGACAACAACCGTGATGGAACGGTTGACATGCGAGAGATTT

TATGTGGGCTTTCCAGCCTCAAGAATTCCAAAGGAGATGATGCCCTCCGT

TTGTGCTTCCAGATGTATGATGCAGATCGATCCGGGTGTATCACAAAGGA

AGAAGTAGCATCCATGCTTAGAGCTTTGCCGGATGACTGTCTTCCCGTTG

ATATCACGGAACCTGGCAAATTGGACGAGATTTTCGACAGAATGGATGCC

AACAGTGATGGAAAAGTCACCTTTGAGGAATTCAAAGCTGCTATGCAGAG

AGATAGCTCCCTCCAAGATGTAGTCCTTTCTTCCCTTCGCCCACTATAGT

TTTTCCATTTTCCATCCAACCAATCACAATGTTTGGGGCTTTCCTTTTAC

CTATGTTCATACTGATGGTCTCTTTTGAGTCCACTATGTGTATTTTTACT

TGGTAAGATTTACTCTCGCTTTTATTCTCCAACCAAACATATCCTTGGAG

AAAAGAGAGGGAATGCAGTGTTGAATTGTTTGAAATGTCTAGATTTTTCT

GTGACATTTAGGAACCTTTGCATTCTCTAATTATGTTATTGAGTACCAGA

AAGGCAGAAAGCTAACATGTTCATCATAAATACTGTAATTAAACTAGAAA

AGCCAACACTTTTCAGTCATTACATCCAATGTGTGATATAGGTAACAGAT

ACTGTAATGGAGATGCAAAGTTTGGTTTGAGAGAAGTGAGAAGTTGCAGG

GTAGACTCTTTTTTTTTCCCTATGCTATTTCCCAGTTTGAGAGATCAAAG

ATTAATACGTTGCGAATCTAAGTTTCATTTAAGGTCTATCGTTGACCAAT

AAATTATTATATATGCAAGATGTGATTCGAACTTTCGACCCTTGTTTAAG

CAGATGACTGAATTGACCA

**9.AhNNLC1**

TTCTTTTTTTTTTTTTAATTTTTTTTATGGTTTTTGAAAAGAAGTGGTCG

CTCCCAGTGTAGAGTGTGGGAGCGCGCAAGAGCAGCAACAATGTCTGGAT

TCTCCTTCGGTTCTTCTTCTTCCTCTCAGTCTTCGTCTTCTTCTCCCTTC

TCCTTGACAAATCCTCCATCCTCTTCCGCTTCCTCTTCCGCATTCTCCTT

CGGCTCATCCACTCCCTCCACTGGTTTCTCATTCGGATCCTCCTCCCTCT

TCTCTTCCACCACCGCCACCGCAAACCCTAGCTCCGCCGCTTCTTCCTCA

CCAAGTCCATTCTCTTTCTCATTCGCTTCTTCATCCTCCACCGCAGGAGG

AAGCGGCGGTGCCACTACTACCGCACCCTCCTTCGGCTTTGGCTCCACAC

CTTCCTCCTCCGCAGCCTCTGCGCCGTCATTTTCCTTCGGTTTTGGGTCC

GCGCCGACCGCATCTGGCTCGGCTCCTGCTCCAGCTCCGTCTCTGTTCGG

CTCTGCTTCTTCGGCTTCCACTGCGGCAGCTTCGAGTTCCGGTTCTTCCA

TTTTCGGTGCCGCGAGTTCAGGCTCCTCCTTGTTCTCCACTCCGTCTTTT

GGTGGAACTTCCTCTGCCACGACGCCGTTTGGGGCAAAACCCTCTGCTGC

GACGACACCATTTGCCGGAGCATCTTCTGCTTCTTCGGCGTCACCGTTTG

GCGGAGCATCTTCTGCTCCACCCTCGATATTTGGTGGAGCATCTTCTGCT

TCAACCACACTCTTTGGGGGAACTTCATCTGCAACGACGTCATTTGGATC

AACACCTTCTTCAACAACAGCGGCTGCGAGTAAGCCATTTGGAGGGTTTT

CGCTCTCCCCGTCGGCGGCTTCCTCTTCTGCTGCAACTACAACTCCTTCA

TTCTCAAGCGTGTTCGCCACTGGTGCTTCTTCTTCCTCTTCGAGCTCATC

TTCTCTGTTTACGGGGTTTGCAAAACCATCTGCACCAACACCAACAACAA

CTGCAGCCTCTACTGCCGCTGCGTCTGCACCGACGCCGACTAGCACTACT

GGATTCTCCTTCGGAAATGCAACTTCGTCTGCTTCTCAACCATCTTTCGG

GTTCCCCAATGCAGCTGTTTCATCACCAGCTTCATCCGCTTCTACAGCTT

CCAGCACACCGGCTTCAAAACCTCCTGGTTCTTTCTCGTTCACCACAGCA

TCAGCGCCATTGTTTTCGACTGTAACTGCCACCACAGCTTCCACACCAGC

CGCGGCTGCCAGCGGCAGTACCCCGTCGTCTTCTGTGCCTGCATTTGGTA

TCCCAGCTTCAACTGCTCCGGCAATTGCTGCATCTTCGTTATCGGGGACT

CCAGCAGCTTCTGCTGGTGCTGCCTCCTCTACTAGCGGAGGATCATCTTT

TGCTGGATTTGGTGTGGGGAGCTCTGCATCAACCGGTTCCTCCACTGCTT

CTTTTGGGACTGGGTTTTCTTTTGCAACCAAGGCATCTGCAGCATCAACA

CCGGCTGTTTCAAGTTCTGCACTTGCATTTGGAGTTAGCAGTACCACAAC

AACTGCTCCGACAATTTCCAGCTCCAGTGCAAGTGCAACTCAGACATCAT

CTGCTCTTGTTGTGGCTTCCACTAGTGGAACTACTTCAACTGTCAGCACT

TCAGTTGCTGCTGCTGCGGCTCCCAAATTGCCATCTGAAATTACAGGGAA

GACTGTGGAAGAGATTATCAAAGAGTGGAATACTGAGTTGCAAGAGCGTA

CTGGAAAATTTAGGAAACAGGCTAATGCTATAGCTGAATGGGATCGCAGA

ATTTTGCAGAATCGTGATGTTCTATTGAGGCTTGAGATCGAAGTAGCAAA

AGTAGTTGAGACACAGTCAAACATGGAGCGGCAATTGGAGTTAATTGAGA

CTCATCAACAGGAGGTTGACAAGGCTTTACAAAGCATGGAAGAAGAAGCT

GAACGTATTTACAAGGATGAGCGTGGATTGCTTCTTGATGATGAAGCTGC

TTCTACAAGAGATGCAATGTATGAGCAGTCTGAACTGATAGAAAGGGAAC

TAGAGCAGATGACAGAACAGATCAAATCCATCATCCAATCTCTCAACTCA

AACCAGGGTGGAGAGCTTGATGCACTTGATGGAATGACTCCATTAGATGC

AGTAGTTCGAATTCTAAACAATCAACTAACCTCTCTGATGTGGATAGATG

AAAAGGCTGAAGAATTTTCATCCCGCATTCAGAAGCTTGCTAACCAAGGT

TCTGCTTCGGATCGTGAACTGATGGGTCCAAGAATGTGGATGTCCTGATA

TGTCGATCTTGTAAGGACCTGTTCAAAATTTAGATGAAATGCCAGCAGAT

AAACACAGGGATTTGTGAGAAGAGGTTATTGTGCCTTTTGTGTGAGATTT

GAGATGAATTGTTTGAGTGATTGTTACTATTAAATTTTCTGTTAGCCTAA

ATGTTATTTTTTACATATACTGTTTTTAAAAGAATAGAAAAAAATATAAA

TACATTTAATATCTAAATAGGTAATGAACAAAATAATTAAAAGAATGAGA

GACAAGCTTTTTCTTTTGAAAAGCTTATGTTCTTTCCTCAACATTTGTTA

TCATAGTATTCTTGACTATACATGAGGAAAACTAAAAAAAAAATCATACA

TGAGCTGGTCAAAAGAGATTTATATATAAGCAATTTAATTTGATCCATGT

AACTAACTCTACCTAATGAGATAAAGTTGTTTTGTTGTGAACTTGTGATA

TTGCATTCGGCCATCCAAATAAAGGCTTT

**10.AhNNLC2**

AAAAAATCTCTCTTTTTTTTTTTATTATTATTCTTATTTTTTTGGTTTTT

GAAAAGAAGTGGTCGCTCCCAGTGTAGAGTGTGGGAGCGCGCAAGAGCAG

CAACAATGTCTGGATTCTCCTTCGGTTCTTCTTCTTCCTCTCAGTCTTCG

TCTTCTTCTCCCTTCTCCTTGACAAACCCTCCATCCTCTTCCGCTTCCTC

TTCCGCATTCTCCTTCGGCTCATCCACCCCCTCCACTGGTTTCTCATTCG

GATCCTCCTCCCTCTTCTCTTCCACCACCGCCACCGCCACCGCAAACCCT

AGCTCCGTCGCTTCTTCCTCACCAAGTCCATTCTCTTTCTCATTCGCTTC

TTCATCCTCCACCGCAGGAGGAAGCGGCGGCGCCACTACTACCGCACCCT

CCTTCGGCTTTGGCTCCACACCTTCCTCCTCCGCAGCCTCTGCGCCGTCA

TTTTCCTTCGGTTTTGGGTCCGCGCCGACCGCATCTGGCTCGGCTCCTGC

TCCAGCTCCGTCTCTGTTCGGCTCTGCTTCTTCGGCATCCACTGCGGCAG

CTTCGAGTTCCGGTTCTTCCATTTTCGGTGCCGCGAGTTCAGGCTCCTCC

TTGTTCTCCACACCGTCTTTTGGTGGAACTTCCTCTGCCACGACGCCGTT

TGGGGCAAAACCCTCTGCTGCGACGACACCATTTGGCGGAGCATCTTCTG

CTTCTTCGGCGTCACCTTTTGGCGGAGCATCTTCTGCTACACCCTCGCTA

TTTGGTGGAGCATCTTCTGCTTCAACCACACTCTTTGGGGGAACTTCATC

TGCAACGACGTCATTTGGATCAACACCTTCTTCAACAACAGCGGCTGCGA

GTAAGCCATTTGGCGGGTTTTCGCTCTCCCCGTCGGCGGCTTCCTCTTCT

GCTGCAACTACAACTCCTTCATTCTCAAGCGTGTTCGCCACTGGTGCTTC

TTCTTCCTCTTCGAGCTCATCTTCTCTGTTTACGGGGTTTGCAAAACCAT

CTGCACCAACACCAACAACAACTGCAGCCTCTACTGCCGCTGCTTCTGCA

CCGACGCCGACCAGCACTACTGGATTCTCATTTGGAAATGCAACTTCGTC

TGCTTCTCAACCGTCTTTCGGGTTCCCCAATGCAGCTGTTTCATCACCAG

CTTCATCCGCTTCTACAGCTTCCAGCACACCGGCTTCAAAACCTCCTGGT

TCTTTCTCGTTCACCACAGCATCAGCGCCATTGTTTTCGACTGTAACTGC

CACCACAGCTTCCGCACCAGCCGCGGCTGCCAGCGGCAGTACCCCCTCGT

CTTCTGTGCCTGCATTTGGTATCCCAGCTTCAACTGCTCCGGCAATTGCT

GCATCTTCGTTATCGGGGACTCCAGCAGCTTCTGCTGGTGCTGCCTCCTC

TACTAGCGGAGGATCATCTTTTGCTGGATTTGGTGTGGGGAGCTCTGCAT

CAACCGGTTCCTCCACTGCTTCTTTTGGGACGGGGTTTTCTTTTGCAACC

AAGGCATCTGCAGCATCAACGGCGGCTGTTTCAAGCTCTGCACTTGCATT

TGGAGTTAGCAGTACCACAACAACTGCTCCGACAATTTCCAGCTCCAGTG

CAAGTGCAACTCAGACATCATCTGCTCTGGTGGCTTCCACTAGTGGAACT

ACTTCAACTGTCAGCACTTCAGTTGCTGCTGCTGCGGCTCCCAAATTGCC

ATCTGAAATTACAGGGAAGACTGTGGAAGAGATTATCAAAGAGTGGAATA

CTGAGTTGCAAGAGCGTACTGGAAAATTTAGGAAACAGGCTAATGCTATA

GCTGAATGGGATCGCAGAATTTTGCAGAATCGTGATGTTCTATTGAGGCT

TGAGATCGAAGTGGCAAAAGTAGTTGAGACACAGTCAAACATGGAGCGGC

AATTGGAGTTAATTGAAACTCATCAACAGGAGGTTGACAAGGCTTTACAA

AGCATGGAAGAAGAAGCTGAACGTATTTACAAGGATGAGCGTGGATTGCT

TCTTGATGATGAAGCTGCTTCTACAAGAGATGCAATGTATGAGCAATCTG

AACTGATAGAAAGGGAACTAGAGCAGATGACAGAACAGATCAAATCCATC

ATCCAATCTCTTAACTCAAACCAGGGTGGAGAGCTTGATGCACTTGATGG

AATGACTCCATTAGATGCAGTAGTTCGAATTCTAAACAATCAACTAACCT

CTCTGATGTGGATAGATGAAAAGGCTGAAGAATTTTCATCCCGCATTCAG

AAGCTTGCTAACCAAGGTTCTGCTTCGGATCGTGAACTGATGGGTCCAAG

AATGTGGATGTCCTGATATATCGATCTTGTAAGGACCTGTTCAAAATTTA

GATGAAATGCCAGCAGATAAACAAACACAGGGATTTGTGAGAAGAGGTTG

TTGTGCCTCTTGTGTGAGATTTGAGATGAATTGTTTGAGTGATTGTTACT

ATTAAATTTTCTGTTGGCCTAAATGTTATTTTTTACATATACTGTTTTTA

AAAGAATAGAAAAAAAATATAAATACATTTAATATCTAAATAGGTAATGA

ACAAAATAATTAAAAGAATGAGAGACTAGCTTTCGTTTGAATAGCTTATG

TTCTTTCCTCAACATTTGTTATCATAGTATTCTTGACTATACACGAGGAA

AACTCAAGAAAATCATAAATGATGTGGTCAAAAGAAATTTACATATAAGC

AATTTCATTTGTAGACATGATACTATGATAGGATTCAATAGCATCGATTG

ATCTGTGTAACTAACTCTACCTAATGAGATAAAGCTGCTTTGTTGTAATA

TTTTTCATTTGGCCATCCAAATAAAGGCTTTTAGTTCATTCAAATAAACT

AAATATCATTTAATCTTAGAATCATTAGTGATTTAATCTCCAGTTTATTT

TAAAGCTTTTAATTGAAGAAGTATCTATCTACATTGAATATATTTTTGTT

AGTTGTTTCAATTATTCAAAATCTAGCGATATGCAAATACGAGTCTGTAT

CTTGCAAAATGCATTTTCAGGTTGATTGACACAGCAACTTGCTAAATATT

TGTATTATAAAATTGAATACAATGAGAAAGCTAATACATACACTCTCAAA

TGTCTCGGGCGACAGACTAGTGTCTTATGTACAAGAAAAAGTTGTGACAC

GAATGAGGCAAAAGATTGTGCAAGATGTCAAGATTGTCCAAACAAATCGA

AATCAACAGCCACGAATGAAACTACTCTTGCATGTTCACACACCAATCAG

AAGGTGTGCGTAGTTCATGAAATGTTTTACATTCTATGATTTTTGGCTTA

GAAATAAGGTTCCCCAGCGCACAATATTCCCAAGAATGTGAGACATCTGA

TGAACCACATTTCCTAAGATCTTTCCAATCGCGCCATGATCACGATCAAG

AGGATTACAATGCAGAGAAGCAGAGATGACCTGAGTTTCTCGTGCAGCAC

TGCCGGATCTACGTGTTGCGTCTTTGGAGAATGAAAGAAATCATTTACCA

GTGACTGGAAGTTTTCTATCAGTTCCCAGACCTTTCCTTTTATGAGTTGT

AGTATCCAGCTTAACAGATACATGGCGAGCTCATTTTCTTCAGAATTCTG

AGGCGTCTTGTTCTCATCTAGATGAAAGAATACAACGTGGAATGAGTCAG

TTCGTTAGAGGTTGAAGCAATCTAGGATTTCAATATTTCATGAAACAGAC

ATTGGCATTTGGCAATATTGGTGTCGTTCATTCAACATAGCTTCTTTGTG

ACAGTTTTCACTTAATAACCACTAGTAATTATTATTGTTATTATTATTCA

AAATACTCAAATGCCATGCCAATAGTCACAAAAAAAATGCCATGCCAATG

ACTCACTAGTCAAGTGGCAAAGTCAACTAGAAAAGAAGGTGTTTATGGAC

TAAACATCCAGGGGGAAAACAAACTGTTTTGATGCGTGGCTCCCTAAGTT

ATGACTAAACACTCTTTCTAGGTTATATATCAGGAGGCTGGAAGGATTGG

TTTCCGCTATGTTGGTAAACCCACCAAAGGTAGTATAAAGAACAAGGTCA

AAAGTAAAAAAGAAATACTCAACTGCACTTGCTAGCCGCTGCATCTTAGT

GATTTGATTTTGTTTAGCTTCTTCATTTTATAAAGGGTTAAGTACATTTT

TTGTTCTTTTAAGTTTGACAAAAGTTTAAAAAATATCTCTAAATTTTATT

TTGTTTCAATTTTGTTCCATAAGTTTTCGATTTGCATCAAAATATATCTC

TGGTAGCTAATTTTTCAAAAAAATTAGAATCAATTCAACAATAATTACAT

AAAAATAACATTCAACACAAGCAAATCAAACATAATTGTCATGCATTATT

GTTGGATTGGTCTTAAATTTTTTTAGTTTAACTGTCGGAATTTGGATCCT

CTAAATTTTAAATTTCACTTTAAATGGCAAAGCATGATCTATTACTGTTT

ATTTCATCGGTGGGAGTAAGAGAAAATATGAAAGAAAAAACATTTAATGG

TAGGAGATCTTACTATATACCCTAAAGTGAAAATCTAAAATTTAGAAGAT

CCAAATTCTAACTGTAAGAGGTATATTTGATGCAAATAAAAATCTTTTGA

GACAATAAAATTTAGGGAAGTATTTTAAAATTTTTGCCAAACTTTAGGGA

CAAAAATATATTTTACCGTTATATAAAATAAGAGGGGGTCTTGGAGCACC

GATGAGTTCTCTCCGTGTGATTTTGAGGTCAAATTCAAACTGTGAAATGA

GTCCCTAATCCGTTTATCAGTTAAAATACATTATACGCTTTCCTGGAATT

TGCATAAAGCGGAATATTTGTGCACAGGGTTGAGCTTCATTTTATAAAAT

AAATATGCATATAACTAGAAATAAAGACCTGAATCAGGCATCTCTAGGAG

CTTTTGATGCCGCACATGGCTCATGACAGCATCCCAAATAGCTTTATCAG

ATGACAGCGACACCACTAATCTCTACAAGGAAATTGTAGTCCAATACAAA

GTTAACGAACAAATCTAACAGAGGTCATAAAAATTTCAACAATAAATGCA

AAAATAATAACAAATTTTGAAGAGAATAGTCGCCAAATAGCGTTAATTAT

AATGAGAAGATCTGTATATAAATGAAAAAAATTCCAAGATGTTGGGAGAT

CAAAACAATATAGTGGAAAAGTAAGAACCCATAAAAGTATGTCTTTAAGA

TAACAAAATTTATCACCCATAAAACAAGATAATTATTACAGAAACTAATT

TTATAGATTGACAATTTAAAATAATCATTTTACAGAACGCAAAGGAATCA

AGCCATATTCATTATTTATTTATTTTCAATATCTATTACTTATTGAAGTA

TATTGATCGTTCTTTTTTTTTTTTTGTCGTACACGATGATTGAGTAAAAC

TGAATTATACCGGGTAGAAAATTACCCAATTGCCTAAAATTATTCTCATT

TTAATAATCATATCCATGTTGGATTATGGTTATATACGTAGTTTACTATG

TAAATTTGTACTGTTTCACTTTGAGAAGCATAAAAAAAACTTTCAAATGT

TGTAAAACTTCAAAATGGAACCTTAATTGATGGATCAGTTTGCAGCAACT

GGATTGCATCATATAATCGTTTATATCCCTCAGACAGAAATATCCTCGAA

TCAGAAGAGCTTGTGATTTGTCGATCTGAGCTCGAGGATGATACCACTTC

TATAAAACTGGCAAATCAATTTGAAAAGGAATACTTGAGTTTCTAATTGT

TATGGTAGTTTGCGCAAGTCAGTAATACACTAATTCTAGAAAAATTGAAA

CCACATGAAGTGCAGTCCAACTCTTTTGACCGGGAAATACACAAAATAGT

CCAGGTAGGAAATAGACTAAATAAATTGGAATTTTTGTTTATTTCCAAAC

AAATTCATCCCTCCTTAGCTAGATCTTATCATAAGACATACTAATCCTTC

AAGACTACAAAAGAACACGGAATTAATTTACAAGAATTTTTAGAGAATAA

AAATTTGTTAAGTACCAAAGTGTAGCATATGATATTCTGACCGATTTGGT

ATCTTATTTGTTGTTTAAGATTAAAACGGGAATCAATTTAATGACTAAAA

TCTTTAATTTTAAAATCCCCAGAACACTGGAAAATTACCCATCAACATGT

CCGAACAAACTCAAAACCTAAGTCATGGTAATTTCCCCACAAATATTTCC

AAAAACAAGCAAATTCCAGAACTAACAGTTCAAATTATGTAAAAGCAGCT

CAAGTAACAAAAGAAACTCCCAGAATGCTAACATTAGGTAAGAATAAGAA

ATTGAGAACTATATAGAACTCACTTTAGAAGAGCTGATATGGCATCCTCA

ACTTCCCGTTGAGATGGAACCGTTTCAAAGACGTGATCGTAGAGACTACT

GTTGGCTTTTTTGGCTACTTCTTTCGGATCCACCGGCATAAGCTCTCTAC

TAGGTTTATTGCTGCGAGAGATAGAAGTAGATAAGGTAGTGTTTGCGTTT

GGAGGCTTGAGATGATTAGGAGAAGAAAATGAAGATGAAGATGGCGGCGG

AGGAGAAAGCTTTTCCAGATTTGAGCTTAACTGAGGCACAGAAGGACGCT

GAGGCATGGTTCCACCACCCATGACCGACCGAATTTCCAGATTCAAGGTT

GTTGAGGTGAGCAATGATTGTCGCTGCTTACTCTCTTTTCTTATCTATGG

ATGCTACGGGTTGGTGTCATTTGACAGGCTTTTGGTACACTGGTTAATTA

TGGTGCACTATATTAATTTAGTTTTAGTAAGATGAATGCTATGCTGAATA

AGAAAAATTGTTTTTATTAATT

**11.AhNPR1**

CACCTCCATTTATTAATTATTATTATTATTGACATCAGCATGAACTTGAC

AATTATAAAAGAGTGCACAGCACCAAATATCAAATAAAATAATAAAAACC

AGAACTTGGAGTATCTTCAAGAATCTTTACATGAAAGAAACTGTTTGAAG

ACTGAAGAAGCCGAATCCGAATCCTCTTACCTTTTTTTTTTTTTTGTTTA

AGACAAGAAAGATTGTTTTGTTAGATCTGTTTTGGGAGCACCAACATGAG

AGATGGTGGAGTGTTATCTTCAGGTACCATGCTGGAACCTACTCCTCCTC

CTCCTCCTCCTTCACCACTACCACCACCGCCACCACCTCCTTCAGAGGTA

GCAACAACAACAACAACATCAATATTAATGGACTTTGATTACATCAATGA

ACTCTTTGTAGATGGTTGCTGGTTAGCAGCTTCTTCTTCTTCTGCTGCTG

CTGCTGCCGATGGATCTGCTGATTTCTATGTGTCAAGTCCTTCTTTTTCA

AACCCCATATTTGATCCTTTCTCTTGGCCTTCCTTGGATACTGAACAAAT

TGAATCCCAAGAACGAGAACAGGAACATGAACAACAACAAGAACAACCAC

CTTTTAGCCATGACTTAGTTGTTGCTGCTAATAATTGTAGCCAGAATCAG

CAACAACAGTATCATCACTATGAGAATCAGTCAGTGGAAAACAATAATCC

AAATCCTAATAATCCTTCTGAGTTTTTCAGAAGGTGGTGGATTGCACCAA

GTTCTAATCCGGGACCTGGATCTTATGTAGTTGAGAAGCTATTGAAGGCA

CTCATGTGTATCAAAGATGTGAACAGAAACAAAGACATGCTTATACAAAT

ATGGATTCCAGTTATAAACAGAGGAGGCACTCAGATTCTCAGAACCAATG

GGCTTCCATTCTCACTTGAATCAAGCTCTGTGAACCTTGCAAAGTACAGG

GAAATTTCGGAGGTGTACCAGTTCTCCGCCGAGGAGGATTCGAAGGAGTT

GGTGCCCGGATTGCCAGGGAGAGTTTACAAGGAGAAGGTTCCTGAGTGGA

CTCCTGATGTTAGATTCTTCAGGAGCTATGAATATCCAAGAGTTGACCAT

GCTCAAGTCTATGATGTGAGAGGATCATTGGCACTTCCAATCTTTGAGCA

AGGTAGCAAGAATTGCTTAGGTGTTGTTGAAGTTGTCATGACTCAACAGA

AAATCAACTATCGCCCTGAACTTGAAAGTGTTTGCCAAGCACTTGAGGCT

GTTAATCTTACAAGCTCAAAACTTCCAACTATTCAGAATGTGAAGCGTAC

ATCATGTGAAAAGTCCTATGAAACTGCATTGCCTGAGATTCAAGAAGTAT

TAAGATCTGCATGTGAAATTCACAAATTGCCATTGGCACAGACATGGATT

CCATGCATCAAACAAGGCAAAGAAGGATGCCGGCACTCGGAAGACAACTA

TCCACACTGCATATCTCCAGTGGAGCATGCTTGCTATGTTGGTGACCCTT

CAATCCAGGTCTTTCATGAGGCATGCTCTGAGCATCACTTGTTAAAAGGC

CAAGGTGTTGCCGGCGGAGCATTCATGACGAATCAGCCTTGCTTCGCACC

CGACATAACTTTGTTAAGCAAGACAGACTATCCACTTTCTCATCATGCAA

GGATGTTTGGATTGCGCGCTGCAGTCGCCATACGCTTGCGAAGCATCTAT

AATAGTTCAGATGACTTTGTTCTTGAGTTCTTCTTGCCTTTGGAATGCAT

TGACAATGATGAACAGAAGAAGATGCTCACTTCATTGTCCTTGATCATAC

AAAGGGTTTGCCATAGCTTGAGGGTTATATCAGATAAGGAAGTGGAGGAG

GAAACTGATTTTTCAGCTGAAGTGATAGCTCATGAAGATAGTGGCACTTT

TGCTAGTGCTGCAGCGTGGCCAGAACCACTGCAGAGTCAAATTGTTGCTT

CATTGGGTGCTCAAGAAAAATCAAGTGAAACTATGGGGACGAGTTTCTCC

GACCAAAGGCAACAACAGCAAGAGAGTTCTGTTTTGAAAGGGAACCTTGA

CTCTAATGGGGAGTGTTCTACTTATAATGTCGGAAACTTGTCAAGTAAAA

CCGGAGACAAGAAGAAGAGCAAAGTAGATAAGACTATCACTCTGCAAGTT

CTTCGCCAACACTTCGCCGGAAGCCTAAAAGATGCAGCAAAGAACATTGG

TGTTTGTACTACAACCTTAAAAAGGATCTGCCGGCAACATGGGATAAAAC

GTTGGCCTTCAAGGAAGATCAAGAAGGTTGGTCATTCCTTGCAGAAGCTT

CAACTTGTGATTGACTCAGTCCAAGGTGCTTCTGGTGCATTCCAAATAGA

TTCCTTCTATTCAAAATTCCCAGACTTAGCTGCTTCTCCAAATCTATCAG

GAACAAGCCTGTTCTCAAATCTCAAGCAATGTGATAATAATCCAAACTCA

CTAAGCATACAGCCGGATCCTGGTTCGCTGAGTCCGGAAGGCGCATCAAA

ATCGCCGTCCTCTTCTTGCAGCCAGAGCTCGATTTCTAGCCATCCATGTT

CCAGCATGGCTGAGCAACAGAATCATCATCACACAAATAATAATTTTGAT

AGTAGCAAAGATCAAATGGTGTTATTGGTTGGAGAGAATTCTAGCGGCGA

TGGCTTGTTGAAGAGGATAAGAAGTGAAGCAGAATTGAAAAGCTTGAATG

AAGATAGAGCAAAAGTAGTCATGCCAAGATCGCAAAGTCAAGAAACACTT

GGTCAACATAACCTTAAAAATGGGCACCATGGATCATTGTCAAGAACCAA

AAGCAAAGGGACTCAAAAGGAGGATGCACCTTATAGAGTGAAAGTAACAT

ATGGGGATGAGAAAGCAAGGTTCAAGATGCCAAAGAATTGGGGATATGAA

GATCTTGTGCAAGAAGTTGGTAGGAGATTCTGCATAAGTGACATGAACAA

ATTTGATCTCAAATATTTGGATGATGATTATGAATGGGTGCTACTAACCT

GTGATGATGATTTAGAAGAATGCATTGAAGTTTGTCAATCATCTGAGAGC

ACCACCATCAAGCTTTGTCTTCAATTCTCTAATACTAATCATAGTATGCG

AAACCCTTTAGAATTTAGATAGCAATAATAGAAGTGAACCTTTTTTTTCA

CTTGTAAATTATAAGATTGTTTGTCATGTTGTGTGAATCAATCTTAGAAA

GTTTGTAGTTTAAAGGTTCAACAATTCTTCTTTGAAGACTTTGTTTTGGC

AGATTATAAGGACAAAGATGTACATATATCCATATTGCTTGAAGCAATAA

TCAAGTTCAACTTTCCTTACCTCTTAGTCTATCTAATTAGAGTTTGACTA

ACGAATTTTCTTAGTATCTTTTAATTTTTTTTTTTAATACTTTACTTAAA

GAACGAAAGTTCTGGATGTGATTTATTATCATTTAAATGAAAGCATGTTA

AGGGTTATCGTGCTTAAGTTTTTGATTGTTTCTCGATGATAATTAATAAA

TAATTTAGAGGCAAAGGACACCTAGGGATTTTGATATACAGAAATGAGAT

AATTAATTAAAATTAATCATATTCGTTAGTTGTTGTTTCCAGCGCGACAC

AATCATGGCACCGCTCTTGTTTGCATCGCAT

**12.AhNPR2**

TTATTATTATTATTATTATTATTGACATCAGCATGAACTTGACAATTATA

AAAGAGTGCACAGCACCAAATATAAAATAAAATAATAAAAACCAGAACTT

GGAGTATCTTCAAGAATCTTTACATGAAAGAAACTGTTTGAAGACTGAAG

AAGCCGAAGCCGAAGCCGAATCCGAATCCTCTTACCTTGTGTTTTTGTTT

AAGACAAGAAAGATTGTTTTGTTAGATCTGTTTTGGGAGCACCAACATGA

GAGATGGTGGAGTGTTATCATCAGGTACCATGCTGGAACCTACTCCTCCT

CCTCCTCCTCCTCCTCCACCACCACCACCACCAGCACCACCTCCTTCAGA

GGTAGCAACAACAACAACATCAATATCAATGGACTATGATTACATCAATG

AACTCTTTGTAGATGGTTGCTGGTTAGCAGCTTCTTCTTCTTCTGCTGCT

GCTGCTGCTGCTGATGGATCTGCTGATTTCTATGTGCCAAGTCCTTCTTT

TTCAAACCCCATATTTGATCCATTCTCTTGGCCTTGCTTGGATACTGAAC

AAATTGAATCCCAAGAACGAGAACGAGAACAAGAACATGAACATGAAAAA

CAACAACAACCACCACCTTTTAGCCATGACTTAGTTGTTGCTGCTAATAG

TTGTAGCCGGAATCAGCAACAACAGTATCATCACTATGAGAATCAGTCAG

TGGAAAACAATAATCCAAATCCTAATAATCCTTCTGAGTTTTTCAGAAGG

TGGTGGATTGCACCAAGTTCTAATCCGGGACCTGGATCTTATGTCGTTGA

GAAGCTATTGAAGGCACTCATGTGTATCAAAGATGTGAACAGAAACAAAG

ACATGCTTATACAAATATGGATTCCAGTTATGAACAGAGGAGGCACTCAG

ATTCTCAGAACCAATGGGCTTCCATTCTCACTTGAATCAAGGTCTGTGAA

CCTTGCAAAGTACAGGGAAATCTCGGAGGTGTACCAGTTCTCTGCCGAGG

AGGATTCGAAGGAGTTGGTGCCCGGATTGCCAGGGAGAGTTTACAAGGAG

AAGGTTCCTGAGTGGACTCCTGATGTTAGATTCTTCAGGAGCTATGAATA

TCCAAGAGTTGACCATGCTCAAGTCTATGATGTGAGAGGATCATTGGCAC

TTCCAATCTTTGAGCAAGGTAGCAAGAATTGCTTAGGTGTTGTTGAAGTT

GTCATGACTCAACAGAAAATCAACTATCGCCCTGAACTTGAAAGTGTTTG

CCAAGCACTTGAGGCTGTTAATCTTACAAGCTCAAAACTTCCAACTATTC

AGAATGTGAAGCATACATCATGTGAAAAGTCCTATGAAACTGCATTGCCT

GAGATTCAAGAAGTATTAAGATCTGCATGTGAAATTCACAAATTGCCATT

GGCACAGACATGGATTCCATGCATCAAACAAGGCAAAGAAGGATGCCGGC

ACTCAGAAGACAACTATCCACACTGCATATCTCCAGTGGAGCATGCTTGC

TATGTTGGTGACCCTTCAATCCAGGTCTTTCATGAGGCATGCTCTGAGCA

TCACTTGTTAAAAGGCCAAGGTGTTGCCGGCGGAGCATTCATGACGAATC

AGCCTTGCTTCGCACCCGACATAACTTTGTTAAGCAAGACAGACTATCCA

CTTTCTCATCATGCAAGGATGTTTGGATTGCGCGCTGCAGTCGCCATACG

CTTGCGAAGCATCTATAATAGTTCAGATGACTTTGTTCTTGAGTTCTTCT

TGCCTTTGGAATGCATTGACAATGATGAACAGAAGAAGATGCTCACTTCA

TTGTCATTGATCATACAAAGGGTTTGCCATAGCTTGAGGGTTATATCAGA

TAAGGAATTGGAGGAGGAAACTGATTTTTCAGTTGAAGAAGTGATAGCTC

ATGAAGATAGTGGCACTTTTGCTAGTGCTGCAGCGTGGCCAGAACCACTG

CAGAGTCAAATTGTTGCTTCATTGGGTGCTCAAGAAAAATCAAGTGAAAC

TATGGGGACGAGTTTCTCCGACCAAAGGCAACAACAGCAAGAGAGTTCTG

TTTTGAAAGGGAACCTTGACTCTAATGGGGAGTGTTCTACTTATAATGTC

GGGAACTTGTCAAGTAAAACCGGAGACAAGAAGAAGAGCAAAGTAGATAA

GACTATCACTCTGCAAGTTCTTCGCCAACACTTCGCCGGAAGCCTAAAAG

ATGCAGCAAAGAACATTGGTGTTTGTACTACAACGTTAAAAAGGATCTGC

CGGCAACATGGGATAAAACGTTGGCCTTCAAGGAAGATCAAGAAGGTTGG

TCATTCCTTGCAGAAGCTTCAACTTGTAATCGACTCGGTCCAAGGTGCTT

CTGGTGCATTCCAAATAGATTCCTTCTATTCAAAATTCCCAGACTTAGCT

GCTTCTCCAAATCTATCAGGAACAAGCCTGTTCTCAAATCTCAAGCAATG

TGATAATAATCCAAACTCACTAAGCATACAGCCGGATCCTGGTTCGCTGA

GTCCGGAAGGCGCATCAAAATCGCCGTCCTCTTCTTGCAGCCAGAGCTCG

ATTTCTAGCCATCCATGTTCCAGCATGGCTGAGCAACAGAATCATCATCT

CACAAATAATAATTTTGATAGTAGCAAAGATCAAATGGTGTTATTGGTTG

GAGAGAATTCTAGCGGCGATGGCTTGTTGAAGAGGATAAGAAGTGAAGCA

GAATTGAAAAGCTTGAATGAAGATAGAGTAAAAATAGTCATGCCAAGATC

GCAAAGTCAAGAAACACTTGGTCAACATAACGTTCAAAATGGGCACCATG

GATCATTGTCAAGAACCAAAAGCAAAGGGACTCAAAAGGAGGATGCAGCT

TATAGAGTGAAAGTAACATATGGGGATGAGAAAGCAAGGTTCAAGATGCC

TAAGAATTGGGGCTATGAAGATCTTGTGCAAGAAGTTGGTAGGAGATTCT

GCATAAGTGACATGAGCAAATTTGATGTCAAATATTTGGATGATGATTAT

GAATGGGTGCTACTAACCTGTGATGATGATTTAGAAGAATGCATTGAAGT

TTGTCAATCATCTGAGAGCACCACCATCAAGCTTTGTCTTCAACTCTCTA

ATACTAATCATAGTATGCGAAACCCTTTAGAATTTAGATAGCAATATTAG

AAGTGAACCTTTTTTTTTCACCTGTAAATTATAAGATTGTTTGTCATATT

GTGTGAATCAATCTTAGAAAGTTTGTAGTTTAAAGGTTCAACAATTCTTC

TTTGAAGACATTGTTTTGGCAGATTATAAGGACAAAGATGTACATATATC

CTTATTGCTTGAAGCAATAATCAAGTTCAACTTTCCTTAACTCTCAGTCT

ATCTAATTAGAGTTTGACCAACGAATTTTCTTAGTATCTTTTTTTTTTTT

TAATACTTTACTTGAACGAAAGTTCTGGATGTGATTTATTATCATTTAAA

TGAGAGCATGTTAAGGGTTATCGTGCTTAAGTTTTGGATTGTTTGTCGAT

GATAATTAATAAATAATTTAGAGGCAAAGGACACCTGGGGATTTTGATAT

ATAGAAATGACATCATTAATTAAAACTAATCATATTTGTTAGTTGTTGTT

TCCAGCGCGACACAATCATGGCACCGCTCTTGTTTGCATCGCAT

**13.AhNPR3**

CATATAGCCATATTTCTCTGTATTTTGCTGTTCCCACCCCTTCTCTCTCT

CTCTCTCTCTTCACCATAATCACAAAGTTCTAATCTTTATCACTCTGCAA

CTCCAAATTCTCCTTCAAAACAATCCCTTTTCTCTTCTTTGAGAATGCCA

GACTCCTGTGAAGAAAAATCTGAACTTCCTTCCAAGTCAAAGCCACAAGA

AGAACATGGTTTTCCCATGGATTTTGATATATACCTTGAAAGTTCATCAT

GGCCCATGGATCACACCCCTTCTGCTTCCAACCCCATGTCTCCTTTCATC

ATAACAACCTCCTCTGAACAACCTTTTTCTCCTCTCTGGGCTTTCTCTGA

TGTTGAAGATGATCACAGGCATGTCAGGGTTGTTGCAGGTGATAATACAA

ATACAGCAATTGAAACTGAAAATCCGGTTGAAAATGATGACAACAAAAAA

ATAGTGTCACCCCCCTTTGTGCCTCTTCCACCTATAAAAATTCCAGATGG

TTATTGTCTAATTAAGGAAAGAATGACACAAGCACTTCGCCACTTCAAAC

AGTTGACTGAACAGAATTTTCTGGCTCAGGTTTGGGCACCTGTGAGGAAT

GGCAACCGTTATGCACTCACAACTTCAGGTCAACCATTTGTTCTTGATCC

ACATAGTAATGGACTACATCAGTACAGAACAGTTTCCCTGATGTATATGT

TTCCTGTTGATGGGGAGAATGATGAAATCTTGGGACTTCCCGGTCGAGTT

TTTCAGCAGAAATTGCCAGAATGGACTCCCAATGTTCAGTATTATACAAG

TAGAGAATACTCTCGGAGAAACCATGCACAACATTACAATGTCCGCGGAA

CGTTGGCTTTGCCCGTGTTTGAATCACCAGGGCAGTCATGTGTTGGTGTA

TTGGAGCTGATAATGACTTCAGAGAAGGTTAATTATGCTCCTGAGGTTGA

TAAAGTCTGCAAAGCCCTTGAGGCAGTGAATTTGAGGAGTTCAGAAATTC

TGGAACATCCATTTGCTCAGATTTGCAACGAAGGTCGCCAGAATGCGTTA

GCAGAGATTTTGGAGATATTGACAGTGGTATGTGAAACTCATAATTTACC

TCTTGCACAAACATGGGTTCCCTGTAGGCATCGGAGTGTTTTGGCAAACG

GCGGTGGTCTTAAGAAAAGCTGTTCTAGTTTTGATGGCCACTGCATGGGG

CGAGTTTGCATGTCTGCCTCCGATGTAGCATTCTATGTCATTGATGCTCA

TACATGGGGTTTCCATGATGCTTGCGCTGAGCATCACTTACAACAAGGTC

AAGGTGTTGCTGGCAGGGCATTTTTATCCCATAACATGAGCTTCTGTGGA

AACATAACTCAATTCTGCAAAACTGATTATCCTCTAGTTCATTATGCTCT

CATGTTTGGGTTAACCAGCTGCTTCGCAATCTGTTTACAAAGTTCTCATA

CCGGAAGTGATGATTATGTGTTGGAGTTTTTTCTGCCACCTAGCATCACA

AACTTTTATGAACAAAAGGATTTGTTGGGATCTATATTGGCAACAATGAA

GCAGAATTTCCAGAGTCTTAAGGTTGCTGCCGGTGTGGAACTCGAGGAGG

GTTGTACAATTGAAGTTGTAGAACCAATAAATGAAAGAATTCATTTGAGC

CTTGAATCTGTTCCAGTTGCTCAATCTGCTAAATCACCACAACCTACGCT

CAATGCCTCGCTAAATAAGGATGATGGAGTGCCACAAGGTCCGCTAGAAC

AGCAAATGCCGGCATGGTTGGATGATATAAATGATGGAGGGAATCTTGGT

GATAATGCAGGTGGAAGCACGAATCTGATGACTTCCTTAGAGGCTAAAAT

CAAGAAGAAACCCTCTGAAAGAAAACGTGGAAAAGCCGAGAAAATGATTA

GTCTTGAAGTTTTGCAACGTTACTTCAGTGGAAGTCTGAAGGATGCTGCA

AAGAGCCTTGGTGTTTGCCCGACGACTATGAAGCGTATCTGTAGGCAGCA

TGGAATATCTCGTTGGCCGTCAAGAAAGATCAACAAGGTTAACCGTTCCT

TGTCGAAGCTCAAGTGTGTCATTGAATCAGTCCAAGGTGCAGAAGGAGCA

TTTGCTTTGAATTCTGTAAATAAAGACCCACTTCCCATTGCTGCCGGTTC

CTTTACCGAGCCTTGTACTTCAAAAATGTTCAACCGGAATGCCTCGTTGA

GCATCCAGCCATCCAAGACTCAGATCAATGAAAATGATTTGGATACTTCG

AGAGTGTCAGAGACAAACCGACAGGTGAGGATGCAAGATCAATTCCTTGA

AGGAGAACAGAGTCCTGAGAAGGTGATCCACGAAGAAGGTTGGTCTACTC

AAGAAGTTGGCACTAAAGATCCAGAGAAGTTTAGAAATCTTAGTGGTTCA

AGTGAAGATAGCGCAAATCCCCATTCTCACGATTCATGTCATGGTAGTCC

CCCAAATGAAATTTCACCTGCAAATATATTTAGACCATTCAACAAAGAGA

AATCTGTTCCGTTAAGAGTTTCCGCAGAGTCAACAATGCAGCCAACTAAT

GCACTGAATTATGCAAATGCATATACTGCGCTTAACGTCGAGAGAACAGA

ACCTCAAGAACCATTTGGCAGAATGCTACTTGAGGGTGTTGGTAGTTCTA

AAGACTTGAGAAATTTGTGCCCTTTGGAGGATCAAGCTTTAGAAGCTTGT

GGGGTTAATCCACCATGTCATGACTTGGCTCCCAAGCAATGCATGATGGA

TATTACCCTTAATAGCAACAACACTATGATACCTTTTGCAACTAAGAAAG

AGATGAAGAGTGTGACAATTAAGGCAACATATAAAGAAGATATCATAAGG

TTTAGGGTGTCTTTGAACTGTGGCATTGTTGAACTGCAGGAAGAAATTTC

AAAGAGATTGAAACTAGAAATTGGAGCATTTGATATAAAGTATCTGGACG

ATGATAATGAATGGGTTTTGATATCATGTGATGCAGATCTTCAGGAGTGC

ATGGATGTTTTAACTTCATCAGGAAGCAACATGATAAGGCTTGTGGTGCA

TGACACAGTTTCCATTCTTGGAAGCTCATGTGAGAGCTCTGGGAACTGAG

GATAAATAGGCTTGTATATATAGTTCCTAAGCTTGTACTCAATTATTTAG

ATTGTTGTACTTATGCAAGGATAGTTTTTTCCCCCAAAATTTTTTAAAGG

ATTGTGGTTTCGGATCAGTTCAAATTGCATAGCATGACAGTTTCCAATTC

TTTAAGATTATGTGATTCAAAAATGTATGTACCCACTAAAAATCAACTAC

TAGTTTTC

**14.AhNPR4**

CACATAGCCATATTTCTCTGTACTTCGCTGTTCCCACCCCTTCTCTCTCT

CTCTCTTCACCATAATCACAAAGTTCTAATCTTTATCACTCTGCAACTCC

AAATTCTCCTTCAAAACAATCCCTTTTCTCTTCTTTGAGAATGCCAGACT

CCTGTGAAGAAAAATCTGAACTTCCTTCCAAGTCAAAGCCACAAGAAGAA

CATGGTTTTCCCATGGATTTTGATATATACCTTGAAAGTTCATCATGGCC

CATGGATCACACCCCTTCTGCTTCCAACCCCATGTCTCCTTTCATCATAA

CAACCTCCTCTGAACAACCTTTTTCTCCTCTCTGGGCTTTTTCTGATGTT

GAAGATGATCACAGGCATGTCAGGGTTGTTGCAGGTGATAATACAAATAC

AAATACAGCAATTGAAACTGAAAATCCGGTTGAAAATGATGACAACAAAA

AAATAGTGTCACCCCACTTTGTGCCTCTTCCACCTATTAAAATTCCAGAT

GGTTATTGTCTAATTAAGGAAAGAATGACACAAGCACTTCGCCACTTCAA

ACAGTTGACTGAACAGAATTTTCTGGCTCAGGTTTGGGCACCTGTGAGGA

ATGGCAACCGCTATGCACTCACAACTTCAGGTCAACCATTTGTTCTTGAT

CCACATAGTAATGGACTACATCAGTACAGAACAGTTTCCCTGATGTATAT

GTTTCCTGTTGATGGGGAGAATGATGAAATCTTGGGACTTCCAGGTCGAG

TTTTTCAGCAGAAATTGCCAGAATGGACTCCCAATGTTCAGTATTATACA

AGTAGAGAGTACTCTCGGAGAAACCATGCACAACATTACAATGTCCGCGG

AACGTTAGCTTTGCCTGTGTTTGAATCACCAGGGCAGTCATGTGTTGGTG

TATTGGAGCTGATAATGACTTCAGAGAAGGTGAATTATGCTCCTGAGGTT

GATAAAGTCTGCAAAGCCCTTGAGGCAGTGAATTTGAGGAGTTCAGAAAT

TCTGGAACATCCATTTGCTCAGATTTGCAACGAAGGTCGCCAGAATGCGT

TAGCAGAGATTTTGGAGATATTGACAGTGGTATGTGAAACTCATAATTTA

CCTCTTGCACAAACATGGGTTCCCTGTAGGCATCGGAGTGTTTTGGCAAA

TGGCGGCGGTCTTAAGAAAAGCTGTTCTAGTTTTGATGGCCACTGCATGG

GGCGAGTTTGCATGTCTGCCTCCGATGTAGCATTCTATGTCATTGATGCT

CATACATGGGGTTTCCACGATGCTTGCGCCGAGCATCACTTACAACAAGG

TCAAGGTGTTGCTGGCAGGGCATTTTTATCCCATAACATGAGCTTCTGTG

GAAACATAACTCAATTCTGCAAAACTGATTATCCTCTAGTTCATTATGCT

CTCATGTTTGGGTTAACCAGCTGTTTCGCAATCTGTTTACAAAGTTCTCA

TACCGGAAGTGATGATTATGTGTTGGAGTTTTTTCTGCCACCTAGCGTCA

CAAACTTTTATGAACAAAAGGATTTGTTGGGATCTATATTGGCAACAATG

AAGCAGAATTTCCAGAGTCTTAAGGTTGCTGCCGGTGTGGAACTCGAGGA

GGGTTGTACAATTGAAGTTGTAGAACCAATAAATGAAAGAATTCATTTGA

GCCTTGAATCTGTTCCAGTTGCTCAATCTGCTAAATCACCACCACCTACG

CTCAACGCCTCGCTAAATAAGGATGATGGAGTGCCACAAGGTCCGCTAGA

ACAGCAAATGCCGGCATGGTTGGAAGATATAAATGATGGAGGGAATCTTG

GTGATAATGCAGGTGGAAGCATGAATCCGATGACTTCCTTAGATGCTAAA

ATCAAGAAGAAACCCTCTGAAAGAAAACGTGGAAAAGCCGAGAAAATGAT

TAGTCTTGAAGTTTTGCAACGTTACTTTAGTGGAAGTCTGAAGGATGCTG

CAAAGAGCCTTGGTGTTTGCCCGACGACTATGAAGCGTATCTGTAGGCAG

CATGGAATATCTCGTTGGCCGTCAAGAAAGATCAATAAGGTTAACCGTTC

CTTGTCGAAGCTCAAGTGTGTCATTGAATCAGTCCGAGGTGCAGAAGGAG

CATTTGCTTTGAATTCTGTAAATAAAGATCCACTTCCCATTGCTGCCGGT

TCCTTTACCGAGCCTTGTACTTCAAAAATGTTCAACCGGAATGCCTCGTT

GAGCATCCAGCCATCCAAGACTCAGATGAATGAAAATGATTTGGATACTT

CGAGAGTATCAGAGACAAACCGACAGGTGAGGATGCAAGATCAGTTGCTT

GAAGGGGAACAGAGTCCTGAGAAGGTGATCCATGAAGAAGGTTGGTCTAC

TCAAGAAGTTGGCACTAAAGATCCAGAGAAGTTTAGAAATCTTAGTGGTT

CAAGTGAGGATAGTGCAAATCCCCATTCTCACGATTCATGTCATGGTAGT

CCCCCAAATGAAATTTCACCTGCAAATATATTCAGACCATTCAATAAAGA

GAAATCTGTTCCGTTAAGAGTTTCGGCAGAGTCAACAATGCAGCCAACTA

ATGCACTGAATTATGCAAATGCATATACTGCGCTTAACGTCGAGAGGACA

GAACCTCAAGAACCATTTGGCAGAATGCTACTTGAGGGTGTTGGTAGTTC

TAAAGACTTGAGAAATTTATGCCCTTTGGAGGATCAAGCTTTAGAAGCTT

GTGGGGTTAATCCACCATGTCATGACTTGGCTCCCAAGCAATGCATGATG

GATACTACCCTTAATAGCAACAACACTATGATACCTTTTGCACCTAAGAA

AGAGACGAAGAGTGTGACGATTAAGGCAACATATAAAGAAGATATCATAA

GGTTTAGGGTGTCTTTGAACTGTGGCATTGTTGAACTGCAGGAAGAAATT

TCAAAGAGATTGAAACTAGAAATTGGAGCATTTGATATAAAGTATCTGGA

CGATGATAATGAATGGGTTTTGATATCATGTGATGCAGATCTTCAGGAGT

GCATGGATGTTTTAACTTCATCAGGAAGCAACATGATAAGGCTTGTGGTG

CATGACACGGTTTCCATTCTTGGAAGCTCTTGTGAGAGCTCTGGGAACTG

AGGATAAATAGGCTTGTATATATAGTTCCTAAGTTTGTACTCAATTATTT

AGATTGTTGTACTTATGCAAGGATAGTTTTTTCCCCCAAAATTTTTTAAA

GGATTGTGGTTTCTGATCAGTTCAAATTGCATAGCATGACATTTTCCAGT

TCTTCAAGATTATGTGATTCAAAAATGTATGTACTCACTAAATATCAACT

ACTAGTTTTC

**15.AhNPR5**

AGGCATTGAAGGTAGAAAAAAATTGACATGGAATCACACTCTCTATGCCA

TTACCACCATCATCGTCATCGTCATCACCATTCATCTCTTCTATGCTCTG

CACAACTTGGTCTTCACAAAGTTGAAGTCTTTGTCACACTTCACGCACGG

CACAAGCACAAAGTTTCTTGCTTTTCCACAACATAGCACTCTCAGATTCA

GAGCAACATAACAATGTCAGAATCTGAAGAAGATAAAACAGACTTTGCTT

CTCCAAAGTCAAAGGAGGAACAACAACAACAATCACCACCTCAACTTCCT

CCTCCTTCCGCCATGGATTTCGATCTGGACTTGGAAACTTCGTGGCCTTT

GGATCACTTGTCCTTCGTCTCCAATAACCCCATGTCACCTTTTCTGTTCC

CAATCTCATCTGAACAACCTTCTTCTCCTCTTTGGCTCTTCTCTGATGCA

GAAGATGAAAGGCACAACAACACTCTTGCTTCAGCTCCAGCTTTCTCTGA

TTTCCACAAGATATTCTCTTGTGATTCAAACTCAGTAACTGAAAAACCAG

TGGAGAATGCTAATGATGAAGACAAGAAACTGTTGCCACCCATTGTGGCT

ATGCCACCATTGGAAATTTTGGATAGATATTGTGTAATAAAGGAAAGGAT

GACACAAGCGCTTCGATACTTCAAAGAGTTGACAGAACAGAATGTTCTGG

CGCAGGTTTGGGCACCTGTTAGAAATGGTAATAGGTTTGTGCTTACAACT

TCAGGTCAACCATTTGTTCTTGATCCACATAGTAATGGACTCCACCAGTA

TCGAACGGTGTCCCTGATGTACGTGTTTTCGGCGGATGGAGAGAAAGAAG

AAAGTCTTGGACTTCCTGGTCGAGTTTATCAGCAAAAAGTACCAGAATGG

ACACCTGATGTTCAGTATTATTCTACTAAGGAGTATCCTCGCAGAGATCA

TGCACAACATTACAATGTCCGCGGTACCTTGGCTTTGCCTGTCTTTGAAC

CTTCAATGCAGTCTTGTGTAGGTGTGTTGGAGTTGATCATGACTTCACAG

AAGATTAACTATGCTCCTGAAGTTGACAAAATTTGCAGAGCTCTTGAGGC

AGTGAATTTGAAGAGTTCAGAAATTTTAGGCCACCAATACACTCAGATTT

GTAATGAAGGCCGCCAGAATGCGCTAGCAGAGATCTTGGAGATATTGACA

GTGGTTTGCGAAACTCACAATTTACCTTTGGCGCAAACATGGGTTCCATG

TAGGCATCGGAGTGTTCTGGCCCATGGAGGTGGCCTAAAGAAAAGTTGTT

CAAGTTTTGATGGTAGTTGCATGGGGAAAGTTTGCATGTCTACAACTGAT

ATAGCATTCTATATCATAGATGCTCATTTATGGGGTTTCCGAGAGGCCTG

TGTCGAACATCACTTACAGCAAGGTCAAGGGGTTGCTGGCAGGGCTTTTT

CTTCACATAGCATGAGCTTCTGCAGGAACATTACCCGATTCTGCAAAATT

GATTACCCTTTAGTTCATTATGCTCTCATGTTTGGGTTAACTAGCTCCTT

TTCAATCTGTTTGCGAAGCTCGCACACCGGAGATGATGACTATGTATTAG

AGTTTTTTCTGCCACCTAGGATCACAGACTTTAACGAACAGAAGGCTTTG

TTGGGATCCATATTGACAATAATGAAACAACATTTCCAGAGCCTTAAGAT

TGCTTCTGGTGTTGAACTTGAACAGAATGCTTTGGTTGAAACTATTGAAG

CAACAATTGAAGGAGTTCATCTGAGGTTTGAATCTATTCCAGTTAGACAG

GATGCTTCACCAAATGTGAGGGAGGAATTGGCACAAGATCCGTCGTTGCA

GAAAATAATGATGGGCTGTAATGATGGAGGGAGTATTGGTGATCAGATAC

CCTCCTTAGAAACTAAAAACACAAATAAGCCATCAGAGAGGAAACGTGGG

AAAACTGAGAAATCAATAAGTCTTGAAGTTCTACAACGTTATTTTGCTGG

GAGTCTTAAGGATGCTGCAAAGAGCCTTGGTGTTTGCCCCACTACAATGA

AGCGCATCTGCAGGCAGCATGGGATATCCCGTTGGCCATCTCGAAAGATC

AACAAAGTTAACCGTTCCCTGTCCAAGCTCAAGCGTGTTATTGAATCAGT

CCAAGGTGCCGAAGGAGCATTCACTTTGAATCCTCTGAGCACAAGTCCTC

TTCCCTTTCCTGAGCATTCTACTCCAAACAAGTTCAGCCAGCAAGCCTCG

CCAACTGAACCTCAGATAAGAGAAAATGAATTAGATGCCTCTAAAGTTTT

AGAAACAACCAGAATTGCCAGAGCACAGTGTCTCGAGAAAATGGTTAATG

ATAAAAGCGGTTCTATTCGGGAGGTAGGGAAGGAAACAAAGGGGCCTAGA

GCAAAGAGTTGCTCTAGTGCAGATAGCACAAATCCTACTTCTCATGGTTC

ATGCCATGGTAGCCCTCCTATCGAAAGCTCGCCCGTAAAAGATATATTCA

TTACATCAAACAATGATCAATGTGTTGGTCTAAGGTCACCAGAGGCAACA

ATGCAGCCGCCGAATAATACACTGAGCTACCCAACGACTTGCGCTATGCC

TGATATGGTGGCAACAGAACTTCAAGAGCCATTCGGAGGAATGCTAGTAG

AAGATGCCGGAAGTTCTAAAGACTTGAGAAACCTATGTCCTTCTGTAGCT

GAGGCAATTGTGGAGGACATGGCCCCGGAACCTTGCCGGACTAATCCTCC

ATTTTCTGGGTTGGCTCCCAAACAATGCATGGATCCCCTTAAGGAGACAG

TGACACCTTTTGCATCTAGAAAAGAAATGAAGACTGTCACTATTAAGGCA

ACATATAGAGAAGATATCATAAGGTTTAGGGTTTCTTTGAATTGTGGAAT

TGTGGAACTGAAAGAAGAAGTTGCCAAAAGGTTGAAACTAGAAGTAGGTA

CTTTTGATATCAAGTACCTTGATGATGATCATGAATGGGTTTTGATAGCA

TGTGATGCAGATCTACAAGAGTGCATAGATGTTTCAAGATCATCAGCAAG

TAACATAATAAGAGTTTTGGTGCATGAAATAACTTCCCATCTTGGAAGCT

CATGCGAGAGCTCAGGGGAGTAAGTAGTAGCTGTATATAGCTCAAGTTTG

TACTCAAATGTTTAGGTTCTTGTGATCTTAGGAAGGTAGGACAAAAAAGA

AAAAAAAAAGGAGATTTTGGTTAAGATGATCTCAATTTGTAAGAAACTTA

GATGAAAGGATTAGCAATCCTTGGTATGTATGACCCCAAGTGTAAGTTAC

CATTTATTTATCATGAGATAAAGCTAGGATTCTTTGGTAACACATTTATT

ATGGTTAACTTTTTGACAAAGTTTCATATCGGTATTCATTTTGTTCCAGA

AAGTGATTACTCAAACTAA

**16.AhNPR6**

AGGCATTGAAGGTAGAAAAAAATTGACATGGAATCACACTCTCTATGCCA

TTACCACCATCATCGTCATCGTCATCACCATTCATCTCTTCTATGCTCTG

CTCTGCACAACTTGGTCTTCACAAAGTTGAAGTCTTTGTCACACTGCACG

CACGGCACAAGCACAAAGTTTCTTGCTTTTCCACAACATAGCACTCTCAG

ATTCAGAGCAACATAACAATGTCAGAATCTGAAGAAGATAAAACAGACTT

TGCTTCTCTAAAGTCAAAGGAGGACCAACAACAATCACCACCTCAACTTC

CTCCTCCTTCCGCCATGGATTTCGATCTGGACTTGGAAACTTCGTGGCCT

TTGGATCACTTGTCCTTCGTCTCCAATAACCCCATGTCACCTTTTCTGTT

CCCAATCTCATCTGAACAACCTTCTTCTCCTCTTTGGCTCTTCTCTGATG

CAGAAGATGAAAGGCACAACAACACTCTTGCTTCAGCTCCAGCTTTCTCT

GATTTCCACAAGATATTCTCTTGTGATTCAAACTCAGTAACTGAAAAACC

AGTGGAGAATGCTAATGATGAAGACAAGAAACTGTTGCCACCCATAGTGG

CTATGCCACCATTGGAAATTTTGGATAGATATTGTGTAATAAAGGAAAGG

ATGACACAAGCGCTTCGATACTACAAAGAGTTGACAGAACAGAATGTTCT

GGCGCAGGTTTGGGCACCTGTTAGAAATGGTAATAGGTTTGTGCTTACAA

CTTCAGGTCAACCATTTGTTCTTGATCCACATAGTAATGGACTCCACCAG

TATCGAACGGTGTCCCTGATGTATGTGTTTTCGGCGGATGGAGAGAAAGA

AGAAAGTCTTGGACTTCCTGGTCGAGTTTATCAGCAAAAAGTACCAGAAT

GGACACCTGATGTTCAGTATTATTCTACTAAGGAGTATCCTCGCAGAGAT

CATGCACAACATTACAATGTCCGCGGTACCTTGGCTTTGCCTGTCTTTGA

ACCTTCAATGCAGTCTTGTGTAGGTGTGTTGGAGTTGATCATGACTTCAC

AGAAGATTAACTATGCTCCTGAAGTTGACAAAATTTGCAGAGCCCTTGAG

GCAGTGAATTTGAAGAGTTCAGAAATTTTAGGCCACCAATACGCTCAGAT

TTGTAATGAAGGCCGCCAGAATGCGCTAGCAGAGATCTTGGAGATATTGA

CAGTGGTTTGCGAAACTCACAATTTACCTTTGGCGCAAACATGGGTTCCA

TGTAGGCATCGGAGTGTTCTGGCCCATGGAGGTGGCCTAAAGAAAAGTTG

TTCAAGTTTTGATGGTAGTTGCATGGGGAAAGTTTGCATGTCTACAACTG

ATATAGCATTCTATATCATAGATGCTCATTTATGGGGTTTCCGAGAGGCC

TGTGTCGAACATCACTTACAGCAAGGTCAAGGGGTTGCTGGCAGGGCTTT

TTCTTCACATAGCATGAGCTTCTGTAGGAACATTACCCGATTCTGCAAAA

TTGATTACCCTTTAGTTCATTACGCTCTCATGTTTGGGTTAACTAGCTCC

TTTTCAATCTGTTTGCGAAGCTCGCACACCGGAGATGATGACTATGTATT

AGAGTTTTTTCTGCCACCTAGGATCACAGACTTTAACGAACAGAAGGCTT

TGTTGGGATCCATATTGACAATAATGAAACAACATTTCCAGAGCCTTAAG

ATTGCTTCTGGTGTTGAACTTGAACAGAATGCTTTGGTTGAAACTATTGA

AGCAACAATTGAAGGAGTTCATCTGAGGTTTGAATCTATTCCAGTTAGAC

AGGATGCTTCACCAAACGTGAGGGAGGAATTGGCACAAGATCCGTCGTTG

CAGAAAATAATGATGGGCTGCAATGATGGAGGGAGTATTGGTGATCAGAT

ACCCTCCTTAGAAACCAAAAACACAAATAAGCCATCAGAGAGGAAACGTG

GGAAAACTGAGAAATCAATAAGTCTTGAAGTTCTGCAACGTTATTTTGCT

GGGAGTCTTAAGGATGCTGCAAAGAGCCTTGGTGTTTGCCCCACTACAAT

GAAGCGCATCTGCAGGCAGCATGGGATATCCCGTTGGCCATCTCGAAAGA

TCAACAAAGTTAACCGTTCCCTGTCCAAGCTCAAGCGTGTTATTGAATCA

GTCCAAGGTGCCGAAGGAGCATTCGCTTTGAATCCTCTGAGCACAAGTCC

TCTTCCCTTTCCTGAGCATTCTACTCCAAACAAGTTCAGCCAGCAAGCCT

CTCCAACTGAACCTCAGATAAGAGAAAATGAATTAGATGCCTCTAAAGTT

TTAGAAACAACCAGAATTGCCAGAGCACAGTGTCTCGAGAAAATGGTTAA

TGATAAAAGCGGTTCTATTCGGGAGGTAGGGAAGGAAACAAAGGGGCCTA

GAGCAAAGAGTTGCTCTAGTGCAGATAGCACAAATCCTACTTCTCATGGT

TCATGCCATGGTAGCCCTCCTATCGAAAGCTCGCCCGTAAAAGATATATT

CATTACATCAAACAATGATCAATGTGTTGGTCTAAGGTCACCAGAGGCAA

CAATGCAGCCGCCGAATAATACACTGAGCTACCCAACGACTTGCACTATG

CCTGATATGGTGGCAACAGAACTTCAAGAGCCATTCGGAGGAATGCTGGT

AGAAGATGCCGGAAGTTCCAAAGACTTGAGAAACCTATGTCCTTCTGTAG

CTGAGGCAATTGTGGAGGACATGGCCCCGGAACCTTGCCGGACTAATCCT

CCATTTTCTGGGTTGGCTCCCAAGCAATGCATGGATCCCCTTAAGGAGAC

AGTGACACCTTTTGCAGCTAGAATAGAAATGAAGACTGTCACTATTAAGG

CAACATATAGAGAAGATATCATAAGGTTTAGGGTTTCTTTGAATTGTGGA

ATTGTGGAACTGAAAGAAGAAGTTGCCAAAAGGTTGAAACTAGAAGTAGG

TACTTTTGATATCAAGTACCTTGATGATGATCATGAATGGGTTTTGATAG

CATGTGATGCAGATCTACAAGAGTGCATAGATGTTTCAAGATCATCAGCA

AGTAACATAATAAGAGTTTTGGTGCATGAAATAACTTCCAATCTTGGAAG

CTCATGCGAGAGCTCAGGGTAGTGAGTAGTAGCTGTATATAGCTCAAGTT

TGTACTCAAATGTTTAGGTTCTTGTGATCTTAGCAAGGTAGGACAAAAAA

GAAAAAAAAAAGGGAGATTTTGGTTAAGATGATCTCAATTTGTAAGAAAC

TTAGATGAAAGGATTAGCAATCCTTGGTATGTATGACCCCAAGTGTAAGT

TACCATTTATTTATCATGAGATAAAGCTAGGATTCTTTGGTAACACATTT

ATTATGGTTAACTTTTTTACCTAAAGTTTCATATCGATATTCATTTTGTT

CCAGAAAGTCATTACTCAAACTAA

**17.AhNKLM1**

TCTAGAAGGATGCCAGTGATTGGAGTAATTGAAAATTGAAAAGGAATAGG

CCCCATCTTAAGTCACTCATAGAAAATTCAATCATGTTGACTAGTATTCA

TATTTCATAGTTGGGGGATAAATATCAAGAACGGTGAGTGGGAAACTTAG

ACATAAGCAAGCAGGTTTGACTTGAATGAATGCTGTCCAGTTAGATCTGA

AAGGAAACTAAGTCCATTGTTTCTTTTCTTCTTTTCCTTGCCTCTCACCG

CAGCCACAGCACTTGAAATTCTACACTATATATAATAAACAAACATGTCA

AAATAGAAACAAACAGAGAGAGAAGCCAAAATGAGAGTTTGGCCTGTCCT

TCTCATCTTGCTGTTGCAGTTTGTTGAACCCTCACATGGCCAGACAAAGA

GCCAACAGAACAACACAGGGTTCCAGTGCAGTGGAAGAAGCTATCCATGC

CAGGCCTATGCTTTCTACAGAGCTCAGAGTCAGTTTCTTGACCTAGCTTC

CATTGGAGACCTCTTCCAAGTCAGCCGTCTCATGATTGCAAATCCAAGCA

ACATATCCTCTAATTCAGTTTCCTCTCCTCTAATCCTAAACCAGCAACTC

TTTATTCCCTTAACATGTTCCTGCAACTCCATCAACACCACCTTTGGCTC

CATCTCTTATGCTAACATCTCCTACACCATCAAACCCAACGATACCTTCT

ACTTAGTCTCCACCAACAAGTTTGAGAACCTCACTACCTACCCTTCTGTT

GAGGTTGTTAACCCAAACCTTGTAGCAACCAACCTCCAAATTGGGGACAA

TGCTATATTCCCTGTCTTCTGCAAGTGCCCCGACAAGAACACAACTGTGT

CCAACACAAGAGCCAATTACATGATCTCCTATGTTGTCCAACCGTCCGAC

AACCTATCTTCGATTGCTTCCAGGTTTGGATCTCAGCAGAAGGCCATCAC

TGATGTCAACGGGAACAAATTCAACGTCTATGATACCATATTCGTCCCGG

TGACGAAGCTGCCGGTTCTGTCACAGCCGAATACAAGCACTGCTGCTGCT

CCTTCTCCGACTCCGGCCGGCAGCTCTGATGACAGGGCAGGCACCGTGAG

AGGGCTGGCAATTGGATTGGGGATTGCTGGTTTGCTGCTAATGGTGGTGT

GTGCGGTGTGGATGTATAGGGAGAGTGTGTTGAAGGGCAGGATGTGGGCG

GGCAGGGACGAGGAGGAGCAGAGGCAGAAGGAGGGCAGGGTGTTTTCGGG

AGGGGGAGATGGGAAAGGGAGTAAACCGATGGATGTGAAGCTGATGGCCA

ATGTGTCTGACTGCTTGGACAAGTACAGAGTATTTGGAATTGAGGAACTT

GTAGAAGCCACTGATGCCTTCAGTGACAGTTGCCTCATTCAAGGTTCTGT

TTACAAAGGTACTATTGATGGAGAAACCTATGCAATCAAGAAGATGAAGT

GGAATGCCTATGAGGAGCTCAAGATCTTACAGAAGGTTAACCATGGGAAT

CTGGTGAAGTTAGAAGGGTTTTGCATAGATTCTGAAGAAGGGAATTGCTA

TCTAGTTTATGAGTACGTGGAGAATGGATCTTTGAACTGGTGGCTGCACG

AAGAGGAAGGGAAGAATAAGGAGAAGCTAAACTGGAAGACAAGGGTGAGA

ATAGGCATAGACATAGCAAATGGTCTTCAATACATCCACGAGCACACAAG

GCCAAGAGTAGTGCACAAAGACATAAAGAGCAGCAACATTCTGTTGGACT

CAAACATGAGAGCCAAGATTGCCAACTTCGGGCTTGCCAAGTCAGGAATG

AATGCCATTACAATGCACATTGTGGGAACTCAGGGCTACATTGCTCCCGA

GTATCTGGCCGATGGCGTCGTCTCCACCAAGATGGATGTCTTCTCTTTCG

GGGTGGTGCTCCTGGAGCTCATCTCTGGAAGGGAAGCCATCGACGAGGAG

GGGAATCTACTGTGGATGAGTGCTATGAAGACCTTTGAAGGGGTAAGTAG

TGATCAAGAGAAGGGTAGGAGGGTGAGGGAGTGGATGGACAAGGCAATCT

TGAGGGACACAATATCTATGGATAGTTTGTTGGGAGTTTTGGGGATTGCT

ATTGCTTGTTTGCATAAGGAGCCTTCAAAGAGGCCTAGCATAGTGGATGT

TGTCTATGCTCTCTGCAAGAGTGATGATGCAGGGTTTGAAACCTCTGAGG

ATGGGATTGGATCCCCAAAGGTCACTGCTAGGCCCACGATGATGTGGATT

CATAAGAACACTCCAGAAGGGCACCCAGTGGAACAATCTTATGTTGTTGT

CATTCTTTTGGCAAGAAATATTGCTGCTGCTCCTTCTTCCCTTCGTAGAT

TCGGACGGCGATATGCGATGCTATTTCTCGGAGGAGCAGATGCAAGAGAG

GCTCTTGTTTATGCAGATATGATAGCTGCAAACCAAGATGCTTCTCTTAC

TGTGATTCGATTCTTGTCCGCAAACTATGTAGGGGACAAAGAAAGGGAGA

AGAAGCTAGATGATGGAATTGTGACATGGTTTTGGGTTAAGAATGAGACA

AACAACAGAGTGAAGTACAGAGAAGTGGTGGTTAAGAATGGAGAAGAAAC

AATTGCAAAGCAGAATTAA

**18.AhNKLM2**

TGATTGGAGTAATTGAAAACAGAAAAAGAATGGGCCCCATCTTAAGTCAC

TCATAGAAAATTTAATCATGTTGACTAGTATTCATAGTTGGGGGATAAAT

ATCAAGAACGGTGAGTGGGAAACTTAGACATAAGCAAGCAGGTTTGACTT

GAATGAATGCTGTCGAGTTAGATCTGAAAGGAAACTAAGTCCATTGTTTC

TTTTCTTCTTTTCCTTGCCTCTAACCGCAGCCACAGCACTTGAAATTCTA

CACTATATATAATAAACAAACATGTCAAAATAGAAACAAACAGAGAGAGA

AGCCACAATGAGAGTTTGGCCTGTACTTCTCATCTTGCTGTTGCAGCTTG

TTGAACCCTCACATGGCCAGACAAAGAGCCAACAGAACAACACAGGGTTC

CAGTGCAGTGGCAGAAGCTATCCATGCCAGGCCTATGCTTTCTACAGAGC

TCAGAGTCAGTTTCTTGACCTAGCTTCCATTGGAGACCTCTTCCAAGTCA

GCCGTCTCATGATTGCAAACCCAAGCAACATATCCTCTGATTCAGTTTCC

TCTCCTCTAATTCAAAACCAGCAACTCTTTATTCCCTTAACATGTTCCTG

CAACTCCGTCAACACCACCTTTGGTTCCATGTCTTATGCTAACATCTCCT

ATACCATCAAACCCAACGATACCTTCTTCTTAGTCTCCACCATCAAGTTT

GAGAACCTCACTACCTACCCTTCTGTTGAGGTTGTTAACCCAAACCTTGT

AGCAACCAACCTCCAAATTGGGGACAATGCTATATTCCCTGTCTTCTGCA

AGTGCCCAGACAAGAACACAACTGTGTCCAACACAAGAGCCAATTACATG

ATCTCCTATGTTGTCCAACCGTCCGACAACCTATCTTCGATTGCTTCCAG

GTTTGGATCTCAGCAGAAGGCCATCACTGATGTCAACGGGAACAAATTCA

ACGTCTATGATACCATATTCGTCCCGGTGACGAAGCTGCCGGTTCTATCA

CAGCCGAATACAAGTACTGCTGCTGCTCCTTCTCCTACTCCGGCCGGCAG

CTCCGATGATAGGACAGGCACCGTGAGAGGGCTGGCAATTGGATTGGGGA

TTGCTGGTTTGCTGCTGATGGTGGTGTGTGCGGTGTGGTTGTATAGGGAG

AGTGTGTTGAAGGGCAGGATGTGGGCGGGCAGGGACGAGGAGGAGCAGAG

GCAGAAGGAAGGCAGGGTGTTTTCGGGAGGGAGAGATGGGAAAGTGAGTA

AACCGATGGATGTGAAGCTGATGGCCAATGTGTCTGACTGCTTGGACAAG

TACAGAGTCTTTGGAATTGAGGAACTTGTAGAAGCCACTGATGCCTTCAG

TGACAGTTGCCTCATTCAAGGTTCTGTTTACAAAGGTACTATTGATGGAG

AAACCTATGCAATCAAGAAGATGAAGTGGAATGCCTATGAGGAGCTCAAG

ATCTTACAGAAGGGGCGGAGAAAAAAACAGTGGAATCACTCTGAAGAAGG

GAATTGCTATCTAGTTTATGAGTACGTGGAGAATGGATCTTTGAACTGGT

GGCTGCACGAAGAGGAAGGGAAGAATAAGGAGAAGCTAAACTGGAAGACA

AGGGTGAGAATAGGCATAGACATAGCAAATGGTCTTCAATACATCCACGA

GCACACAAGGCCAAGAGTAGTGCACAAAGACATAAAGAGCAGCAACATTC

TGTTGGACTCAAACATGAGAGCCAAGATTGCCAACTTCGGCCTTGCCAAG

TCAGGAATGAACGCAATTACAATGCACATTGTGGGAACTCAGGGCTACAT

TGCTCCTGAGTATCTGGCCGATGGCGTCGTCTCCACCAAGATGGATGTCT

TCTCTTTCGGGGTGGTGCTCCTGGAGCTCATCTCTGGAAGGGAAGCCATC

GACGAGGAGGGGAATCTACTGTGGATGAGTGCTATGAAGACCTTTGAAGG

GGTAAGTAGTGATGAAGAGAAGGGTAGGAGGGTGAGGGAGTGGATGGACA

AGGCAATGTTGAGGGACACAATCTCTATGGATAGTTTGTTGGGAGTTTTG

GGGATTGCTATTGCTTGTTTGCATAAGGAGCCTTCAAAGAGGCCTAGCAT

AGTGGATGTTGTCTATGCTCTCTGCAAGAGTGATGATGCAGGGTTTGAAA

CCTCTGAGGATGGGATTGGATCCCCAAAGGTCACTGCTAGGTGA

**19.AhNKLM3**

ATGGCTTTCTTTCTACCCTCTCTCTCAAGTAGTATTTTTCTTGCATTCAT

GTTGTTCTCCGTCACCAGCATCCCAACTCAATCACAACAGGTTAATGGAA

CAGACTTTTCATGCCCAGTGGATTCACCTTCATCCTGTGGAACATATGTG

ACATACATTGCTAAATCTCCAAACTTCTTGAGCCTTTCTAACATATCTGA

CATATTTGACACCAGCCCTTTATCCATTGCAAGAGCAAGTAACATAAAGA

ATGAGGGTGACAAGCTGGTTCCAGGCCAAGTCTTACTGATACCTGTCACT

TGTGGTTGCACTCAAAACCAATCTTTCGCCAATATTACCTATGAGCTAAG

GCAGGGTGATGTGTACGACATTGTCTCAAAAACAACATATGAGAATCTCA

CAAATTGGCGTGCTGTCAACAATTCAAACCCAGATTTGAATCCAGTTCTG

CTGCCAATAGGTGTGAAAGTATTGTTCCCTTTATTCTGCAGGTGCCCTTC

TAAGAAACAGTTACAGAAAGGGATAGAATATATGATCACCTATGTGTGGC

AGAACAATGACAATGTTTCCTCTGTAGCAGCCAAGTTTGGTGCATCGCCG

GTGGACATATTGTCCGAAAACAACTACGGTGGAAACTTCACAGCTGCAAC

CTATCTTCCGGTTTTAATTCCTGTGACGAAGTTGCCAGTTCTTACTCAAC

CCGAGGCTTCACATGGAAGAAAGAGAAGCATTCAAATCCCTGTTATAATT

AGTATTAGCCTGGGGTTCACCCTTGTTGTTGCTGTTATAGTAATATCAAT

GGTTTATGCTTATCTTTATCAGAGAAAGAGGACTTTGAATAGGAGAGACT

TATCTGCTGGGACAGCAGATAAGCTACTCTCTGGAGTTTCAGGCTACGTG

AGTAAGCCAACCGTGTATGAAGCCAATGAGGTTATCAAAGCCACCATGAA

TCTCAGCGAACAGTGCAAGCTTGGGGGCACAGTTTACAAGGCCAAAATAG

AAGGGCAGGTCTTGGCAGTGAAAAAAGTGAATCAAGTAGTTTCTGAGGAG

CTGAATATTCTGCAGAAGGTGAATCATGGAAACCTGGTGAAACTGATGGG

TGTATCTTCAGACAGTGATGGAAACCATTTCCTGGTTTATGAGTATGCTG

ATAACGGGTCCCTTGATGAGTGGCTCTTCTCCAAGTTGTCTTTGAAGGCC

TCGCTTACATGGTATCAGAGGATTAACATAGCATTGGATGTTGCCATGGG

TCTGCAATACTTGCATGAGCACACTTATCCAAGAATAGTCCATAGGGACA

TCACAACAAGTAACATCCTTCTTGACTCCAACTTCAAGGCCAAGATAGGG

AACTTCTCCATGGTCAGAACTACTACGAATCCCATGATTTCCAAGATCGA

TGTCTTTGCTTTCGGGGTTGTTCTCATTGAGTTGCTGACAGGCAAGAAAG

CCATGACAACAAAGGCAGATGGCGAGGTAGTAATGCTGTGGAAGGATATT

AGGAAGATGTTTGAAGTGGAAGACGAAAAGGAAAAGGAGGAGTGTCTGAG

AAGATGGATGGATCCTAAGCTAGAGTGCCTTTACCCTGTGGATTATGCTC

TCAGCTTGGTCACGTTGGCCGCGAATTGCACGGCCGATGTATCATTGTCT

AGACCAACCATGGCAGAAGTTGTTCTTGGCCTCTCCCTTCTCACTCAACC

ATCTCAAGCTGCACTAGAGAGATCATTGACTTCTTCTGCGTTGGAAGCAG

AGGTTACTCATGTGGCTACTCCCATAGCAGCACGTTAA

**20.AhNNup1**

GAAAAAAATATTAGCCTACAAATTTCTTTTGCTTATTAATTTCATTTAAT

CTTTTAGCACCTAAGAAATTTGCTGCCACTCAGCACTACCCTTTTTGTCC

TTTAGTCAATGGGGTTGCTTTCAGCTAAGGCTAATACTGGTTTCTTTTTT

CATACAAACCCAAAACCCTAAAAATCACAGTCCTTTTTGAAATCATGTCC

CGCGTCGCCTCCGACACCGTCGGCAATGGCGCTCTGGTCCCATTCTCCGA

AGACACCAAAGACTCACTGGCGGTCTACCCTCTCCACCACGGCCTCGCCC

CTCCAATTTCCCGCATTGCCATCTCCTGGGCCCGCGGCAACTCCCTCCGC

GTCTCCCTCTTCGCCGCTCCCTCTTCAGAACATTCTCGAACCCCGCAGGA

TCATTCTGGAGGTAAGGTCCTCGAAGTTAAGCTTGGCGTCGGAGACCCGG

AGATTTCCGATTCCCGTTGGCGCCAAATCGCGTACGGCTCCGTTGCCCCC

TTTGCCCTGCTCCAGAGTCGCCGGAGTGCACTTTCGGAAATGATCAAGTC

TTCTTCGCCGTATCAGATGGATTGGTGGGAGAATGTGCTTGAGTATAGCA

AGGACATAACTTCACTTCTCGGTGGACCAAAGTTGCCACCTGGTCCAATA

ATTGAAGAACGAACTGATATCGTTAAGAAACGTGAGGAGCCAACATGTTT

GAAAGCAGCATGGGAGTTGTTGGAAATATTTTATGCAGACAAGCAATCTC

AAGCATGGTTACCTGAAAAGCTTGTTGATTGGTTAGCTGATTATGACAGC

CTCTTTTCAAGCACACATGAAACAGTTTATGGCAAGCTTGCTCGGTTTCA

GAAGGAGCTTGTCAACATACAGGTGCTTGAGGATGACCCTAGATATTGGG

AAGTGATGTCATCTGCACTCTCAGTTGGTTGGCTTGATATTGTGGTGAAA

ATGCTGCGGTTGCATGGATCTTATCAACTAGATCAGCTTAGTAATCGTGA

GTTAGAGAATGGGCTTGTGGAGGCAGTTGCTGTTCTTATTTCCAAAATGC

CCCGCCTATGTCATGAATCTACTAATGGAAAATTAGGTGAACTCTTTAAA

TCCAAGCCTGACTTCATCAAGGCCTGGGAAAAATGGAGATCTCAAATTAC

AAAACTGGATTGTAGTCCATTCTGGATTCAATGTGATAATCATCATACAC

GTGAGGGATTGAGAAACTTGCTACAAATTATGCTGGGCAACACTGAAAGT

CTCTGCATGGCTACATGTTACTGGATTGAGTTGTATATTTCTCATTTTCT

TTACATCAGGCCATTTACAACGGGAATAGAAAGCATGTATAATTTGGCAC

AAAAATGCATCCAACTGAAACCACCATCAAGTAACCATAAGTTGACTGGA

CTTATAGTTGGAATTCTTGAAGAAAATACTGAGGTTGTTTTAGCAGAATC

TTCTAGAGAATTTGGCCCTTGGATGGTTGCACATGCGATCGAATTGTTGA

CTGCAGGGAGTGAGCAAGCAGAGATTGTTCTACATGATGAGCGTTATAAT

TTGGGGGGAATCAGCATGATAGAACTACATCGTCTTGTGTATGCTCAAGT

ATTATCATCACATGCCTTGACCTGGCAAATAGCTCCAATATATTTGACAT

CATGCATGAAGCAAGGAATGGGCTTGTTAGAGAATTTGTTGTACAGGCAA

TCCATTCAACATAATGACTTGTTGCTAAAGAACATTGAGATCTGCCGCTT

GTATGAGCTTGATCATATTAGTTCCGATATCATGAAGGTTGCTGGAGTAT

ATCACTGGAAGCATGGTCATAAAGGTGCTGGTGTATATTGGCTTCAACGG

TCCAAAGATACTAGTCGTCTTAATCGGATTGCTCAACAATTATTTGATTC

TGTTGGAAAGTCAATCTCTGATGAAAGCTTTAAGCAATGGGAAGGTCTAA

TTGAATTATTGGGTTCTGAATCCAAGCCTGCTGGGGGTCTTGAATTTTTG

CACAAGTATAGGGATTTCAAAAGATCCCTACAGCAGATATATGGTGGAAA

GTCAACGGATGCTGCCCGACAAGCAGTAGGCTCTCTCATACTGCTGATGA

AAAATCCATCAACCCCTCAGCGCTTTTGGCTGCCGCTTCTGTATGACTCG

TTGAAGCTGTTTAATTGGAAAGAGTGTCCTCTTCTAAATGTCTCTGAGAC

CAATCTTCTGTTGAACAAACTTCAAGAGTTATCTTGGGCGAGGTTGCGCC

CGCACTTCTCTGAACCTAACCTACCTGCCGAGGCACTGAGCTCTATTAGG

CTGGCTCTGGCAACAAATCTCGGTCGTGCTATACTTGATGAATAAGCTGA

CACTAATTTATCGATGAGCGTCAATATCATTGAACCCTCGGTCCCTCTCT

TTAGCTTTCAGAATGCAAAAGGTGGTGTTTGAAGTTGCTAATTGCTATGC

TCCATAATTGTCACCTGCTGTAAATTTGTATGGACGATTGGTTAGTTTGT

ACGATTGAGCAATAGCTGTATTACTATTATC

**21.AhNNup2**

TATTATTTGTGTTCAGCACTACCCTTTTTGTCCTTTAGTCAATGGTGTTG

CTTTCAGCTAAGTGCTAATACAATAGTGTTTCTTTTTCATACAAACCCAA

AACCCTAAAAATCACAGTCCTGTTTGAAATCATGTCCCGCGTCGCCTCCG

ACACCGTCGGCAATGGCGCTCTGGTCCCATTCTCCGAAGACACCAAAGAC

TCACTGGCGGTCTACCCTCTCCACCACGGCCTCGCCCCTCCGATTTCCCG

CATTGCCATCTCCTGGGCCCGCGGCAACTCCCTCCGCGTCTCCCTCTTCG

TCACTCCCTCTTCAGAACCTTCTCAAACCCCGCAGGATCAGTCTGGAGGT

AAGGTCCTCGAAGTTAAGCTTGGCGTCGGAGACCCGGAGATTTCCGATTC

TCGTTGGCGCCAAATCGCGTACGGCTCCGTTGCCCCCTTTGCCCTGCTCC

AGAGTCGCCGGAGTGCACTTTCGGAAATGATCAAGTCTTCTTCGCCGTAT

CAGATGGATTGGTGGGAGAATGTGCTTGAGTATAGCAAGGACATAACTTC

ACTTCTCAGTGGACCAAAGTTGCCACCTGGTCCAATAATTGAAGAACGAA

CTGATATCGTTAAGAAACGTGAGGAGCCAACATGTTTGAAAGCAGCTTGG

GAGTTGTTGGAAATATTTTATGCAGACAAGCAATCTCAAGCATGGTTACC

TGAAAAGCTTGTTGATTGGTTAGCTGATTATGACAGCCTCTTTTCAAGCA

CACATGAAACAGTTTATGGCAAGCTTGCTCGGTTTCAGAAGGAGCTTGTT

GACATACAGGTGCTTGAGGATGACCCTAGATATTGGGAAGTGATGTCATC

TGCACTCTCAGTTGGTTGGCTTGATATTGTGGTGAAAATGCTGCGGTTGC

ATGGATCTTATCAACTAGATCAGCTTAGTAATCGTGAGTTAGAGAATGGG

CTTGTGGAGGCAGTTGCTGTTCTTATTTCCAAAATGCCCCGCCTATGTCA

TGAATCTACTAATGGAAAATTAGGTGAACTCTTTAAATCCAAGCCTGACT

TCATCAAGGCCTGGGAAAAATGGAGATCTCAAATTACAAAGCTGGATTGT

AGTCCATTCTGGATTCAATGTGGTAATCACCATACACGTGAGGGATTGAG

AAACTTGCTACAAATTATGCTGGGTAACACTGAAAGTCTCTGCATGGCTA

CATGTTACTGGATTGAGTTGTATATTTCTCATTTTCTTTACATCAGGCCA

TTTACAACGGGAATAGAAAGCATGTATAATTTGGCACAAAAATGCATCCA

ACTGAAACCACCATCAAGTAACCATAAGTTGACTGGACTTATAGTTGGAA

TTCTTGAAGAAAATACTGAGGTTGTTTTAGCAGAATCTTCTAGAGAATTT

GGTCCTTGGATGGTTGCACATGCGATCGAATTGTTGACTGCAGGGAGTGA

GCAAGCAGAGATTGTTCTACATGATGAGCGTTATAAATTGGGGGGAATCA

GCATGATAGAACTACATCGGCTTGTGTATGCTCAAGTATTATCATCACAT

GCCTTGACCTGGCAAATAGCTCCAATATATTTGACATCATGCATGAAGCA

AGGAATGGGCTTGTTAGAGAATTTGTTGTACAGGCAATCCATTCAACATA

ATGACTTGTTGCTAAAGAACATTGAGATCTGCCGCTTGTATGAGCTTGAT

CATATTAGTTCCGATATCATGAAGGTTGCGGGAGTATATCACTGGAAGCA

TGGTCATAAAGGTGCTGGTGTATATTGGCTTCAACAGTCCAAAGATACTA

GTCGTCTTAATCGGATTGCTCAACAATTATTTGATTCTGTTGGAAAGTCA

ATCTCTGATGAAAGCTTTAAGCAATGGGAAGGTCTAATTGAATTATTGGG

TTCTGAATCCAAGCCTGCTGGGGGTCTTGAATTTTTGCACAAGTATAGGG

ATTTCAAAAGGTCCCTACAGCAGGTATATGGTGGAAAGGCAACGGATGCT

GCTCGACAAGCAGTAGGCTCTCTCATACTGCTCATGAAAAATCCATCAAC

CCCTCAGCGCTTTTGGCTGCCGCTTCTGTATGACTCGTTGAAGCTGTTTA

ATTGGCACGAGTGTCCTCTTCTAAATGTCTCTGAGACCAATCTTCTGTTG

AACAAACTTCAAGAGTTATCTTGGGCGAGATTGCGCCCGCACTTCTCTGA

ACCTAACCTACCTGCCGAGGCACTGAGCTCTATTAGGCTGGCTCTGGCAA

CAAATCTTGGTCGTGCTATACTTGATGAATAAGCTGACACTAATTTATCG

ATGAGCGTCAATACCATTGATCCCTCGGTCCCTCTCTTTAGCTTTCAGAA

TGCAAAACGTGGTGTTTGAAGTTGCTAATTGCTATGCTCCATGATTGTCA

CCTGCTGTAAATTTGTATGGACGATTGGTTAGTTTGTAAGATTGGGCAAT

AGTTGTATTACTATTATCTACCTTTGGAATGCTTATGTAGCAGCAATAAC

ATGATATAAATAGATTTCACACTTTTTAGTTTTGAAGTCCAGGCATATCA

CCAAAGAGTATAAGCACTTCTCTTATCACCGACACAGTGAGTTACTACTT

GCCACTTTGAATGGAGATATGGCCAAAGAAATTGCTAATCGTGCATTTCT

ATGTTACACCCATTTCTCTCTTTTTCACTCTTTTATTTCTTGTCACATTT

TTATCTTTCAACAATTAAACTAGGCGAAAGCTAGTGC

**22.AhNNup3**

TTTTTTTTTTTAATAAATGTACTTTTGAAAATTTAAACCTGCACTATGAT

TTCATTTGAACAAGGGCTCTTCTCAATTTGTGTTTACTTTCGTCTCTGCA

TAGTCGTTTCCCTCTTCCGATTCAAATGCTGTCTCCTTTGAGTCTGTGAC

TGTAAGTGTGGAAGTAAGCCTAAACCTCCCACTCCGCTCATAAACCCTAA

CACGTGGCGCATCCTTAACGAATGTTTTCCTCAGCACCCAAGAAAAAGAA

CACCTATGCTACTCCACTCCGAGATCACGGTGGTGCTGCTGCCGCCGCCG

CCGCCAACCTCTTCCATTCTCCGGCAACACCTCAGTCTCGACAAAGAAGC

TCCTTCATCTTCAACGAAAACGCCGTCCCTAACCGCCCTTCTACCGGCAC

TCCCGCTCCCTGGGCTCCTCGTTTATCCGTCCTCGCCAGGGTTCCTCAAG

TTGATAGAAGTGGAAAAGGTGATGACACAGATCCAATTAAGCCGGTTTTC

GTTGCGGAGTTTCCTCAATTGGTTCGCGATGAGCAAGCTACCTTGCTTCA

CAAACGAGTTTCTGTTGAAGGTTTAGGGTCTGGTGGGATCGACAAAGACA

CTTCCCTTGCTTGGATTACTTGTGGGAATAGGGTCTTCATTTGGAGTTAC

TTGTCGCCGGCATCGGGGATGAGATGTGTTGTTCTTGAGATTCCCTCGAA

AGTTTTGGAAGACGGCGACACTGGCAAAAGTGATGCTGGGAGCTGGTTGC

TTTGTGTTGTTAATTGTGATGACACGTCTAAGGGGACGAATAAAGTCCCT

AAGCATTGCAGTTCTGCTGCTGTTATTATGTGTAATTGGAAAACTCGGGC

TGTTATTTACTGGCCTGATATATATTCTGAATCACACAATCCTCCAGTCA

CTAGCGTTGCATCCTCTGATGAGTTGGAGACTGTTTTGACTCCTGATAGG

AGAAGTTCCTTTGGCAAGCACCGGCGACAAAGTAAGGTGGGTGGTAGCTT

GAATGGATTGCACACTTTCAACTCTTTGATTGCTTCTGTGGTTCCTGGTT

GTAAGTTTGTGTGTGTTGCTCTTGCATGTAGCTCAAATGGTGAGCTTTGG

CAGTTTCACTGTGGTCCAGATGGCATTCGTCGAAGGAAAGTATATGAAAA

TGTTACGCGTTCGCCTCAGCAAGGAGGTGAATCAGGTCAAAATGTGAGCA

ACAAATGGTATCCAAGGTCATTGACATGGCGTTTTCCACATCATTCTCCT

AAGGAATCAAATCGGCAATTCTTCCTATTGACAGACCATGAGATACAGTG

TTTTAAGGTAGAACTCAGTTCTGATATGCATGTTTCAAAGCTTTGGTCCC

AGGAAATTGTTGGAACAGATGCTGAAGTTGGCATTAAGAAAGATCTTGCT

GGTCAAAAGAAAATCTGGCCTCTTGATGTGCAGGTGGATGATCATGGAAA

AGTGATCACCATTCTTGTTGTAACCTTATGTAATGACCGGATTAGCAGTT

CAAGCTACATGCAGTATTCTATTCTAACCCTGCAATATAAATCTGGGTTG

GATTCTGAGACTACAAATGACAGGATTTTGGAGAAAAAGTCTCCAATGGA

GGTGATAATCCCAAAAGCTAGATATGAAGATGAAGATTTCTTGTTTTCCA

TGAGGCTTAGAGTTGGAGGCAAGCCTTCGGGGTCTACGGTCGTAATATCT

GGGGATGGAACAGCAACAGTTTCCCATTATCATAGAAACTTAACTCGCCT

CTACAAATTTGATTTACCCTATGATGCTGGAAAGGTACTAGATGCCTCAG

TTCTTCCTTCTGCAGATGATTATGAAGAAGGTGCCTGGGTTGTATTAACA

GAGAAAGCAGGAATATGGGCAATACCAGAGAAGGCTGTCATACTTGGTGG

AGTAGAACCACCTGAGCGGAGCCTATCACGGAAGGGCAGTTCAAATGAAA

GATCTGCCCAAGAAGAGATAAGAAATCTTACAGTTGCAGGTAATTTTGCT

CCCAGAAGGGCTAGCTCTGAAGCATGGGGTACTGGAGACAGACAGAGGGC

TGTTTTAAGTGGGGTTGCACGTCGAACTGCACAAGATGAAGAATCAGAAG

CTTTACTAAATCTTCTTTTCAATGATTTTTTATCATCTGGCCAAGTTGAC

AGGGCACTTGAAAAGCTGGAAACTTCTGGTTCATTTCAAAGGGATGGAGA

AACAAATGTTTTTGTACGGACTAGCAAATCAATCATTGACACCTTAGCTA

AACACTGGACAACAACCAGAGGAGCTGAGATTTTGGCTATGGCAGTTGTT

TCCACCCAACTCTTGGAAAAGCAGCAGAAACATCAAAAGTTCCTTCAGTT

TCTTGCATTATCCAAGTGCCATGAGGAGCTGTGTTCTAGACAGAGACACG

CATTGCAAATTATATTGGAACATGGTGAAAAGCTATCTGCAATGATTCAG

CTTAGGGAACTGCAAAATATGATTAGCCAGAATCGTTCAGCTAGTGTTAA

CTCCTTGGGTTCTAGTTCGGATATACAGATGTCAGGTGCTCTTTGGGACC

TGATACAATTGGTTGGTGAGAGAGCTCGGCGTAATACTGTCCTTCTGATG

GATAGAGATAATGCTGAAGTATTCTACAGTAAGGTTTCAGATCTTGAAGA

TTTCTTTTACTGCTTAGATGCAGAAATAGAATATGTTATAAGACCAGAAC

ACCCGTTTGAAATCCAGTTTCAGAGGGCGTGTGAACTCTCAAATGCATGT

GTTACCATAATTACGACATGCTTAAACTATAAGAATGAGAATCATCTATG

GTATCCACCTCCTGAAGGTTTAACACCTTGGTATTGTCAACCTGTTGTAC

GTAAGGGTATTTGGAGTGGTGCTTCTGTTCTGCTTCGGTTGTTAAGTGAA

ATATCTGGGTTTGATAAATCATCAAAATTAGATTTGTATAGTCATTTAGA

AGCTCTAGCTGAAGTGCTACTTGAGGCATATTCAGGTGCTGTTACAGCTA

AAATTGAGTGTGGAGAAGAACATAAAGGTCTATTAAATGAATATTGGGAG

AGGCGGGATGCGCTTCTTGAATCTCTTTATCAACAGGTTAAAGAATTTGA

GGCTACCTATAAGGATTCAATTGAAGGATCCGAAGGGATGACTGGAGATG

CAATTTTGAAGATTATGTCACATCTGCTATCAATTGCTAAACGTCATGGA

TGCTACAAAGTTATGTGGACAATATGCTGTGATGTAAATGATTCAGAATT

GCTGAGAAATATTATGCATGAGAGCTTGGGTCCTGATGGGGGTTTTAGTT

ACTATGTCTTTAAGAAACTTCATGAAAGCAGACAATTCTCTGAGCTCTTG

AGGCTTGGTGAAGAATTTCCAGAGGAGCTGTCTGTTTTCTTAAAAGAGCA

TCCAGATCTTCTTTGGCTTCATGATTTGTTCCTTCATCAATATTCATCTG

CTTCAGAAACACTTCATGAATTGGCTCTAGCACAAAATGTTCAATCTACC

TCGGTTGCTGAAGAAGGAGAACAAGAGTATTCGAAGTTGAATCTAAAACT

AAGTGACAGAAAGAATCTTTTATATCTCTCAAAGATAGCTGCCTTTGCAG

CTGGTAGAGATGCTGGTACTCAAGTGAAGGTGGGCCGCATTGAGGCTGAT

TTGAAGATCCTAAAATTACAGGAGCAAGTAATGGAAGGATTCCCTTCGAT

TAAAGATATGCAACTTGTTGAACACCAGCTGCTTCATCCAGAAGACTTGA

TTAAGTTGTGCCTGGAAGGCGAAGGGCGAGAATTCTCGCTGTGGGCCTTT

GATGTGTTTGCATGGACCAGTGCCTCATTTCGCAAGGTATACAGAAGGCT

TTTGGAAGATTGCTGGAGAAAAGCTGCTAGTCAAGATGATTGGAGTAAAC

TCCATGATTCATACATAGTTGAAGGATGGAGTGATGAGGAAACCCTGCAG

AACTTGAAGAGTACCATACTGTTCCAGGCTTCAAGCAGGTGTTATGGACC

TGGAGCTGTAACTTTCGAAGAAGGGTTCGACCAAGTATTGCCCTTGAGAC

AAGAAAATATGGAGACTCCTGGGGATACAAGTTCTTCAGTTGAAGCAATA

TTGATGCAACACAAGGATTTCCCCGTTGCAGGCAAGCTTATGCTAATGGC

AATCATGTTGGGTTGTGAGGAAGGTGGTGATACCACATACGAAGAAGGGC

CTTCACCTATGGAATAGTGTATTACAATCTAGTGTAGGTAGCATGACATG

TGAAATGCAGCTTTTGTTTCGTTCCCCCTTTAACCATTACATTGCATTTC

ATAGGTAATGATAGGAGTCGTGAGCCAATATTAAAAGAGAAATGTTTCTT

TTACTTCATTTCTACTTACCATTGCTGCTCCTTACAACTTGTTTTGCGGG

AACAAAACGTTGCGTGTGCTTTTTTTTTTATTTGTAAAAATTGTATAATG

AAAATGGCTCGACGCTAATTGAATTAGGTTGGGTTAGGCGACATCTTTTT

CATTTTTTTTGGTTGATCTAAATCTAATGCTGTTGTTTTTTTAAATTTAT

TTTTCTTACGGGCACACCCTTCAGAACACAGTTACACAGACTTCGTTGTA

TCACAAAGACACATATAGTAACTGCCTAACTGATAAAACTGACACATTAG

TACATTACAGTATAGTAGTATAC

**23.AhNNup4**

TCTTCGCTATTTTTTTTTTAAATAAATGTACTTTTGAAAATTTAAACCTG

CATTATGATTTCATTTGAACAAGGGCTCTTCTCAATTTGTGTTTACTTTC

GTCTCTGCATAGTCGTTTCCCTCTTCCGATTCAAATGCTGTCTCCTTTGA

GTCTGTGACTGTAAGTGTGGAAGTAAGCCTAAACCTCCCACTCCGCTCAT

AAACCCTAACACGTGGCGCATCCTTAACGAATGTTTTCCTCAGCACCCAA

AAAAAAGAACACCAATGCTACTCCACTCCGAGATCACGGTGGTGGTGCTG

CCGCCGCCGCCGCCAACCTCTTCCATTCTCCGGCAACACCTCAGTCTCGA

CAAAGAAGCTCCTTCATCTTCAACGAAAACGCCGTCCCTAACCGCCCTTC

TACCGGCACTCCCGCTCCCTGGGCTCCTCGTTTATCCGTCCTCGCCAGGG

TTCCTCAAGTTGATAGAAGTGGAAAAGGTGATGACACAGATCCAATTAAG

CCGGTTTTCGTTGCGGAGTTTCCTCAATTGGTTCGCGATGAGCAAGCTAC

CTTGCTTCACAAACGAGTTTCTGTTGAAGGTTTAGGGTCTGGTGGGATCG

ACAAAGACACATCCCTTGCTTGGATTACTTGTGGGAATAGGGTCTTCATT

TGGAGTTACTTGTCGCCGGCATCGGGGATGAGATGTGTTGTTCTTGAGAT

TCCCTCGAAAGTTTTGGAGGACAGCGACACTGGCAAAAGTGATGCTGGGA

GCTGGTTGCTTTGTGTTGTTAATTGTGATGACACGTCTAAGGGGATGAAT

AAAGTTCCTAAGCATTGCAGTTCTGCTGCTGTTATTATGTGTAATTGGAA

AACTCGGGCTGTTATTTACTGGCCTGATATATATTCTGAATCACACAAGC

CAGTCATTAGCGTTGCATCATCTGATGAGTTGGAGACTGTTTTGACTCCT

GATAGGAGAAGTTCCTTTGGCAAGCAGCGGCGACAAAGTAAGGTGGGTGG

TAGCTTGAATGGATTGCACACTTTCAACTCTTTGATTGCTTCTGTGGTTC

CTGGTTGTAAGTTTGTGTGTGTTGCTCTTGCATGTAGCTCAAATGGTGAG

CTTTGGCAGTTTCACTGTGGTCCAGATGGCATTCGTCGAAGGAAAGTATA

TGAAAATGTTACGCGTTCACCTCAGCATGGAGGTGAATCAGGTCAAAATG

TGAGCAACAAATGGTATCCAAGGTCATTGACGTGGCGTTTTCCACATCAT

TCTCCTAAGGAATCAAATCGGCAATTCTTCCTATTGACAGACCATGAGAT

ACAGTGTTTTAAGGTAGAACTCAGTTCTGATATGCATGTTTCAAAGCTTT

GGTCCCAGGAAATTGTTGGAACAGATGCTGAAGTTGGCATTAAGAAAGAT

CTTGCTGGTCAAAAGAAAATCTGGCCTCTTGATGTGCAGGTGGATGATCA

TGGAAAAGTGATCACCATTCTTGTTGTAACCTTATGTAATGATCGGATTA

GCAGTTCAAGCTACATGCAGTATTCTATTCTAACCCTGCAATATAAATCT

GGGTTGGATTCTGAGACTACAAATGACAGGATTTTGGAGAAAAAGTCCCC

AATGGAGGTGATAATCCCAAAAGCTAGATATGAAGATGAAGATTTCTTGT

TTTCCATGAGGCTTAGAGTTGGAGGCAAGCCTTCGGGGTCTACGGTCGTA

ATATCTGGGGATGGAACAGCAACAGTTTCCCATTATCATAGAAACTTAAC

TCGCCTCTACAAATTTGATTTACCCTATGATGCTGGAAAGGTACTAGATG

CCTCAGTTCTTCCTTCTGCAGATGATTATGAAGAAGGTGCCTGGGTTGTA

TTAACAGAGAAAGCAGGAATATGGGCAATACCAGAGAAGGCTGTCATACT

TGGTGGAGTAGAACCACCTGAGCGGAGTCTATCACGGAAAGGAAGTTCAA

ATGAAAGATCTGCCCAAGAAGAGATAAGAAATCTTACAGTTGCAGGTAAT

TTTGCTCCCAGAAGGGCTAGCTCTGAAGCATGGGGTACTGGAGACAGACA

GAGGGCTGTTTTAAGTGGGGTTGCACGTCGAACTGCACAAGATGAAGAAT

CAGAAGCTTTACTAAATCTTCTTTTCAATGATTTTTTATCATCTGGGCAG

GTTGACAGGGCACTTGAAAAGCTGGAAACTTCTGGTTCATTTCAAAGGGA

TGGAGAAACAAATGTTTTTGTGCGGACTAGCAAATCAATCATTGACACCT

TAGCTAAACACTGGACAACAACCAGAGGAGCTGAGATTTTGGCTATGGCA

GTTGTTTCCACCCAACTCTTGGAAAAGCAGCAGAAACATCAAAAGTTCCT

TCAGTTTCTTGCATTATCCAAGTGCCATGAGGAGCTGTGTTCTAGACAGA

GACACGCATTGCAAATTATATTGGAACATGGTGAAAAGCTATCTGCAATG

ATTCAGCTTAGGGAACTGCAAAATATGATTAGCCAGAATCGTTCAGCTAG

TGTTAACTCCTTGGGTTCTAGTTCGGATATACAGATGTCAGGTGCTCTTT

GGGACCTGATACAATTGGTTGGTGAGAGAGCTCGGCGTAATACTGTCCTT

CTGATGGATAGAGATAATGCTGAAGTATTCTACAGTAAGGTTTCAGATCT

TGAAGATTTCTTTTACTGCTTAGATGCAGAAATAGAATATGTTATAAGAC

CAGAACACCCGTTTGAAATCCAGTTTCAGAGGGCATGTGAACTCTCAAAT

GCATGTGTTACCATAATTACGACATGCTTAAACTATAAGAATGAGAATCA

TCTATGGTATCCACCTCCTGAAGGTTTAACACCTTGGTATTGTCAACCTG

TTGTACGTAAGGGTATTTGGAGTGGTGCTTCTGTTCTGCTTCGGTTGTTA

AGTGAAATATCTGGACTTGATAAATCATCAAAATTAGATTTGTATAGTCA

TTTAGAAGCTCTAGCTGAAGTGCTACTTGAGGCATATTCAGGTGCTGTTA

CAGCTAAAATTGAGTGTGGAGAAGAACATAAAGGTCTATTAAATGAATAT

TGGGAGAGGCGGGATGCACTTCTTGAATCTCTTTATCAACAGGTTAAAGA

ATTTGAGGCTACCTATAAGGATTCAATTGAAGGATCCGAAGGGATGACTG

GAGATGCAATTTTGAAGATTATGTCACATCTGCTATCAATTGCTAAACGT

CATGGATGCTACAAAGTTATGTGGACAATATGCTGTGATGTAAATGATTC

AGAATTGCTGAGAAATATTATGCATGAGAGCTTGGGTCCTGATGGGGGTT

TTAGTTACTATGTCTTTAAGAAACTTCATGAAAGCAGACAATTCTCTGAG

CTCTTGAGGCTTGGTGAAGAATTTCCAGAGGAACTGTCTGTTTTCTTAAA

AGAGCATTCAGATCTTCTTTGGCTTCATGATTTGTTCCTTCATCAATATT

CGTCTGCTTCAGAAACACTTCATGAATTGGCTCTAGCACAAAATGTTCAA

TCTACCTCAGTTGCTGAAGAAGGAGAACAAGAGTATTTGAAGTTGAATCT

AAAACTAAGTGACAGAAAGAATCTTTTATATCTCTCAAAGATAGCTGCCT

TTGCAGCTGGTAGAGATGCTGGTACTCAAGTGAAGGTGGACCGCATTGAG

GCTGATTTGAAGATCCTAAAATTACAGGAGCAAGTAATGGAAGGATTCCG

TTCGATTAAAGATAATCAACTTGTTGAACACCAGCTGCATCATCCAGAAG

ACTTGATTAAGTTGTGCCTGGAAGGCGAAGGGCGAGAATTCTCGCTGTGG

GCCTTTGATGTGTTTGCATGGACCAGTGCCTCATTTCGCAAGGTATACAG

AAGGCTTTTGGAAGATTGCTGGAGAAAAGCTGCTAGTCAAGATGATTGGA

GTAAACTCCATGATTCATACATAGTTGAAGGATGGAGTGATGAGGAAACC

CTGCAGAACTTGAAGAGTACCATACTGTTCCAGGCTTCAAGCAGGTGTTA

TGGACCTGGAGCTGCAACTTTCGAAGAAGGGTTCGACCAAGTATTGCCCT

TGAGACAAGAAAATATGGAGACTCCTGGGGATACAAGTTCTTCAGTTGAA

GCAATATTGATGCAACACAAGGATTTCCCCGTTGCAGGCAAGCTTATGCT

AATGGCAATCATGTTGGGTTGTGAGGAAGGTGGTGATACCACATACGAAG

AAGGGCCTTCCCCTATGGAATAGTGTATTACAATCTAGTGTAGGTAGCAT

GACATGTGAAATGCAGCTTTTGTTTCGTTCCCCTTTAACCATTACATTGC

ATTTCACATTCACAATGGGAGTCGTGAGCCAATATTAAAAGAGAAATGTT

TCTTTTACTTCATTTCTACTTACCATTGCTGCTCCTTACAACTTGTTTTG

CGGGAACAAAACGTTGCGTGTGCTTTTTTTTTTTTGATGTAAAAATTGTA

TAATGAAAATGGCTCGACGCTAATTGAATTAGGTTGGGTTAGGCGACATC

TTTTTCATTTTTTTTGGTTGATCTAATGCCGTTGTTTTTTAAATTTATTT

TTCTTACGGGCACACCCTTCAGAACACAGTTACACAGACTTCGTTGTATC

ACAAAGACACATATAGTAACTGTCTAACTGATAAAACTGACACATTAGTA

CATTACAGTATAGTAGTATAC

**24.AhNKTyr**

ATGGCTTTCTTTCTACCCTCTCTCTCAAGTAGTATTTTTCTTGTATTCAT

GTTCTCCATCACCAGCATCCCAACTCAATCACAACAGGTTAATGGAACAG

ACTTTTCATGCCCAGTGGATTCACCTTCTTCCTGTGGAACATATGTGACA

TACATCGCTAAATCTCCAAACTTCTTGAGCCTTTCTAACATATCTGACAT

ATTTGACACCAGCCCTTTATCCATTGCAAGAGCAAGTAACATAAAGAATG

AGGGTGACAAGCTGGTTCCAGGCCAAGTCTTACTGATACCTGTCACTTGT

GGTTGCACTCAAAACCAATCTTTCGCCAATATCACCTATGAGCTAAGGCA

GGGTGATATGTACGACTTTGTCTCAAAAACAACATATGAGAATCTCACAA

ATTGGCGTGCTGTCAACGATTCAAACCCAGATTTGAATCCAGTTCTGCTG

CCAGTAGGTGTGAAAGTATTGTTCCCTTTATTCTGCAGGTGCCCTTCTAA

GAAGCAGTTACAAAAAGGGATAGAATATATGATCACCTATGTGTGGCAGA

ACAATGACAATGTTTCCTCTGTAGCAGCCAAGTTTGGTGCATCGGCAGTG

GACATATTGTCCGAAAACAACTATGGTGGAAACTTCACAGCTGCAACCTA

TCTTCCGGTTTTGATTCCTGTGACGAAGTTGCCGGTTCTTACTCAACCCG

AGCCTTCACATGGAAGAAAGAGAAGCATTCAAATCCCTGTTATAATCAGT

ATTAGCCTGGGGTTCACCCTTGTTGTTGCTGTTATAGTAATATCAATGGT

TTATGCTTATCTTTATCAGAGAAAGAGGACTTTGAATAGGAGAGACTCAT

CTGCTGGGACAGCAGATAAGCTACTCTCTGGAGTCTCAGGCTACGTGAGT

AAGCCAACCGTGTATGAAGCCAATGAGGTTATCAAAGCCACCATGAATCT

CAGCGAACAGTGCAAGCTTGGGGGCACAGTTTACAAGGCCAAAATAGAAG

GGCAGGTCTTGGCAGTGAAAAAAGTGAATCAAGTAGTTTCTGAGGAGCTG

AATATTCTGCAGAAGGTGAATCATGGAAACCTGGTGAAACTGATGGGTGT

ATCTTCAGACAGTGATGGAAACCATTTCCTGGTTTATGAGTATGCTGATA

ACGGGTCCCTTGATGGGTGGCTCTTCTCCAAGTTGTCTTTGAAGGCCTCG

CTTACATGGTATCAGAGGATTAACATAGCATTGGATGTTGCCATGGGTCT

GCAATACTTGCATGAGCACACTTATCCAAGAATAGTCCATAGGGACATCA

CAACAAGTAACATCCTTCTTGACTCCAACTTCAAGGCCAAGATAGGGAAC

TTCTCCATGGTCAGAACTACTACAAATCCCATGATTTCCAAGATCGATGT

CTTTGCTTTCGGGGTTGTTCTGATTGAGTTGCTTACAGGCAGGAAAGCCA

TGACAACAAAGGCAGATGGTGAGGTAGTAATGCTGTGGAAGGATATTAGG

AAGATGTTTGAAGTGGAAGATGAAAAGGAAAAGGAGGAATGTCTGAGAAG

ATGGATGGATCCTAAGCTAGAGTGCCTTTACCCTGTGGATTATGCTCTCA

GCTTGGCCACGTTGGCCGCGAATTGCACGGCGGATGTATCATTGTCTAGA

CCAACCATGGCAGAAGTTGTTCTTGGCCTCTCCCTTCTCACTCAACCATC

TCAAGCTGCACTAGAGAGATCATTGACTTCTTCTGCGTTGGAAGCAGAGG

TTACTCATGTGGCTACTCCCATAGCAGCACGTTAA

**25.AhNSur1**

GTCAACATGAAGTTTATTTGTGAAATGGTGAAATTATCATTGTCGGTTAT

AATAATACAATCGAGCATCATATTCTCACGAGAACTTGGGAGTCCAAATC

ATATCAAGACAGCTACTTTTTATACTAAAAAATTCGTATTAGAACCTGGA

AAAATTACCAGAAAAAATTTTTTCGATGTTGAGTTTCCAAGAGGCCACAC

TGGAATTAAGAATTTACAAGCTGAACTAGTCGATGAACACGGAAATTCTA

TACCACTGTATGAGGCTTACCTACATCATTATTTCGTTTTTAGATATTTT

GAGAATATCACCATGTCGCAATATGCTAATAAAAGTCAACCTGCCTATGG

TAAGTATTTTAGGAGAAACGATGGTGTATGTCAAGGTTATGTTAATTCAA

TTTCTTGGGGACTTGGAGGTGATGCACGAAAAACTAGCGTAGAACTACCA

GATCCATTTAGAGTAGAAGTAGGTATACATCCTGAGGATGTTCCAAAGGA

ATACGATGAGGAGAAATGGTTTATTAATGTTTTGGCTATTGACACACGTG

GTACAGAAGATAAGAAAGGTTGTTCCCAATGCAGATGTGATCTTTATAAT

GTCAAAAGTCAAGATTTGAAAAACACAACAAGCATTGATGGAAAATTATT

GTTTAGTAGTGATTACAAAGGAGGAATTTTTTGTTGTGAGAAAAAATCTC

AATGTAAATTACAAAAAGGATACAAAAAAGAACAAAAGAGAAAAGTTACC

CTTAAATATACAATATCATGGGTTGAATGGGATCAACACCAAGTGCCTCT

TAAGTTTTATATTCTTGATGTTACTGATCAAGTGACATATAATGGATCAA

AACCAATTCATAATTGTGCAGTAGAGTATTCTATAATTCCGGAAAAGGCT

AATGAAGAACAGTACTATATCAAAAAAACAAATGTTCCAATGGAAAAAGG

TGGTAATGTCATTTATACTACTGCTCACATACATTCAGGAATTGTTAATG

CAACTTTATATGGAGAGGATGGAAGAATATTATGTGAAATTAAGGCGATT

AGTGGAATGGGAAAAGAGGCAGGAAATGAAGAAGGTTATGCTATTGGAGC

GTCTGGGTGCTACCCAAAACCAGGCTCTATGAAGATTAAAGATGGTGAAA

TTTTAACTATAGAATTTGCACATGAAAACAAATACACTACTGGACTTATG

GGGCATTTTTATGTTTATTTGGCAGAAGAGTTGCCAAAATCTTTTTAA

**26.AhNSur2**

GTCAACATGAAGTTTATTTGTGAAATGGTGATATTATCATTGTCGGTTAT

AATAATACAATCGAGCATCATATTCTCACGAGAACTTGGGAGTCCAAATC

ATATCAAGACAGCTACTTTTTATACTAAAAAATTCGTATTAGAACCTGGA

AAAATTACCAGAAAAAATTTTTTCGATGTTAAGTTTCCAAGAGGCCACAT

TGGAATTAAGAATTTACGAGCTGAACTAGTTGATGAACACGGAAACTCTA

TACCATTGTATGAGGCTTACCTGCATCATTATTTCATTTTTAGATATTTT

GAGAATATCACAATGTCACAATATGCTAATAAAAGTCAACCTGCCTATGG

TAAGTATTTTAGGAGAAACGATGGTGTATGTCAAGGTTATGTTAATTCAA

TTTCTTGGGGTCTTGGAGGTGATGCACGAAAAACTAGCGTAGAGCTACCA

GATCCATTTAGAGTAGAAGTAGGTACGCATCCTGAGAATGTTCCAAAGGA

ATACGATGAGGAGAAATGGTTTATTAATGTTTTGGCTATTGACACACGTG

GTACAGAAGATAAGAAAGGTTGTTCCCAATGCAGATGTGATCTTTATAAT

GTCAAAAGTAAAGATTTGGAAAACACAACAAGCGTTGACGGAAAATCATT

GTTTAGTAGTGATTACAAAGGAGGAATTTTTTGTTGTGAGAAAAAATCTC

AATGTAAATTACAAAAAGGATACAAAAAAGAACAAAAGAGAAATGTTACC

CTTAAATATACAATATCATGGGTTGAATGGGATCAACACCAAGTGCCTCT

TAAGTTTTATATTCTTGATGTTACTGATCAAGTGACATATAATGGATCAA

AACCAATTCATAATTGTGCAGTAGAGTATTCCATAACTCCGAAAAAGACT

AATGAAGAACAGTACTATATAAAAAAAACAAATGTTCCAATGGAAAAAGG

TGGTAATGTCATTTATACTACGGCTCACATACATTCAGGAATTGTTAATG

CAACTTTATATGGAGAGGATGGAAGAATATTATGTGAAATTAAGGCAATC

AGTGGAACGGGAAAAGAGGCAGGAAATGAAGAAGGTTATGCTGTTGGAGC

GTCTGGGTGCTACCCAAAACCAAGCTCTATGAAGATTAAAGATGGTGAAA

TTTTAACCATAGAATTTGTACATGAAAACAAATACACTACTGGACTTATG

GGGCATTTTTATGTTTATTTGGCAGAAGAGTTGCTAAAATCTTTTTAA

**27.AhNSur3**

AAATCTGTAAATGTCACTATTATTAGAGTATCTAGCTCCATGTCTCCATG

TTTACCACCCAATACGGCAATACCCATCCATGCTAGATTAAATGAAACTA

AGAGAAGCCAACCACTCCCATCTTGCGCCACGTGCAACAGTTCCACACAC

AATTAATTCTGTTCGATTACATCAGAATTTTCCAAATGATTTTGGTTTTT

GGACTTAGTAGATTTAGCTTCATTCCTAGAAGGAATCTTGTTCACTAATC

ACTATCCATAGCATGCTCGTTTCAGTTTCACATATGATGTGACGACGTCT

CACTCATCAAATTTGCATGGCCTATAAAAATCTCTCTGGATTGTCAAAAT

TTAAACGTTGCCGTCTTGTGTTGTGCCTCTCTTGAAGGTTAACTATCAAC

ATGTCTTATGCATCTAAAGATATGGTGATTTCATTGGCAATACTATTGCT

TATGTTAGGCACACCATGCTCAAGTGCTTTTTGGAAGACTCGGAATAAGA

TTAAGACAGCTGTTTATCTTTCTCCCAAGATTGAACTTGGGCCAGGGTCA

GTTTCGAATAAATTTTACTATGATATTGAGTTTCCAAGAGGTCATGTTGC

GATTAAGAGTTTCAATGCTGAAGTAGTTGATGAAGCTGGAAACCCTGTAC

CTCTCCATAAAACTTATCTCCACCATTGGATTATTGTTGGATACCATGAA

TCCAAATCAAAACTTGCGACACACACAAAACATGATCTTCATCGTGTGGT

TTGTGTGTCAGACTCAGTCTCAAAGTCACATATTATACTAAGAAATAGTG

GCGTATGTCAGGGAAATATTCTTGGACAGTATTTTGGACTTGGATCCGAA

ACACGAGGAACGGCTACGGATATTCCAGATCCTTTTGGGATAGAAATAGG

AAATCCAGCAGAAATTCCAGAAGGATATGAGGAGAAATGGTTGCTCAATG

TCCACGCCATCGATACACGAGGTGTAGAGGATAAGCTAGGCTGCACTGAG

TGTAAGTGTCACCTTTATAATGTTACAGTCAATGAATATGGCAACCCTTT

GCCTCCAGATTACACAGGGGGTTTGTACTGTTGCTATGATGAGACTCAGT

GCAGGTTGAAGAAAGGCTTTCAAGGTCCAAAGAGAAGCCTCTATCTGAGA

TACACTGTGAAATGGATTGATTGGGACGAATATGTTGTTCCTGTTAAGAT

TTATATAATTGATGTGACTGATACTTTGAAAATATCAGATACTTCAAATA

TAGCAAGCTCAAATCATGATTGCCGGATTGAGTATGAAGTTGATCCTTGC

AACATAGACCCCAAGAAAGGTAATGGTTGTCTTGATGTGAAGAGGACAAC

CGTTCCATTGGAAAAGGGTGGTTATGTGGTCTATGCTGTAGCTCATCAGC

ATTCAGGAAATGAATCAGGTTACATTGTAGGAATGACCACTTGTTATCCT

AAACCAGGTTCTGTAAAGATCATTGATGGCGAAAAATTAACTCTGGAGTC

TAACTACAGCAGCACCACGCGAGGTCACACCGGAGTCATGGGGCTTTTCT

ACCTACTGGTTGCAGAACAGCTTCCTCATCAACATTAG

**28.AhNSur4**

ATGAAGTTTATACCTGAAATGTTATTACTAGCATTAACAATCATACTGTT

GCAATTAAACATCATATTCGCACAACAATATGAGAATTCAAATCATATCA

AGACGGCAACTTTTTATAGTGAACAATTTGTGTTGGAACCAGGAAAGGTT

ACGGTAACAGATTTATTCGATATTGAGTTTCCAAGAGGACACATCGGAAT

CAAGAATTTTCAAGCCGAGTTAGTTGATGAACATCGGAATTCTTTGCCAT

TATATGAAGCTTACCTGCACCATTATTTTGTTTTAAGATATTTTGAAAAT

GTCACCATGTCACGTCAAGCTAATCAAAGTCAGCCCATATACGGTAAGTA

TTTTAGAAGAAACGATGGAGTATGCCAGGGTAGTGTTAATTCATATTCCT

GGGGGCTTGGAGTTGATGCACGAAAAACTAGCTTAGAACTACCAGATCCA

TTTAGAATAGAAGTAGGTACGCACCCTGAGAATGTCCCAAAGGAGTATAA

TGAAGAGAAATGGTTATTCAATATCATGGTTATTGACACACGTGGTACAG

AAGACAAAAAAGGTTGCACTGAATGCAGATGTGACCATTATAATGTCAAA

AGTGAAGACTTTATAAGCAAAACCGGTATTGATGGGAAACTAATGTCTAG

TGATTATAAAGGAGGAATTTTTTGTTGTGAAAAGACTTCTCAATGCAAAT

TACAAAAAGGATATAATAACAGACAAGAGAGAAAAGCTTCCCTTAAATAT

ACAGTAACATGGGTTGATTGGGATCAATACCAAGTACCTATTAAGTTTTA

CATACTTGATGTTACTGATCAAGTGACATATAATGGCTCCGAACCAATTC

ATAATTGTATGGTAGAGTATTCTATCACTCCGCAAAATACTGATATTGGA

CATTATCATATTAAAAGGACAAAGATCCCAATGAAAAAGGGTGGTAATCT

AATCTACTCTACAGCTCACGTGCATCCAGGAATCGTTAATGCAACTTTAT

ATGTGGAGAATGGAAAGGTATTATGTGCGGTTAAACCAACATACGGCACA

GGAGAAGAGCCAGGAAATGAAAAAGGCTATGTTGTTGGAATGTCTGGTTG

TTATCCAAAACCAGGCTCTATCAAGATTCAGGATGGCGAAATATTAACTG

TTGACAAGCATTGCCAGCTTGGTGTAATATTGGCTCGAGAAAAAAAATAT

CTTCCAAGTCATTCTAGGTCAACATCCTTTAAAGGCACGAGTCAACAAGA

GCATATAGCTCAAAGTTCATCTATTCAAAATGAAGTTCATAGTCATAGCC

TATCAGATGTTGATTTTCTTAATGATGAAGAGAAATAA

**29.AhNSur5**

ATGAAGTTTATACCTGAAGTGTTATTACTATCATTAACAATTATACTGTT

GGAATCAAGCATCATATTCGCACGACAATATAAGAATTCAAATCATATCA

AGACGGCTACTTTTTATAGTGAGCAATTTGTATTGGAACCAGGAAAGGTT

ACGATAACAGATTTATTCGATATTGAGTTTCCAAGAGGACACATCGGAAT

CAAGAATTTTCAAGCCGAGTTAGTTGATGAACATCGGAATTCTTTGCCAT

TATATGAAGCTTACCTGCACCATTATTTTGTTTTAAGATATTTTGAAAAT

GTTACTATGTCACGTCAGGCTAATCAAAGTCAGCCCATATACGGTAAGTA

TTTTAGAAGAAATGATGGAGTATGCCAGGGTAGTGTTAATTCATATTCCT

GGGGGCTTGGAGTTGATGCACGAAAAACTAGCTTAGAACTAACAGATCCA

TTTAGAATAGAAGTAGGTACGCACCCTGAGAATGTCCCAATGGAGTATAA

TGAAGAGAAATGGTTATTCGATATCATGGTTATTGACATACGTGGTACAG

AAGACAAAAAAGGTTGCACCGAATGCAGATGTGACCATTATAATGTCAAA

AGTGAAGACTTTGTAAGCAAAACTGGTATTGATGGGAAACCAATGTCTGT

AACATGGGTTGATTGGGATCAATACCAAGTGCCTATTAAGTTTTACATAC

TTGATGTTACTGATCAAGTGACATATAATGGCTCCGAACCAATTCATAAT

TGTATGGTAGAGTATTCTATCACTCCACAAAATACTGACATTGGGCATTA

TCATATTAAAAAGACAAAGATCCCAATGAAAAAGGGTGGTAATCTAATCT

ACTCTACAGTTCATGTGCATCCAGGAATCGTTAATGCAACTTTATATGGG

GAGAATGGAAAGGTATTATGTGCGGTTCAACCAACATACGGCACAGGAGA

AGAGCCAGGAAATGAAAAAGGCTATGTTGTTGGAATGTCTGGAAAGGTTA

CGATAACAGATTTATTCGATATTGAGTTTCCAAGAGGACACATCGGAATC

AAGAATTTTCAAGCCGAGTTAGTTGATGAACATCAGAATTCTTTGCCATT

ATATGAAGCTTACCTGCACCATTATTTTGTTTTAAGATATTTTGAAAATG

TCACCATGTCACGTCAGGCTAATCAAAGTCAGCCCATATACGGTAAGTAT

TTTAGAAGAAATGATGGAGTATGCCAGGGTAGTGTTAATTCATATTCCTG

GGGGCTTGGAGTTGATGCACGAAAAACTAGCTTAGAACTACCAGATCCAT

TTAGAATAGAAGTAGGTACGCACCCTGAGAATGTCCCAAAGGAGTATAAT

GAAGAAAAATGGTTATTCGATATCATGGTTATTGACACACGTGGTACAGA

AGACAAAAAAGGTTGCACCGAATGCAGATGTGACCATTATAATGTCAAAA

GTGAAGACTTTGTAAGCAAAACCGGTATTGATGGGAAACCAATGTCTGGT

GATTATAAAGGAGGAATTTTTTGTTGTGAAAAGACTTCTCAATGCAAATT

ACAAAAAGGATATAATAACAAACAACAGAGAAAAGCTTTCCTTAAATATA

CAATAACATGGGTTGATTGGGATCAATACCAAGTGCCTATTAAGTTTTAC

ATACTTGATGTTACTGATCAAGTGACATATAATGGCTCCGAACCAATTCA

TAATTGTATGGTAGAGTATTCTATCACTCCACAAAATACTGACATTGGGC

ATTATCATATTAAAAAGACAAAGATCCCAATGAAAAAGGGTGGTAATCTA

ATCTACTCTACAGTTCATGTGCATCCAGGAATCGTTAATGCAACTTTATA

TGGGGAGGAGAAGAGCCAGGAAATGAAAAAGGCTATGTTGTTGGAATGTC

TGGTTGTTATCCAAAACCAGGCTCTATCAAGATTCAGGATGGAGAAATAT

TAA

**30.AhNSur6**

ATGAAGTTTATACCTGAAGTGTTATTACTATCATTAACAATTATACTGTT

GCAATCAAGCATCATATTCGCACGACAATATGAGAATTCAAATCATATCA

AGACGGCTACTTTTTATAGTGAGCAATTTGTATTGGAACCAGGAAAGGTT

ACGATAACAGATTTATTCGATATTGAGTTTCCAAGAGGACACATCGGAAT

CAAGAACTTTCAAGCCGAGTTAGTTGATGAACATCGAAATTCTTTGCCAT

TATATGAAGCTTACCTGCACCATTATTTCGTTTTAAGATATTTTGAAAAT

GTCACCATGTCACGTCAAGCTAATCAAAGTCAGCCCATATACGGTAAGTA

TTTTAGAAGAAACGATGGAGTATGCCAGGGTAGTGTTAATTCATATTCCT

GGGGGCTTGGAGTTGATGCACGAAAAACTAGCTTAGAACTACCAGATCCA

TTTAGAATAGAAGTAGGTACGCACCTGAGAATGTCCCAAAGGAGTAGAAT

GAAGAGAAATGGTTATTCAATATCATGGTTATTGACACACGTGGTACAGA

AGACAAAAAAGGTTGCACCGAATGCAGATGTGACCATTATAATGTCAAAA

GTGAAGACTTTCAAAACCGGTATTGATGGGAAACCAATGTCTAGTGATTA

TAAAGGAGGAATTTTTTGTTGTGAAAAGACTTCTCAATGCAAATTACAAC

AAGGATATAATAACAGACAACAGAGAAAAGCTTCCCTTAAATATACAGTA

ACATGGGTTGATTGGGATCAATACCAAGTACCTATTAAGTTTTACATACT

TGATGTTACTGATCAAGTGACATATAATGGCTCCGAACCAATTCATAATT

GTATGGTAGAGTATTCTATCACTCCGCAAAATACTGATATTGGACATTAT

CATATTAAAAGGACAAAGATCCCAATGAAAAAGGGTGGTAATCTAATTTA

CTCTACAGCTCACGTGCATCCAGGAATCGTTAATGCAACTTTATATAGGG

AGAATGGAATGGTATTATGTGCGGTTCAACCAACATACGGCACAGGAGAA

GAGCCAGGAAATGAAAAAGGCTATGTTGTTGGAATGTCTGGTTGTTATCC

GAAACCAGGCTCTATCAAGATTCAGGATGGCGAAATATTAACCGTTGATT

ATCTTGGTTCGAGAAAAAAACATCTTGCAAGTCATTCTAGGTCAACATCT

TCTAAAGGCACGAGTCAAAAAGAGCATATAGCTCAAAGTTCATCTATTCA

AAATGAAGTTCATAGTCATAGCCTATCAGATGTTGATTTTCTTAATGTTG

AAGAGGTGGATGTGGTTGCTATTTCTCGAAAGAAGTGGAACACCACATGA

**31.AhNSur7**

TATGGAACTATTTCTCTGTTTTCTCTAAATATTTTATCCATTTGGGTGAC

ATTTGTGATTTCAGACGTGAGGAAGTTTCTCTCCTCAAATATTTTGCCTA

TAAATCGTGGTTGCTGCTTCCGCATTCAATTGCACTCTGCCTTGTGGTGT

GGCACTCTTTGAATTGAATGTCAACCAACAAAATGTGTGAGGCGATTTCA

TTGGCAATACTCTTGCTGGTGTTAGGCAGTCCATGCTCAGCTTCTTTATT

TTCTTGGAAGACTGAGAATAAAATTAAGACAGCAGTTCATCTTTCCCCAA

AGATTGAAATAGGACCGGGAGCGGTTTCCAATAAAAATTACTATGATATT

GATTTTCCAAGAGGTCATGTTGCTCTCAAGGGTTTCACTGCTGAAGTTGT

TGATGAAGCTGGAAACTCTGTACCCCTCCATGAAACTTACCTCCACCATT

GGGCTCTTATAAGATACCGTCAATCCAAGTCTAAACTTGCAACACACGCA

AGCTATGATCCTCATCGAGTTCTTCATGTGTCAGACTCAATCTTCGAATC

AGGGGTTGTGAGAAATAGTGGCATATGCCAGGGAAATGTTCTTGGACAGT

ATTATGGTATTGGATCTGAAACAAGAGGAACAAATACGGATATTCCAGAT

CCTTTTGGGTTAGAAATAGGCAATCCTGAAGAAGGGTATGAGGAGAAATG

GATGCTTAATATCCACGCCATCGATACGCGAGGCGTGGAGGATAAGTTGG

GGTGCACTGAGTGTAGGTGTGACCTTTATAATGTTACAGTCAATGAATTT

GGCAAGCCTTTGCCTCCAGATTACATAGGAGGTTTGAATTGTTGCTATCA

TGAGACTCAGTGCAGGTTGAAGAAAGGCTTTCAAGGTCCCAAGAGAAGCC

TCTATTTGAGATACACAGTGAAGTGGATTGATTGGGATGAATATGTTGTT

CCTGTTAAGATTTATATAATTGATGTCACTGATACTTTGAAAATTTCAGA

TACTTCAAGTATAACAAGCTCAGATCATGATTGCCGGGTTGAGTATGAAG

TAGATCCTTGCAACAGAGACACCAAGAAAGGTAATGATTGTCTTGATGTG

AAGAGAACAAGCCTCCCATTTCCAAAGGGTGGCTATGTTGTGTATGGTGT

AGCTCATCAACATTCAGCTGGAATTGGAGCAACTCTATATGGACAGGATG

GAAGGGTAATATGTACCTCAATGGCAAAATATGGAACTGGAGATGAAGCA

GGAAATGAGGCAGGTTACATTGTAGGAATGACCACTTGTTATCCTAAACC

AGGTTCTGTAAAGATCATTGATGGCGAAAAAGTAACCCTGGAGTCTAACT

ACAGCAGCAGCAGTCGAAGTCACACTGGAGTCATGGGGCTTTTCTACCTT

CTGGTTGCAGAACAGCTTCCTCATCAACATTACTTCACTCATTCCTCTTC

ATTCTTCAGGAATATCAACAATGTAATTAATTAATAGGAGTGGTGTGTCA

TGTAATATAATATAAATATGTGAAAAGTAAAACAAATAATTCAGGACATT

AGGGTGAAGAATATATATACGTGTGTTGTGTTGAAAAGAAGCCTTGCCTT

CATGGCCTGTTGTATATTTGTAAACAAAATGTTTTTGGTCAATCGGCGTT

GATTAAAATAATATAATTTCTGTGGGGTTGCGCACTCTATTTCTGCCTGA

TCTCCTTGTCTTCTCCGCTTCCGTGATTGAACAAAAGATCTTTGAAAATA

ACAAAATAAAAATTGTATATTCTGTTTGTGCTGATATTTTGTTTTCTTCG

TTTAAAAGAAATCTAGTCATGAATTGATGATTAATTAG

**32.AhNSur8**

TATGTAACTATTTCTCTTCTTGTCTAATTTTTTTATCCATTTGGGTGACA

TTTGTGATTTCAGACGTGAGGATGTTTCTCTCCTCAAATATTTTGCCTAT

AAATCCTGGTTGCTGGTTCCGCATTCAATTACACTCTGCCTTGTGGTGTG

GTGTGGCACTCTTGAATTGAATGTCAACCAACAAAATGTGTGAGATGATT

TCATTGGCAATACTATTGCTGGTGTTAGGCAGTCCATGCTCAGCTGCTTT

TTCTTGGAAGACTGAGAATAAAATTAAGACAGCAGTTCATCTTTCCCCCA

AGATTGAAATAGGACCGGGATCGGTTTCGAATAAAAATTACTATGATATT

GATTTTCCAAGAGGTCATGTTGCTCTCAAGGGTTTCACTGCTGAAGTTGT

TGATGAAGCTGGAAACTCTGTACCCCTCCATGAAACTTACCTCCACCACT

GGGCTCTTATAAGATACCGTCAATCCAAGTCCAAACTTGCAACACACGCA

AGCTATGATCCTCATCGTGTGCTTCATGTGTCAGACTCAATCTCAGAATC

AGGGGTTGTGAGAAATAGTGGCATATGCCAGGGAAATGTTCTTGGACAGT

ATTATGGAATTGGATCTGAAACAAGAGGAACAAATACGGATATTCCAGAT

CCTTTTGGGTTAGAAATAGGCAATCCTGAAGAAGGGTATGAGGAGAAATG

GATGCTTAATATCCACGCCATCGATACGCGAGGCGTAGAGGATAAGTTGG

GGTGCACTGAGTGTAGGTGTGACCTTTATAATGTTACAGTCAATGAATTT

GGCAAGCCTTTGCCTCCAGATTACATAGGGGGTTTGAATTGTTGCTATCA

TGAGACTCAGTGCAGGTTGAAGAAAGGCTTTCAAGCTCCCAAGAGAAGCC

TCTATTTGAGATACACAGTGAAGTGGATGGATTGGGATGAATATGTTGTT

CCTGTTAAGATTTATATAATTGATGTGACTGATACTTTGAAAATTTCAGA

TACTTCAAGTATAACAAGCTCAGATCATGATTGCCGGGTTGAGTATGAAG

TAGATCCTTGCAACACAGACACCAAGAAAGGTAATGATTGTCTTGATGTG

AAGAGAACAAGGCTCCCATTTCCAAAGGGTGGTTATGTTGTGTATGGTGT

AGCTCATCAGCATTCAGCTGGAATTGGAGCAACTCTATATGGACAGGATG

GAAGGGTAATATGTACCTCAATGGCAAATTATGGAACTGGAGATGAAGCA

GGAAATGAGGCAGGCTACATTGTAGGAATGACCACTTGTTATCCTAAACC

AGGTTCTGTAAAGATCATTGATGGCGAAAAATTAACCCTGGAGTCAAACT

ACAGCAGCAGCACTCGAAGTCACACTGGAGTCATGGGGCTTTTCTACCTA

CTGGTTGCAGAACAGCTTCCTCATCAACACTACTTCACCCATTCCTCTTC

TTTCTTCAGGAATAGGAATATCAACAATGTATTTAACTAATAGGAGTGGT

GTGTCATGTAATATAATATAAATATGTGAAAAGTAAAACAAATAATTCAG

GCCATTAGGGTGAAGAATATATACGTGTGTTTTGTTGAAAGGAGGATGCT

CTCGTAAAGAGGCATAAAATGTCTTTTTGTAAAGACATTTATATATTTAT

TGTACGTATTAATGAATCGTTTATTTTTGAATTTCTTAATAAACTAGAAT

AAAATCGATTTTTTTATAAGAATAACAATAAATTAAATTATTATTAAA

**33.AhNSur9**

CTTTCATATTAATAACTAATCATTATATTCTTTTGGAAGGGGAAACTATT

TGAGTTACAGATGAAGATGTGGAATGTAAATCATTGTTGTTGGGCAATGG

TAGTGATGTTATCAAGCACAGTAGTGCCATATTCATGGGCCTTTGTGAAT

GAAAAAACTTCTGTATTTTTATCTCCCAAGATTGAGATAGGAGCAGGGAA

AAGTTCCAACAAATTTTACTACGATGTTGATTTCCCAAGAGGCCATATTG

CACTAAAGAGTTTCAATGCTGAAATAGTTGATGAAAAAGGAAACTCTGTG

CCTCTCTATGAGGCCTATCTTCACCATTGGATTGTTATGAAGTACCATCA

ACCCAAAAATGCCACAAAAACAAACCCAGGTATTGAAATTGTGCAAAACA

GTGGCTTATGCCAATACAATACTCTTCCATATTACTTTGGAGTTGGATCA

GAAACAAGAGGAGTAAAAACACACATTCCAGACCCTTATGCAATAGAAGC

TGGAAACCCTCCAGAAGGGTATGATGAGAAATGGGTAATAAATGTTCATG

CAATTGACACAAGGGGTGTCCAAGACAAGATTGGGTGCATTGAGTGCAGG

TGTGATCTCTTCAACATAACAAAAGATTCAGATGGAAAACCTTTGAGTCC

AAGTTACCATGGAGGGTTGACTTGTTGCCCTGATGAGAGTCAATGCTTGT

TGAAGAAAGGATTCAAACCTCAAAACAGAACCTTGTATCTTAAATACACT

GTCAAGTGGGTCACCTGGGAACACTTTATATTGCCTCTTAGGGTTTATGT

GCTTGATGTTACTGATGTTGTCAAAAATAACACACATAATTGCCTGGTTG

AGTATGATGTTTTGCCTTGCAAGTATGGAGGAGGTAAATGCGTTGATGTG

AGGAGAACAAAGCTTCCAATGAACAAAGGAGGTTATGTCATTTATGGTGT

TGCTCATGAACATGTTGGTGGCATTGGTTCAACTCTCTACGGCCAGGATG

GAAAAGTTATATGCAATTCACTACCAAAATATGGAAATGGAAGTGAAGCA

GGAAATGAGAAAGGGTACCTGGTGGGAATGACAACTTGCTACCCTCAACC

TGGCTCTATCAAGATCTCTAATGGTGAAGTTTTGACTTTGGAGGTTGACT

ACAGCAACACTAAGCTGCATAGTGGTGTTATGGGACTTTTCTACCTCCTT

GTTGCTGATGACCTCCCACATCACAAGAACTAA

**34.AhNSur10**

CTTTCATAGTAATAACTAATCATTATATTCTTTTGGAAAGGGAAACTATT

TGAGATCTAGATCAAGATGTGGAATGTAAATCATTGTTGTTGGGCAATGG

TAGTGATGTTATCAAGCACATATTCATGGGCCTTTGTGACTGAAAAAACA

GCTGTTTTTTTATCTCCCAAGATTGAGATAGGAGCAGGGAAAAGTTCAAA

CAAATTATACTACGATGTTGATTTCCCTAGAGGCCATATTGCACTCAAGA

GTTTCAATGCTGAAATAGTTGATGAGAAAGGAAACTCTGTGCCTCTCTAT

GAGGCCTATCTTCACCATTGGATTGTTATGAAGTACCATCAACCCAAAAA

TGCCACAAAAACAAACCCAGGTATTGAAATTGTGCAAAACAGTGGGTTAT

GCCAATACAATACTCTTCCATATTACTTTGGAGTTGGATCAGAAACAAGA

GGAGTAGAAACACACATTCCAGACCCTTATGGAATAGAAGCTGGAAACCC

TCCAAAAGGGTATGATGAGAAATGGGTAATAAATGTTCATGCAATTGACA

CAAGGGGTGTCCAAGACAAGATAGGCTGCATTGAGTGCAGGTGTGATCTC

TTCAACATCACAAAGGATTCAGATGGAAAACCTTTGAGTCCAAGTTACCA

TGGAGGCTTGACTTGTTGCCCTGATGAGAGTCAATGCTTGTTGAAGAAAG

GATTCAAGCCTCAAAACAGAACCTTGTATCTTAAATACACTGTCAAGTGG

GTCACCTGGGAACACTATATATTGCCTCTTAGGGTTTATGTGCTTGATGT

TACTGATGTTGTCAAAAATAACACACATAATTGCCTGGTTGAATATGATG

TTTTGCCTTGCAAGTATGGAGGTAAATGCGTTGATGTGAGGAGGACAAAG

CTTCCAATGAACAAAGGAGGTTATGTCATTTATGGTGTTGCTCATGAACA

TGTTGGTGGCACTGGTTCAACTCTCTACGGCCAGGATGGAAGAGTTATAT

GCAATTCACTACCAAAATATGGAAATGGAAGTGAAGCAGGAAATGAGAAA

GGGTACCTGGTGGGAATGACAACTTGCTACCCTCGACCTGGCTCTATCAA

GATCTCTAATGGTGAAGTTTTGACTTTGGAGGTTGACTACAGCAACACTA

AGCTGCATAGTGGTGTAATGGGACTTTTCTACCTCCTTGTTGCTGATGAC

CTCCCACATCACAAGAACTAA

**35.AhNSur11**

AAATCTGTAAATGTCACTATTATTAGAGTATCTAGCTCCATGCCTCCATG

TTTACCACCCATCCATGCTAGACGTCATTAAATTAAGCTAAGAGAAGCCA

ACCACTCCCATCTTGCGCCACGTGCAACAGTTCCACACACAATTAATTCT

GTTCGATTACATCAGAATTTTCCAAATGATTTTGCTTTTTGGACTTAGTA

GATTTAGCTTCATTCTTAGAAGGAATCTTGTTCACTATCCATACCATGCT

CGTTTCAGTTTCACATATGATATGAGGACGTCTCACTCATCAAATTTGCA

TGGCCTATAAAAATCTCTCTGGATTGTCAAAATCTAAACGTTGCCGTCTT

GTGTTGCGCCTCTCTTTAAGGTTAACTATCAACATGTCTTATGCATCTAA

AGATATGGTGATTTCATTGGCAATACTATTGCTTATGTTAGGCACACCAT

GCTCAAGTGCTTTTTGGAAGACTCAGAATAAGATTAAGACAGCTGTTCAT

CTTTCTCCAAAGATTGAACTTGGGCCAGGGTCAGTTTCGAATAAATTTTA

CTATGATATTGAGTTTCCAAGAGGTCATGTTGCGCTTAAGAGTTTCAATG

CTGAAGTAGTTGATGAAGCTGGAAACCCTGTACCTCTCCATAAAACTTAT

CTCCACCATTGGATTATTGTTGGATACCATGAATCCAAATCAAAACTTGC

GACACACACAAAATATGATCTTCATCGTGTGGTTCGTGTGTCAGACTCAG

TCTCAAAGTCACATATTATACTAAGAAATAGTGGCGTATGTCAGGGAAAT

ATTCTTGGACAGTATTTTGGACTTGGATCCGAAACACGAGGAACGGCTAC

GGATATTCCAGATCCTTTTGGGATAGAAATAGGAAATCCTGCAGAAATTC

CAGAAGGATATGAGGAGAAATGGTTGCTCAATGTCCACGCCATCGATACA

CGAGGTGTAGAGGATAAGCTAGGCTGCACTGAGTGTAAGTGTCACCTTTA

TAATGTTACAGTCAATGAATACGGCAACCCTTTGCCTCCAGATTACGCAG

GGGGTTTGTACTGTTGCTATGATGAGACTCAGTGCAGGTTGAAGAAAGGC

TTTCAAGGTCCAAAGAGAAGCCTCTATCTGAGATACACTGTGAAATGGAT

CGATTGGGACGAATATGTTGTTCCTGTTAAGATTTATATAATTGATGTGA

CTGATACTTTGAAAATATCAGATACTTCAAATATAGCAAGCTCAAATCAT

GATTGCCGGATTGAGTATGAAGTTGATCCTTGCAACATAGACCCCAAGAA

GAAAGGTAATGGTTGTGTTGATGTGAAGAGGACAACCGTCCCATTGGAAA

AGGGTGGTTATGTGGTCTATGCTGTAGCTCATCAGCATTCAGGTGGAATC

GGATCAACTCTATATGGACAGGATGGAAGGGTAATATGTACCTCAATGGC

AAATTATGGAACTGGGGATAATGCAGGAAATGAGTCAGGTTACATTGTAG

GAATGACCACTTGTTATCCTAAACCAGGTTCTGTAAAGATCATTGATGGC

GAAAAATTAACTCTGGAGTCTAACTACAGCAGCACCACGCGAGGTCACAC

CGGAGTCATGGGGCTTTTCTACCTACTGGTTGCAGAACAGCTTCCTCATC

AACATTAGTTCACTCATTCCTCTCCTTTCTTCAGGAATATAAACAATGTA

TTCAACTCATTTTGGCAATAATGTTAATAG

**36.AhNSur12**

ATGGCGAGGCGACAAGGAAATCTCACCATGTTCGGGCTTTCTATCTCTCC

TAAACTCTGCGCGTCTCTCTCTCTTCCCTTCGTCGGCGACGCGACGGGCG

CGATGGAAAACATGAAGTTTATACCTGAAGTGTTATTACTATCATTAACA

ATTATACTGTTGCAATCAAGCATCATATTTGCACGACAATATGAGAATTC

AAATCATATCAAGACGGCTACTTTTTATAGTGAGCAATTTGTATTGGAAC

CAGGAAAGGTTACGATAACAGATTTATTCGATATTGAGTTTCCAAGAGGA

CACATCGGAATCAAGAATTTTCAAGCCGAGTTAGTTGATGAACATCGGAA

TTCTTTGCCATTATATGAAGCTTACCTGCACCATTATTTTGTTTTAAGAT

ATTTTGAAAATGTCACCATGTCACGTCAGGCTAATCAAAGTCAGCCCATA

TACGGTAAGTATTTTAGAAGAAACGATGGAGTATGCCAGGGTAGTGTTAA

TTCATATTCCTGGGGGCTTGGAGTTGATGCACGAAAAACTAGCTTAGAAC

TACCAGATCCATTTAGAATAGAAGTAGGTACGCACCCTGAGAATGTCCCA

AAGGAGTATAATGAAGAGAAATGGTTATTCGATATCATGGTTATTGACAC

ACGTGGTACAGAAGACAAAAACGGTTGCACCGAATGCAGATGTGACCATT

ATAATGTCAAAAGTGAAGACTTTGTAAGCAAAACCGGTATTGATGGAAAA

CCAATGTCTGGTGATTATAAAGGAGGAATTTTTTGTTGTGAAAAGACTTC

TCAATGCAAATTACAAAAAGGATATAATAACAAACAACCGAGAAAAGCTT

CCCTTAAATATACAGTAACATGGGTAGATTGGGATCAATACCAAGTGCCT

ATTAAGTTTTACATACTTGATGTTACAGATCAAGTGACATATAATGGCTC

CGAACCAATTCATAATTGTATGGTAGAGTATTCTATCACTCCACAAAATA

CTGACATTGGGCATTATCATATTAAAAAGACAAAGATCCCAATGAAAAAG

GGTGGTAATCTAATCTACTCTATAGTTCATGTGCATCCAGGAATCGTTAA

TGCAACTTTATATGGGGAGAATGGAAAGGTATTATGTGCGGTTCAACCAA

CATACGGCACAGGAGAAGAGCCAGGAAATGAAAAAGGCTATGTTGTTGGA

ATGTCTGGTTGTTATCCAAAACCAGGCTCTATCAAGATTCAGGATGGCGA

AATATTAACGTTAAATAAAATATAA

**37.AhNSur13**

AAAAACATGAAGTTTATACCTGAAGTGTTATTACTATCATTAACAATTAT

ACTGTTGCAATCAAGCATCATATTCGCACGACAATATGAGAATTCAAATC

ATATCAAGACGGCTACTTTTTATAGTGAGCAATTTGTATTGGAACCAGGA

AAGGTTACGATAACAGATTTATTCGATATTGAGTTTCCAAGAGGACACAT

CGGAATCAAGAATTTTCAAGCCGAGTTAGTTGATGAACATCGGAATTCTT

TGCCATTATATGAAGCTTACCTACACCATTATTTTGTTTTAAGATATTTT

GAAAATGTCACCATGTCACGTCAAGCTAATCAAAGTCAGCCCATATACGG

TAAGTATTTTAGAAGAAACGATGGAGTATGCCAGGGTAGTGTTAATTCAT

ATTCCTGGGGGCTTGGAGTTGATGCACGAAAAACTAGCTTAGAACTACCA

GATCCATTTAGAATAGAAGTAGGTACGCACCCTGAGAATGTCCCAAAGGA

GTATAATGAAGAGAAATGGTTATTCAATATCATGGTTATTGACACACGTG

GTACAGAAGACAAAAAAGGTTGCACCGAATGCAGATGTGACCATTATAAT

GTCAAAAGTGAAGACTTTGTAAGCAAAACCGGTATTGATGGGAAACCAAT

GTCTAGTGATTATAAAGGAGGAATTTTTTGTTGTGAAAAGACTTCTCAAT

GCAAATTACAAAAAGGATATAATAACAGACAAGAGAGAAAAGCTTCCCTT

AAATATACAATAACATGGGTTGATTGGGATCAATACCAAGTACCTATTAA

GTTTTACATACTTGATGTTACTGATCAAGTGACATATAATGGCTCCGAAC

CAATTCATAATTGTATGGTAGAGTATTCTATCACTCCGCAAAATACTGAT

ATTGGACATTATCATATTAAAAGGACAAAGATCCCAATGAAAAAGGGTGG

TAATCTAATCTACTCTACAGCTCACGTGCATCCAGGAATCGTTAATGCAA

CTTTATATGGGGAGAATGGAAAGGTATTATGTGCGGTTCAACCAACATAC

GGCACAGGAGAAGAGCCAGGAAATGAAAAAGGCTATGTTGTTGGAATGTC

TGGTTGTTATCCAAAACCAGGCTCTATCAAGATTCAGGATGGCGAAATAT

TAACTGTT

**38.AhNSur14**

ATGGCACACATAGAAAACATGAAGTTTATACCTGAAGTGTTATTACTATC

ATTAACAATTATATTGTTGCAATCAAGCATCATATTCGCACGACAATATG

AGAATTCAAATCATATCAAGACGGCTACTTTTTATAGTGAGCAATTTGTA

TTGGAACCAGGAAAGGTTACGATAACAGATTTATTCGATATTGAGTTTCC

AAGAGGACACATCGGAATCAAGAATTTTCAAGCCGAGTTAGTTGATGAAC

ATCGAAATTCTTTGCCATTATATGAAGCTTACCTGCACCATTATTTCGTT

TTAAGATATTTTGAAAATGTCACCATGTCACGTCAAGCTAATCAAAGTCA

GCCCATATACGGTAAGTATTTTAGAAGAAACGATGGAGTATGCCAAGGTA

GTGTTAATTCATATTCCTGGGGGCTTGGAGTTGATGCACGAAAAACTAGC

TTAGAACTACCAGATCCATTTAGAATAGAAGTAGGTACGCACCCTGAGAA

TGTCCCAAAGGAGTATAATGAAGAGAAATGGTTATTCAATATCATGGTTA

TTGACACACGTGGTACAGAAGACAAAAAAGGTTGCACCGAATGCAGATGT

GACCATTATAATGTCAAAAGTGAAGACTTTGTAAGCAAAACTGGTATTGA

TGGGAAACCAATGTCTAGTGATTATAAAGGAGGAATTTTTTGTTGTGAAA

AGACTTCTCAATGCAAATTACAACAAGGATATAATAACAGACAACAGAGA

AAAGCTTCCCTTAAATATACAGTAACATGGGTTGATTGGGATCAATACCA

AGTGCCTATTAAATTTTACATACTTGATGTTACTGATCAAGTGACATATA

ATGGCTCCGAACCAATTCATAATTGTATGGTAGAGTATTCTATCACTCCG

CAAAATACTGATATTGGACATTATCATATTAAAAGGACAAAGATCCCAAT

GAAAAAGGGTGGTAATCTAATTTACTCTACAGCTCACGTGCATCCAGGAA

TCATTAATGCAACTTTATATGGGGAGAATGGAAAGGTATTATGTGCGGTT

CAACCAACATACGGCACAGGAGAAGAGCCAGGAAATGAAAAAGGCTATGT

TGTTGGAATGTCTGGTTGTTATCCAAAACCAGGCTCTATCAAGATTCAGG

ATGACGAAATATTAACTGCT

**39.AhNSur15**

GTCAACATGAAGTTTATTTGCGAAGTGGTGATATTATCATTTTCAATTAT

AGTAATACAATCGAGCATCACATTCTCACGAGAACTTGAAGGTCCAAATC

ATATCAAGACAACTACTTTTTATACCAAAACGTTCGTACTAGAACCAGGA

AAGGTTAGTAGAAAAACTTTTTTCGATGTTGAGTTTCCAAGAGGCCACAT

TGGAATCAAGAATTTACAAGCCGAACTAGTTGATGAACACGGAAACTCTA

TACCACTATATGAGGCTTACCTGCACCATTATTTTGTTTTAAGATATTTT

GAAAATATCACCATGTCACAACATGCTAATGAAAGTCAACCTAATTACGG

TAAGTATTTTAAGAGAAATGATGGTGCATGTCAAACTTTCGTTAATTCAA

TTTCTTGGGGTCTTGGAGTTGACGCACGAAGAACTAACACAGAACTACCA

GATCCATTTAGAGTAGAAGTAGGTACGCATCCTGAGGATGTTCCAAAGGA

GTATGATGAAGAGAAATGGTTAATCAATATTTTGGTCATTGACACACGTG

GTGCAGAAGACAAGAAAGGTTGCTCCCAATGCAGATGTGACCTTTTAAAC

GTCAAAAGTGAAGATTTGAGAAACACAACAGGCGTTGATGGAACACCATT

GTCTAGTGATTACAAAGGAGGAATCTTTTGTTGCGAGAAGAAGTCTCAAT

GCAAATTACAAAAGGGATACAATGAAAAACAAAAGAGAAAAGTTGCTATT

AAATATACAATATCATGGGTTGAATGGGATCAACAGCAAGTGCCTCTTAA

GTTTTATATTCTTGATGTTACTGATCAAGTCACATATAATGGATCCGAAC

CAATTCATCATTGCGCGGTAGAGTATTCTATAAATCCAGAAAAGACTGAT

GAAGGACATTACCATATTAAGAAAACAAATATTCCAATGAAAAAAGGTGG

TAGTCTCATCTATATTACTGCTCATGTACATTCAGGAATTGTTAATGCAA

CATTATATGGAGAGGATGGAAGAAGATTATGTGAAATTAAGCCAATATAT

GGAACGGGAAAAGAGGCAGGCAATGAAGAAGGTTATGCTGTTGGAGCGTC

TGGTTGCTATCCAAAACCGGGCTCTATGAAGATTAAAGATGGTGAAAATT

TAACTGCAGAATTTATACATGAAAACAAATATACCACTGGACTTATGGGG

CATTTCTATGTCTATTTGGCAGAAGACTTACCAAAATCTTTCTAA

**40.AhNSur16**

GTCAACATGAAGTTTATTTGCGAAGTGGTGATATTATCATTTTCAATTAT

AGTAATACAATCGAGCATCACATTCTCACGAGAACTTGAAGGTCCAAATC

ATATCAAGACAACTACTTTTTATACCAAAACGTTCGTACTAGAACCAGGA

AAGGTTAGCAGAAAAACTTTTTTCGATGTTGAGTTTCCAAGAGGCCACAT

TGGAATCAAGAATTTACAAGCCGAACTAGTTGATGAACACGGAAACTCGA

TACCACTGTATGAGGCTTACCTGCACCATTATTTTGTTTTAAGATATTTT

GAAAATATCACCATGTCACAACATGCTAATGAAAATCAACCTAATTACGG

TAAGTATTTTAAGAGAAATGATGGTGCATGTCAAACTTTCGTTAATTCAA

TTTCTTGGGGTCTTGGAGTTGACGCACGAAGAACTAGCACAGAACTACCA

GATCCATTTAGAGTAGAAGTAGGTACGCATCCTGAGGATGTTCCAAAGGA

GTATGATGAAGAGAAATGGTTAATCAATATTTTGGTCATTGACACACGTG

GTGCAGAAGACAAGAAAGGTTGCTCCCAATGCAGATGTGACCTTTTAAAC

GTCAAAAGTGAAGATTTGAGAAACACAACAGGCGTTGATGGAACACCATT

ATCTAGTGATTATAAAGGAGGAATTTTTTGTTGCGAGAAGAAGTCTCAAT

GCAAATTACAACAGGGATACAATGAAAAACAAAAGAGAAAAGTTGCTATT

AAATATACAATATCATGGGTTGAATGGGATCAACAGCAAGTGCCTCTTAA

GTTTTATATTCTTGATGTTACTGATCAAGTCACATATAATGGATCCGAAC

CAATTCATCATTGCGCGGTAGAGTATTCTATAAATCCAGAAAAGACTGAT

GAAGGACATTACCATATTAAGAAAACAAATATTCCAATGAAAAAAGGTGG

TAGTCTCATCTATATTACTGCTCATGTACATTCAGGAATTGTTAATGCAA

CATTATATGGAGAGGATGGAAGAAGATTATGTGAAATTAAGCCAATATAT

GGAACGGGAAAAGAGGCAGGCAATGAAGAAGGTTATGCTGTTGGAGCGTC

TGGTTGCTATCCAAAACCGGATTCTATGAAGATTAAAGATGGTGAAAATT

TAACTGCAGAATTTATACATGAAAACAAATATACCACTGGACTTATGGGG

CATTTCTATGTCTATTTGGCAGAAGACTTACCAAAATCTTTGTAA

**41.AhNSur17**

GTCAACATGAAGTTTATTTGCGAAGTGGTGATATTATCATTTTCAATTAT

AGTAATACAATCGAGCATCACATTCTCACGAGAACTTGAAGGTCCAAATC

ATATCAAGACAACTACTTTTTATACCAAAACGTTCGTACTAGAACCAGGA

AAGGTTAGCAGAAAAACTTTTTTCGATGTTGAGTTTCCAAGAGGCCACAT

TGGAATCAAGAATTTACAAGCCGAACTAGTTGATGAACACGGAAACTCGA

TACCACTGTATGAGGCTTACCTGCACCATTATTTTGTTTTAAGATATTTT

GAAAATATCACCATGTCACAACATGCTAATGAAAATCAACCTAATTACGG

TAAGTATTTTAAGAGAAATGATGGTGCATGTCAAACTTTCGTTAATTCAA

TTTCTTGGGGTCTTGGAGTTGACGCACGAAGAACTAGCACAGAACTACCA

GATCCATTTAGAGTAGAAGTAGGTACGCATCCTGAGGATGTTCCAAAGGA

GTATGATGAAGAGAAATGGTTAATCAATATTTTGGTCATTGACACACGTG

GTGCAGAAGACAAGAAAGGTTGCTCCCAATGCAGATGTGACCTTTTAAAC

GTCAAAAGTGAAGATTTGAGAAACACAACAGGCGTTGATGGAACACCATT

ATCTAGTGATTATAAAGGAGGAATTTTTTGTTGCGAGAAGAAGTCTCAAT

GCAAATTACAACAGGGATACAATGAAAAACAAAAGAGAAAAGTTGCTATT

AAATATACAATATCATGGGTTGAATGGGATCAACAGCAAGTGCCTCTTAA

GTTTTATATTCTTGATGTTACTGATCAAGTCACATATAATGGATCCGAAC

CAATTCATCATTGCGCGGTAGAGTATTCTATAAATCCAGAAAAGACTGAT

GAAGGACATTACCATATTAAGAAAACAAATATTCCAATGAAAAAAGGTGG

TAGTCTCATCTATATTACTGCTCATGTACATTCAGGAATTGTTAATGCAA

CATTATATGGAGAGGATGGAAGAAGATTATGTGAAATTAAGCCAATATAT

GGAACGGGAAAAGAGGCAGGCAATGAAGAAGGTTATGCTGTTGGAGCGTC

TGGTTGCTATCCAAAACCGGATTCTATGAAGATTAAAGATGGTGAAAATT

TAACTGCAGAATTTATACATGAAAACAAATATACCACTGGACTTATGGGG

CATTTCTATGTCTATTTGGCAGAAGACTTACCAAAATCTTTGTAA

**42.AhNSur18**

GTCAACATGAAGTTTATTTGCGAAGTGGTGATATTATCATTTTCAATTAT

AGTAATACAATCGAGCATCACATTCTCACGAGAACTTGAAGGTCCAAATC

ATATCAAGACAACTACTTTTTATACCAAAACGTTCGTACTAGAACCAGGA

AAGGTTAGCAGAAAAACTTTTTTCGATGTTGAGTTTCCAAGAGGCCACAT

TGGAATCAAGAATTTACAAGCCGAACTAGTTGATGAACACGGAAACTCGA

TACCACTGTATGAGGCTTACCTGCACCATTATTTTGTTTTAAGATATTTT

GAAAATATCACCATGTCACAACATGCTAATGAAAATCAACCTAATTACGG

TAAGTATTTTAAGAGAAATGATGGTGCATGTCAAACTTTCGTTAATTCAA

TTTCTTGGGGTCTTGGAGTTGACGCACGAAGAACTAGCACAGAACTACCA

GATCCATTTAGAGTAGAAGTAGGTACGCATCCTGAGGATGTTCCAAAGGA

GTATGATGAAGAGAAATGGTTAATCAATATTTTGGTCATTGACACACGTG

GTGCAGAAGACAAGAAAGGTTGCTCCCAATGCAGATGTGACCTTTTAAAC

GTCAAAAGTGAAGATTTGAGAAACACAACAGGCGTTGATGGAACACCATT

ATCTAGTGATTATAAAGGAGGAATTTTTTGTTGCGAGAAGAAGTCTCAAT

GCAAATTACAACAGGGATACAATGAAAAACAAAAGAGAAAAGTTGCTATT

AAATATACAATATCATGGGTTGAATGGGATCAACAGCAAGTGCCTCTTAA

GTTTTATATTCTTGATGTTACTGATCAAGTCACATATAATGGATCCGAAC

CAATTCATCATTGCGCGGTAGAGTATTCTATAAATCCAGAAAAGACTGAT

GAAGGACATTACCATATTAAGAAAACAAATATTCCAATGAAAAAAGGTGG

TAGTCTCATCTATATTACTGCTCATGTACATTCAGGAATTGTTAATGCAA

CATTATATGGAGAGGATGGAAGAAGATTATGTGAAATTAAGCCAATATAT

GGAACGGGAAAAGAGGCAGGCAATGAAGAAGGTTATGCTGTTGGAGCGTC

TGGTTGCTATCCAAAACCGGATTCTATGAAGATTAAAGATGGTGAAAATT

TAACTGCAGAATTTATACATGAAAACAAATATACCACTGGACTTATGGGG

CATTTCTATGTCTATTTGGCAGAAGACTTACCAAAATCTTTGTAA
