# Supplementary material for: Genome wide identification and characterization of nodulation related genes in Arachis hypogaea
Source: PLoS One. 2022 Sep 9;17(9):e0273768. doi: 10.1371/journal.pone.0273768 (PMC9462762; doi:10.1371/journal.pone.0273768)
Supplement: S2 Text — (DOCX) [file pone.0273768.s002.docx]

**1.AhNMTL1**

MMEKSDNLVLLRQVVPYVLCLCIFIRSASASEGFESIACCADSNYKDPLTNLDYKTDYAWFSDTRSCRPITSVLKHSAYGRLRVFDIEKGKRCYNLATTKDQVYLIRGTFPSENEPGKGSFGVSIGVTVLGTVRSSSQDLRIEGVFRATKNNTDFCLVTEEGNPYISQLELRSVSEEYLQGLNSSVLKLINRSNLGGKEDDIRYPIDQSDRIWKRTTTSPYTPISFNISILDHKSNVTPPLKVLQTALTHPERLEFNHNGLEVKEDYEYLVFLYFLELNNSVREGLRVFDIYVNSEIKKASFDVLSEGSNYRHIVLNASSANGSLNLTLVKASGSVFGPSCNAYEIMQVRPWKQETNQTDLVAILKIREELITENHDNKVLQSWTGDPCMLFAWQGISCDFSNDAPVIIKLDLSLSNLKGPLPSSVTELTNLQIMNLSHNNFNGHIPSFPSSSMLTSLDLRYNDLMGSLPPSYNSLPHLKSIYYGCNDRMDHKLSGNLNISINTDNGSCQKGNDFPQQVAIISAVACGSFLIAMAVGTIFIYRYRQRLNPLEAFGRKSNPMITNVIFSLNSIDDFLIKSISIQAFTLEYIEVATEKYNILIGEGGFGSVYRGTLENGQVVAVKVRSATSTQGTREFDNELNLLSAIRHENLVPLLGYCNENDQQILVYPFMSNGSLQDRLYGEPAKRKILDWPTRLSIALGAARGLAYLHSFPERSVIHRDVKSSNILLDHSMCAKVADFGFSKYAPQEGDSAVSLEVRGTAGYLDPEYYTTQQLSAKSDVFSFGVVLLEIVSGREPLNIHRPRNEWSLVEWAKPYIRASKIEEIVDPGIKGGYHAEAMWRVLEVALQCVEPFSTNRPAMDDVMRELEDALIIENNASEYMKSIESLGSNRYSIVIEKRVPPSTSSMVESTIIPQSLNHPQPR

**2.AhNMTL2**

MMEKSDNLVLLRQVVPYVLCLCIFIRSASAIEGFESIACCADSNYKDPLTNLDYKTDYAWFSDTRSCRPITSVLKHSTYGRLRVFEIEKGKRCYKLATTKDQVYLIRGTFPSENAPGKGSFGVSIGVTVLGTVRSSSQDLRIEGVFRATKNNTDFCLVTEEGNPYISQLELRSVSEEYLQGLNSSVLKLINRSNLGGKEDDIRYPIDQSDRIWKRTTTSPYTPISFNISILDHKSNVTPPLKVLQTALTHPERLEFNNNGLEVKEDYEYLVFLYFLELNNSVREGQRVFDIYVNSEIKKASFDVLSEGSNYRHIVLNASSANGSLNLTLVKASGSVFGPSCNAYEIMQVRPWKQETNQTDLEVILKIREELITENHDNKVLQSWTGDPCMLFAWQGISCDFSNGAPVITKLDLSLSNLKGPLPSSVTELTNLQIMNLSHNNFNGYIPSFPSYSMLTSLDLRYNDLMGSLPPSYNSLPHLKSIYYGCNDRMDHKLSGNLNISINTDNGSCQKGNDFPQQVAIISAVACGSFLIAMAVGTIFIYRYRQRLNPLEAFGRKSNPMITNVIFSLNSIDDFLIKSISIQAFTLEYIEVATEKYNILIGEGGFGSVYRGTLENGQVVAVKVRSATSTQGTREFDNELNLLSAIRHENLVPLLGYCNENDQQILVYPFMSNGSLQDRLYGEPAKRKILDWPTRLSIALGAARGLAYLHSFPERSVIHRDVKSSNILLDHSMCAKVADFGFSKYAPQEGDSAVSLEVRGTAGYLDPEYYTTQQLSAKSDVFSFGVVLLEIVSGREPLNIHRPRNEWSLVEWAKPYIRASKIEEIVDPGIKGGYHAEAMWRVLEVALQCVEPFSTNRPAMDDVMRELEDALIIENNASEYMKSIESLGSNRYSIVIEKRVPPSTSSMVESTIIPQSLNHPQPR

**3.AhNKEF1**

MGNCNACARADVVDSTTTTITKPTTNNRKPNPFSSTPARPANPIRVLNNDIPAGPQHRARISDDYILGRELGRGEFGITYLCTDRETKEALACKSISKRKLRTAVDVDDVRREVKIMSTLPPHPNVVQLKGAYEDDENVHIVMELCEGGELFDRIVARGHYSERAAAGVFRTIAEVVRMCHANGVMHRDLKPENFLFANKKENSPLKAIDFGLSVFFKPGERFSEIVGSPYYMAPEVLKRNYGPEVDVWSAGVILYILLCGVPPFWAETEQGVALAILRGVIDFKREPWPHISDSAKSLVRQMLEPDPKKRLTAEQVLGGNWEGDCGKFWNEITLDFLAHPWLQNAKKAPNVPLGDLVRSRLKQFSMMNRFKKKALRVIADHLSVEEVEIIKDMFTLMDTDKDGRVTYEELKAGLRKVGSQLAEPEIKLLMEVADVDGNGILDYGEFVAVTIHLQKMENDEHFRKAFKYFDKDSSGYIEFGELQEALADESGETDHDVLNDIMREVDTDKDGRISFEEFVAMMKTGTDWRKASRQYSRERFKSLSLNLMKDGSLQLHDGISGQAVVV

**4.AhNKEF2**

MGNCNACARADVVDSTTTTTTKPNTNNRKSNPFSSTPARPANPIRVLNNDIPAGPQHRARISDDYILGRELGRGEFGITYLCTDRETKEALACKSISKRKLRTAVDIDDVRREVKIMSTLPPHPNVVQLKGAYEDDENVHIVMELCEGGELFDRIVARGHYSERAAAGVFRTIAEVVRMCHANGVMHRDLKPENFLFANKKENSPLKAIDFGLSVFFKPGERFSEIVGSPYYMAPEVLKRNYGPEIDVWSAGVILYILLCGVPPFWAETEQGVALAILRGVIDFKREPWPHISDSAKSLVRQMLEPDPKKRLTAEQVLGGNWEGDCGKFWNEITLDFLAHPWLQNAKKAPNVPLGDLVRSRLKQFSMMNRFKKKALRVIADHLSVEEVEIIKDMFTLMDTNKDGRVTYEELKAGLRKVGSQLAEPEIKLLMEVADVDGNGILDYGEFVAVTIHLQKMENDEHFRKAFKYFDKDSSGYIEFGELQEALADESGETDHDVLNDIMREVDTDKDGRISYEEFVAMMKTGTDWRKASRQYSRERFKSLSLNLMKDGSLQLHDGISGQAVVV

**5.AhNKEF3**

MGNTCRGSLRGKYFQGFSQPEDPSRRSNPSDPSDSEHLPKQNPNSADNNNSNNNNNNNNNINNNKRNLPFKKDTIMRRGPDNQAYYVLGHKTHNIRDLYTLGRKLGQGQFGTTYLCTENSTNIEYACKSISKRKLISKEDVEDVRREIQIMHHLAGHKNIVTIKGAYEDPLYVHIVMELCSGGELFDRIIQRGHYTERKAAELTKIIVGVVETCHSLGVMHRDLKPENFLLVNKDDDFSLKAIDFGLSVFFKPGQVFTDVVGSPYYVAPEVLLKHYGPEADVWTAGVILYILLSGVPPFWAETQQGIFDAVLKGHIDFDSDPWPLISDSAKDLIRKMLCSRPSERLTAHEVLCHPWICENGVAPDRALDPAVLSRLKQFSAMNKLKKMALRVIAESLSEEEIAGLREMFQAMDTDNSGAITFDELKAGLRRYGSTLKDTEIRDLMEAADVDNSGTIDYGEFIAATIHLNKLEREEHLIAAFRYFDKDGSGYITVDELQQACIEHNMTDVFLEDIIREVDQDNDGRIDYGEFAAMMQGNAGIGRRTMRNSLNLSMRDAPGV

**6.AhNKEF4**

MGNTCRGSLRGKYFQGFSQPEDPSRRSNPSDPSDSEHLPKQNPNSADNNNNSNNNNNININNNKRNLPFKKDTIMRRGPDNQAYYVLGHKTHNIRDLYTLGRKLGQGQFGTTYLCTENSTNIEYACKSISKRKLISKEDVEDVRREIQIMHHLAGHKNIVTIKGAYEDPLYVHIVMELCSGGELFDRIIQRGHYTERKAAELTKIIVGVVETCHSLGVMHRDLKPENFLLVNKDDDFSLKAIDFGLSVFFKPGQVFTDVVGSPYYVAPEVLLKHYGPEADVWTAGVILYILLSGVPPFWAETQQGIFDAVLKGHIDFDSDPWPLISDSAKDLIRKMLCSRPSERLTAHEVLCHPWICENGVAPDRALDPAVLSRLKQFSAMNKLKKMALRVIAESLSEEEIAGLREMFQAMDTDNSGAITFDELKAGLRRYGSTLKDTEIRDLMEAADVDNSGTIDYGEFIAATIHLNKLEREEHLIAAFRYFDKDGSGYITVDELQQACIEHNMTDVFLEDIIREVDQDNDGRIDYGEFAAMMQGNAGIGRRTMRNSLNLSMRDAPGV

**7.AhNNLC1**

MSGFSFGSSSSSQSSSSSPFSLTNPPSSSASSSAFSFGSSTPSTGFSFGSSSLFSSTTATANPSSAASSSPSPFSFSFASSSSTAGGSGGATTTAPSFGFGSTPSSSAASAPSFSFGFGSAPTASGSAPAPAPSLFGSASSASTAAASSSGSSIFGAASSGSSLFSTPSFGGTSSATTPFGAKPSAATTPFAGASSASSASPFGGASSAPPSIFGGASSASTTLFGGTSSATTSFGSTPSSTTAAASKPFGGFSLSPSAASSSAATTTPSFSSVFATGASSSSSSSSSLFTGFAKPSAPTPTTTAASTAAASAPTPTSTTGFSFGNATSSASQPSFGFPNAAVSSPASSASTASSTPASKPPGSFSFTTASAPLFSTVTATTASTPAAAASGSTPSSSVPAFGIPASTAPAIAASSLSGTPAASAGAASSTSGGSSFAGFGVGSSASTGSSTASFGTGFSFATKASAASTPAVSSSALAFGVSSTTTTAPTISSSSASATQTSSALVVASTSGTTSTVSTSVAAAAAPKLPSEITGKTVEEIIKEWNTELQERTGKFRKQANAIAEWDRRILQNRDVLLRLEIEVAKVVETQSNMERQLELIETHQQEVDKALQSMEEEAERIYKDERGLLLDDEAASTRDAMYEQSELIERELEQMTEQIKSIIQSLNSNQGGELDALDGMTPLDAVVRILNNQLTSLMWIDEKAEEFSSRIQKLANQGSASDRELMGPRMWMS

**8.AhNNLC2**

MSGFSFGSSSSSQSSSSSPFSLTNPPSSSASSSAFSFGSSTPSTGFSFGSSSLFSSTTATATANPSSVASSSPSPFSFSFASSSSTAGGSGGATTTAPSFGFGSTPSSSAASAPSFSFGFGSAPTASGSAPAPAPSLFGSASSASTAAASSSGSSIFGAASSGSSLFSTPSFGGTSSATTPFGAKPSAATTPFGGASSASSASPFGGASSATPSLFGGASSASTTLFGGTSSATTSFGSTPSSTTAAASKPFGGFSLSPSAASSSAATTTPSFSSVFATGASSSSSSSSSLFTGFAKPSAPTPTTTAASTAAASAPTPTSTTGFSFGNATSSASQPSFGFPNAAVSSPASSASTASSTPASKPPGSFSFTTASAPLFSTVTATTASAPAAAASGSTPSSSVPAFGIPASTAPAIAASSLSGTPAASAGAASSTSGGSSFAGFGVGSSASTGSSTASFGTGFSFATKASAASTAAVSSSALAFGVSSTTTTAPTISSSSASATQTSSALVASTSGTTSTVSTSVAAAAAPKLPSEITGKTVEEIIKEWNTELQERTGKFRKQANAIAEWDRRILQNRDVLLRLEIEVAKVVETQSNMERQLELIETHQQEVDKALQSMEEEAERIYKDERGLLLDDEAASTRDAMYEQSELIERELEQMTEQIKSIIQSLNSNQGGELDALDGMTPLDAVVRILNNQLTSLMWIDEKAEEFSSRIQKLANQGSASDRELMGPRMWMS

**9.AhNPR1**

MRDGGVLSSGTMLEPTPPPPPPSPLPPPPPPPSEVATTTTTSILMDFDYINELFVDGCWLAASSSSAAAAADGSADFYVSSPSFSNPIFDPFSWPSLDTEQIESQEREQEHEQQQEQPPFSHDLVVAANNCSQNQQQQYHHYENQSVENNNPNPNNPSEFFRRWWIAPSSNPGPGSYVVEKLLKALMCIKDVNRNKDMLIQIWIPVINRGGTQILRTNGLPFSLESSSVNLAKYREISEVYQFSAEEDSKELVPGLPGRVYKEKVPEWTPDVRFFRSYEYPRVDHAQVYDVRGSLALPIFEQGSKNCLGVVEVVMTQQKINYRPELESVCQALEAVNLTSSKLPTIQNVKRTSCEKSYETALPEIQEVLRSACEIHKLPLAQTWIPCIKQGKEGCRHSEDNYPHCISPVEHACYVGDPSIQVFHEACSEHHLLKGQGVAGGAFMTNQPCFAPDITLLSKTDYPLSHHARMFGLRAAVAIRLRSIYNSSDDFVLEFFLPLECIDNDEQKKMLTSLSLIIQRVCHSLRVISDKEVEEETDFSAEVIAHEDSGTFASAAAWPEPLQSQIVASLGAQEKSSETMGTSFSDQRQQQQESSVLKGNLDSNGECSTYNVGNLSSKTGDKKKSKVDKTITLQVLRQHFAGSLKDAAKNIGVCTTTLKRICRQHGIKRWPSRKIKKVGHSLQKLQLVIDSVQGASGAFQIDSFYSKFPDLAASPNLSGTSLFSNLKQCDNNPNSLSIQPDPGSLSPEGASKSPSSSCSQSSISSHPCSSMAEQQNHHHTNNNFDSSKDQMVLLVGENSSGDGLLKRIRSEAELKSLNEDRAKVVMPRSQSQETLGQHNLKNGHHGSLSRTKSKGTQKEDAPYRVKVTYGDEKARFKMPKNWGYEDLVQEVGRRFCISDMNKFDLKYLDDDYEWVLLTCDDDLEECIEVCQSSESTTIKLCLQFSNTNHSMRNPLEFR

**10.AhNPR2**

MRDGGVLSSGTMLEPTPPPPPPPPPPPPPAPPPSEVATTTTSISMDYDYINELFVDGCWLAASSSSAAAAAADGSADFYVPSPSFSNPIFDPFSWPCLDTEQIESQEREREQEHEHEKQQQPPPFSHDLVVAANSCSRNQQQQYHHYENQSVENNNPNPNNPSEFFRRWWIAPSSNPGPGSYVVEKLLKALMCIKDVNRNKDMLIQIWIPVMNRGGTQILRTNGLPFSLESRSVNLAKYREISEVYQFSAEEDSKELVPGLPGRVYKEKVPEWTPDVRFFRSYEYPRVDHAQVYDVRGSLALPIFEQGSKNCLGVVEVVMTQQKINYRPELESVCQALEAVNLTSSKLPTIQNVKHTSCEKSYETALPEIQEVLRSACEIHKLPLAQTWIPCIKQGKEGCRHSEDNYPHCISPVEHACYVGDPSIQVFHEACSEHHLLKGQGVAGGAFMTNQPCFAPDITLLSKTDYPLSHHARMFGLRAAVAIRLRSIYNSSDDFVLEFFLPLECIDNDEQKKMLTSLSLIIQRVCHSLRVISDKELEEETDFSVEEVIAHEDSGTFASAAAWPEPLQSQIVASLGAQEKSSETMGTSFSDQRQQQQESSVLKGNLDSNGECSTYNVGNLSSKTGDKKKSKVDKTITLQVLRQHFAGSLKDAAKNIGVCTTTLKRICRQHGIKRWPSRKIKKVGHSLQKLQLVIDSVQGASGAFQIDSFYSKFPDLAASPNLSGTSLFSNLKQCDNNPNSLSIQPDPGSLSPEGASKSPSSSCSQSSISSHPCSSMAEQQNHHLTNNNFDSSKDQMVLLVGENSSGDGLLKRIRSEAELKSLNEDRVKIVMPRSQSQETLGQHNVQNGHHGSLSRTKSKGTQKEDAAYRVKVTYGDEKARFKMPKNWGYEDLVQEVGRRFCISDMSKFDVKYLDDDYEWVLLTCDDDLEECIEVCQSSESTTIKLCLQLSNTNHSMRNPLEFR

**11.AhNPR3**

MPDSCEEKSELPSKSKPQEEHGFPMDFDIYLESSSWPMDHTPSASNPMSPFIITTSSEQPFSPLWAFSDVEDDHRHVRVVAGDNTNTAIETENPVENDDNKKIVSPPFVPLPPIKIPDGYCLIKERMTQALRHFKQLTEQNFLAQVWAPVRNGNRYALTTSGQPFVLDPHSNGLHQYRTVSLMYMFPVDGENDEILGLPGRVFQQKLPEWTPNVQYYTSREYSRRNHAQHYNVRGTLALPVFESPGQSCVGVLELIMTSEKVNYAPEVDKVCKALEAVNLRSSEILEHPFAQICNEGRQNALAEILEILTVVCETHNLPLAQTWVPCRHRSVLANGGGLKKSCSSFDGHCMGRVCMSASDVAFYVIDAHTWGFHDACAEHHLQQGQGVAGRAFLSHNMSFCGNITQFCKTDYPLVHYALMFGLTSCFAICLQSSHTGSDDYVLEFFLPPSITNFYEQKDLLGSILATMKQNFQSLKVAAGVELEEGCTIEVVEPINERIHLSLESVPVAQSAKSPQPTLNASLNKDDGVPQGPLEQQMPAWLDDINDGGNLGDNAGGSTNLMTSLEAKIKKKPSERKRGKAEKMISLEVLQRYFSGSLKDAAKSLGVCPTTMKRICRQHGISRWPSRKINKVNRSLSKLKCVIESVQGAEGAFALNSVNKDPLPIAAGSFTEPCTSKMFNRNASLSIQPSKTQINENDLDTSRVSETNRQVRMQDQFLEGEQSPEKVIHEEGWSTQEVGTKDPEKFRNLSGSSEDSANPHSHDSCHGSPPNEISPANIFRPFNKEKSVPLRVSAESTMQPTNALNYANAYTALNVERTEPQEPFGRMLLEGVGSSKDLRNLCPLEDQALEACGVNPPCHDLAPKQCMMDITLNSNNTMIPFATKKEMKSVTIKATYKEDIIRFRVSLNCGIVELQEEISKRLKLEIGAFDIKYLDDDNEWVLISCDADLQECMDVLTSSGSNMIRLVVHDTVSILGSSCESSGN

**12.AhNPR4**

MPDSCEEKSELPSKSKPQEEHGFPMDFDIYLESSSWPMDHTPSASNPMSPFIITTSSEQPFSPLWAFSDVEDDHRHVRVVAGDNTNTNTAIETENPVENDDNKKIVSPHFVPLPPIKIPDGYCLIKERMTQALRHFKQLTEQNFLAQVWAPVRNGNRYALTTSGQPFVLDPHSNGLHQYRTVSLMYMFPVDGENDEILGLPGRVFQQKLPEWTPNVQYYTSREYSRRNHAQHYNVRGTLALPVFESPGQSCVGVLELIMTSEKVNYAPEVDKVCKALEAVNLRSSEILEHPFAQICNEGRQNALAEILEILTVVCETHNLPLAQTWVPCRHRSVLANGGGLKKSCSSFDGHCMGRVCMSASDVAFYVIDAHTWGFHDACAEHHLQQGQGVAGRAFLSHNMSFCGNITQFCKTDYPLVHYALMFGLTSCFAICLQSSHTGSDDYVLEFFLPPSVTNFYEQKDLLGSILATMKQNFQSLKVAAGVELEEGCTIEVVEPINERIHLSLESVPVAQSAKSPPPTLNASLNKDDGVPQGPLEQQMPAWLEDINDGGNLGDNAGGSMNPMTSLDAKIKKKPSERKRGKAEKMISLEVLQRYFSGSLKDAAKSLGVCPTTMKRICRQHGISRWPSRKINKVNRSLSKLKCVIESVRGAEGAFALNSVNKDPLPIAAGSFTEPCTSKMFNRNASLSIQPSKTQMNENDLDTSRVSETNRQVRMQDQLLEGEQSPEKVIHEEGWSTQEVGTKDPEKFRNLSGSSEDSANPHSHDSCHGSPPNEISPANIFRPFNKEKSVPLRVSAESTMQPTNALNYANAYTALNVERTEPQEPFGRMLLEGVGSSKDLRNLCPLEDQALEACGVNPPCHDLAPKQCMMDTTLNSNNTMIPFAPKKETKSVTIKATYKEDIIRFRVSLNCGIVELQEEISKRLKLEIGAFDIKYLDDDNEWVLISCDADLQECMDVLTSSGSNMIRLVVHDTVSILGSSCESSGN

**13.AhNKLM1**

MRVWPVLLILLLQFVEPSHGQTKSQQNNTGFQCSGRSYPCQAYAFYRAQSQFLDLASIGDLFQVSRLMIANPSNISSNSVSSPLILNQQLFIPLTCSCNSINTTFGSISYANISYTIKPNDTFYLVSTNKFENLTTYPSVEVVNPNLVATNLQIGDNAIFPVFCKCPDKNTTVSNTRANYMISYVVQPSDNLSSIASRFGSQQKAITDVNGNKFNVYDTIFVPVTKLPVLSQPNTSTAAAPSPTPAGSSDDRAGTVRGLAIGLGIAGLLLMVVCAVWMYRESVLKGRMWAGRDEEEQRQKEGRVFSGGGDGKGSKPMDVKLMANVSDCLDKYRVFGIEELVEATDAFSDSCLIQGSVYKGTIDGETYAIKKMKWNAYEELKILQKVNHGNLVKLEGFCIDSEEGNCYLVYEYVENGSLNWWLHEEEGKNKEKLNWKTRVRIGIDIANGLQYIHEHTRPRVVHKDIKSSNILLDSNMRAKIANFGLAKSGMNAITMHIVGTQGYIAPEYLADGVVSTKMDVFSFGVVLLELISGREAIDEEGNLLWMSAMKTFEGVSSDQEKGRRVREWMDKAILRDTISMDSLLGVLGIAIACLHKEPSKRPSIVDVVYALCKSDDAGFETSEDGIGSPKVTARPTMMWIHKNTPEGHPVEQSYVVVILLARNIAAAPSSLRRFGRRYAMLFLGGADAREALVYADMIAANQDASLTVIRFLSANYVGDKEREKKLDDGIVTWFWVKNETNNRVKYREVVVKNGEETIAKQN

**14.AhNKLM2**

MRVWPVLLILLLQLVEPSHGQTKSQQNNTGFQCSGRSYPCQAYAFYRAQSQFLDLASIGDLFQVSRLMIANPSNISSDSVSSPLIQNQQLFIPLTCSCNSVNTTFGSMSYANISYTIKPNDTFFLVSTIKFENLTTYPSVEVVNPNLVATNLQIGDNAIFPVFCKCPDKNTTVSNTRANYMISYVVQPSDNLSSIASRFGSQQKAITDVNGNKFNVYDTIFVPVTKLPVLSQPNTSTAAAPSPTPAGSSDDRTGTVRGLAIGLGIAGLLLMVVCAVWLYRESVLKGRMWAGRDEEEQRQKEGRVFSGGRDGKVSKPMDVKLMANVSDCLDKYRVFGIEELVEATDAFSDSCLIQGSVYKGTIDGETYAIKKMKWNAYEELKILQKGRRKKQWNHSEEGNCYLVYEYVENGSLNWWLHEEEGKNKEKLNWKTRVRIGIDIANGLQYIHEHTRPRVVHKDIKSSNILLDSNMRAKIANFGLAKSGMNAITMHIVGTQGYIAPEYLADGVVSTKMDVFSFGVVLLELISGREAIDEEGNLLWMSAMKTFEGVSSDEEKGRRVREWMDKAMLRDTISMDSLLGVLGIAIACLHKEPSKRPSIVDVVYALCKSDDAGFETSEDGIGSPKVTAR

**15.AhNNup1**

MSRVASDTVGNGALVPFSEDTKDSLAVYPLHHGLAPPISRIAISWARGNSLRVSLFAAPSSEHSRTPQDHSGGKVLEVKLGVGDPEISDSRWRQIAYGSVAPFALLQSRRSALSEMIKSSSPYQMDWWENVLEYSKDITSLLGGPKLPPGPIIEERTDIVKKREEPTCLKAAWELLEIFYADKQSQAWLPEKLVDWLADYDSLFSSTHETVYGKLARFQKELVNIQVLEDDPRYWEVMSSALSVGWLDIVVKMLRLHGSYQLDQLSNRELENGLVEAVAVLISKMPRLCHESTNGKLGELFKSKPDFIKAWEKWRSQITKLDCSPFWIQCDNHHTREGLRNLLQIMLGNTESLCMATCYWIELYISHFLYIRPFTTGIESMYNLAQKCIQLKPPSSNHKLTGLIVGILEENTEVVLAESSREFGPWMVAHAIELLTAGSEQAEIVLHDERYNLGGISMIELHRLVYAQVLSSHALTWQIAPIYLTSCMKQGMGLLENLLYRQSIQHNDLLLKNIEICRLYELDHISSDIMKVAGVYHWKHGHKGAGVYWLQRSKDTSRLNRIAQQLFDSVGKSISDESFKQWEGLIELLGSESKPAGGLEFLHKYRDFKRSLQQIYGGKSTDAARQAVGSLILLMKNPSTPQRFWLPLLYDSLKLFNWKECPLLNVSETNLLLNKLQELSWARLRPHFSEPNLPAEALSSIRLALATNLGRAILDE

**16.AhNNup2**

MSRVASDTVGNGALVPFSEDTKDSLAVYPLHHGLAPPISRIAISWARGNSLRVSLFVTPSSEPSQTPQDQSGGKVLEVKLGVGDPEISDSRWRQIAYGSVAPFALLQSRRSALSEMIKSSSPYQMDWWENVLEYSKDITSLLSGPKLPPGPIIEERTDIVKKREEPTCLKAAWELLEIFYADKQSQAWLPEKLVDWLADYDSLFSSTHETVYGKLARFQKELVDIQVLEDDPRYWEVMSSALSVGWLDIVVKMLRLHGSYQLDQLSNRELENGLVEAVAVLISKMPRLCHESTNGKLGELFKSKPDFIKAWEKWRSQITKLDCSPFWIQCGNHHTREGLRNLLQIMLGNTESLCMATCYWIELYISHFLYIRPFTTGIESMYNLAQKCIQLKPPSSNHKLTGLIVGILEENTEVVLAESSREFGPWMVAHAIELLTAGSEQAEIVLHDERYKLGGISMIELHRLVYAQVLSSHALTWQIAPIYLTSCMKQGMGLLENLLYRQSIQHNDLLLKNIEICRLYELDHISSDIMKVAGVYHWKHGHKGAGVYWLQQSKDTSRLNRIAQQLFDSVGKSISDESFKQWEGLIELLGSESKPAGGLEFLHKYRDFKRSLQQVYGGKATDAARQAVGSLILLMKNPSTPQRFWLPLLYDSLKLFNWHECPLLNVSETNLLLNKLQELSWARLRPHFSEPNLPAEALSSIRLALATNLGRAILDE

**17.AhNNup3**

MFSSAPKKKNTYATPLRDHGGAAAAAAANLFHSPATPQSRQRSSFIFNENAVPNRPSTGTPAPWAPRLSVLARVPQVDRSGKGDDTDPIKPVFVAEFPQLVRDEQATLLHKRVSVEGLGSGGIDKDTSLAWITCGNRVFIWSYLSPASGMRCVVLEIPSKVLEDGDTGKSDAGSWLLCVVNCDDTSKGTNKVPKHCSSAAVIMCNWKTRAVIYWPDIYSESHNPPVTSVASSDELETVLTPDRRSSFGKHRRQSKVGGSLNGLHTFNSLIASVVPGCKFVCVALACSSNGELWQFHCGPDGIRRRKVYENVTRSPQQGGESGQNVSNKWYPRSLTWRFPHHSPKESNRQFFLLTDHEIQCFKVELSSDMHVSKLWSQEIVGTDAEVGIKKDLAGQKKIWPLDVQVDDHGKVITILVVTLCNDRISSSSYMQYSILTLQYKSGLDSETTNDRILEKKSPMEVIIPKARYEDEDFLFSMRLRVGGKPSGSTVVISGDGTATVSHYHRNLTRLYKFDLPYDAGKVLDASVLPSADDYEEGAWVVLTEKAGIWAIPEKAVILGGVEPPERSLSRKGSSNERSAQEEIRNLTVAGNFAPRRASSEAWGTGDRQRAVLSGVARRTAQDEESEALLNLLFNDFLSSGQVDRALEKLETSGSFQRDGETNVFVRTSKSIIDTLAKHWTTTRGAEILAMAVVSTQLLEKQQKHQKFLQFLALSKCHEELCSRQRHALQIILEHGEKLSAMIQLRELQNMISQNRSASVNSLGSSSDIQMSGALWDLIQLVGERARRNTVLLMDRDNAEVFYSKVSDLEDFFYCLDAEIEYVIRPEHPFEIQFQRACELSNACVTIITTCLNYKNENHLWYPPPEGLTPWYCQPVVRKGIWSGASVLLRLLSEISGFDKSSKLDLYSHLEALAEVLLEAYSGAVTAKIECGEEHKGLLNEYWERRDALLESLYQQVKEFEATYKDSIEGSEGMTGDAILKIMSHLLSIAKRHGCYKVMWTICCDVNDSELLRNIMHESLGPDGGFSYYVFKKLHESRQFSELLRLGEEFPEELSVFLKEHPDLLWLHDLFLHQYSSASETLHELALAQNVQSTSVAEEGEQEYSKLNLKLSDRKNLLYLSKIAAFAAGRDAGTQVKVGRIEADLKILKLQEQVMEGFPSIKDMQLVEHQLLHPEDLIKLCLEGEGREFSLWAFDVFAWTSASFRKVYRRLLEDCWRKAASQDDWSKLHDSYIVEGWSDEETLQNLKSTILFQASSRCYGPGAVTFEEGFDQVLPLRQENMETPGDTSSSVEAILMQHKDFPVAGKLMLMAIMLGCEEGGDTTYEEGPSPME

**18.AhNNup4**

MFSSAPKKKNTNATPLRDHGGGAAAAAANLFHSPATPQSRQRSSFIFNENAVPNRPSTGTPAPWAPRLSVLARVPQVDRSGKGDDTDPIKPVFVAEFPQLVRDEQATLLHKRVSVEGLGSGGIDKDTSLAWITCGNRVFIWSYLSPASGMRCVVLEIPSKVLEDSDTGKSDAGSWLLCVVNCDDTSKGMNKVPKHCSSAAVIMCNWKTRAVIYWPDIYSESHKPVISVASSDELETVLTPDRRSSFGKQRRQSKVGGSLNGLHTFNSLIASVVPGCKFVCVALACSSNGELWQFHCGPDGIRRRKVYENVTRSPQHGGESGQNVSNKWYPRSLTWRFPHHSPKESNRQFFLLTDHEIQCFKVELSSDMHVSKLWSQEIVGTDAEVGIKKDLAGQKKIWPLDVQVDDHGKVITILVVTLCNDRISSSSYMQYSILTLQYKSGLDSETTNDRILEKKSPMEVIIPKARYEDEDFLFSMRLRVGGKPSGSTVVISGDGTATVSHYHRNLTRLYKFDLPYDAGKVLDASVLPSADDYEEGAWVVLTEKAGIWAIPEKAVILGGVEPPERSLSRKGSSNERSAQEEIRNLTVAGNFAPRRASSEAWGTGDRQRAVLSGVARRTAQDEESEALLNLLFNDFLSSGQVDRALEKLETSGSFQRDGETNVFVRTSKSIIDTLAKHWTTTRGAEILAMAVVSTQLLEKQQKHQKFLQFLALSKCHEELCSRQRHALQIILEHGEKLSAMIQLRELQNMISQNRSASVNSLGSSSDIQMSGALWDLIQLVGERARRNTVLLMDRDNAEVFYSKVSDLEDFFYCLDAEIEYVIRPEHPFEIQFQRACELSNACVTIITTCLNYKNENHLWYPPPEGLTPWYCQPVVRKGIWSGASVLLRLLSEISGLDKSSKLDLYSHLEALAEVLLEAYSGAVTAKIECGEEHKGLLNEYWERRDALLESLYQQVKEFEATYKDSIEGSEGMTGDAILKIMSHLLSIAKRHGCYKVMWTICCDVNDSELLRNIMHESLGPDGGFSYYVFKKLHESRQFSELLRLGEEFPEELSVFLKEHSDLLWLHDLFLHQYSSASETLHELALAQNVQSTSVAEEGEQEYLKLNLKLSDRKNLLYLSKIAAFAAGRDAGTQVKVDRIEADLKILKLQEQVMEGFRSIKDNQLVEHQLHHPEDLIKLCLEGEGREFSLWAFDVFAWTSASFRKVYRRLLEDCWRKAASQDDWSKLHDSYIVEGWSDEETLQNLKSTILFQASSRCYGPGAATFEEGFDQVLPLRQENMETPGDTSSSVEAILMQHKDFPVAGKLMLMAIMLGCEEGGDTTYEEGPSPME

**19.AhNKEF5**

MGHESRKLLDEYEVSEILGRGGFSVVRKGIKKSSSDEKTHVAIKTLRRVSASTTTPGCLPRERSNMGFPTWRQVSVSDALLTNEILVMRKIVENVSPHPNVVDLYDVYEDSNGVHLVLELCSGGELFDRIVAQDRYSETEAATVIRQIAAGLEAIHKANIVHRDLKPENCLFLDKRKDSPLKIMDFGLSSVEEFTDPVVGLFGSIDYVSPEALSQGKITAKSDMWSLGVILYILLSGYPPFIAQSNRQKQQMIMNGNFSFYEKTWKGISQSAKQLISSLLTVDPSRRPSAQELLSHPWVIGDVAKDDQMDPEIVSRLQSFNARRKLRAAAIASVWSTTVFLRTKKLKSLIGSYDLTEEEIENLRIHFKKICGNGDNATLSKFEEVLKAINMPSLIPLAPRIFDLFDNNRDGTVDMREILCGLSSLKNSKGDDALRLCFQMYDADRSGCITKEEVASMLRALPDDCLPVDITEPGKLDEIFDRMDANSDGKVTFEEFKAAMQRDSSLQDVVLSSLRPL

**20.AhNKEF6**

MGHESRKLLDEYEVSEILGRGGFSVVRKGIKKSSSDEKTHVAIKTLRRVSASTTTPGCLPRERSNMGFPTWRQVSVSDALLTNEILVMRKIVENVSPHPNVVDLYDVYEDSNGVHLVLELCSGGELFDRIVAQDRYSETEAATVIRQIAAGLEAIHKANIVHRDLKPENCLFLDKRKDSPLKIMDFGLSSVEEFTDPVVGLFGSIDYVSPEALSQGKITAKSDMWSLGVILYILLSGYPPFIAQSNRQKQQMIMNGNFSFYEKTWKGISQSAKQLISSLLTVDPIRRPSAQELLSHPWVIGDVAKDDQMDPEIVSRLQSFNARRKLRAAAIASVWSTTVFLRTKKLKSLIGSYDLTEEEIENLRIHFKKICGNGDNATLSKFEEVLKAINMPSLIPLAPRIFDLFDNNRDGTVDMREILCGLSSLKNSKGDDALRLCFQMYDADRSGCITKEEVASMLRALPDDCLPVDITEPGKLDEIFDRMDANSDGKVTFEEFKAAMQRDSSLQDVVLSSLRPL

**21.AhNPR5**

MSESEEDKTDFASPKSKEEQQQQSPPQLPPPSAMDFDLDLETSWPLDHLSFVSNNPMSPFLFPISSEQPSSPLWLFSDAEDERHNNTLASAPAFSDFHKIFSCDSNSVTEKPVENANDEDKKLLPPIVAMPPLEILDRYCVIKERMTQALRYFKELTEQNVLAQVWAPVRNGNRFVLTTSGQPFVLDPHSNGLHQYRTVSLMYVFSADGEKEESLGLPGRVYQQKVPEWTPDVQYYSTKEYPRRDHAQHYNVRGTLALPVFEPSMQSCVGVLELIMTSQKINYAPEVDKICRALEAVNLKSSEILGHQYTQICNEGRQNALAEILEILTVVCETHNLPLAQTWVPCRHRSVLAHGGGLKKSCSSFDGSCMGKVCMSTTDIAFYIIDAHLWGFREACVEHHLQQGQGVAGRAFSSHSMSFCRNITRFCKIDYPLVHYALMFGLTSSFSICLRSSHTGDDDYVLEFFLPPRITDFNEQKALLGSILTIMKQHFQSLKIASGVELEQNALVETIEATIEGVHLRFESIPVRQDASPNVREELAQDPSLQKIMMGCNDGGSIGDQIPSLETKNTNKPSERKRGKTEKSISLEVLQRYFAGSLKDAAKSLGVCPTTMKRICRQHGISRWPSRKINKVNRSLSKLKRVIESVQGAEGAFTLNPLSTSPLPFPEHSTPNKFSQQASPTEPQIRENELDASKVLETTRIARAQCLEKMVNDKSGSIREVGKETKGPRAKSCSSADSTNPTSHGSCHGSPPIESSPVKDIFITSNNDQCVGLRSPEATMQPPNNTLSYPTTCAMPDMVATELQEPFGGMLVEDAGSSKDLRNLCPSVAEAIVEDMAPEPCRTNPPFSGLAPKQCMDPLKETVTPFASRKEMKTVTIKATYREDIIRFRVSLNCGIVELKEEVAKRLKLEVGTFDIKYLDDDHEWVLIACDADLQECIDVSRSSASNIIRVLVHEITSHLGSSCESSGE

**22. AhNPR6**

MSESEEDKTDFASLKSKEDQQQSPPQLPPPSAMDFDLDLETSWPLDHLSFVSNNPMSPFLFPISSEQPSSPLWLFSDAEDERHNNTLASAPAFSDFHKIFSCDSNSVTEKPVENANDEDKKLLPPIVAMPPLEILDRYCVIKERMTQALRYYKELTEQNVLAQVWAPVRNGNRFVLTTSGQPFVLDPHSNGLHQYRTVSLMYVFSADGEKEESLGLPGRVYQQKVPEWTPDVQYYSTKEYPRRDHAQHYNVRGTLALPVFEPSMQSCVGVLELIMTSQKINYAPEVDKICRALEAVNLKSSEILGHQYAQICNEGRQNALAEILEILTVVCETHNLPLAQTWVPCRHRSVLAHGGGLKKSCSSFDGSCMGKVCMSTTDIAFYIIDAHLWGFREACVEHHLQQGQGVAGRAFSSHSMSFCRNITRFCKIDYPLVHYALMFGLTSSFSICLRSSHTGDDDYVLEFFLPPRITDFNEQKALLGSILTIMKQHFQSLKIASGVELEQNALVETIEATIEGVHLRFESIPVRQDASPNVREELAQDPSLQKIMMGCNDGGSIGDQIPSLETKNTNKPSERKRGKTEKSISLEVLQRYFAGSLKDAAKSLGVCPTTMKRICRQHGISRWPSRKINKVNRSLSKLKRVIESVQGAEGAFALNPLSTSPLPFPEHSTPNKFSQQASPTEPQIRENELDASKVLETTRIARAQCLEKMVNDKSGSIREVGKETKGPRAKSCSSADSTNPTSHGSCHGSPPIESSPVKDIFITSNNDQCVGLRSPEATMQPPNNTLSYPTTCTMPDMVATELQEPFGGMLVEDAGSSKDLRNLCPSVAEAIVEDMAPEPCRTNPPFSGLAPKQCMDPLKETVTPFAARIEMKTVTIKATYREDIIRFRVSLNCGIVELKEEVAKRLKLEVGTFDIKYLDDDHEWVLIACDADLQECIDVSRSSASNIIRVLVHEITSNLGSSCESSG

**23.AhNKLM3**

MAFFLPSLSSSIFLAFMLFSVTSIPTQSQQVNGTDFSCPVDSPSSCGTYVTYIAKSPNFLSLSNISDIFDTSPLSIARASNIKNEGDKLVPGQVLLIPVTCGCTQNQSFANITYELRQGDVYDIVSKTTYENLTNWRAVNNSNPDLNPVLLPIGVKVLFPLFCRCPSKKQLQKGIEYMITYVWQNNDNVSSVAAKFGASPVDILSENNYGGNFTAATYLPVLIPVTKLPVLTQPEASHGRKRSIQIPVIISISLGFTLVVAVIVISMVYAYLYQRKRTLNRRDLSAGTADKLLSGVSGYVSKPTVYEANEVIKATMNLSEQCKLGGTVYKAKIEGQVLAVKKVNQVVSEELNILQKVNHGNLVKLMGVSSDSDGNHFLVYEYADNGSLDEWLFSKLSLKASLTWYQRINIALDVAMGLQYLHEHTYPRIVHRDITTSNILLDSNFKAKIGNFSMVRTTTNPMISKIDVFAFGVVLIELLTGKKAMTTKADGEVVMLWKDIRKMFEVEDEKEKEECLRRWMDPKLECLYPVDYALSLVTLAANCTADVSLSRPTMAEVVLGLSLLTQPSQAALERSLTSSALEAEVTHVATPIAAR

**24.AhNKTyr**

MYDFVSKTTYENLTNWRAVNDSNPDLNPVLLPVGVKVLFPLFCRCPSKKQLQKGIEYMITYVWQNNDNVSSVAAKFGASAVDILSENNYGGNFTAATYLPVLIPVTKLPVLTQPEPSHGRKRSIQIPVIISISLGFTLVVAVIVISMVYAYLYQRKRTLNRRDSSAGTADKLLSGVSGYVSKPTVYEANEVIKATMNLSEQCKLGGTVYKAKIEGQVLAVKKVNQVVSEELNILQKVNHGNLVKLMGVSSDSDGNHFLVYEYADNGSLDGWLFSKLSLKASLTWYQRINIALDVAMGLQYLHEHTYPRIVHRDITTSNILLDSNFKAKIGNFSMVRTTTNPMISKIDVFAFGVVLIELLTGRKAMTTKADGEVVMLWKDIRKMFEVEDEKEKEECLRRWMDPKLECLYPVDYALSLATLAANCTADVSLSRPTMAEVVLGLSLLTQPSQAALERSLTSSALEAEVTHVATPIAAR

**25. AhNSur1**

MKFICEMVKLSLSVIIIQSSIIFSRELGSPNHIKTATFYTKKFVLEPGKITRKNFFDVEFPRGHTGIKNLQAELVDEHGNSIPLYEAYLHHYFVFRYFENITMSQYANKSQPAYGKYFRRNDGVCQGYVNSISWGLGGDARKTSVELPDPFRVEVGIHPEDVPKEYDEEKWFINVLAIDTRGTEDKKGCSQCRCDLYNVKSQDLKNTTSIDGKLLFSSDYKGGIFCCEKKSQCKLQKGYKKEQKRKVTLKYTISWVEWDQHQVPLKFYILDVTDQVTYNGSKPIHNCAVEYSIIPEKANEEQYYIKKTNVPMEKGGNVIYTTAHIHSGIVNATLYGEDGRILCEIKAISGMGKEAGNEEGYAIGASGCYPKPGSMKIKDGEILTIEFAHENKYTTGLMGHFYVYLAEELPKSF

**26.AhNSur2**

MKFICEMVILSLSVIIIQSSIIFSRELGSPNHIKTATFYTKKFVLEPGKITRKNFFDVKFPRGHIGIKNLRAELVDEHGNSIPLYEAYLHHYFIFRYFENITMSQYANKSQPAYGKYFRRNDGVCQGYVNSISWGLGGDARKTSVELPDPFRVEVGTHPENVPKEYDEEKWFINVLAIDTRGTEDKKGCSQCRCDLYNVKSKDLENTTSVDGKSLFSSDYKGGIFCCEKKSQCKLQKGYKKEQKRNVTLKYTISWVEWDQHQVPLKFYILDVTDQVTYNGSKPIHNCAVEYSITPKKTNEEQYYIKKTNVPMEKGGNVIYTTAHIHSGIVNATLYGEDGRILCEIKAISGTGKEAGNEEGYAVGASGCYPKPSSMKIKDGEILTIEFVHENKYTTGLMGHFYVYLAEELLKSF

**27.AhNSur3**

MSYASKDMVISLAILLLMLGTPCSSAFWKTRNKIKTAVYLSPKIELGPGSVSNKFYYDIEFPRGHVAIKSFNAEVVDEAGNPVPLHKTYLHHWIIVGYHESKSKLATHTKHDLHRVVCVSDSVSKSHIILRNSGVCQGNILGQYFGLGSETRGTATDIPDPFGIEIGNPAEIPEGYEEKWLLNVHAIDTRGVEDKLGCTECKCHLYNVTVNEYGNPLPPDYTGGLYCCYDETQCRLKKGFQGPKRSLYLRYTVKWIDWDEYVVPVKIYIIDVTDTLKISDTSNIASSNHDCRIEYEVDPCNIDPKKGNGCLDVKRTTVPLEKGGYVVYAVAHQHSGNESGYIVGMTTCYPKPGSVKIIDGEKLTLESNYSSTTRGHTGVMGLFYLLVAEQLPHQH

**28.AhNSur4**

MSRQANQSQPIYGKYFRRNDGVCQGSVNSYSWGLGVDARKTSLELPDPFRIEVGTHPENVPKEYNEEKWLFNIMVIDTRGTEDKKGCTECRCDHYNVKSEDFISKTGIDGKLMSSDYKGGIFCCEKTSQCKLQKGYNNRQERKASLKYTVTWVDWDQYQVPIKFYILDVTDQVTYNGSEPIHNCMVEYSITPQNTDIGHYHIKRTKIPMKKGGNLIYSTAHVHPGIVNATLYVENGKVLCAVKPTYGTGEEPGNEKGYVVGMSGCYPKPGSIKIQDGEILTVDKHCQLGVILAREKKYLPSHSRSTSFKGTSQQEHIAQSSSIQNEVHSHSLSDVDFLNDEEK

**29.AhNSur5**

MSRQANQSQPIYGKYFRRNDGVCQGSVNSYSWGLGVDARKTSLELTDPFRIEVGTHPENVPMEYNEEKWLFDIMVIDIRGTEDKKGCTECRCDHYNVKSEDFVSKTGIDGKPMSVTWVDWDQYQVPIKFYILDVTDQVTYNGSEPIHNCMVEYSITPQNTDIGHYHIKKTKIPMKKGGNLIYSTVHVHPGIVNATLYGENGKVLCAVQPTYGTGEEPGNEKGYVVGMSGKVTITDLFDIEFPRGHIGIKNFQAELVDEHQNSLPLYEAYLHHYFVLRYFENVTMSRQANQSQPIYGKYFRRNDGVCQGSVNSYSWGLGVDARKTSLELPDPFRIEVGTHPENVPKEYNEEKWLFDIMVIDTRGTEDKKGCTECRCDHYNVKSEDFVSKTGIDGKPMSGDYKGGIFCCEKTSQCKLQKGYNNKQQRKAFLKYTITWVDWDQYQVPIKFYILDVTDQVTYNGSEPIHNCMVEYSITPQNTDIGHYHIKKTKIPMKKGGNLIYSTVHVHPGIVNATLYGEEKSQEMKKAMLLECLVVIQNQALSRFRMEKY

**30.AhNSur6**

MKFIPEVLLLSLTIILLQSSIIFARQYENSNHIKTATFYSEQFVLEPGKVTITDLFDIEFPRGHIGIKNFQAELVDEHRNSLPLYEAYLHHYFVLRYFENVTMSRQANQSQPIYGKYFRRNDGVCQGSVNSYSWGLGVDARKTSLELPDPFRIEVGTHLRMSQRSRMKRNGYSISWLLTHVVQKTKKVAPNADVTIIMSKVKTFKTGIDGKPMSSDYKGGIFCCEKTSQCKLQQGYNNRQQRKASLKYTVTWVDWDQYQVPIKFYILDVTDQVTYNGSEPIHNCMVEYSITPQNTDIGHYHIKRTKIPMKKGGNLIYSTAHVHPGIVNATLYRENGMVLCAVQPTYGTGEEPGNEKGYVVGMSGCYPKPGSIKIQDGEILTVDYLGSRKKHLASHSRSTSSKGTSQKEHIAQSSSIQNEVHSHSLSDVDFLNVEEVDVVAISRKKWNTT

**31.AhNSur7**

MSTNKMCEAISLAILLLVLGSPCSASLFSWKTENKIKTAVHLSPKIEIGPGAVSNKNYYDIDFPRGHVALKGFTAEVVDEAGNSVPLHETYLHHWALIRYRQSKSKLATHASYDPHRVLHVSDSIFESGVVRNSGICQGNVLGQYYGIGSETRGTNTDIPDPFGLEIGNPEEGYEEKWMLNIHAIDTRGVEDKLGCTECRCDLYNVTVNEFGKPLPPDYIGGLNCCYHETQCRLKKGFQGPKRSLYLRYTVKWIDWDEYVVPVKIYIIDVTDTLKISDTSSITSSDHDCRVEYEVDPCNRDTKKGNDCLDVKRTSLPFPKGGYVVYGVAHQHSAGIGATLYGQDGRVICTSMAKYGTGDEAGNEAGYIVGMTTCYPKPGSVKIIDGEKVTLESNYSSSSRSHTGVMGLFYLLVAEQLPHQHYFTHSSSFFRNINNVIN

**32.AhNSur8**

MFLSSNILPINPGCWFRIQLHSALWCGVALLNMSTNKMCEMISLAILLLVLGSPCSAAFSWKTENKIKTAVHLSPKIEIGPGSVSNKNYYDIDFPRGHVALKGFTAEVVDEAGNSVPLHETYLHHWALIRYRQSKSKLATHASYDPHRVLHVSDSISESGVVRNSGICQGNVLGQYYGIGSETRGTNTDIPDPFGLEIGNPEEGYEEKWMLNIHAIDTRGVEDKLGCTECRCDLYNVTVNEFGKPLPPDYIGGLNCCYHETQCRLKKGFQAPKRSLYLRYTVKWMDWDEYVVPVKIYIIDVTDTLKISDTSSITSSDHDCRVEYEVDPCNTDTKKGNDCLDVKRTRLPFPKGGYVVYGVAHQHSAGIGATLYGQDGRVICTSMANYGTGDEAGNEAGYIVGMTTCYPKPGSVKIIDGEKLTLESNYSSSTRSHTGVMGLFYLLVAEQLPHQHYFTHSSSFFRNRNINNVFN

**33.AhNSur9**

MKMWNVNHCCWAMVVMLSSTVVPYSWAFVNEKTSVFLSPKIEIGAGKSSNKFYYDVDFPRGHIALKSFNAEIVDEKGNSVPLYEAYLHHWIVMKYHQPKNATKTNPGIEIVQNSGLCQYNTLPYYFGVGSETRGVKTHIPDPYAIEAGNPPEGYDEKWVINVHAIDTRGVQDKIGCIECRCDLFNITKDSDGKPLSPSYHGGLTCCPDESQCLLKKGFKPQNRTLYLKYTVKWVTWEHFILPLRVYVLDVTDVVKNNTHNCLVEYDVLPCKYGGGKCVDVRRTKLPMNKGGYVIYGVAHEHVGGIGSTLYGQDGKVICNSLPKYGNGSEAGNEKGYLVGMTTCYPQPGSIKISNGEVLTLEVDYSNTKLHSGVMGLFYLLVADDLPHHKN

**34.AhNSur10**

MWNVNHCCWAMVVMLSSTYSWAFVTEKTAVFLSPKIEIGAGKSSNKLYYDVDFPRGHIALKSFNAEIVDEKGNSVPLYEAYLHHWIVMKYHQPKNATKTNPGIEIVQNSGLCQYNTLPYYFGVGSETRGVETHIPDPYGIEAGNPPKGYDEKWVINVHAIDTRGVQDKIGCIECRCDLFNITKDSDGKPLSPSYHGGLTCCPDESQCLLKKGFKPQNRTLYLKYTVKWVTWEHYILPLRVYVLDVTDVVKNNTHNCLVEYDVLPCKYGGKCVDVRRTKLPMNKGGYVIYGVAHEHVGGTGSTLYGQDGRVICNSLPKYGNGSEAGNEKGYLVGMTTCYPRPGSIKISNGEVLTLEVDYSNTKLHSGVMGLFYLLVADDLPHHKN

**35.AhNSur11**

MAYKNLSGLSKSKRCRLVLRLSLRLTINMSYASKDMVISLAILLLMLGTPCSSAFWKTQNKIKTAVHLSPKIELGPGSVSNKFYYDIEFPRGHVALKSFNAEVVDEAGNPVPLHKTYLHHWIIVGYHESKSKLATHTKYDLHRVVRVSDSVSKSHIILRNSGVCQGNILGQYFGLGSETRGTATDIPDPFGIEIGNPAEIPEGYEEKWLLNVHAIDTRGVEDKLGCTECKCHLYNVTVNEYGNPLPPDYAGGLYCCYDETQCRLKKGFQGPKRSLYLRYTVKWIDWDEYVVPVKIYIIDVTDTLKISDTSNIASSNHDCRIEYEVDPCNIDPKKKGNGCVDVKRTTVPLEKGGYVVYAVAHQHSGGIGSTLYGQDGRVICTSMANYGTGDNAGNESGYIVGMTTCYPKPGSVKIIDGEKLTLESNYSSTTRGHTGVMGLFYLLVAEQLPHQH

**36.AhNSur12**

MARRQGNLTMFGLSISPKLCASLSLPFVGDATGAMENMKFIPEVLLLSLTIILLQSSIIFARQYENSNHIKTATFYSEQFVLEPGKVTITDLFDIEFPRGHIGIKNFQAELVDEHRNSLPLYEAYLHHYFVLRYFENVTMSRQANQSQPIYGKYFRRNDGVCQGSVNSYSWGLGVDARKTSLELPDPFRIEVGTHPENVPKEYNEEKWLFDIMVIDTRGTEDKNGCTECRCDHYNVKSEDFVSKTGIDGKPMSGDYKGGIFCCEKTSQCKLQKGYNNKQPRKASLKYTVTWVDWDQYQVPIKFYILDVTDQVTYNGSEPIHNCMVEYSITPQNTDIGHYHIKKTKIPMKKGGNLIYSIVHVHPGIVNATLYGENGKVLCAVQPTYGTGEEPGNEKGYVVGMSGCYPKPGSIKIQDGEILTLNKI

**37.AhNSur13**

MKFIPEVLLLSLTIILLQSSIIFARQYENSNHIKTATFYSEQFVLEPGKVTITDLFDIEFPRGHIGIKNFQAELVDEHRNSLPLYEAYLHHYFVLRYFENVTMSRQANQSQPIYGKYFRRNDGVCQGSVNSYSWGLGVDARKTSLELPDPFRIEVGTHPENVPKEYNEEKWLFNIMVIDTRGTEDKKGCTECRCDHYNVKSEDFVSKTGIDGKPMSSDYKGGIFCCEKTSQCKLQKGYNNRQERKASLKYTITWVDWDQYQVPIKFYILDVTDQVTYNGSEPIHNCMVEYSITPQNTDIGHYHIKRTKIPMKKGGNLIYSTAHVHPGIVNATLYGENGKVLCAVQPTYGTGEEPGNEKGYVVGMSGCYPKPGSIKIQDGEILTV

**38.AhNSur14**

MAHIENMKFIPEVLLLSLTIILLQSSIIFARQYENSNHIKTATFYSEQFVLEPGKVTITDLFDIEFPRGHIGIKNFQAELVDEHRNSLPLYEAYLHHYFVLRYFENVTMSRQANQSQPIYGKYFRRNDGVCQGSVNSYSWGLGVDARKTSLELPDPFRIEVGTHPENVPKEYNEEKWLFNIMVIDTRGTEDKKGCTECRCDHYNVKSEDFVSKTGIDGKPMSSDYKGGIFCCEKTSQCKLQQGYNNRQQRKASLKYTVTWVDWDQYQVPIKFYILDVTDQVTYNGSEPIHNCMVEYSITPQNTDIGHYHIKRTKIPMKKGGNLIYSTAHVHPGIINATLYGENGKVLCAVQPTYGTGEEPGNEKGYVVGMSGCYPKPGSIKIQDDEILTA

**39.AhNSur15**

MKFICEVVILSFSIIVIQSSITFSRELEGPNHIKTTTFYTKTFVLEPGKVSRKTFFDVEFPRGHIGIKNLQAELVDEHGNSIPLYEAYLHHYFVLRYFENITMSQHANESQPNYGKYFKRNDGACQTFVNSISWGLGVDARRTNTELPDPFRVEVGTHPEDVPKEYDEEKWLINILVIDTRGAEDKKGCSQCRCDLLNVKSEDLRNTTGVDGTPLSSDYKGGIFCCEKKSQCKLQKGYNEKQKRKVAIKYTISWVEWDQQQVPLKFYILDVTDQVTYNGSEPIHHCAVEYSINPEKTDEGHYHIKKTNIPMKKGGSLIYITAHVHSGIVNATLYGEDGRRLCEIKPIYGTGKEAGNEEGYAVGASGCYPKPGSMKIKDGENLTAEFIHENKYTTGLMGHFYVYLAEDLPKSF

**40.AhNSur16**

MKFICEVVILSFSIIVIQSSITFSRELEGPNHIKTTTFYTKTFVLEPGKVSRKTFFDVEFPRGHIGIKNLQAELVDEHGNSIPLYEAYLHHYFVLRYFENITMSQHANENQPNYGKYFKRNDGACQTFVNSISWGLGVDARRTSTELPDPFRVEVGTHPEDVPKEYDEEKWLINILVIDTRGAEDKKGCSQCRCDLLNVKSEDLRNTTGVDGTPLSSDYKGGIFCCEKKSQCKLQQGYNEKQKRKVAIKYTISWVEWDQQQVPLKFYILDVTDQVTYNGSEPIHHCAVEYSINPEKTDEGHYHIKKTNIPMKKGGSLIYITAHVHSGIVNATLYGEDGRRLCEIKPIYGTGKEAGNEEGYAVGASGCYPKPDSMKIKDGENLTAEFIHENKYTTGLMGHFYVYLAEDLPKSL

**41.AhNSur17**

MKFICEVVILSFSIIVIQSSITFSRELEGPNHIKTTTFYTKTFVLEPGKVSRKTFFDVEFPRGHIGIKNLQAELVDEHGNSIPLYEAYLHHYFVLRYFENITMSQHANENQPNYGKYFKRNDGACQTFVNSISWGLGVDARRTSTELPDPFRVEVGTHPEDVPKEYDEEKWLINILVIDTRGAEDKKGCSQCRCDLLNVKSEDLRNTTGVDGTPLSSDYKGGIFCCEKKSQCKLQQGYNEKQKRKVAIKYTISWVEWDQQQVPLKFYILDVTDQVTYNGSEPIHHCAVEYSINPEKTDEGHYHIKKTNIPMKKGGSLIYITAHVHSGIVNATLYGEDGRRLCEIKPIYGTGKEAGNEEGYAVGASGCYPKPDSMKIKDGENLTAEFIHENKYTTGLMGHFYVYLAEDLPKSL

**42.AhNSur18**

MKFICEVVILSFSIIVIQSSITFSRELEGPNHIKTTTFYTKTFVLEPGKVSRKTFFDVEFPRGHIGIKNLQAELVDEHGNSIPLYEAYLHHYFVLRYFENITMSQHANENQPNYGKYFKRNDGACQTFVNSISWGLGVDARRTSTELPDPFRVEVGTHPEDVPKEYDEEKWLINILVIDTRGAEDKKGCSQCRCDLLNVKSEDLRNTTGVDGTPLSSDYKGGIFCCEKKSQCKLQQGYNEKQKRKVAIKYTISWVEWDQQQVPLKFYILDVTDQVTYNGSEPIHHCAVEYSINPEKTDEGHYHIKKTNIPMKKGGSLIYITAHVHSGIVNATLYGEDGRRLCEIKPIYGTGKEAGNEEGYAVGASGCYPKPDSMKIKDGENLTAEFIHENKYTTGLMGHFYVYLAEDLPKSL
