# Supplementary material for: Genome wide identification and characterization of nodulation related genes in Arachis hypogaea
Source: PLoS One. 2022 Sep 9;17(9):e0273768. doi: 10.1371/journal.pone.0273768 (PMC9462762; doi:10.1371/journal.pone.0273768)
Supplement: S3 Text — (DOCX) [file pone.0273768.s003.docx]

**1. *AhNMTL1***

GGCCAGCATTTAACCATCAAAAGAAAATTGAATGATTTTACACTATTAGATGCAATCTCA

CACCAGTAAAAATATCATTAATAGCTAATTGATGGCTAAAAATCACAAAATTTGCTGGTC

TTCTAGACTTTCTCAAACAGGATATGGATAAATGTTAGAGTGTTAGATGAATATGTATTC

AAGATACGTTTGACACATAGAAGTATTATGCTTCATAACTTTTTTTTTTTTAGTTACTAG

ATACTTGATAAGATTTATTTTAAGAGTGAAAAGTCAAAACTATACTAAAATACCATAAGG

TAAATGTTATATATTACCATTGTTCCTTCACTTCTATAGACAATAACCCACCGAAATGCA

TGCGTCTCAGTAAATTGAGAGAATGTGGTGGTGGCAGCGTAATCGTGTACGTAGAAGAAT

ACGACACTTTGTTAATACTTTAGTGCACCTCAAAATAACAATAAGAAATCAACTGAGAAT

GCCGTTTCCAATTAGAAATGGAATTTGTGATGATCTCAGGTTGATGAATATGTATATAAC

AAATGTTACATTGCCCAACTTTAATTACTTGCAAGTTTAGAATAACGGAAGCTAGGGACA

AGTTTTGATGAAGCTCAAAGTATAGCGTCTTCGTTTTAATTTTTAGACCAATTATATACT

ATACAAAAATACTAACGTAGCTCTTTTTTTTTTTTAAGTTTTATTTACTGGTAACTCCTA

CTGTCCAAGTCAATGGGGGGTGCATGCTCTTTCTGCCTCTGATTGAGCCACTTCTGATCA

TCGTGTTGATTTCTCATCAGTTTCAAAGTTGAAAATCTGAAAGGGAATTACTGTTAAGCA

TAATATAAGGATAGTTGCATTGACACTCTTGACAATACAATTATTTATTCATTTTATATT

ATTTAACTCTATCTCCACAAAAATACGTACAGAAGAAATAGTTCCTTTTTTTTTGTCTTA

TGTTTAATATCTTATTTTCAATCATGTTGCATCCATCGTTAGAGTGAAAGGGAGGGGGGA

ATGAAGGAAGAACAGGACCAATCAAGGACATTAATTACTCTCTCTAATTATATTAAATCA

CATAATCAAGTAAAGCAACAACAATTAATAAATATGAACCGAAGAAAACATATTTAACTA

AGGATTTTGTACATAGTGCAAGCTCCCTAGCTAGCTAACTCCTCTTCTATCTCCTTGTTA

GGAAGTTCCTACACGTTTCAAGTCAAACCCTTTATTCTTAATATCCATTCGAGTTCTATG

ATCTTCCATCTAACATTTGGATAAGTAATGATTTCAAATTCTTCCAATAGAAGAATTTTT

ATTGAGGTTCATGGTAGTAGTATACTGTAGGGTTGACCAAGGTGCCTTAGTTTTTGTTAT

CTGAATTAATAGTAGGACTAGTGAGTGTTGGTTGCATAAGGCAGAGAAAGAAGTGAGAAA

TTCAAGAGATTTGTATAGTGGCATGGCTTCATGTTTAGCAACCCCATGGGGTAACCTATA

A

**2. *AhNMTL2***

GGCCAGCATTTAACCATCAAAAGAAAATTGAATGATTTTACACTATTAGATGCAATCTCA

CACCAGTAAAAATATCATTAATAGCTAATTGATGGCTAAAAATCACAAAATTTGCTGGTC

TTCTAGACTTTCTCAAACAGGATATGGATAAATGTTAGAGTGTTAGATGAATATGTATTC

AAGATACGTTTGACACATAGAAGTATTATGCTTCATAACTTTTTTTTTTTTAGTTACTAG

ATACTTGATAAGATTTATTTTAAGAGTGAAAAGTCAAAACTATACTAAAATACCATAAGG

TAAATGTTATATATTACCATTGTTCCTTCACTTCTATAGACAATAACCCACCGAAATGCA

TGCGTCTCAGTAAATTGAGAGAATGTGGTGGTGGCAGCGTAATCGTGTACGTAGAAGAAT

ACGACACTTTGTTAATACTTTAGTGCACCTCAAAATAACAATAAGAAATCAACTGAGAAT

GCCGTTTCCAATTAGAAATGGAATTTGTGATGATCTCAGGTTGATGAATATGTATATAAC

AAATGTTACATTGCCCAACTTTAATTACTTGCAAGTTTAGAATAACGGAAGCTAGGGACA

AGTTTTGATGAAGCTCAAAGTATAGCGTCTTCGTTTTAATTTTTAGACCAATTATATACT

ATACAAAAATACTAACGTAGCTCTTTTTTTTTTTTAAGTTTTATTTACTGGTAACTCCTA

CTGTCCAAGTCAATGGGGGGTGCATGCTCTTTCTGCCTCTGATTGAGCCACTTCTGATCA

TCGTGTTGATTTCTCATCAGTTTCAAAGTTGAAAATCTGAAAGGGAATTACTGTTAAGCA

TAATATAAGGATAGTTGCATTGACACTCTTGACAATACAATTATTTATTCATTTTATATT

ATTTAACTCTATCTCCACAAAAATACGTACAGAAGAAATAGTTCCTTTTTTTTTGTCTTA

TGTTTAATATCTTATTTTCAATCATGTTGCATCCATCGTTAGAGTGAAAGGGAGGGGGGA

ATGAAGGAAGAACAGGACCAATCAAGGACATTAATTACTCTCTCTAATTATATTAAATCA

CATAATCAAGTAAAGCAACAACAATTAATAAATATGAACCGAAGAAAACATATTTAACTA

AGGATTTTGTACATAGTGCAAGCTCCCTAGCTAGCTAACTCCTCTTCTATCTCCTTGTTA

GGAAGTTCCTACACGTTTCAAGTCAAACCCTTTATTCTTAATATCCATTCGAGTTCTATG

ATCTTCCATCTAACATTTGGATAAGTAATGATTTCAAATTCTTCCAATAGAAGAATTTTT

ATTGAGGTTCATGGTAGTAGTATACTGTAGGGTTGACCAAGGTGCCTTAGTTTTTGTTAT

CTGAATTAATAGTAGGACTAGTGAGTGTTGGTTGCATAAGGCAGAGAAAGAAGTGAGAAA

TTCAAGAGATTTGTATAGTGGCATGGCTTCATGTTTAGCAACCCCATGGGGTAACCTATA

A

**3. *AhNKEF1***

TTAATTCGAGTTCCAATAACTCTAGCCTTTCCCAAGACTAGGACCTGAGG

AATAAAAATTAATTCATCCACTTAACTTACCTTCATAGTTAGAGGCCAACAAAGTGGAAG

AAAAATCCAATTCTCATTACAATTGATAAGGATAACCAGGATAGGACTTCCTGTTTTCAT

CCTTGCCAAGAGTTTATTTTATAGTTATTTATTTATTTTATTCTTCTTATGCAACATACT

GCTCCCTAATTTCTAAAAACCCCTAATTTACAAACTCATAACCAATAATAAGAACATACC

TCCCTGCAATTCCTTGAGAAGACCACCCGAGGTTTAAATACTCGGTTATCAATTTTAAAA

GGGGTTTGTTACTTGTGACAACCAAAACGTTTGTATGAAAGGACTTTTGAAGGTTTAGAA

ACTATACTTGCAACGAGGATTTATCCGTAAATTTCTAGACCACGCAAAAGTTTTCTCTCA

TCAAAATGGCGCCGTTGCCGGGGAATTGTAAACGTGTGCCTTATTATTGGTTATTGTAAA

TATTTTCTTTTGCTTATTGATTTATTTTTAATTTTATTTTAATTTTTTTGCTTTTTCGTA

AATTAAGAGGTTATTAGTTTTTATTTTAAAATTTTTATCTTATCTTATCTTATTTCAAAA

ATCAAATTTCAAAATTCAAATTAATTTTTTTTTTCAAATTTCAAATTCAAAATTTAATAA

CCTTTTAATTTCAATTTGTTTTTATTTTCGTTTTATTTGTTACTATGAACTCTCACCCCT

TTGGCTATGAGTCTGGTTACAATAATGTTGCAGGAAGAAGAAATTACAATGAGAACAGGC

ATCAAGGATGGAACAATCAAAGATGGGAAGAGCCACAAGGATTTGATCAACCCTCATGGC

AACAACCACCTCTAATGGACTATCAACAACCACCACCATATGCCTATGAATCCTCTCCTC

AACATGACTTTGGACCACCATACTCACAAGCCCCTTTCCGCCATTCACCTCCATATGACC

CTAACCCTCAACCACCATACCAACCACCTTATGAGCCATATGAACCATATATAGAACCAC

CCCAATTCCAACCCAATTACTCACAAGAACCACCACTTCAATATTCACCATCTCCATATC

ATTATCAAGATGAACCACCTTCCTATTATGAACCCTCTTTCCCAACCAATGAATCCTCAT

ATCCACCCCAACCTCCAATGAATGACAGACTTCATGTTATTCTTCAAAGGCAAGAAAGGA

TGAATAAGAGTGTGCAAAATTTTGCGGCCACCTTTGGCGAGTTAGTAAATATAATAGCTT

CCCAATATCTGAGCACTCAAAGAACTCCCATGGCTACATGTGGAGAATCAAAAGAAAAGC

AAAGCATGAAGGAGACACTAAAAACTTCGGTGGACAATGAGGAACATGGCTTTGTATTGG

AACAAGTGGAGGAAGCCATAATAGTTGCAGAGGAAGAAGTGGTTGAAGACTTAGGAGATG

CAGAACCTCC

**4. *AhNKEF2***

ACGTGGTGGCTAGCCACTGCTTTCTCCCAGGAAAACTCTCATCTCCGATAAT

GGAAGTGCAACTTTCACAATTCATTCAACAGCATATATATGCGTTTATACATAGCCATAA

TCATGGCCCCGCCGTAACACGGCAATAATCCAGCCATCCGGCTCACGGTTAAATCCATAA

CCAGCCATTTCATCAACAATTACAGCCTTTCGGCCCATGACATAACAAGCACTTCCACCA

CCATCCTCCGCATCTCACATATTCATGCTTGATCCTCATTGATCATCCATTTTTCCCTTG

ATTCACTCGCAAGTTGTCACATTCACTAGCCCTTCTTCTCATAGCTAGACATATCATAAT

GATTTAAGACATAAGTGGTGAGATCGGAGGCTTAGAAGTATGAAATTTGGCTTTTAAAAC

TCAAAAATCAACTTTGGGATGAAAACAGGTCCACGCGTACGCGCACTCCACGCGCACGTA

TGGATGGCCTCGAAAACTCATCGACACGTAAGCGTCATGCACGCTAACGCGTGGATTGAA

AACTAGCCAAACGACGTGCACGCGTCAACCACGCGTACGCGTGGGTACTCTCGTGCCCCA

GGCACAAAACTGGCACAGTTCTGGCATAACTCTCTGGAAAATGGCTGGGCATTGGGTGCA

GCACATCGGCGCGCCCGCGTACATCACGCGCACGCGTGGATGGCGCTTTCGAGAAGAACG

GCGCGTACGCGCCAAGTGCGCCTACGCGCGGGGGGTCATTCTACTAAAAATTTTTCTAAG

TTAAAAGCTGCAGAATTCACAGTTTCAACCCCCAATCTTCCGACGGACATAACTTCCTCA

TTTTAAATCGTTTTTCACCCGTTCTTCGAACGGCAGGGACATCCCGGATCCAATTTTATT

TCTAAACAGATTTGGTACAAAACAGAAATCTGTAGTCCAAGTTATGTCCTATCAAAGTAT

GCCCAAAAACCATGTTTTTCATACAAAACCACAACATGCCATTTTCAAAACAAGCCATAT

TCAACTCTTTTCAAAATCAATCAAAACATGCCATTTTCATCCCTTTTCTTTGAAATCAAT

CAAAATATATCAATTTCAACATCAAGCCTCCTCAACTCACACATTGACACTTTACCACAA

TTTATCAAAATCACTATCTCATCATTTTAACCCACTTTACCCAAGTGGCTCAAACTCAAA

TATATTGACATATCATATACTCTTCCTCATGCCAATTCTCAACAATATCAATTCCACTAA

ATCATCATTGTACACAATCAATATCATACTCACCATCAATATGGTTCAACCCACAATTCA

ACCATAACCAATCATCAAGCATATATCACAACATGCATATTTCTCATACATCATACCATT

AAGGCATCATTAATCATCATCACATATATGACCACATCATATATATCAACAATTCAACAA

CATCAACAATTCAATGCCTATCTTAGGGCCTCTAGCCTAAGTATTTCCTACCATATTACA

TATTAGAT

**5.*AhNKEF3:***

ATATTGCTAAGAATTTTATGAAAAATTCCGAGGAATTATATGAACTATT

TGTATTAGATGCCTATTACCAAGTGTAAGAATATATAAGTGGTTTTTAATTAAAAAATTT

TTAAAATCTCCTTATTGGTAGTGACCTATGTTTAGTTAGCTGGGGAAAAGTGTTAAATGA

TGGTTTGAGGAAAAATAATCTTTTGTTTAGATTTTTGTCTCTTCAAAAGAATTTAGGTAG

CGTTTGAAAAAGAGATAGAGACGGAGAGATAAATTAAAATAAGTTTTAGTATTGTATTTG

GTGTAATATAAAAGTTAGAGATTGAAATAAGAATGAAGTTTTAATTTAATTTATACAAAG

AATAAAATTAAAATTAATTAATTGAAATGACGATATTTTAAGTAAAAAATGTTATTAAAA

TTTTAGTTTTTATCTCCAAAAATTTCAATTGCTTGTGTTTCTATTTTTTAGAGATATTAA

AATACTAAAATGTTAGAAACAAAAATTAAAATTTTAGTACTAATTTTTGAACTAATAAAT

ATGCTACTAAATTTTAATCTCTCAATCCAAATACTTCAAAACAAACGCTACTTTATAACC

AGTGTAGCATCTAGGAGATAAAAAGAATAATACCGTAAAAATACAAGGATATACTATTTT

ATTTTATTTTGAGCGAAGGGAATTTGAAAAAAGTCTTGAAAGTCGTAGAAGTTAGAACCA

TGGTACAATGCAGCATGTGCCACCCATATTAGATTAGATTAGATTCTCACTGCCTCAAAT

GTCCATTACTCTATTATTATTTTTGGGGTCCTACTTCATCCTTCTGCCCCTGCAGAATAT

TTCTTTTTATAATATATGTGGCATTTTAAACTTGAACAAAATGACAATCGATGAAGTAAA

CTTCCATGTATGTATAGCTTTTGAATTGAACTGGGTTCTTAATAGTATCATACGATCGTT

TCCACAAAAAAGGAAAAGGTTCTTAACTAGTTCGATCGATTCATTAAATCTGGTATATTA

TTTACACACTAAAAAATAATAATTAAATTAATTATTATATATTTATGTATAAATATATGT

ATTATTTTACACATTTTCAATATGTACTTTATGCTTGATCATTATTTTTTAGTGTATATG

TAGTATAATTGTAAAATAAACTAAAAGAAAATCTATGTAAAACAAAAAATCCTAAAACAC

TTGTTAAAATATAATAATATTTTTTATTTTTTTATATATTTAATGTATAATTTTTAATTT

TTTATGTTGTTAACTAATGCACTAAAAATACTTATTAGTAAGAAGACTAAGATCCTAAAC

CATCTACTTTTAATGTCATCAAGTGAACTGTTACCAAAGTTAAAGAATTTGAATTGAATT

GAATTGAACAACTCAATTAACTATTTTTACACTACTCAATTGAATCTGTTCTGTTGTTTT

CTCTTGTCCTTTCAAATGAGATACGAGAGGTGTAAAGTTGGAGTAAAATAGGCAAATCAG

TAATGGTAGAA

**6. *AhNKEF4***

ACAAAGGCAAAAAAAATTGCAAGTATGAC

ATATATAAATATATAATGGCCCATATACTAAAACAACTTTTACATTTGAATTGAAGTCAA

TGGTACAGTTCCTTTAGGTAATATTATAACAATGACAATGCAACTGTCTATAAAAGAATG

GCCTATTGATTGGAAAAAAAGGCCTCTACTGTATGTTAGGTATTGAAGATCGGTTTTGCA

ACATGTTCAATTAATAATAATAATAATAATAATAATAATAATAATAATAATAATAAGTGG

AGCACACTTGGCTTTCCTGAATATCAACCTCACATAACTGTCATAGTAAGTAATCAATC

TTAGTGGAAAGAGTTTCAACGTGGAACAACAGAATGAGCTGGCACAAAAATTTACCACCA

AAGGAATTTCCCAAGTTAAACTTTTCCCTCATATTTGAAATTGAGTTTGTAATCATGTTG

TAGACTTGCTAGGGTATTTCTGCAAGGTCCCTCTGCAAATGGGAAAAAGAAATTAGAATC

CTTCTTTAATTCATATCCTCAAATTTGTTCGTTCTGGAGTACTTACCCCCAAAAATAGTA

TGAAGATATTGAAGATCATCAACTGAACAAATATCTATCAAAGCATAGATACCGGGTCTT

AGAGCTTCATCAATTTCTCTGCAAATAATGATCCAATCACCCACATTAGCTAATGTAATG

GTTATGAACAGTGGCTGCAGTCATGAAAATCAGCTCCTCAACCTACTAATTATCATCTCT

TGATACTTTCTAACTGTTTTTATTTTGATATGGATGAAGTTTAACGTAAGTAAAAAAAAA

AAAATAGAAAGGGGGAATTAGTTGTGATAGGAATTGTAATATTGCAAGTAGTAAATCTGA

TATTTTAAAAAAATGGACAAACGAATGAAAACTTCTATACAACCTTCTGATGCCACCTCT

TTTTGGACCATAACCTGTGTAAACCCATATATAGTTGGATAAGAACAGAGAACATTGCCG

GCCGAATATATCTTTTTGCTGTCTTATCTGCATCATGATTATAAAAACAAAATGGGTAAG

CAAAAGGAGAATAACAAGTATAAAGTTTAAAATAATGGAAATAAACAAAATGTTATGTTA

ATGTCCAAAGTTTATTATTTCTATATTCCTTTCCTTCTGCAACCAAAAATAATATAATTT

CATTCAAATTAGTAGTTTAGTTAGATTTCTCATATCATGATCAATGTGAAGCTACAAAGG

AAATCAAACAATTTCAGAATCAGAAAAGACCACTAAATTCACAGTCATCTTCTTAATGAA

CTAGTTCAATGTATTGAAACACACGACATGTTAATTATTAAGCAGGCGACACTAATTTGA

TGCCACGCACCTCTTCAAATACCCTTCGCAGAAAACAAGCACATTTCACTCCCTCATCAA

TTTCCCATGAAAAGCAACCTTTGTTTACTGTTGATTCATTATCCACTACTGTCTCCAAAC

AATTAAGAAGAACAGCAACAGAAGCTTCAAG

**7.*AhNKEF5***

GACAAGTTAGTA

ATACACACGCGCGTGCGTGTGCAAACACACACACACACACACAAAACCCTTAAAACACTA

ACCTGGAAGCACAAACGGAGGGCATCATCTCCTTTGGAATTCTTGAGGCTGGAAAGCCCA

CATAAAATCTCTCGCATGTCAACCGTTCCATCACGGTTGTTGTCGAACAAGTCAAATATG

CGTGGTGCTAGAGGAATTAGTGATGGCATATTTATTGCTTTCAGTACCTCCTCAAACTTA

GAGAGCGTGGCATTGTCCCCATTTCCACATCTGTTAAGTGTTAAACACAGTCATATTAGA

ATTAGATGAGCTTTAATATAGTTGAATGTGTTGAGACATTGAAATGGTACAAGTTGTTTC

ATGTAATGTGGGACAGTTTAAATGGGATTAAAATTAAATTAAAAAAAACTGTTATTTTAT

CTCAATCCCTAATAAAGATAATCTTTAAGGGGATGAGGATTACCAAATACTTGAAAAATG

ATCATGGTTAGATTTTTAAGTAATTGAGAGACTTCTAATACCATACCTTTTTAAGTCCAG

GACTAAACATCAGAAGACTAAAGTTTATAAATGTAGAATTTAATATTACATAAAAAATAA

GTAGCCATAAACCTCAAAAACTAGCTAAATAGGTGAAGTTTATTGAGCACTAATAAATTT

TCAACAATTAATGACTCTAATAAATATTTTTTCGGTGTATATTCTGATTACTTACATCTT

CTTGAAGTGTATCCTAAGATTTTCAATTTCCTCTTCTGTAAGATCATAGGATCCTATCAA

GGATTTCAGTTTCTTAGTTCTCAAGAACACTGTGGTGCTCCATACGCTTGCAATTGCAGC

TGCCCGGAGCTTGCGACGAGCATTGAAGCTTTGCAACCTTGAGACAATCTCAGGGTCCAT

CTGATCATCTTTCGCTACATCACCTATGACCCATGGATGACTCAGGAGCTTATCCAAGAC

ATAGACAAATGAAGCTATGAGTTCTTATTATCTGATTATGTTAATATACTATCTGAAAAC

TTTAATCCGCTTGAAATACGATAAAATTATTGAAAATAATTTAGCACCGAATTTGAGACG

TAATTAGGAACTATGAAGGCATCACAATGCTTTATTGTTGTCTTGTACAGCAGTGAGAAA

TTGTGTATATTATGAAAACCTTAAAATATCAGTTGAGTTGTCTCCATGTGATTTCAAGAT

CATTAGTTCAAGTCATGTAATCAATTACCAATTCTTGCATTAGGTTAGATTACCCTTTGT

TTCTATGGAGTTTTTTGGCAAGCTTCTAATTTGCTAGCAACTCAACAAGACCGTACTTGT

TGAGTTGCCCCTTTATTTAGAGAATAATATATTTTTTAAAAAATATTAAGTACATACTAT

TTAGTTGTGTGTTATTTACAATATATGAAATGAAATGTATTCTTTGAAAGAATGGATGGC

TACTGTTTGTATAGGAACAAGAAGGAAAACAAAATTTTCTTTTTATAT

**8.*AhNKEF6***

CGCACACCCACACCCACACGCGCGTGTGTGTGCAAACACACACACACACACACAAAACCCTTAAAACACTAACCTGGAAGCACAAACGGAGGGCATCATCTCCTTTGGAATTCTTGAGGCTGGAAAGC

CCACATAAAATCTCTCGCATGTCAACCGTTCCATCACGGTTGTTGTCGAACAAGTCAAAT

ATGCGTGGTGCTAGAGGAATTAGTGATGGCATATTTATTGCTTTCAGTACCTCCTCAAAC

TTAGAGAGCGTGGCATTGTCCCCATTTCCACATCTGTTAAGTGTTAAACACAGTCATATT

AGAATTAGATGAGCTTTAATATAGTTGAATGTGTTGAGACATTGAAATGGTACAAGTTGT

TTCATGTAATGTGGGACAATTTAAATGGGATTAAAATTAAATAAAAAAAAAACTGTTATT

TTATCTCAATCCCTATTAAAGATAATCTTTAAGGGCATGAGGATTACCAAATACTTGAAA

AATGATCACGGTTAGATTTTTAAGTAATTGAGAGACTTCTAATACCATACCTTTTTAAGT

CCAGGACTAAACATCAGAAGACTAAAGTTTATAAATGTAGAATTTAATATTACATAAAAA

ATAAGTAGCCATAAACCTCAAAAACTAGTTAAATAGGTGAAGTTTATTGAGCACTAATAA

ATTTTCAACAATTAATGACTCTAGTAAATATTTTTTTCGGTGTATATTCTGATTACTTAC

ATCTTCTTGAAGTGTATCCTAAGATTTTCAATTTCCTCTTCTGTAAGATCATAGGATCCT

ATCAAGGATTTCAGTTTCTTGGTTCTCAAGAACACTGTGGTGCTCCATACGCTTGCAATT

GCAGCTGCCCGGAGCTTGCGACGAGCATTGAAGCTTTGCAACCTTGAGACAATCTCAGGG

TCCATCTGATCATCTTTCGCTACATCACCTATGACCCATGGATGACTCAGGAGCTTATCC

AAGACATAGAGAAATGAAGCTATGAGTTCTTATTATCTGATTATGTTAATATACTATCTG

AAAACTTTAATCCGCTTGAAATATGATAAAATTATTGAAAATAATTTAGCACCGAATTTG

AGACGTAATTAGGACCTATGAAGGCATCACAATGCTTTATTGTTGTCTTGTACAGCAGTG

AGATATTGTGTATATTATGAAAACCTTAAAATATCAGTTGAGTTGTCTCCATGTGATTTC

AAGATCATTAGTTCAAGTCATGTAATCAATTACCAATTCTTGCATTAGGTTAGATTGCCC

TTTGTTTCTATGGAGTTTTTTGGCAAGCTTCTAATTTACTAGCAACTCAACAAGACCGTG

CTTGTTGGGTTGCCCCTTTATTTAGAGAATAATATATTTTTTAAAAAAATCAAGTACATA

CTATTTAGTTGTGTGTTATTTACAATATATGAAATGAAATGTATTCTTTGAAAGAATAGA

TGGCTACTGTTTGTATAGGAACAAGAAGGAAAACAAAATTTTCTTTTTATAT

**9. *AhNNLC1***

TTATGATTTTGTAGGTGAATAATGTTTTTATTTGTGAAAATTGTTATTCATCGTTGATGG

TTTGAATTAAATGTAATGTAAAAGTTCAGCATTAGAGAAAATATTTTTCTGTATTTGCAG

CAAATTTGGGTGTAAAACCAAGATATTTGAGTGTAACACGAAGATATTTGAGTGTATTTT

TCAAGAACTTTCGATGTATGTGTGCTGATAAGTTCTGCATAATTCAAAATTTTTCTTCTC

CCTCCTCTTCATCTTCTGCTGCTGCTTCTCCTTTTTCTTCATCATCATCATCATCATTAT

CATCGTTTTTTTTCTTATTCATCTTTTTTTCTTGTTTTATCTTCTCAAGTTTCTTCTTGT

TTTACTCTCTTAACAAGAATAAAAAAATCAAACAAAAAAGAAGAAGAAACACATAATGGT

ACAAAATTACTTGGAAGAGAATGGACTTACATTCATTCAACTAAAAGAATGAAAGAAATA

AGAAAAAGAAGAAGAAGAAAAAAAATGCAGCATTAGAGGAAATATTTTTCTTTATTTGCA

GTAAATTTGGGTGTAACACGAAGATATTTGAGTGTATTGTTTAAGAATTTTCGGTGTATG

TGTGTTGATAAGTTCTGCATAATTCGAAACTCTTCCTCTTCCTCCTCCTCATCTTCTGTT

GCTTCTTCTTCTTCATCTTCATATTTCATTTTCTCATAATTGTTTATGAATTCAGCTTCG

CAACTTCCTTCACTTTTCGCGACGATTTTCAAAAATTTTTGAGAGATTTTGATCCTTGTT

TAAATTTTGAGCGTTGCGATTTTGATTGAGGAAAAAGAACCGTTTACATTATTCAAGAGT

TAGGAAAATACGCAACCTAAACTGAAAGTCATTTTTGTTGAATTTAGGCCAATTTATATG

AACTTATATACCAAAAAGACTTGTATGCATAGCATATCTCATTCTCATTTTCAACCTAAT

TTCTTGGTTAAGTGTTATATTTCTAATGTAAGGAATCAAAGCTTTCTTGTTTATTTCCAT

AATATTCATGAAATTCACATCCCCCCGTTTAGTATAGTGATTACAAGATTTCTTCTAAAA

TTTCCAATTTTTCCATTGAAGATTTTATTTGTGATGTTATCACACTACAATTTTGTCATA

TATAGTAAATAGAGAGTAAAGAGTATAGCTAGTTTGCTATTTTTACTAGTTTGACATGAA

GTTATTACAGTTAATTAATTAGTTTATTATTTATTTTAATTAATCTCAAATTAATATTAT

TTTTTTCAAATTCAACCCAAAAAAAAGGTTTTGACAGATCAGCCCACCAGTTAGAAAAAA

ATGGGGACGGACTTAAATTGTCAGACAGCCTTTTTGGTGCGCGGACCCTGAAAACCCCCT

TACAAGTTACAACTGGAAATCTAAATCTCTTTCTTTTTTTTTTTTTAATTTTTTTTATGG

TTTTTGAAAAGAAGTGGTCGCTCCCAGTGTAGAGTGTGGGAGCGCGCAAGAGCAGCAACA

A

**10.*AhNNLC2***

TAAGTTCTGCATAATTCAAAACTCTTCTTCTCCCTCCTCTTCATCTTCTGCTGCTGCTTC

TTTTTTTTTTTTATCATCATCATCATCATTATCATCTTTTTTTCTTATTCATCTTTTTTT

CTTGTTTTATCTTCTCAAGTTTCTTCTTGTTTTACTCTCTTAACAAAAATAAAAAAATCA

AATAAAAAAGAAGAAAAAACACATAATGGTACAAAATTACTTGGAAGAGAATGAACTTAC

ATTCATTCAACTAAAAGAAAGACTGAAAGAAAGAAATAAGGAAAAAAAGAAGAAAAAATG

CAGCATTAGAGGAAATATTTTTTTTATTTGCAGTGAATTTGGGTGTAACACGAAAATATT

TGAGTGTATTGTTTAATAATTTTTGATGTATGTGTGCTGATAAGTTCTGCATAATTCGAA

ACTCTTCCTCTTCCTTCTCCTCATTTTCTGCTGCTTCTTCTTCTTCATCTTCTTATTCCA

TTTTCTCATAATTCTTTACAAATTCAGTTTCGCAACTTCTTTCACTTTTCACGACGATTT

TAAAATTTTTTTGAGAGATTTTGATCATTGTTTGAATTTTGAGCATTGCGATTTTAATTG

AAGAAAAAGAACCGTTTACATTATTTAAGAGTTAGAAAAAATGTGTGTTTACACGCAATC

TAAACTTAAGGTCATTTTTGTTAGATTTTGACCAATTTATATAAACTTATATATCCAAAA

AACTTATGTGTAATATATCTCATTCTCATTTTCAACCTAATTTGTTGGTTAAGTGTTATA

TTTCTAATGTAAGGAATCAAAGCTTTCTTGTTTATTTCCATAATATTCATGAAATTCACA

TCACCCCCTTTAGTATAGTAATTACAAGATTTCTTCTAAAATTTCCAATTTTTCCATTGA

AGATTTTATTTGTGATGTTATCAGTTATCACACTACAATTTTGTCATATATAGTAAAATA

GAGAGTAAAGAGTATAGCTAGTTTGCTATTTTTACTAGTTTGACATGAAGTTATTACATT

TAATTAATTAGTTTCTTATTTATTTTATTTTAAAATTTTTTTGAGAGATTTTGATCTTTT

GTTTGAATTTTGAGCATTGTGATTTTGATTGAGGAAAAAGAACCGTTTACATTATTTAAG

CGGACGAGTGAGTTGACCACTCGACCAACCCAAGTTGGTTAGTTTCTTATTTATTTTAAT

TAATCTCAAATTAATATTATTTTGTTTTCAAATTCAATCCAAAAAACCTTTATGACAGAT

TAGCCCACCAGGTAGAAAAAAATGGGGACGGACTTAAATTGTCAGACAGCCTTTTTAGTG

CGAGGACCCTGAAAGCCCCCTTACAAGTTACAACTGGAAATCTAAATCGCTATTTTTTTT

TTCATCGACCAAAAAAAAAAATCTCTCTTTTTTTTTTTATTATTATTCTTATTTTTTTGG

TTTTTGAAAAGAAGTGGTCGCTCCCAGTGTAGAGTGTGGGAGCGCGCAAGAGCAGCAACA

A

**11.*AhNPR1***

CTTGACTTTTTGTTTGATTTTAAAAGTTTTCTATTTCAATCATATCTTTTTCAAAAC

CACCTAACTACTTTTCTCTATCCAAATTTTTGAAAATCACTAACAACTTTTCAAAAATCT

CTTTTTATTTAATTAATTTAATCAGTTTTCAAATTTTATTTCATCTCTTCTCTTAAATGT

CGAACACTAACTAATAATTAAAATAAAAAATACAAATGTTTTCCTTTTATTTTAATTTAA

ATTCTCTCTCTCTCTCTCTCTCTCTCTCTCTCTCTCTCATCTCTTTCTATTTATTTATTT

ATTTACTAACACTTCTTTTCTTCTTATAATTTGAACCCTCTTCCCCTCTCTGTGTTTGAA

TTCTTCATCTTCTCTCTTCTTCACTCTACTCTTCTATTCTTCTACTCACATAAAGGAATC

TCTATACTGTGACATAGAGGATTCCTATTCTTTTCTGTTCTCTTCTTTTTCATATGAGCA

GGAGCAAGGATAAGAACATTCTTGTTGAAGCTGATCCGGAACCTGAAAGGACTCTAAAGA

GGAAGCTAAGAGAAGCTAAAGCACAACACTCTGGAGAGGACCTGACAGAATTTTTCGAAA

AAGAAAAAGAGATGGCCGAACCCAATAACAATGGTGGAGATGCAAGGAAGATGCTTGGTG

ACTTTATTGCACCCTCTTCTGACTTCTGTGGAAGGAGCATCTCAATTCCTGTAATTGGAG

CAAACAACTTTGAGCTTAAGCCTCAATTAGTTTCTCTAATGCAGCAGAATTGTAAGTTTT

ATGGACTTCTATTGGAAGATCCTCATCAGTTCTTAGCTAAATTCTTGCAAATCTGTGATA

CTATTAAGACCAATGGGGTTAATACTAAGGTCTACAGACTTAAGTTTTTCCCCTTTGCTG

TAAAAGACAGAGCTAGGACATGGTTGGACTCACAACCTAAAGATAGCCTGAACTCTTGGG

AAAAGTTGGTCAATACTTTCTTGGCCAAATTCTTTCCACCTCAAAAGTTGAGCAAGCTTA

GAGTGGAAGTCCAAACCTTCAGACAGAAGGAAGGTGAATCCCTCTATGAAGCTTGAGAAA

GATACAAGCAATTGATCAGAATGTGTCCTTCTGACATATTTTCAGAATGGAGCATCATAT

GTATATTCTATGATGGTCTGTCTGAATTATCCAAGATGTCATTAGATCATTCTGCAGGAG

GATCTCTTCATCTGAAGAAGACGACTGCATAAGCCCAGAAACTCATTGAAATGGTTGCAA

ATAACCAATTCATGTATACTTCTGAAAGGAATCCTGTGAATAATGGGACAACTCAGAAGA

AAGGAGTTCTTGAGATTGATACTATGAATGCAATATTGGCTCAGAACAAAATATTGACTC

AGCAAGTCAATATGATTTCTCAGAGTCTGTTTGGAATGCAAGCTGCATCAAGCAGTACTA

AGGAGGCTTCCTCTGAAGAAGAAGCCTATGACCCTGAGAATCCTGCAATGGCAGAAGTGA

ATT

**12.*AhNPR2***

AAAGAAGGAAAAGTAATAGTGTATGTATGTAATTGTAAACAGATTTTGAAACAGAGAAT

CAGATATGGGATAGGGGAAACATCATGTTGTGGCTGCCAATGCCCATGCCATTTGCCTTT

TTAATCAAACCACCTACCAAAGCATATCCCCTTTTTCGTTCATTTTCATGTTTGATTTAT

TTATTAATATCTTTTGACATCACATCTTGTCATATACGTGATTATGTATTGAATTTAAGA

TATTATGAAAATTCAACTTAATCTAAGAATAAAACATACAGATTTAATATTAAAAATAGA

TGTTAAAAAAATACATAAAAGTAATACATATAACATATTCTTAAATATTAAAAATTTAAA

AAGATAAAAATACAAAAAAAGATGTTTCTATTACTGCTATATTTTAAAAAATTAAAAAAG

TATATATTTTTCGATCTCTAATTTCAAATGCAAATTTTACTTTTTACAATAAAATTTTTT

TTGAAAGCCAATAATTTTTAGAATTTTTAATTGATTAACATAAATATTAAATTATTTTTA

ATAAATAAATTTAACTAATTTATATATGTAAATTCTGAAAAATATAAATACAAACTATAT

TGATTTATGTGTATAAAATTCAGATAAATATAAATATAAATTATATATTTCTTATGTGTA

AAATATTTATAAATATAAATATAAATTATTACTAATTAAATATTAACAAAAAATGATAAT

ATTTACTGAACACTTAGCATTGTACTTTCCAATATTGTATCTCATGATATACTAAGCAAT

TATAAGCATAACATATCCCATCTATTACATGCATTGGCACCTGCCAAGTGATTTGTTTTC

ACTTTTCAGGTGGCTCAACTGCCACATAAGACGGCGCTGCCACAGAGAGAAGAGCAGTGA

TGGATCTTAGCTCATATAGGTGCTGCTTACTTGCAATGATTATATACCTCACCACATTAG

TAGTGTCTTCATAAAATGGCTGCATGAGCCATCATTCAAATCCTTGAATCCACACACCAC

TATAAAAGGGTCAACTAGGATACCCTTCTTTCTCTCAACTCGCATAATAAAAAAGAAACT

AAGTCCCTTCTTCCCTCCAATGGCATCAAAGTTCTTCTTCACCTCTCTCACTTTGCTGGT

TCTTTTCTGCTTGCTTCTTGTGAAAGACACTTCTGGTTTGTAAATAATCTTTTTCTTCTT

GTTTCTGTCACAAACTTTGGAAGAAGAAAAAAAGAAGGTATCATTCATAACATGTTTTGT

TTAATGGCAGATTATTGCAATGCTAATAAGCATGGATGCTTTGATGCTCAGGCAGCTGTT

CTAAAGGAGATTCGAAATAGAAAGGTCTATCACTACTTATACTTTAGATTCTATACTTTT

GTTTGTGGTACAATTTATAACACTATTTCTACGTTGTTAATTGGTGCAGGTGTTTTTTGC

TTTCAAGGATAAGAAAACAAGTCTGAAGGCTATGCTGCAAGGATCATCATCATCAAGAAT

G

**13.*AhNPR3***

GTTAGGGCGTAGAGTGAAAGTTACTTGGATACCATCTATGTGATTTTTACATGGATGTAG

AGCTTTTTCCTTTTCTATTAACGGCGTAATAATATGGTTTGATGACGTAGACCGTTAGTG

ACACGTGTTACTTTATAGTTTGACCACGTATAATGGTATGATAATGTATTGATCAATGAC

ACGTGACATACTAACATACTAACCTAGACGGTTGTACCACGTGTCCAAAATGCAATTAGT

GTAATTACAATCTCACAAACAATTTAATGTATGACACCAATCTCAAAAATCACTGTAACT

CATTATTTATGCTAATAACATATATCAAAATAAATTCTTACTTTATACACAAGACATACA

AATAACTTCTTCTAAGTTATACAGCAACATGATACAATAATGTCATTTTCTTTATTTCAG

TGTCTATTCAGTAATATCTTGCATAAGTTTCTCCTTGTCACATCTCACATATAACTCATA

GCAGTGGCTTTTAGAACCATGATCGTCTAATTATTATAGCATGAAAAAGAAAACAATTAT

ATAATAATGAAAAAAATTGGCACTTTGGTCATTGTAATTTGTAAATAATGAAAATAATAA

AGAGAAAAAGACAAGTCCCTTTGTTTATTGGCACATAAAAGACAAGTACTGAGGACCAAA

CTCTTTGGCAGCAATTTGTGGCAACAATGGAATTCTCTTTGACTCTTGTTGTTATTACTA

ATATTTTTTTTTCTCTCTAAAATTTCAAGAAAATTCTAGAAATTTTTATTAATATTCAGT

TCGATTAAATTCCAAAAATTAATTTTGACAGTTCTACTTGTACTTGACTCCAGTACTAAA

ATCTTAAACTAAGAATATATCCAATTTAATTTTAATGCACTATTATTATAAAGAAATTAG

TAAAAATAATTAATTTTTTATTTATTATTAAATAATATTTTATAGTTGAATAATTTTGTA

AATGACTATTAGGATATTAAAATTCAGCTCTCTCTATATACTTTAATGTTAATGAATGAG

TTAGTTATATTACTTTATTATTAAAATAGCATAGAATTAGATAATACCATTCACACACCT

AATTAATATTATTATATGAAATATTCATTGAATTTAAGAAAAAAGATAAAAGAAAAAATA

GTACAAATTATCATTTAATCATGAGATTAAGGCATTAACAATGTAAAATTTTTTACAATA

CTTGAATCACATCTGTTTTATTTAGATAATTATTTTTAAGTAATTATAAAAAAATATTTT

TTTTTCTACCATGAATGCAAACAAATGGCATAGCAATGGTGAAAAAAAAAAAATTCTGAT

ACATATTCCAATTTCTCTGTCTTCTTCTGAAGTTTTCATATAGCCATATTTCTCTGTATT

TTGCTGTTCCCACCCCTTCTCTCTCTCTCTCTCTCTTCACCATAATCACAAAGTTCTAAT

CTTTATCACTCTGCAACTCCAAATTCTCCTTCAAAACAATCCCTTTTCTCTTCTTTGAGA

A

**14. *AhNPR4***

ATGCTAATAACATATATCAAAATAAATTCTTACTTTATACACAAGACTTAGAAATAACTT

CTACTAAGTTATATGAAAATGGATTCTCTCTATTTTTTTTATTAGAGAGAATAAAGTGTG

ATTTTTCACCTTTAATTTTATAAATGGGATCAGAAATTAATAAAAAAGAGAAAATAATAA

ATATAAGATCAAATACTAGACACTATCTAATTTTTTTTTCACTCCCAAATTATACAACAA

CATGATACAATAATGTCATTTTCTTTATTTCAGTGTCTATTCAGTAATATCTTGCATAAG

TTTCTCCTTGTCACATCTCACATATAACTCATAGCAGTGGCTTTTAGAACCATGATCGTC

TAATTATTATAGCATGAAAAAGAAAACAATTATATAATAATGAAAAATTGCCACTTTGGT

CATTGTAATTTGTAAATAATGAAAATAATAAAGAGAAAAAGACAAGTCCCTTTGTTTATT

GGCACATAAAAGACAAGTACTGAGGACCAAACTCTTTGGCAGCAATTTGTGGCAACAATG

GAATTCTCTTTGACTCTTGTTGTTATTACTATTTTTTTTTTCTCTCTAAAATTTCAAGAA

AATTCTAGAATTTTTTTATTAATATTCAGTTCGATTAAATTCCAAAAATTAATTTTGACA

GTTCTACTATGTACTTGACTCCAGTACTAAAATCTTAAACTAAGAATATATCCAATTTAA

TTTTAATGCACTATTATTATAAATAAAGTACACACATATATATTAGTAAAAATAATTAAT

TTTTTATTTATTATTAAATAATATTTTATAGTTGAATAATTTTGTAAATGACTATTAGGA

TATTAAAATTCAACTCTCTCTATATACTTTAATGTTAATGAATGAGTTAGTTATATTACT

TTATTATTAAAATAGCATAGAGTTAGATAATACCATTCACACATCTAATTAATATTAGGT

GGAAACTCATGTATAGTTAACTTTATGTGAAGTTGATATCTAAGAGTCGTTAAATGAAAA

TTAGTCAAATCAGTTAAACTATCTAACAGTTCTGAGATATCAACGTCACATAAAGTCGAT

TTCACTTTAATATTATTATATGAAATATTCATTGAATTTAAAAAAAAAGATAAAAGAAAA

AATAGTACAAATAATCATTTAATCATGAAATTAAGGCATTAACAATGTAAAATTTTTTAC

AATAGTTAAATCACATCTGTTTTATTTAGATAATTATTTGTAAGTAATTATAAAAAATAT

TTTTTTTCTACCATGAATGCAAACAAATGGCATAGCAATGGTGAAAAAAAAAAAAAACTC

TTTGATACATATTCCAATTTCTCTGTCTTCTTCTGAAGTTTTCACATAGCCATATTTCTC

TGTACTTCGCTGTTCCCACCCCTTCTCTCTCTCTCTCTTCACCATAATCACAAAGTTCTA

ATCTTTATCACTCTGCAACTCCAAATTCTCCTTCAAAACAATCCCTTTTCTCTTCTTTGA

G

**15. *AhNPR5***

AACAAGTTCTCAACCTCGCTTTATGGAAAATGAAGGAGGGGATACAAGTAGTTGTGTGGT

TTCTGTGTTCATTTCGGTGGTTCTGCCTTGTTGGTAGTTGCATGCATAGCCATTTCAGGT

TATCCTTCTTTTATTCCTACTGCTCTCAATTTTTTATGAATGAACAAGACAAATTAGTAA

CTAGTAAATGGAATGGAAAGGCTATAATTAACAGAAATTGTCCTCCAACAATCAACATAA

GCAACTTCAAATTATCCTATAAATATAATCAAATCTGAAATATTTCACATAATAAAATAA

TCAATTATTTACGAATGTACACCACTGCCAATGTATATAAGGTGCTTAATTTGTTCCAAG

CATGTCATTATTAGAGATATAACCATTAATATTACCTTCTCCTATAAATTTTTTTGGAGA

AAAAATACTAATTAGAATTGAAAAGGAATTCTATTATTAAGTTATTAACAATATTATAAG

CATAAAATACGTATACGTGTTTATATCTGCATTAAGAGACTGAATATCTTTCTTCTTTTC

GTTTTGTTTTTTTATCAAAATATCAAATACAAAGACGCATGTTAGAACAGTTGATGTAGC

TGCCGATGTGGCTCTGTTGCATACCAATCATTTTGTTAAAGTCATAATCAGTGAAAAAAT

ATTTATAATGACAACAAAAAGGAAGACAAGGAGGAAAACACCCAAGCCATCTACTTCTCT

CTTCTCTCCTCTCTCCTCCGTATGTGGATAAGGCTCCCACTATTTTAAATAGAAGCCAAA

TATTGTCATTTATGATGTAATGCAATTATTTTGTTCGTGGATGGAAAATTTTGTATCAAA

TAATAATGGATTCATTTTGTACCTTACATTACTCTTGTTATTACTATCTTAGCAACAAAT

AACTATCAAGGAAAGATCTATGAAAACGAAACAGATTTCATTGGGTATGGATTTTGAAGT

CACTTTCACATCTAAATTTGAAGAACCAACCACAAAATATAATCACTCTTTAAATTTCTT

TCTATCTATTATATTCAATATAATCTTGAATTATTGTTTGTCTAATCAATAATCATATAT

AATCTTACTTATTTACAATGACATTTAAACGAAAAAAGAAAAATAAAGTATCGGTAAAAT

TAGTTTGAGTAATGACTTTCTGGAACAAAATGAATATCGATATGAAACTTTAGGTAAAAA

AGTTAACCATAATAAATGTGTTACCAAAGAATCCTAGCTTTATCTCATGATAAATAAATG

GTAACTTACACTTGGGGTCATACATACCAAGGATTGCTAATCCTTTCATCTAAGTTTCTT

ACAAATTGAGATCATCTTAACCAAAATCTCCCTTTTTTTTTTCTTTTTTGTCCTACCTTG

CTAAGATCACAAGAACCTAAACATTTGAGTACAAACTTGAGCTATATACAGCTACTACTC

ACTACCCTGAGCTCTCGCATGAGCTTCCAAGATTGGAAGTTATTTCATGCACCAAAACTC

T

**16. *AhNPR6***

AAGTTCTCAACCTCGCTTTATGGAAAATGAAGGAGGGGATACAAGTAGTTGTGTGGTTTC

TGTGTTCATTTCGGTGGTTCTGCCTTGTTGGTAGTTGCATGCATAGCCATTTCAGGTTAT

CCTTCTTTTATTCCTACTGCTCTCAATTTTTTATGAATGAACAAGACAAATTAGTAACTA

GTAAATGGAATGGAAAGGCTATAATTAACAGAAATTGTCCTCCAACAATCAACATAAGCA

ACTTCAAATTATCCTATAAATATAATCAAATCTGAAATATTTCACATAATAAAATAATCA

ATTATTTACGAATGTACACCACTGCCAATGTATATAAGGTGCTTAATTTGTTCCAAGCAT

GTCATTATTAGAGATATAACCATTAATATTACCTTCTCCTATAAATTTTTTTGGAGAAAA

AATACTAATTAGAATTGAAAAGGAATTCTATTATTAAGTTATTAACAATATTATAAGCAT

AAAATACGTATACGTGTTTATATCTGCATTAAGAGACTGAATATCTTTCTTCTTTTCGTT

TTGTTTTTTTATCAAAATATCAAATACAAAGACGCATGTTAGAACAGTTGATGTAGCTGC

CGATGTGGCTCTGTTGCATACCAATCATTTTGTTAAAGTCATAATCAGTGAAAAAATATT

TATAATGACAACAAAAAGGAAGACAAGGAGGAAAACACCCAAGCCATCTACTTCTCTCTT

CTCTCCTCTCTCCTCCGTATGTGGATAAGGCTCCCACTATTTTAAATAGAAGCCAAATAT

TGTCATTTATGATGTAATGCAATTATTTTGTTCGTGGATGGAAAATTTTGTATCAAATAA

TAATGGATTCATTTTGTACCTTACATTACTCTTGTTATTACTATCTTAGCAACAAATAAC

TATCAAGGAAAGATCTATGAAAACGAAACAGATTTCATTGGGTATGGATTTTGAAGTCAC

TTTCACATCTAAATTTGAAGAACCAACCACAAAATATAATCACTCTTTAAATTTCTTTCT

ATCTATTATATTCAATATAATCTTGAATTATTGTTTGTCTAATCAATAATCATATATAAT

CTTACTTATTTACAATGACATTTAAACGAAAAAAGAAAAATAAAGTATCGGTAAAATTAG

TTTGAGTAATGACTTTCTGGAACAAAATGAATATCGATATGAAACTTTAGGTAAAAAAGT

TAACCATAATAAATGTGTTACCAAAGAATCCTAGCTTTATCTCATGATAAATAAATGGTA

ACTTACACTTGGGGTCATACATACCAAGGATTGCTAATCCTTTCATCTAAGTTTCTTACA

AATTGAGATCATCTTAACCAAAATCTCCCTTTTTTTTTTCTTTTTTGTCCTACCTTGCTA

AGATCACAAGAACCTAAACATTTGAGTACAAACTTGAGCTATATACAGCTACTACTCACT

ACCCTGAGCTCTCGCATGAGCTTCCAAGATTGGAAGTTATTTCATGCACCAAAACTCTTA

T

**17. *AhNKLM1***

AATAATGTGCTTCTTTAATTTAAAAAACTCTGTATAATAAGCTGATAACAATGATCAATC

ATGAGAGACAAAGATGGAAACGTCACTCACCAAATAACTAAAAGGAACTATTCCATTAAC

TGATAATGTTTTATTCTTATTTAAGTGTTCCTCAAAATAGATTAGTTAGCATAAATGAAA

AATAATAACCAAAAGAAACCCAATCTGATAACGTTTTGGATCAGAAATATATGTCTGTTT

ATTTATTGAGTCCTGCTAGGGAGCCAAGCTCTTGACTTTTCGGATCTAGAGCTCTGATAC

CATATCATGATACCACTCATCTCAAAAACTTCAGCTGATAGAAAAAGATAACACTAATGG

TTATATCTCCATTGGCTCCCTATACTTTTTTATGTTATATTTTAAGGAATAATAAGCGAC

CACATGTAACGATTCCACTGGTTTTTTTCTCCGCCCCTAATAATACCTTAGGGAAACATT

AAAAAAATAATGATGTGCATATAGATCTAGGCATCTCCAGGTTTATTCCCTTGGCTTGAG

TATGCTTAATAAAATAAAGTATTTCGAAACACACGACATACATGATAGGAAGTTAGAAAG

AATAAAGATAATTTTACGAGTGAAATTGATTACCTTCTGTAAGATCTTGAGCTCCTCATA

GGCATTCCACTTCATCTTCTTGATTGCATAGGTTTCTCCATCAATAGTACCTTTGTAAAC

AGAACCTTGAATGAGGCAACTGTCACTGAAGGCATCAGTGGCTTCTACAAGTTCCTCAAT

TCCAAATACTCTGTACTTGTCCAAGCAGTCAGACACATTGGCCATCAGCTTCACATCCAT

CGGTTTACTCCCTTTCCCATCTCCCCCTCCCGAAAACACCCTGCCCTCCTTCTGCCTCTG

CTCCTCCTCGTCCCTGCCCGCCCACATCCTGCCCTTCAACACACTCTCCCTATACATCCA

CACCGCACACACCACCATTAGCAGCAAACCAGCAATCCCCAATCCAATTGCCAGCCCTCT

CACGGTGCCTGTTGTTATGAGTATGCTTAGTGGATTATTCAAAAACCATGACATTCACTA

AGCAATTCTAGTTTTTAGTAAATACTCACGTACAGTTATCTTCACGTGAAGTTGATAGCC

GAGAACAATTACATTGACTAAATTGTTAAACAGTTCTCAACTATCAACTTCATATGATTT

TAGGTGAGTTTTAAATGTCTAGTGGTTATTTTCAATATATTGAAACAAAAAATATTTAGT

AAATTGTTTAGCGAGGTTGTTACCTGCCCTGTCATCAGAGCTGCCGGCCGGAGTCGGAGA

AGGAGCAGCAGCAGTGCTTGTATTCGGCTGTGACAGAACCGGCAGCTTCGTCACCGGGAC

GAATATGGTATCATAGACGTTGAATTTGTTCCCGTTGACATCAGTGATGGCCTTCTGCTG

AGATCCAAACCTGGAAGCAATCGAAGATAGGTTGTCGGACGGTTGGACAACATAGGAGAT

C

**18. *AhNKLM2***

CTTAGAGGAGGGGAAAATTTCAACCTTTCGGAAATTCGTAACCTTCAAATACAGGAGAGG

TGATTTAACTAATAAGAATGGGCATGGAGTATTATATACAAGAATTAGGGCTCTATCTAT

CAAATATTGTTTGTTATGTATGTATCCATGACTATATATTTTCGGTCAAATGAATTGTCT

TAGTTCAAACTTAGAAAAAGTAGTATTAGAATAACGTGTATGATCAATATAAACCTAATG

AAATAACAAAACATGAATTATTTGGAGGCTGGAGTGCTCCTTGGGTTTTTTACTTTTCTA

GATTCTTTTATCGAAGAAATTTTCAGGTTTCAACTTTGAAGTGACTGGTCTATAAGTGAG

CAGTGTTAACAGTGACACTATTTAGGCGATAGGCACAAATTAATAATCAACTTAAAGTGT

GAATTACGGAAATTATTGCTATCCAGGACATAGAAATTTCTTGCAATCAATGTTCATAGT

CCAATTTTTGCAATAACTTGTGACTTTGTTAAGATAGAAATAAATAATGACCTCTCCAAT

ACGTTTAGTTTGCATTTAGTAGTCAACTCTCAAGTCTCTATTGGGAACCAAATCTGACAC

GACTTTCTTGTGTAGTATATGCGTTCCTCGTACTCTCATCTTTTGTGTTTCATTTTCACA

CCAAAGGGCAGGACCAATAATATTTTCATAAATTTTATTGCTTTATTCTAAACTAGACCC

TATAGCCAATCACCAATCGTTTTAATTTTATTTGCCCAAACCAAACGTTACAATGATGGT

TGAATAAAGTAATCAATCTATTAACCAACTCAGTTGGTCGCTAATTCACTCGTTTTGCTT

TGGTAAATGATAAATGATAAATTTTTAAATAAAAATTAAATTCAAATAGATTAATTTTTA

ACATATTAGACTAAAAGATATCTTTAAAAGCCATAAAAAAATAATCAATCCATTTGATAT

GATTAATTACTTAATTGTCTACAAAAAATGCTCAACAGTCATTTAGCTGTGTATTATAAT

TATTAGTGATCCAATAACCTTCAAAGTTTCTATGTACTATTGATATTAAATATTAGGTAA

TTTTTATTGTATTTAAAAAATAGTTTTAATTTGCTAAAAAAATATAAAAAATTAAATTAA

AAAATTTAAATACTATTTAATAAAAACCATGGAATTAGTCTAGAAGTATACCAGTGATTG

GAGTAATTGAAAACAGAAAAAGAATGGGCCCCATCTTAAGTCACTCATAGAAAATTTAAT

CATGTTGACTAGTATTCATAGTTGGGGGATAAATATCAAGAACGGTGAGTGGGAAACTTA

GACATAAGCAAGCAGGTTTGACTTGAATGAATGCTGTCGAGTTAGATCTGAAAGGAAACT

AAGTCCATTGTTTCTTTTCTTCTTTTCCTTGCCTCTAACCGCAGCCACAGCACTTGAAAT

TCTACACTATATATAATAAACAAACATGTCAAAATAGAAACAAACAGAGAGAGAAGCCAC

A

**19. *AhNKLM3***

ATTTTTCGAACCTTGGGGATATAAAAACAGTGCAGAGACTCTTTGCAATGGTTAGGCAGT

GTGGTGTGGAGCCAGATGCTTATCTCTATAAGGTGCTTATCCAAGGTTACTGCAAGTCAA

AAAGAGCTGCACTAGCATGGAGGCTATTTGAAGATATGAGGAATTCAGGTCTGATGCCTG

ATTCTGCCACAAAAGAATTGCTAGTGAAAAGCCTCTGGAAAGAAGGGAGACGAAGAGAAG

CTGCAGCTATTGAAGAGAGTTGTGAGGAAGTAAATGTGGTACTTCCGCATGCATTGCGTG

GTCATGAATGGACTGTCAGCTCTGCTGATCTCTCAAAAGTTTATAATCTTTATTCCGATT

GTTTTCCTTCAAATGGTGGCTAGAGTTATAGACGGGGTCTGATATGATAGATTTTTGGTT

TCTTCGTTTATCAAACTTCAGCACACACTCTAACAGGTATGAAACATACAGAATCCCTTA

ACTTGGGATTTGAAGGGAGGAGGTGGTAAGGGATGCAAAATATTGTTTAAAATTTCAGTT

TTCAGTAAATATTTGTGTTCTGCAAACTTGTTTTGACCAGAGCATCAGTTAATATTCAAA

AGTGAGCCCTTGTTGTACTGGCTAGTGTAGGTTTCTTTGTATGTAATTACTTATTAGTCA

AGTGCCTGAAATTAACTTTGCTTAATCATCATTAATGAATGACTAGTTGATATGTGGAGA

ACCACATTCAAACATGGTTGCTGACTTTTAAATTTGTAGTACATTATTTGTTGCAATTTT

TATTGACGTTGATTATCATGTTGCAAGTTTCTTTTGCTTATCTTTCTCTAAACAGTAGTA

GGTAGATTACAAAATCTCTTATTCTATTGTTTTCGATTAGATAGATGGAATCACGTATAT

GAACCGACACCGTGAAGCATGAAAGCCGCACTCAAATATTCAAGAAGCTATTTCAAAAAA

TATGAAACCAGGAAAAAAATGCAAGTGTAAAATAATGCTTATGAAAAATTGTATGCAGTG

TAGGTAGTAATTCTTATAAAATTCATTTGACCCCAATCCTTTTAGCACGTATAAATCTTG

TGGAGACACTACATTTTAAGGCGTGGTCTAGTGATAGATAGACATGGAATTGGCATCAGA

ATTTTCTCTTATGATATCTCAAATTGGAGATTCTTTGCAGAAAAGTTAAAAATGACCATC

CTGGAATCTTCATTGGCATTTTATTATACTCTGCTGTGCGCATGAATAGCATACTATTGA

TATATGTAAAGTCAATGTTTTCCTTAACATATAATCTTAGACTTACTTGGGAATTTGATA

TCACTGCATAAATAATTAGGAATAGATATGGTAAAACAATATACCAATAACTCTTCTGTT

AAACCAAAGGCTATTTTATTGACAAAGAATCCTCCATATCACCAAAAATCATGAGTGTCT

TTGACTTCTGATAATAAAAGTTTCCTTTATGTTCTTTCCCTCCTACTCAACTTCAGAAAA

A

**20. *AhNNup1***

GGTTGAAAGAGG

CCTTGGTTGATTGCTCTTGAAGTTTGGAGAAGAACCAAAGTGAACCAATTGAACCGGGTT

GGAGTTTTGCAAAAGATGGACCAAAAGTTTGAGCAAAAGTTAGGGGCTAACTTTTGCCCA

AACTTTTCATATCAGCACCCATATCCAGTTGATACCAACGTTGGTGCCAAAGTTAGGGGT

CTAACTTTGGCTTCAACGTTGGCCTTACCTTGTGCACTTGTGGCGCCAACGTTAGCCACC

AAGTTAGGGGCTAACGTTGGCGCAAACTTTTGCTCCTCCCCTTGTGATTTTCATGTGCCA

ACGTTAGCCACCAAGTTAGGGGCTAACGTTGGCGCAAACTTTTGGTGCCCAGGGAGGTTT

TCTTCATGCCAACGTTAGCCTCAAAGTTAGGGGCTAACGTTGGCGCAAACTTTTGGTGCC

CAGGGGTGATTTTCATGTTCCAACGTTAGCCCAAAAGTTAGGGGCTAACGTTGGGGCTAA

CTTTTCACCCAAAAGTGTGTGCAAAAGTTTGAGGCTAACTTTAGGTCCAGCTTTTTGCTT

CCTGGTTCAATTTCACTTATTCCATTGTCTTCTCTTTACTCCTAGCTATTCCTTCTTGCT

TCAACCTTTCTCCAAGCTTTCTTCACCTATCATTAATCAACCAAACACATCAAAGCTATG

CTTAAAATCATGAGATATTCATTCTTTCATAATATGTAACAATTATAGTATAAAACGTCA

TGAAATAGCATGAATTCATACATGGTTGATTAAATCAAAGGAAACATGAAAATCTACCCA

ATTAGCTTGCTTATGGCTCAAGAAAGTGCATAATTCTAATGAAAACAAAAGAAAAAGACT

AGTTAAAATTGGCTAAGATGACTTGTCATCACAACACCAAACTTAAAGCTTGCTTGTCCT

CAAGCAAGAAATAAATTTATGCTCCAAGGTTCTTTCAATTAAGATGGATTGAAGAATGAC

TTGTAAAGTCCAGTGAGTGAAGTGATCAAGTACAGTGGGGTGAACTCTAAATCATGTGCT

CTTGCAAGGGCTTCAGTGCTCACTAGTCCTCACATATTGGGAGTCTTAGGTCATAGGATT

TTCATCCAAATGGTATCATGGAGATCTCTTTATATGTAGTCACCTTGAAGCAGCTTATAA

TTTCAGTGCTTTGGCCTCGACTCTAAGTGTCATGTCTCAAAGCGGCTCTTTAGATAAGCT

TTCAATCAATACTCCTAAACCAGTTGGTTTTAAGGTATTAGGTGTTGAAGCACCCCTAAG

GATTTACTTGCTCAAGCCTCTTTCCTTGACACAGCTCAACCACAAGCATTTACTAGGCTA

ACAACTCTTTGATTTTTGTTTCTTCTTTCTTTTTCTGCCTAGTAATTGATGCTCAGAGCC

TTGGGCCATGTTCTTTTTGCTTTTGTATTTTCTTTATTTTCTTTTGTTTTGTTTGCTGCT

TCTTGGATCAATAGATTTTTGAGAATCTCTACAATACTTCTTTGAACT

**21. *AhNNup2***

TTGAAAGAGG

CCTTGGTTGATTGCTCTTGAAGTTTGGAGAAGAACCAAAGTGAACCAATTGAACCGGGTT

GGAGTTTTGCAAAAGATGGACCAAAAGTTTGAGCAAAAGTTAGGGGCTAACTTTTGCCCA

AACTTTTCATATCAGCACCCATATCCAGTTGATACCAACGTTGGTGCCAAAGTTAGGGGT

CTAACTTTGGCTTCAACGTTGGCCTTACCTTGTGCACTTGTGGCGCCAACGTTAGCCACC

AAGTTAGGGGCTAACGTTGGCGCAAACTTTTGCTCCTCCCCTTGTGATTTTCATGTGCCA

ACGTTAGCCACCAAGTTAGGGGCTAACGTTGGCGCAAACTTTTGGTGCCCAGGGAGGTTT

TCTTCATGCCAACGTTAGCCTCAAAGTTAGGGGCTAACGTTGGCGCAAACTTTTGGTGCC

CAGGGGTGATTTTCATGTTCCAACGTTAGCCCAAAAGTTAGGGGCTAACGTTGGGGCTAA

CTTTTCACCCAAAAGTGTGTGCAAAAGTTTGAGGCTAACTTTAGGTCCAGCTTTTTGCTT

CCTGGTTCAATTTCACTTATTCCATTGTCTTCTCTTTACTCCTAGCTATTCCTTCTTGCT

TCAACCTTTCTCCAAGCTTTCTTCACCTATCATTAATCAACCAAACACATCAAAGCTATG

CTTAAAATCATGAGATATTCATTCTTTCATAATATGTAACAATTATAGTATAAAACGTCA

TGAAATAGCATGAATTCATACATGGTTGATTAAATCAAAGGAAACATGAAAATCTACCCA

ATTAGCTTGCTTATGGCTCAAGAAAGTGCATAATTCTAATGAAAACAAAAGAAAAAGACT

AGTTAAAATTGGCTAAGATGACTTGTCATCACAACACCAAACTTAAAGCTTGCTTGTCCT

CAAGCAAGAAATAAATTTATGCTCCAAGGTTCTTTCAATTAAGATGGATTGAAGAATGAC

TTGTAAAGTCCAGTGAGTGAAGTGATCAAGTACAGTGGGGTGAACTCTAAATCATGTGCT

CTTGCAAGGGCTTCAGTGCTCACTAGTCCTCACATATTGGGAGTCTTAGGTCATAGGATT

TTCATCCAAATGGTATCATGGAGATCTCTTTATATGTAGTCACCTTGAAGCAGCTTATAA

TTTCAGTGCTTTGGCCTCGACTCTAAGTGTCATGTCTCAAAGCGGCTCTTTAGATAAGCT

TTCAATCAATACTCCTAAACCAGTTGGTTTTAAGGTATTAGGTGTTGAAGCACCCCTAAG

GATTTACTTGCTCAAGCCTCTTTCCTTGACACAGCTCAACCACAAGCATTTACTAGGCTA

ACAACTCTTTGATTTTTGTTTCTTCTTTCTTTTTCTGCCTAGTAATTGATGCTCAGAGCC

TTGGGCCATGTTCTTTTTGCTTTTGTATTTTCTTTATTTTCTTTTGTTTTGTTTGCTGCT

TCTTGGATCAATAGATTTTTGAGAATCTCTACAATACTTCTTTGAACTTC

**22. *AhNNup3***

CAATTTCATGGTATGATTTGGATCTAGCAGGAGGCAATGCAGTGTATGGTGTTGATTTCT

TGAGATAAATGAGAATTCCAATGTATTTGGTGGTTCTATGCCAAATCTTGATGTACCTGC

TGCTAGTTATGAGTAAGCTAGCACCAAGATTATTAATGTTGGCACCAACAACAACATCAT

CATCACCAGACATGCTTGCAGTTAGTTAGTTGGTTGGTAGTTTTGTAAGGTAACTCAGGA

TCAAAATAGCAATAAGAATGAATGATTGAACAAATGGTTCCTTGTTCATTTATGGAGAGA

AAAAGCTAGGCTATGATATAAATGATAGTGATGATTTCTAAATAAAGAGAGCAGGAAGAA

TATCTGAAGAATCTAAGATCCTATTTTTCAATGCACAAACCAAATCATTTTGGCTAGTTG

AGTATTGATTACTGAGTAGGAAAACAAGAGAATGGTCCTATATTTATATACAAAATAAGT

GTATAAATGCTATTTTTTATTTTTATTTAAAAAGTTTGAAATTGCAATAGAATTTACAAG

GTAGACATTAAACTCTTCGCATGTGTTTGATTTTTAGTTTTTTACTAATATTTTATTCGG

GCACTTCTAGATACCTGCCACGTGTCCACGTTTACGCGGGGCGGCTACTGCACTCACAAT

CACAAAACACTACACTAACTGACTTTTGCGGGCAGTGCATTGCCGGCCAGTGGAGTTTGG

AAATTTGGCCCAAATTTCCTCATACATCTTTCCTTTACCTAACTTTTATTATTACTTTTT

TCAATGAATAATAATTAATACCATGAGACTTGAGCTATATTATGGATTTACGGCATGCAA

AACACTGGAAAAGTCGAATTGATAATTCAAAATTAATAAAAGAGCAAGTGAGCAATCCGG

GTATGGTGTGGTTTATAAAAATGACTAATTTTCTCCCAATTAAATATTGATGGGTTGATT

TTTATTGACATGAAAAACTTAAAATTTTGAGCTACTTGTTAACTTCTACTTATAGTATAT

TCATTAGTTAAAAAATGAGCTACAGTATACTACTATACTGTAATGTACTAATGTGTCAGT

TTTATCAGTTAGGCAGTTACTATATGTGTCTTTGTGATACAACGAAGTCTGTGTAACTGT

GTTCTGAAGGGTGTGCCCGTAAGAAAAATAAATTTAAAAAAACAACAGCATTAGATTTAG

ATCAACCAAAAAAAATGAAAAAGATGTCGCCTAACCCAACCTAATTCAATTAGCGTCGAG

CCATTTTCATTATACAATTTTTACAAATAAAAAAAAAAGCACACGCAACGTTTTGTTCCC

GCAAAACAAGTTGTAAGGAGCAGCAATGGTAAGTAGAAATGAAGTAAAAGAAACATTTCT

CTTTTAATATTGGCTCACGACTCCTATCATTACCTATGAAATGCAATGTAATGGTTAAAG

GGGGAACGAAACAAAAGCTGCATTTCACATGTCATGCTACCTACACTAGATTGTAATACA

C

**23. *AhNNup4***

CACCAAGATTATTAATGTTGGCACCAACAACAACATCATCATCACCAGACATGCTTGCAG

TTAGTTAGTTGGTAGTTTTGTAAGGTAACTGAGGATCAGAATAGGAACAAGAATGAATGG

TTGGTTCCTTGTTCATTTATGGAGAGAAAAGGCTAAATAAAGAGAGCATGAAGAATATCT

GAAGAATCTAAGATCCTATCTTTCAATGCACAAACCAAATCATTTTGGTTAGTTGAGTAT

TGATTACTGAGTAGGAAAACAAGAGAATGGTCCTATATTTATATATAAAAATTAAGTGTA

TAAATGCTATTTTTTTATTTTTACTTAAAAAGCTTGAAATTGCAATAGAATTTACAAGGT

AGACATTAAACTCTACGCATGGGTTTGATTTTTAGTTTTTTTACTAATATTTTATTCGGG

CACTTCTAGATAGCTGCCACGTGTCCACCTTTACGCGGGGCGGCTGGAAAAGTACTGCAC

TCACAATCACAAAATACTACACTAACTGACTTTTGCGGGCAGTGCATTGCCGGCCAGTGG

AGTTTGGAAATTTGGCCCAAATTTCCTCATACATGATACATCTTTCCTTTACCTAACTTT

TATTATTTCTTCCTCATAATAATTAATATCATGAGACTTGAGCTATATTATGGGTTTACG

GCATGCGAAACACTGGAAAGTCGAATTGATAATTCAAAATTAATAAAAGAGCGAGTGAGC

AATCCGGGTATGGTGTGGTTTATAAAATTGACTAATTTTGTCCCAATTAAATATTGATGG

CTTGATTTTTATTGACATGAAAAACTTAAAATTTTGAGCTAGTTGTTAACTTCTACTTAT

AGTATAGGAAAGTTATCAGGTGTACCCGAGAATATCCATGTTCTAGTTGTTTTAATCGTT

AATTTTAATTAATATATATTATATATATTTTTTATAATTCAAATTAACAGTTAAAATAAT

TAGAACACTGATGTTCTCGATACACTTGAAACTCTTCCTATATTCATTAGTTAAAAAATG

AGCTACAGTATACTACTATACTGTAATGTACTAATGTGTCAGTTTTATCAGTTAGACAGT

TACTATATGTGTCTTTGTGATACAACGAAGTCTGTGTAACTGTGTTCTGAAGGGTGTGCC

CGTAAGAAAAATAAATTTAAAAAACAACGGCATTAGATCAACCAAAAAAAATGAAAAAGA

TGTCGCCTAACCCAACCTAATTCAATTAGCGTCGAGCCATTTTCATTATACAATTTTTAC

ATCAAAAAAAAAAAAGCACACGCAACGTTTTGTTCCCGCAAAACAAGTTGTAAGGAGCAG

CAATGGTAAGTAGAAATGAAGTAAAAGAAACATTTCTCTTTTAATATTGGCTCACGACTC

CTATGAGGACCATTATCGTTATCAGCCATTGTGAATGTGAAATGCAATGTAATGGTTAAA

GGGGAACGAAACAAAAGCTGCATTTCACATGTCATGCTACCTACACTAGATTGTAATACA

C

**24. *AhNKTyr***

ATTGAAGAGAGTTGTGAGGAAGTAAATGTGGTACTTCCGCATGCATTGCGTGGTCATGAA

TGGACTGTCAGCTCTGCTGATCTCTCGAAAGTTTATAATCTTTATTCCGATTGTTTTGCT

TCAAATGGTGGCTAGAGTTATAGACGGGGTCTGATATGATAGATTTTTGGTTTCTTCGTT

TATCAAACTTCAGCACACACTCTAACAGGTATGAAACATACAGAATCCCTTAACTTGGGA

TTTGAAGGGAGGAGGTGGTAAGGGATGCAAAATATTGTTTAAAATTTCAGTTTTCAGTAA

ATATTTGTGTTCTGCAAACTTGTTTTGACCAGAGCATCAGTTAATATTCAAAAGTGAGCC

CTTGTTGTACTGGCTAGTGTAGGTTTCTTTGTATGTAATTACTTATTAGTCAAGTGCCTG

AAATTAACTTTGCTTAATCATAATTAATGAATGACTAGTTGATATGTGGAGAACCACATT

CAAACACGGTTGCTGACTTTTAAATTTGTAGTACATTATTTGTTGCAATTTTTATTGACG

TTGATTATCATGTTGCAAGTTTCTTTTGCTTATCTTTCTCTTAACAGTAGTAGCTAGATT

ACAGAATCTCTTATTCTATTGTTTTCGATTAGATTAGATGGAATCACGTATATGAACCGA

CACCGTGAAGCATGAAAGCCGCACTCAAATATTCAAGAAGGCTATTTCAAAAATATGAAA

CCAGGGAAAAAATGCAAGTGTAAAATAATGCTTATGAAAAATTGTATGCAGTGTAGGTAG

TAATTCTTATAAAATTCATTAGACCCGAATACTTTTAGCACGTATAAATCTTGTGGAGAC

ACTACATTTTAAGGCGTGGTCTAGTGATAGATAGACATGGAATTGGCATCAGAATTTTCT

CTTATGATGTCTCAAATTGGAGATTCTTTGCATATAAGTTAAAAATGACCATCCTGGAAT

CTTCATTGGCATTTTATTATACTCTGCTGTGCGCATGAATAGCATACTATTGATATATGT

AAAGTCAATGTTTTCCTTAACATATAATCTTAGACTTACTTGGAAATTTGATATCACTGC

ATAAATAATTAGGAATAGATATGGTAAAACAGTATACCAATAACTCTTCTGTTAAACCAA

AGGGTATTTTATTGACAAAGAATCCTCCATATCACCAAAAATCCTGAGTGTCTTTGACTT

CTGATAATAAAAGTTTCCTTTATGTTCTTTCCCTCCTTCTCAACTTCAGAAAAATGGCTT

TCTTTCTACCCTCTCTCTCAAGTAGTATTTTTCTTGTATTCATGTTCTCCATCACCAGCA

TCCCAACTCAATCACAACAGGTTAATGGAACAGACTTTTCATGCCCAGTGGATTCACCTT

CTTCCTGTGGAACATATGTGACATACATCGCTAAATCTCCAAACTTCTTGAGCCTTTCTA

ACATATCTGACATATTTGACACCAGCCCTTTATCCATTGCAAGAGCAAGTAACATAAAGA

A

**25. *AhNSur1***

TTCTATATCAAACTCTTGCAGAAGCAACACCCATCTTATGAGCCTGGGTTTTGAATCCTA

CTTTGTAAGTAGATATTTAAGAGCAGCATGGTCAGTGTACACAATCACCTTTGATCCTAC

TAAGTATGATTTAAACTTGTCAATGGCATAAACCACTGTAAGCAATTCTTTTTCTGTGGT

TGTGTAATTTTTCTGGGCATCATTTAAAACACGGCTAGCATAATAAATGACATGCAAAAG

CTTGTCATGCCTCTGCCCCAATACTGCACCAATGGCATGGTCACTGGCATCACACATTAG

TTCGAATGGTAATGTCCAGTCTGGTGCAGAAATAACTGGTGCTGTGACCAGCTTAGCTTT

CAGAGTTTCAAACGCCTGCAGACACTCTGTGTCAAACATAAATGGCATGTCAGCAGCTAG

CAGGTTGCTCAGAGGTTTTGCAATTTTTGAAAAATCCTTTATAAACCTCCTATAGAATCC

TGCATGCCCTAGAAAGCTTCTGATTGCCTTAACATTGACAGGTGGTGGTAATTTTTTAAT

TACTTCAACTTTAGCTTGATCCACCTCTATTCCCTTGTTTGAAATTTTATATCCAAGGAT

AATCCCTTCAGTCACCATAATAACGTATGATCGTTCGGAGGAGTGATGGATGATATCTTA

CATTTTTATGGCGTCAACTTGGATCAAAATTTTGAAGACTTAAAGGGTTATTCCCTCGCT

TTTGTAATCTAGAGGAATTAGGAGATGTCATATATATATATATATATATATATATATGAC

GTAAACTGATGTGCGATGTAAATATGTGAAAGTTCCTGCTTTATTTGTGATTATCTTTGA

TCTTATTTATGAATGTTCATGCTTATCAAACCGAATGCATTTTCAAAAAATTATCCTGTA

AAACAACTGCGCTTTTAACAATGAATCAAGCTCATATAATAAATATTAGTTAATAAATAT

GGAAGCAAGTTAGTAACGCCTAACTTCTAGTATGATCATGACATACTAAAAATTGGGTCA

TTATATTTCTCCAATATTTTTATTGTGTTTTCAAAGTTTAGAATAATTTAATTTTGATCC

AAATATTTTCAAAGTGCTATAATTTTATTAATGTGTTAAAATTATTAAGGATCAATATTT

AATCAATCTTTTTAAACATTCCCTTGATTTATTATGCATACACCTTCGTCCAACCCAACA

GCAACAGGATTGCAGTGGTGTCATGATCACTGAAAAGCAAACACCTTTTTTTAAATAGAA

AATTCCATGTTTCTTAATTCTTATACATTTAATTGAATAAAAATTACATTAGTGTAATTA

CTATGTCAATACATTTTTAATGCGATGACAATTCACGACAAAGAAGCTACTTAATTACAT

AGATCAAATCTATTCATGCACTCTATAAACTTCTCTCATATATTTGTTTGAGATTTTCAC

TATTTTTTTTAATTTTAAAACATAGAGAGAGTAAATTAACATTAGTAATACTCAATAATT

T

**26. *AhNSur2***

ACTAGGTTCATCGTGAATGCATTGTCGAGCTTGTCTCAAAAGAAGTCCTCGCTTAATGAT

GACATAATGACAATGAGGATTGAGATGCTTGATGATACAACAAAGGTGTTCCTTTTACTG

ATCTTCCGGAGAATTCCGTGCCTTCAGAAAAAATCTCGCGTACTCGAAAAACGGGGTTGT

TACAACAGAGCTGCCATTGAGGGAAGATGAGATATTGTAATGTGCATAGCTCTGGAAAAA

CCATTTCCGAAATTTGTGCTGAGTAATGTCGGGAGCGGATAGACAACCGACACATGAGCT

CATGACCTACAATAGAACCAGGGGTGCATCATACTTGTTTGCCACATCTTTTTTATGTGC

ATTCTGTATATGTGTGTGTTTTCTTTATTGTATTCTATTTTCTATATTCTCTGGTTGTTA

CTATTTTCTTGTAGTTCCTGTTTATTTGCTATTTCCTATATTTTTATATTCAAGTACTAT

TATTTGGTAATAATTAAAGAATTATCGAACTTAACTAACAACTTCGACCTACTAAGAATC

CCAAGTTCTTACTCTTTTTCTCCCCTTTAAATGGAGCACCGGCAGTCAAGTAATAACGTA

TGATCGTTTGGAGGAGTGATAGATGATATCTTACATATTGATGGCATCAACTTGGATCAA

AATTTTGAAGACTTAAGTGTTATTTCCTTGCTTTTGTAATCTAGAGGAATTAAGAGATGT

CATATATATATATATATATATATATATATAGCTAGCTACTTTTACGGATGTCATAACTAA

GTGATATAAACTAATGTGCGATGTAAATATGTGAAAGTTCCTGCTTTATTTGTGATTATA

TTTGATCTTATTTATGAACGTTCCTGCTTATCAAACCAAATGCATTTTCAAAAAATTATC

CAGTAAAACAATTGCACTTTAACAATGAATCAAGCTCATATAATAAATATTAGTAATAAA

TATGGAAGCAAGTTAGTAACGCCTAACCTCTAGTATGATCATAACATACTAAAAATTGGG

TTGTTACATTTCACCAATATTTTTATTGTGTTTTCAAAGTTTAGAATAATTTAATTTTGA

TCCAAATATTTTTAAAGATCTATAATTTTATTAATGTGTTAAAATTATCACGGATCAATA

TTTAATCAATCTTTTTAAACATTTCCTTGATTTATTATGCATACACCTTCGTCCAATCCA

ACAGCAACGGGATTGCGATGGTGTCATGATCACTAAAAAGAAAATACCTTTTTTTAATAG

AAAACACCATGTTTCTTAATTCTTATACATTTTATTGAATAAAAATTACATTAGTGTAAT

TACTATGTCAATAAATTTTTAATGTAATGACAATTCACAACAAAGAAGTTACTTAATTAC

ACAGATCAAATATATTCATGCACTCTATAAACTTCTCTCATCTTTGTTTGAAATTTTTAC

TATTTTTTTGAATTTTAAAACATAGAAAGGGTAAATTAACATTAGTAGTACTCAATAATT

T

**27. *AhNSur3***

TTATACAATATTATTTTGTTTTAAAATAGTTTATTTTAATATAAATATAATATAACATTT

GTTAAATTTATTTTTTAAAAATAAAATTTTATTATTTTAATATATAAAATTTATAAATTT

ATATATACTAATATTTTATCAGATTGAAAGTCACTTTGAACCAGATTAAAATAGACAAAT

TATTATTTTAAAACCGAATTCAAACCTACATAAACCCGATCCAACACGACCATGAACACC

CCTCCATTAACAATTTAACATGGATTGGCCCATTCCTCTAAATGTAAGATCTGTAAATGT

CACTTTGTTCAAAAAAAAAAATCTGTAAATGTCACTATTATTAGAGTATCTAGCTCCATG

TCTCCATGTTTACCACCCAATACGGCAATACCCATCCATGCTAGATTAAATGAAACTAAG

AGAAGCCAACCACTCCCATCTTGCGCCACGTGCAACAGTTCCACACACAATTAATTCTGT

TCGATTACATCAGAATTTTCCAAATGATTTTGGTTTTTGGACTTAGTAGATTTAGCTTCA

TTCCTAGAAGGAATCTTGTTCACTAATCACTATCCATAGCATGCTCGTTTCAGTTTCACA

TATGATGTGACGACGTCTCACTCATCAAATTTGCATGGCCTATAAAAATCTCTCTGGATT

GTCAAAATTTAAACGTTGCCGTCTTGTGTTGTGCCTCTCTTGAAGGTGAGTCAGTGAAAC

CATTCCATCTTCTTCTTTCATCACTGTTTTTATCCATGGCTATTTCACTTGTTCCTTGCA

ATTTTGTTGATTTGTGTGTGCGCGCGCGTGTTCGTAAACAGTAAAATTTTGCCCTTTTCC

GTGCATTATATGCTAATCAAAGCGTTTTCTATTAATAGTTTATTGCCTTATGTTTTGTAA

TATTTGCTATGTACTTTTTCCCAATTTTTATCAAATTTCTCTTGTTTGTGTTGCTGACAA

GAGCCATGTTTGGTCACGCCTCGTTTTTTCTAATCTCTGTTTAGCCGGTTATAGTATCTC

TCTTGATTGATCCCCGTTTCTAATTTTTGGGATTGAAAATCCCTGTACCGAACCGTATTT

CTGGCTCGAGAGTCGAGACACTTGCCAAACACAATCTTTGTGGAGTAAATGGATCCATTG

AAAGAGTTTAGAGTGGACGAGTATACACAAAAAGATTGGTAAATACTAAAGTACACGGAA

AGATTGTTTTGATGGGCAAAAGTGTATTAGTGGGTTTGTAAGAGGTAAGAAGTAATTTTG

TCCATCAAAACAATCTTTAGATGTACTTTGATATTTATGAATTTTGACGTGTATTTTTGT

CTACTTCAAATTCTTTCACGTGTACTTTTGTCTCTTTATTCATCTTTGTATGAACATAGT

GTATTCTATATAATCTGTTTCTGTTTTGTAACTGGTTTAATTTATAGTTAGTTGTTTCAT

TTGGTAATTAATAATTAATAATAGGGAATTAGGGATTTTGATGACAGGTTAACTATCAAC

A

**28. *AhNSur4***

GAAAACCATATTACTTAAAACAAAGAATAACAATCATGTATAGGTTCAAACTTTAT

TAGTCTTCTTTTCTTCAATGTGTTTTACAAAAGCATATTCTCATTCTGTCCACGTCGTTT

TGGGAAAAAAATTGCTAATTTGTCCTGTAATAATATTATTTGGATTGAATCCTAATTACC

AAATTTATATATCCTACGTATGTAACTTACAATTATTTACTCACAGCAATAACCAAATTA

ATACACAATCCTAATATACCATGATATCTATTCTTACGCATACTCACACACAAGTTCTTA

TATGTATGATTGAGTGTGTTTTGAAGGTAGAGAAAAGCCTTTAAAGTCATCCACAAGCAG

TTAGAGTAATTCTTACTCGCTAATATGGAATTCATGTCGATAATTCTAGTTTGGCTATGA

ATTTTAACCCCACAATGCAAAGGATTTAATCCTATTGGTTCTGGTCTGCTACTATGAGTA

GATTTTTAGCTAAATTAGTCAGTCCTATACACTTTTTTTTCTTCATTCCCATTAATTGTG

TTTGGTTGAAAAAGGTAAGGCATGACAATGAGAAATGGCCCCCCAAAATGAAAAATAATA

ATAGAAACAAAAAAAATGGTTGATTTATAATACTTTAACCATATTCAGATGTAGCTAAAG

TATAGTCCATTATTACATGAATTAATGCATATGTTCCTAAACTTCATCTTAAAAGGTGTT

GCCTAAGAATGTTTAGTATTAAACAAGACTTTAACAGAAAATGAATTGCATTCATAAACA

CCAATACAAAAAACCAAGAAAGAAGAGAATGCGAGTAAATTAAATACATTAAATTAAAAT

CAATTGAAAAAAATAAAGTCACTTTAGAAAGAACAGCAAAAAAGAATTTATATAAAATTA

TAAGATAATACGTCAAAGAAGAGAAATCCTCTAAACTTAAAAGGAGGAATAAAGCGAAGA

AATTATTACAATGAAGTTTACAAATACTACTATAACAATTAATTAGCAATGCATAACAGC

TTTAATACCATCTCCTACACTAAAAAGTTCATCGTATTACTCATGTGCCAAATTGCTTTG

GACCAACTAAAAGTAATTACAGAAATCAAATTAAAATTGAAGATATCATTTCCTCACTCA

CTGAAAACTAATAGGCTAATAGCCCCTTTCTAATTCAAGTTGTAAACCCCGAAAAATTAG

CGAATAATTAATCAATAAATTAATTTTAAATAAAAAAATTAGAAATATAAATTTTATAGT

AAAATAAGATAGAGCTAAGTAAAATAAGAAATTTGACACTAATTTCAAAGAATTCGGCCC

AAAATTGGGCCGAACGGACCAAATCGGTCAAACCGGGCCTATGGGCCCAACCAATCCACT

CATTTAAGTGAGCTTCAGCTCACTTATCCCTCCTATGCATGCAAAGAACGCTGAATTGTA

AGCCTTAGAACTTGGTGGAAGTTGAGTGCTTGCGCTTTGGTGATTTGGGCTTGAGGGGCT

ATGT

**29. *AhNSur5***

ATATGTTAATC

TATGGAAATATCAATAATATATTTATAAATTAGGAAAACATGTTAAGGAAGACAAATAAA

ATATTAAACCATGTGGAGCTTGAATAAGACATATGAAGTTGAGATTCACATAACATGTCA

AAAAGACAAGTACCTTGTTGGAGATTCAATTTTTGATATTTGAAAAAACAATGGAAGGTG

AATTTGTATTGATATGAAATTTTTGTATGTCTACTGCTTTTATAGCAGAATCTAATGACA

ATTTTGTTGAATTTTCAATATTAGATGCCTATTTTGGTGGGCTTTCTAACTATGTGTTCC

ATTTGTTAAGAAACATTGTTTGTTATTAGTTCCAATTAATGTAACAATAGTTTTTATTTT

TTTTGTATACATTAGGTGACAACTTAATTCCAACTCAACTATGAATTCATCTTAGTTGCA

AAATTGTTTAGATAATAAAATATTTTTTTAGATTTTGTCAAATTTGTTTTTTAACCTTAT

CCTCTTCTTATGCGACTCATTTGATATTTTGGCACAAAATAAATCCACATGAATTTATGT

TATAGTTCAAACTAACACAAAATTTTAAAATAAAAGACTTAATAAAGTATACAAATTATT

TAGAAAATTAAATACGATACTGTCTTGGTATTTCGTATATTTAATACTTCTTTTATAATC

TATTAATTTGATAGATGGATACCCTCTTTCTATTTTCTTTCTTTTTTTTTTTTGTAAAAA

ATGGCCTACTTAACTCAGTACTTAGAGCATTGCTTTTATACGGCAATAGTTCAGCAAAAT

TATAATGTGAAATTTAAATTTTTTTATTTATGATAAAAAAAATTTTCTTAAGTTACAAAT

AACTTTAATAGAATTTTTATTTATTTTGTTTGGAGCTTTATGAAGTACAATTTCCTCCAA

TAGGGCATCTAAAAAAAGCAGGTTTTACTTTTACTAATTAAAAATTCTAAACAATTTTTA

ATAGATTTTTTGTGTCATTAATCGAAAATTTATATATCATTTTTGTTATTATTAAACTAA

TTGTATGATATTGAACTAATAAGAAGAAGATGATGACGACGATAGTGATGATAATGAAGG

AAGAGGAGGAAGAAAAAAAAAGAGGAGGAAGAGGAGGAAGAAAGAGGATCGGAGGAGGTG

GTGGTAAAGGAGGAGATGGTGATGATGATAATGTTGATGATCATGATCATGATAATGATA

ATAAAGGTGAAGCAGGAGGAGGATGAGGACGAAGAACAATGAGAACGAAAACGATAACGA

TAACATAAACGCTCGTATATAAATTTGACTTGGTTGAACTTAGTTAAAAAAAAGACTTGA

CTGTCTAGGATTTTGATCTGTAAAATTTTCGCACTAAAATATAATATTTTAACTTAACTA

TTGCATATTTTTTCTTACTTAATTATTGCTTATTTTAACTCCTATTATAAGTGAAAACTA

CTTGATTAATTTAAATAATAATATGAAATATATTATTATTATTATTGTA

**30. *AhNSur6***

AAATATTTTAAAAAAATTAAAACAAAAAACTCTTACTTATTATAAAAGCTGATACTA

ACTCTCTCCACAATGAGTTTAAGCCATCTTTTCTTTGCTAATGATGCCACGTCAGCAATT

CTAATTACTTGGCAAAAGATGAAAACTAACCAATCACTATTTGGTAAAGTGTTTTAAAAA

AATTAAAACAAAAAACTCTTATCTATTATTATTGAATTGAAACATCAAGTACTAATTAAA

TGCAATAAACATACATTAAAAGTGTCATAATAAAAATAATTATAAAAAATTTCAGTTACA

AATATTCAAATTTTACAACTTATACATATTAAAACTCTTACTACTCTTCATTTCTTAATC

TCTCATACTAATTGTCAAAATTCCTTAAATTTAATATAACATTTTTTGTAATAGCTCAAA

AAAAAAAAAAACAAATTTGTGGGCCACTCTTCCTTTACTTTTTCCCTAACCCTAATCACA

CAAAAACACTCTCTCTCAACCCTCAGTCCCTCACTAGCCGTCGAGCTCCCACCCCCGCTC

CTCCCACACCCTCTTCGTAGAAACACACACTCACGGACGCACTCCCCTCGCTCACCCTCA

CGCCGTCGCTCATCCCCCCACACACGCACCTACACTGACGCGGAGAAGAGAGGGAGGTCG

CTGCTGCTGTTCATGCCCATCGTTGCCAACTGCACCGTCGCGGATGAGCGTCTCTGTCGT

CGCGAGCTGCAACCGCCGCCATTGAAGCCACCCTCGCCGTTGTCCTTGAGCGCCAGTGAG

AGGGAGAGCTCGAGGAAGAGAGGAACGTTGTCCCGCCATGCACCGTCGTCACTGGAGCTG

AACCGCCGCCGAAGGCCCGCCGCCGTCCGTGGAGAGTCGTCGCCGTCTGGGTCGCTGCCG

GAGGAAGAGAGCTTGCACAAGGGGTTGCTGGAGTGGCTCGCTACTGCCGCAAGAGCCACT

GCACCTCTGGCCACTGGGAAGTTACATTGAGGTCGCCGTTCTTCTGCCGCCGCCGCTGTC

GGGGTTTCTGGTCAATGGGTATGACGCTGTTGTTGCCGGAACCACCACCGGTGCTGCCGC

TACTTGTTTCCCTTAAGACCGAGTGGTTGTAGTTGCTGAGAAAGTGATTTGGAGCTGAGG

TTGTGGCTGCCGCAATTTCGGGTTGAGAGGAAAGGTTCCGTCGGCGCGTTTGGATTATAG

TTTTAACAATCGAGAACTATAGCCAGCTCTATCTTGTCGCTACTCCGGTTCAAATTCTAC

GCTCATTCGTTCCATTCCACTTTAATCCTCCTTACCTTGAATTTGGCACTCTGGGTTTGG

TATCTTCGGTCCCTAGGGACTACGTTGTGAGTAGGCTTTACATTTATAATTGTTTGATTG

TGCATCTATAATTATTTTGACATATATCTATTATGATCTTTGAATTATACTCACTATATT

TGTCTAGAATGGTTTTAAATTGATTAGAATAATTGATTTATTAAAATTAAATAATTCATT

TTT

**31. *AhNSur7***

TAAAAATTATTTCGTTTACGGTGTAAACAAAATATATGTATT

TATAAATATAAAAATATATTTTTAATGTTTGATCTATTTTAAATTATTAATATTTTTATA

TTTAGAAATACATATATCTCATTTACACGATAAACGAGATATATAGAAATTAAAAAAATT

CATATATCTCGTTTATAGTGTAAACGAGATAATAATAGATGACGTATTTTTATAATAATT

TTTTAAATTATTTATTTTAGTAAATAAAATATTTATTTAATTTATATAAATAAAAAATCC

TAGTGTGTGATTTTTTATAACTTTAATTTTGAGTGAAAGTGACTTTAACTTAACTTTACA

TAATTGTATGTATATATTCCAATTGATGTGTTATTTTTTTTTTTAGTGAGTGCGACTTTA

ACTTTACGATAATTGTGTTTATATAATTAAATTGATGTGTTATTTTCCCCCCAAAAATAC

TGTATGGTAAACACTAAACAATCGATCATTAAATTATTTACTAGATTGTGTTTTAACATG

TATTATTTGATATTTATATTGTGTTATTTATATGTACTTTTGATATATATGTAATTAAGT

ATGTTTTCATTACAAAAGTTTTCAAATAATTATATATTAAATTTTTTTTGTATACAAAGG

ATTTTTTAGACAAAACAATAAATTATACATAAATTTATTTGTACTTTTATTTTTGAGTAA

ACAATAAATAAATATCTGACTTTTTTATCCCAAAGTCTCGATTAATTAAAAAAACAAAAA

TATTTATCATTTCTTTAAAACGTAAGACATCTAAATTTTTTTAAGAGATCAAGTTATTTT

TATATATTTTAAACAAAAAATTTAAATATTTTATATTTTAAAAAATCAAAAATATTTTTA

TATTTTTAATTAGAGAAAAACTTGCCTATCTTCTAATTAGAAAATTATGGAACTATTTCT

CTGTTTTCTCTAAATATTTTATCCATTTGGGTGACATTTGTGATTTCAGACGTGAGGAAG

TTTCTCTCCTCAAATATTTTGCCTATAAATCGTGGTTGCTGCTTCCGCATTCAATTGCAC

TCTGCCTTGTGGTGTGGCACTCTTTGAATTGAATGTAAGCAAATGAAACCATTCTATCCC

TGTTTCCTCCTTCATTATGCATGCTCTTCCCTGTCTCTTTTCTTTTTCATGACCACCCTA

CTTCCTCTCAGATTATTGCTGCTGTCTGTTTAGCTTGTTCCATGTCAATAATGTCAATTT

GGTGGAATTTATAAGTTTATTTTTGTGTTAATAAACACTGAACTTTTTTCCCCTTTTCTA

TACATAAGAAGCTAATCATTGTTTCTCCTATCGCCATAATGTTTTTTTATCAGTTTATGT

CATATTTCTTTTCAGCTTTCTATGAATTTTGCATCATGATTAATAATTGCCTCAGAAACA

TACATAAATATATGCAAAATATCTCTATCTTAATTTATAATTAGCTGATTAGTGAACAAT

AATAAGAATTTTGATGAC

**32. *AhNSur8***

TATGGTTTTTGAAATCAAGTTT

TGAAATCGTTTTGAAGATAATGGAACTTCATAAATACACCCAAATGATTACAGAAATACA

CCCCCAAACGGTTACAAAAATACATCCAAAAAATTATAGAAATACACCAAAAGCATTACA

AAAATACACCCAAGAATTTAAAAAATATACCCAAAATTCGTACACTTTATGCATAATTCA

GAACTCTTCCTCTTTCTCTTCCTCATCTTTTGCTGCTTCTTTTTCTTCTTTATTTTTTTA

TTTCATATTCTCATAATTCTTTTTGGGAGGAAAAAATTAAATAAAAAAAATACATAATGT

TAAAAAATCAAAAGAAGAACGAGGAGAAACACGACAATAAAAATAAAACACTTCAAAAAT

AAGGAAAAAGAAGACGAAGAAGAAGAGGCACGAAAAAAGAAGAAGAATGAGAAGAAGAAA

AAGATGAGACATGAAAAAAAAAATAAAATAAATGACTTATATGACTTGTATGAGAAAATG

CTTGTATGCGAAAAATTTTTCATAATTAAATTGACGTGTTATTTTTTCCCCAAAAATGCT

ATATACGGTAAACACTAAACAATCGATCATTAAATCATTTACTAGATTGTGTTTTAACAA

GTATAATTTCATATTTATATTGTTATTTATATGTATTTTTGATATATATGTAATTAAGTA

TGTTGTCATTACAAAAGTTTACAAATAATTATATATTGAATTTTTTTGTATACAAAGGAT

TTTTTAAACAAAAACAATAAATTATACATAAATTTATTTATACTTTTATTTTGAATAAAC

CACAAATATTCTGACTTTTTTATTTCAATGATAAATAAGTGTTCGACTAATTAAAAAACT

AAAAATATCTATTACCTTTTAAAACATGAGACATTTAAATTCTTTTGAGAAATTAATTTA

TCTTTATATATTTTAAACAAAAAAATATCATATTTTAAAAAATTAAAAATATTCTTATAT

TTTTAATTAGACAATCTCTTCTAATTAAAAAGTTATGTAACTATTTCTCTTCTTGTCTAA

TTTTTTTATCCATTTGGGTGACATTTGTGATTTCAGACGTGAGGATGTTTCTCTCCTCAA

ATATTTTGCCTATAAATCCTGGTTGCTGGTTCCGCATTCAATTACACTCTGCCTTGTGGT

GTGGTGTGGCACTCTTGAATTGAATGTAAGCCAATGAAACCATTCTATCCCTGTTTCCTT

CTTCATTATGCATGCTCTTCCCTCTTTCTTTTCTTTCTCAAATTATTGCTGCTATTCATG

CCTTTTATTCATGGCTGCCTATTGCTGTTTAGCTTGTTCCATGTCAATAATGTCAATTTG

GTGGAATTTTTAAGTTTATTTTTGTGTTAATAAACACTGAATTTTTTCCCCTTTTCTATA

CATAAGAAGCTAATCATTGTTTCTCCCCCCTTAATCAATGTTTTTTTATCAGTTTATGTC

ATATTTCTTTTCAGCTTTTTGTGAATTTTGCATCATGA

**33. *AhNSur9***

GTTTTCCTCAGCTGGGTCAGACGCGCCACGACGACATTCTCCTCGGCTACAACAGGCGCG

CCACGATAGCATTCTCCTCAAGTTGGGTCACAGGTATGCGATGAAGGTGTTGTAGTCACC

TCAATAGTGAGAGGGAGAAGCAGTGAGAGGGGAAGAGATTGGCGGTAATGGATTAGATAG

GTGGCGGACGGCAATGAGGAGGTAGTAGGAGTGTTTCTGTGACTCTGTGAGTATTTTTGA

GGGATAAGTGAGGAGAGAGGAAGAAGAGAAGAGAGGGAGTGACGGTAGAATGGGGTTAGG

GTGGGGTAGTTTAGGAATTTTATTAAAAAATCAACAAAAAATTAGGATTTGGATACTTTT

GTTATACGGAACCCATCTTTTATGGGTACAGCGTTAATTTCTTTAGTTTATAGGTACTTT

TGTCAGCATTCGTAAACTTTATGGATATTTTTGGTAGTTTTTCCACCGAAATTATTTAAT

ACAACAGTATAAATATGATACCAAGACAAAACCTATGAGAGTACCTCTTTTGTTTTTCAG

CAAAAAATAGATATTACGTAATTTAATTGCACTAGAAACAAACACTAAAAAATATACTAT

AAAACTCTCTTTGTAGCAATAATAAAGGATTTGTATTTATTTTAAATTTTTTAAAAAAAT

GGAACAATGGTAAATAACAATACACAATGGTGATGTGAGAGGACATTGACCAAATGGATA

AAAATAAGGAATAATTATTACAAATAGATTCTTTTGAATGATAAAAAAAGATATATTTGC

AATTCACAAGTAAAAACACCCATATAGAAGCAACCTCTCCTTTAATCATATATATATATA

TATATACACGCATACTTTCTCTTGGTGTTGGTCTATAAATTCGTTGTTGTATTGATTTTC

CTTCTTTCATATTAATAACTAATCATTATATTCTTTTGGAAGGGGAAACTATTTGAGGTA

CGCTTCTTCATTCCATCTTCTATCTTATTAACAATTCACTTGAGCATGCCCCTTTCGAAT

TTCAGTTATAGAATACCTACTACTCTATATGCACGTTGACTTTATAATCAAACCATTGCT

AGCTTTCTCTTTGCCTTAACTAATTAGAACTACCTTAGTTTTGTGTAGATGTATACATAC

CTAATAACTTTATCTTTAGATATTGGCATGATAATAAATATAACATATGCTTTTCTTTTT

TGTCATATAGTTCAGAGCATCGGATAAAACTGCCCAGTTAAATCAAATTCCAAATTTCCA

ATCCAATTCTATATGTTTCTTAAATAGTTTATATAGTAAAAACCGAGGAGAAATCAAATA

AAAGTAAGATATTATTCCATTTTGAATCGAGTTAAATTAGTATTAAACCTTTGAATGTGT

GTTTTTTTCACCAATTTGATATTGGGTTCGATTTTCTTAACATTGCATATAAATATATAA

TGTCTTCGTTACTATCTAGATTTGTTTAACATTTTTTTTATATATATATGCTAATTACAG

A

**34. *AhNSur10***

GTTTTCCTCAGCTGGGTCAGACGCGCCACGACGACATTCTCCTCGGCTACAACAGGCGCG

CCACGATAGCATTCTCCTCAAGTTGGGTCACAGGTATGCGATGAAGGTGTTGTAGTCACC

TCAATAGTGAGAGGGAGAAGCAGTGAGAGGGGAAGAGATTGGCGGTAATGGATTAGATAG

GTGGCGGACGGCAATGAGGAGGTAGTAGGAGTGTTTCTGTGACTCTGTGAGTATTTTTGA

GGGATAAGTGAGGAGAGAGGAAGAAGAGAAGAGAGGGAGTGACGGTAGAATGGGGTTAGG

GTGGGGTAGTTTAGGAATTTTATTAAAAAATCAACAAAAAATTAGGATTTGGATACTTTT

GTTATACGGAACCCATCTTTTATGGGTACAGCGTTAATTTCTTTAGTTTATAGGTACTTT

TGTCAGCATTCGTAAACTTTATGGATATTTTTGGTAGTTTTTCCACCGAAATTATTTAAT

ACAACAGTATAAATATGATACCAAGACAAAACCTATGAGAGTACCTCTTTTGTTTTTCAG

CAAAAAATAGATATTACGTAATTTAATTGCACTAGAAACAAACACTAAAAAATATACTAT

AAAACTCTCTTTGTAGCAATAATAAAGGATTTGTATTTATTTTAAATTTTTTAAAAAAAT

GGAACAATGGTAAATAACAATACACAATGGTGATGTGAGAGGACATTGACCAAATGGATA

AAAATAAGGAATAATTATTACAAATAGATTCTTTTGAATGATAAAAAAAGATATATTTGC

AATTCACAAGTAAAAACACCCATATAGAAGCAACCTCTCCTTTAATCATATATATATATA

TATATACACGCATACTTTCTCTTGGTGTTGGTCTATAAATTCGTTGTTGTATTGATTTTC

CTTCTTTCATATTAATAACTAATCATTATATTCTTTTGGAAGGGGAAACTATTTGAGGTA

CGCTTCTTCATTCCATCTTCTATCTTATTAACAATTCACTTGAGCATGCCCCTTTCGAAT

TTCAGTTATAGAATACCTACTACTCTATATGCACGTTGACTTTATAATCAAACCATTGCT

AGCTTTCTCTTTGCCTTAACTAATTAGAACTACCTTAGTTTTGTGTAGATGTATACATAC

CTAATAACTTTATCTTTAGATATTGGCATGATAATAAATATAACATATGCTTTTCTTTTT

TGTCATATAGTTCAGAGCATCGGATAAAACTGCCCAGTTAAATCAAATTCCAAATTTCCA

ATCCAATTCTATATGTTTCTTAAATAGTTTATATAGTAAAAACCGAGGAGAAATCAAATA

AAAGTAAGATATTATTCCATTTTGAATCGAGTTAAATTAGTATTAAACCTTTGAATGTGT

GTTTTTTTCACCAATTTGATATTGGGTTCGATTTTCTTAACATTGCATATAAATATATAA

TGTCTTCGTTACTATCTAGATTTGTTTAACATTTTTTTTATATATATATGCTAATTACAG

A

**35. *AhNSur11***

CATGTTTGGTCACGCCTCTTTAGATGTAC

TCTGATATTTATGAATTGTGAGGTGTATTTTTGTCTATTTCAAACTCTTTCACGTGTACT

TTTGTCTCTTTACTCATCTTTGTATGAACATTGTGTATTCTATATAATCTGCTTCTGTTT

TGTAACTGCTTTAATTTATAGTTAGTTGTTTGGTAATTAATAATTAATAATAGGGAATTA

GGGATTTTGATGACAGGTTAACTATCAACATGTCTTATGCATCTAAAGATATGGTGATTT

CATTGGCAATACTATTGCTTATGTTAGGCACACCATGCTCAAGTGCTTTTTGGAAGACTC

AGAATAAGATTAAGACAGCTGTTCATCTTTCTCCAAAGATTGAACTTGGGCCAGGGTCAG

TTTCGAATAAATTTTACTATGATATTGAGTTTCCAAGAGGTCATGTTGCGCTTAAGAGTT

TCAATGCTGAAGTAGTTGATGAAGCTGGAAACCCTGTACCTCTCCATAAAACTTATCTCC

ACCATTGGATTATTGTTGGATACCATGAATCCAAATCAAAACTTGCGACACACACAAAAT

ATGATCTTCATCGTGTGGTTCGTGTGTCAGACTCAGTCTCAAAGTCACATATTATACTAA

GAAATAGTGGCGTATGTCAGGGAAATATTCTTGGACAGTATTTTGGACTTGGATCCGAAA

CACGAGGAACGGCTACGGATATTCCAGATCCTTTTGGGATAGAAATAGGAAATCCTGCAG

AAATTCCAGAAGGATATGAGGAGAAATGGTTGCTCAATGTCCACGCCATCGATACACGAG

GTGTAGAGGATAAGCTAGGCTGCACTGAGTGTAAGTGTCACCTTTATAATGTTACAGTCA

ATGAATACGGCAACCCTTTGCCTCCAGATTACGCAGGGGGTTTGTACTGTTGCTATGATG

AGACTCAGTGCAGGTTGAAGAAAGGCTTTCAAGGTCCAAAGAGAAGCCTCTATCTGAGAT

ACACTGTGAAATGGATCGATTGGGACGAATATGTTGTTCCTGTTAAGATTTATATAATTG

ATGTGACTGATACTTTGAAAATATCAGATACTTCAAATATAGCAAGCTCAAATCATGATT

GCCGGGTAAGTGATTTCATCACAAAACGTGTATATTGTCTCAATCAAAATTTAACTCCAT

AACATATTTTACTCTTTTTTTGTTACAGATTGAGTATGAAGTTGATCCTTGCAACATAGA

CCCCAAGAAGAAAGGTAATGGTTGTGTTGATGTGAAGAGGACAACCGTCCCATTGGAAAA

GGGTGGTTATGTGGTCTATGCTGTAGCTCATCAGCATTCAGGTGGAATCGGATCAACTCT

ATATGGACAGGTGATGATCAATAGTTATTCTTAATATTTGGAAATTACTTTCTAGTGCCA

TGACTTCATATGAAAAGTTGTTATTTCAACCTGTAATGAATAATGATTCTGTCCTCTAAA

TTCAAAGGTCTCACAGATCAATTTCTGTTTT

**36 *AhNSur12***

GCCTTGTCACCATTAGAGGGATTATGTGCCCTGTCACCATTAAAGGGAATAGGTGCCCTG

TCACCCTTACAACAAGAGAAAAAACACAGACATACTTGTCATCAATCAATCTCAACCATG

TCTCATTTATCATATTCGTTCTATTTCATAATCAAACTCATTTTTCATCATTTTTCTTTC

TCATCTCTTTCCCAAAGCAAGTGGACAACACCACTGCCTCATACTCGAAGTCTCAACTAT

TAACCAAATTCAATTTTATTTTTAAATCCATTTCTCAATATCATTCTCGTATTCCTCGAG

CTAACTTCAAAACCGTTTCCGAATTTAAACTCTTTTCAAAAGACAAAGAAAAACCATTTA

TTAGTTAAATTCCTTGACAAAGCCTTTAGACTTTATGGAAGCGACAACCAATCTCATTTC

AAAACCAAATCATTTAAACTCAAACATAATTCATTCTTTTATTAAATAAATCAAAATCAA

TTAAAACAATTCTTCAATCCAAATTAACTAAAAATAAAACTTCTCAAAATTCTTCTAAAC

ATTTCAGCAGCACCTTCCCTAAAACTCAAACCTTGCCACCCTTCTTGGGTCCCATCCAGA

CCATTTCCAACAACAAAATCATTTTCAATAAATCAAAACGATTTTCAATTTCAAAATATT

TATAAAATCAAACCAATTAAAAATCAAACTGCTTACAAAGTCAAACCATTTTCATATCTT

AAATCATATCTGATTCTTAGAATCATTTTCGATAAATCAAACAAACTAATTCCAATATTC

TCAAGTCATCTTATCATAATCCAGAAACCAAATTCAAGAAACCAGTCCAACAACGACCAA

CTCAACAAACTAAAACAACAAATCAAAATAACCAGCTTCCTCAATAATTCAATAAACTAA

TAAGATAAACAATCACATCCATCCAAATAGTTCAATTCAATTTATAAGACTCACGTAATA

ATAAAAATATATTTTTTATACTATTATCGACTTATAACAACTCTTGAAAGTAAAATGAAT

TTAAAAAAATGCCCCTACCTCGATCGCGACTTAACTACGCGACAAAGCCCTTAAAACTCT

GATTTCGCAGCAACGACGTCAAATCCGACTATACCCGTAGTAGTCACAGCCTCTAAATAC

AGCACGCAACAACCATAGCTCAACTCTAACAGATCAACGTCACATAAAACTTAATAATCT

ATAACAGAACAGTGACAAAGAGGAGTTTTCAGAGCAAAAAGGCTTACTGAACCACTAGGA

AGGAAGGACAAGGATAGTAGCACCTCCGGCGGTGCTTCTCGGTGACCATGGCGCCATTCT

CCAGCAGCCAGGCGCGGCGGTGCGGCCTTTTCCTCATTCGGCGGGTATCCCTACTCACTG

TTCGCGTTCTGCTTGTCCGTGAAGGCAACTCTCCTCGTGGCTCCCTTATCGACAACATGA

CAGCTTTTAGCGGTGCACAACAGCTCGCCAGCGGCGATTCACGGTGAGGGAGATGGCGAA

**37. *AhNSur13***

GTTGT

TTGTTATTATATCCTTTTTGTAATTTGCATTGAGAAGTCTTTTCACAACAAAAAATTCCT

CCTTTATAATCACCAGACATTGGTTTCCCATCAATACCGGTTTTGCTTACAAAGTCTTCA

CTTTTGACATTATAATGGTCACATCTGCATTCGGTGCAACCTTTTTTGTCTTCTGTACCA

CGTGTGTCAATAACCATGATATCGAATAACCATTTTTCTTCATTATACTCCTTTGGGACA

TTCTCAGGGTGCGTACCTACTTCTATTCTAAATGGATCTGGTAGTTCTAAGCTAGTTTTT

CGTGCATCAACTCCAAGCCCCCAGGAATATGAATTAACACTACCCTGGCATACTCCATCA

TTTCTTCTAAAATACTTACCGTATATGGGCTGACTTTGATTAGCCTGACGTGACATGGTG

ACATTTTCAAAATATCTTAAAACAAAATAATGGTGCAGGTAAGCTTCATATAATGGCAAA

GAATTCTGATGTTCATCAACTAACTCGGCTTGAAAATTCTTGATTCCGATGTGTCCTCTT

GGAAACTCAATATCGAATAAATCTGTTATCGTAACCTTTCCTGGTTCCAATACAAATTGC

TCACTATAAAAAGTAGCCGTCTTGATATGATTTGAATTCTCATATTGTCGTGCGTATATG

ATGTTTGATTGCAACAGTATAATTGTTAATGATAGTAATAACACTTCAGGTATAAACTTC

ATGTTTTCTTGCAAATTGTCATATTTATATGACCATCAATCATATGTTTATATGTTAATC

TATGGAAATATCAATAATATATTTATAAATTAGGAAAACATGTTAAGGAAGACAAATAAA

ATATTAAACCATGTGGAGCTTGAATAAGACATATGAAGTTGAGATTCACATAACATGTCA

AAAAGACAAGTACCTTGTTGGAGATTCAATTTTTGATATTTGAAAAAACAATGGAAGGTG

AATTTGTATTGATATGAAATTTTTGTATGTCTACTGCTTTTATAGCAGAATCTAATGACA

ATTTTGTTGAATTTTCAATATTAGATGCCTATTTTGGTGGGCTTTCTAACTATGTGTTCC

ATTTGTTAAGAAACATTGTTTGTTATTAGTTCCAATTAATGTAACAATAGTTTTTATTTT

TTTTGTATACATTAGGTGACAACTTAATTCCAACTCAACTATGAATTCATCTTAGTTGCA

AAATTGTTTAGATAATAAAATATTTTTTTAGATTTTGTCAAATTTGTTTTTTAACCTTAT

CCTCTTCTTATGCGACTCATTTGATATTTTGGCACAAAATAAATCCACATGAATTTATGT

TATAGTTCAAACTAACACAAAATTTTAAAATAAAAGACTTAATAAAGTATACAAATTATT

TAGAAAATTAAATACGATACTGTCTTGGTATTTCGTATATTTAATACTTCTTTTATAATC

TATTAATTTGATAGATGGATACCCTCTTTCTATTTTCTTTCTTTTTTTTTTTTGT

**38. *AhNSur14***

ATTGGATTTGAAATAAATTAAGGTTTTTATTCAAACTCTATACTTATGGATTGTTCTTTT

ATTTATGATTAAAATTCTAGAAATTCGAAAAAATAATGATATTTGACTATTTAAATTAAA

ATTTTGTCTTATTTTATAATTATTGAATTATTTTCGTCTATAATTAAATTATAAAATTGG

TAGTTATGAAATAATAAATTTATATGATTCGATTTTAGATGCTTTATATTTATATCATTT

AATACTTTAAGTTAAAGGTAAAGAAAATTAATTATATTACCATGTATTTTCAGTTAGAGT

AATTTATTGAGAATTAATTAGTATTTTTATGCCACAAATAATATTTTAAAGTAATTTTAG

AGATTAAATTAATTTTTCAAATATTAATACTTTATACTCCAATTTTATTAAAATTACCAA

ACAATCTCTAGGCTGATTAATTTAAATATGGAAAACATTTTGGTAAATTGGGAAAAAATG

GAGCAACTGTGTGTATTACACAGAAATGGACGTGTCAGATGAAATGCACAGCACGCAAGG

GGCGTGGTGCATACAACACGTGGGGCGTAAGTGGTTCTCCACCATCGGATTCCACGTCTG

TGGTGGATCCAGTTTACGCACCAACAAATTCCTGTTAGGCTGCATTAACAAAAATACATGT

TACATTAATTAAATAATAACAACAACAACAACAACAACAAAGCCTTATCCCACTAAGTGGG

GTCGGCTACATGAATCAAACGACGCCATTGTGCTCTGTCATGTATCATGTCTACAGAGAGA

CCGTTTACATGTAGATCTCGTTTGACCACCTCATGGATGGTCTTCTTAGGTCTTCCTCTGC

CTTTCGCCCTTTGTCCATCTTCCATCTCATCCACCCTCCTGACTGGATGTTCTATCGGTCTT

CTTCTCACATGTCCAAACCACCTGAGACGCGATTCAACCATCTTTTCCACAATGGGTGCTACT

CCAACTCTCTCCCTTATATCTTCATTCCTTATTTTATCCAATCGCGTGTGACCACTCATCCAT

CTCAACATCTTCATCTCTGCCACACTCAGCTTATGTTCGTGCTCTCCTTTAGCCGCCCAACACTC

CGTACCATACAGCATAGCCGGTCTTATAGCGGTGCGATAGAATTTACCTTTAAGTTTTAA

AGGCACTTTTTTGTCGCATATAAAATCAGATGCACTCCGCCATTTTGACCAACCTGCTTG

GATCCTATGATTTACATCATGTTCAATCTCTCCATTATCCTGTATGATGCACCCAAGATA

CTTAAAACTTTTAACTTTTCGTAAGGTGTTCTCTCCAATTTTCACCTCTATATTGGAGTT

TTCCCTTCTCAGACTGAACTTACATTCCATATATTCCGTCTTGCTACGGCTTATGCACAG

ACCATACACTTCTAGAGCTTCTCTCCATAACTCCAACTTCTTATTTAGGTCTTCCCTTGA

CTCTCCCATAAGGACGATATCATCGGCAAAAAGCATGCAC

**39. *AhNSur15***

TCCATAGGTATAGAGATTAGTTTGCAATCGACAAAGCCATAGTCAGTAAGCATATCCACA

ATATACTTCCTCTGGTACAACGTGATCTCTTTATCTGAACGTGCTACTTCTAATCCAAAG

AAGAATTTCAAGTCTCCAATACCCTTGATTTTGAACCGATCATCCAAAACACTTTTGATT

TTGTTGATCTCAAGAATGTCATCTCCGGCCAAAATATCCAATTAGAACATATTATTATTA

AACCGATTAGAACAATAATATCCAAGATATATGGGTTGGGTAAGCAATAAAATCTATGGA

TTAAGCCTTAAACTTGGTAGGCCAGCCACAGCCATAGAATCATCCATTCTTGGTTTGAAT

TGTTTTGGATATAATTATTTATGTGTCTTTTTTTATTAGTTTAAATTTTTAAAAGAAATA

GTTTTTTGATATAATATCAAAATTTTGATTCTATGACCTAAAGATCTAAAGTTCAAAGTT

TATTGACCCCAAAAAAACCCATTTTAATATAACGTCAATAAAAAGAGAAAGAAAATCTAT

AAAAAAATTTACACAAAATAAAAAGAGGTTATTGCATAAATTAAAAAATTAAAAATATAA

ATATTTATATGTCTCTTCTTATCAACTTAAGATTTTAAGACAAATAATATCTAATTTTTA

ACAGTTATTAATCATGTGAAATGATCTCATTAATAATCTCTAATGTCGTTTTCTAACAAG

TGTTACGTTTGATTGATGTTTTTCTGCCTCTTCTTATACATACATATATCGAAAGTTTCA

GATTTTTAAAGAACATCCAAAACAAATTTAATAAGTTAAGCAAGAAAACACAACAAAGTT

TGTCATTGTGTAAAATCTAAAGCTAAAATTTAGGTGCTGTAACCTCAGTGTAGTTTAAAA

TATAACTTATATCAATGTGGATTTTAGCAAAAGAAAAAATCTAACAATGAAAAAACAACA

ATACGTAACAAATTAGATTACAATAACAAAAAATGTTACCTAAACTTTTTTTTCCATAAG

ATAAATTCTTAATTAGGATTGGATTGATACAAAAAAAAATTTTGTAAAACCAACACAGTT

TTTGATTAGTTGAATATGCTCTCATGGTTGAAAATTCCACCAAAAATAGCATTTAACAGT

AAAAATTCATTAAGAGTTGGTATTCAATTTGATGTACAAACTAGATCATTGCTCTACTAT

AAAAGCAGCAGGCAATGCAAGAACTCATATCCATAAATATTTATCTTCTATTTTTTTGAA

TATCAAAGACCCTTTTCTAATATGGTAGTTTCCCTTTTCTCTTGCTGTCATGTTATATTA

TTTTTTTTTCCGTTTTTTCTTGAATTCATGTTTTATTTCTTGAATGTTTAATTTGAGTTC

CTCAAAACGATATGTGTTTACGTAGTTTTTATGAATATGTTAAACATATATAATATAAAT

CGCATTACAAACATTATTGTTCACTACTAATCATATAAGCATAATTATTTCCAGGTCAAC

A

**40. *AhNSur16***

TCCATAGGTATAGAGATTAGTTTGCAATCGACAAAGCCATAGTCAGTAAGCATATCCACA

ATATACTTCCTCTGGTACAACGTGATCTCTTTATCTGAACGTGCTACTTCTAATCCAAAG

AAGAATTTCAAGTCTCCAATACCCTTGATTTTGAACCGATCATCCAAAACACTTTTGATT

TTGTTGATCTCAAGAATGTCATCTCCGGCCAAAATATCCAATTAGAACATATTATTATTA

AACCGATTAGAACAATAATATCCAAGATATATGGGTTGGGTAAGCAATAAAATCTATGGA

TTAAGCCTTAAACTTGGTAGGCCAGCCACAGCCATAGAATCATCCATTCTTGGTTTGAAT

TGTTTTGGATATAATTATTTATGTGTCTTTTTTTATTAGTTTAAATTTTTAAAAGAAATA

GTTTTTTGATATAATATCAAAATTTTGATTCTATGACCTAAAGATCTAAAGTTCAAAGTT

TATTGACCCCAAAAAAACCCATTTTAATATAACGTCAATAAAAAGAGAAAGAAAATCTAT

AAAAAAATTTACACAAAATAAAAAGAGGTTATTGCATAAATTAAAAAATTAAAAATATAA

ATATTTATATGTCTCTTCTTATCAACTTAAGATTTTAAGACAAATAATATCTAATTTTTA

ACAGTTATTAATCATGTGAAATGATCTCATTAATAATCTCTAATGTCGTTTTCTAACAAG

TGTTACGTTTGATTGATGTTTTTCTGCCTCTTCTTATACATACATATATCGAAAGTTTCA

GATTTTTAAAGAACATCCAAAACAAATTTAATAAGTTAAGCAAGAAAACACAACAAAGTT

TGTCATTGTGTAAAATCTAAAGCTAAAATTTAGGTGCTGTAACCTCAGTGTAGTTTAAAA

TATAACTTATATCAATGTGGATTTTAGCAAAAGAAAAAATCTAACAATGAAAAAACAACA

ATACGTAACAAATTAGATTACAATAACAAAAAATGTTACCTAAACTTTTTTTTCCATAAG

ATAAATTCTTAATTAGGATTGGATTGATACAAAAAAAAATTTTGTAAAACCAACACAGTT

TTTGATTAGTTGAATATGCTCTCATGGTTGAAAATTCCACCAAAAATAGCATTTAACAGT

AAAAATTCATTAAGAGTTGGTATTCAATTTGATGTACAAACTAGATCATTGCTCTACTAT

AAAAGCAGCAGGCAATGCAAGAACTCATATCCATAAATATTTATCTTCTATTTTTTTGAA

TATCAAAGACCCTTTTCTAATATGGTAGTTTCCCTTTTCTCTTGCTGTCATGTTATATTA

TTTTTTTTTCCGTTTTTTCTTGAATTCATGTTTTATTTCTTGAATGTTTAATTTGAGTTC

CTCAAAACGATATGTGTTTACGTAGTTTTTATGAATATGTTAAACATATATAATATAAAT

CGCATTACAAACATTATTGTTCACTACTAATCATATAAGCATAATTATTTCCAGGTCAAC

A

**41. *AhNSur17***

TCCATAGGTATAGAGATTAGTTTGCAATCGACAAAGCCATAGTCAGTAAGCATATCCACA

ATATACTTCCTCTGGTACAACGTGATCTCTTTATCTGAACGTGCTACTTCTAATCCAAAG

AAGAATTTCAAGTCTCCAATACCCTTGATTTTGAACCGATCATCCAAAACACTTTTGATT

TTGTTGATCTCAAGAATGTCATCTCCGGCCAAAATATCCAATTAGAACATATTATTATTA

AACCGATTAGAACAATAATATCCAAGATATATGGGTTGGGTAAGCAATAAAATCTATGGA

TTAAGCCTTAAACTTGGTAGGCCAGCCACAGCCATAGAATCATCCATTCTTGGTTTGAAT

TGTTTTGGATATAATTATTTATGTGTCTTTTTTTATTAGTTTAAATTTTTAAAAGAAATA

GTTTTTTGATATAATATCAAAATTTTGATTCTATGACCTAAAGATCTAAAGTTCAAAGTT

TATTGACCCCAAAAAAACCCATTTTAATATAACGTCAATAAAAAGAGAAAGAAAATCTAT

AAAAAAATTTACACAAAATAAAAAGAGGTTATTGCATAAATTAAAAAATTAAAAATATAA

ATATTTATATGTCTCTTCTTATCAACTTAAGATTTTAAGACAAATAATATCTAATTTTTA

ACAGTTATTAATCATGTGAAATGATCTCATTAATAATCTCTAATGTCGTTTTCTAACAAG

TGTTACGTTTGATTGATGTTTTTCTGCCTCTTCTTATACATACATATATCGAAAGTTTCA

GATTTTTAAAGAACATCCAAAACAAATTTAATAAGTTAAGCAAGAAAACACAACAAAGTT

TGTCATTGTGTAAAATCTAAAGCTAAAATTTAGGTGCTGTAACCTCAGTGTAGTTTAAAA

TATAACTTATATCAATGTGGATTTTAGCAAAAGAAAAAATCTAACAATGAAAAAACAACA

ATACGTAACAAATTAGATTACAATAACAAAAAATGTTACCTAAACTTTTTTTTCCATAAG

ATAAATTCTTAATTAGGATTGGATTGATACAAAAAAAAATTTTGTAAAACCAACACAGTT

TTTGATTAGTTGAATATGCTCTCATGGTTGAAAATTCCACCAAAAATAGCATTTAACAGT

AAAAATTCATTAAGAGTTGGTATTCAATTTGATGTACAAACTAGATCATTGCTCTACTAT

AAAAGCAGCAGGCAATGCAAGAACTCATATCCATAAATATTTATCTTCTATTTTTTTGAA

TATCAAAGACCCTTTTCTAATATGGTAGTTTCCCTTTTCTCTTGCTGTCATGTTATATTA

TTTTTTTTTCCGTTTTTTCTTGAATTCATGTTTTATTTCTTGAATGTTTAATTTGAGTTC

CTCAAAACGATATGTGTTTACGTAGTTTTTATGAATATGTTAAACATATATAATATAAAT

CGCATTACAAACATTATTGTTCACTACTAATCATATAAGCATAATTATTTCCAGGTCAAC

A

**42. *AhNSur18***

TCCATAGGTATAGAGATTAGTTTGCAATCGACAAAGCCATAGTCAGTAAGCATATCCACA

ATATACTTCCTCTGGTACAACGTGATCTCTTTATCTGAACGTGCTACTTCTAATCCAAAG

AAGAATTTCAAGTCTCCAATACCCTTGATTTTGAACCGATCATCCAAAACACTTTTGATT

TTGTTGATCTCAAGAATGTCATCTCCGGCCAAAATATCCAATTAGAACATATTATTATTA

AACCGATTAGAACAATAATATCCAAGATATATGGGTTGGGTAAGCAATAAAATCTATGGA

TTAAGCCTTAAACTTGGTAGGCCAGCCACAGCCATAGAATCATCCATTCTTGGTTTGAAT

TGTTTTGGATATAATTATTTATGTGTCTTTTTTTATTAGTTTAAATTTTTAAAAGAAATA

GTTTTTTGATATAATATCAAAATTTTGATTCTATGACCTAAAGATCTAAAGTTCAAAGTT

TATTGACCCCAAAAAAACCCATTTTAATATAACGTCAATAAAAAGAGAAAGAAAATCTAT

AAAAAAATTTACACAAAATAAAAAGAGGTTATTGCATAAATTAAAAAATTAAAAATATAA

ATATTTATATGTCTCTTCTTATCAACTTAAGATTTTAAGACAAATAATATCTAATTTTTA

ACAGTTATTAATCATGTGAAATGATCTCATTAATAATCTCTAATGTCGTTTTCTAACAAG

TGTTACGTTTGATTGATGTTTTTCTGCCTCTTCTTATACATACATATATCGAAAGTTTCA

GATTTTTAAAGAACATCCAAAACAAATTTAATAAGTTAAGCAAGAAAACACAACAAAGTT

TGTCATTGTGTAAAATCTAAAGCTAAAATTTAGGTGCTGTAACCTCAGTGTAGTTTAAAA

TATAACTTATATCAATGTGGATTTTAGCAAAAGAAAAAATCTAACAATGAAAAAACAACA

ATACGTAACAAATTAGATTACAATAACAAAAAATGTTACCTAAACTTTTTTTTCCATAAG

ATAAATTCTTAATTAGGATTGGATTGATACAAAAAAAAATTTTGTAAAACCAACACAGTT

TTTGATTAGTTGAATATGCTCTCATGGTTGAAAATTCCACCAAAAATAGCATTTAACAGT

AAAAATTCATTAAGAGTTGGTATTCAATTTGATGTACAAACTAGATCATTGCTCTACTAT

AAAAGCAGCAGGCAATGCAAGAACTCATATCCATAAATATTTATCTTCTATTTTTTTGAA

TATCAAAGACCCTTTTCTAATATGGTAGTTTCCCTTTTCTCTTGCTGTCATGTTATATTA

TTTTTTTTTCCGTTTTTTCTTGAATTCATGTTTTATTTCTTGAATGTTTAATTTGAGTTC

CTCAAAACGATATGTGTTTACGTAGTTTTTATGAATATGTTAAACATATATAATATAAAT

CGCATTACAAACATTATTGTTCACTACTAATCATATAAGCATAATTATTTCCAGGTCAAC

A
