# Supplementary material for: Genome wide identification and characterization of nodulation related genes in Arachis hypogaea
Source: PLoS One. 2022 Sep 9;17(9):e0273768. doi: 10.1371/journal.pone.0273768 (PMC9462762; doi:10.1371/journal.pone.0273768)
Supplement: S1 Table — (DOCX) [file pone.0273768.s004.docx]

| **Functions based Categories** | **Sequence** | **Extracted Cis-elements** | **Specific functions of cis-elements** |
| --- | --- | --- | --- |
| **Stress Response** | GGTTAA/GGTTAAT/GTGTGTGAA | GT1-motif | light responsive element |
|  | CACGTT/TAAACGTG/TACGTG/CACGTC/CACGAC/TAACACGTAG/ACACGTG(G/t)CACC/ACACGTGT/CACGTG/tgACACGTGGCA/ACACGTGGC/CACGTGAAA/GCCACGTGGA/CAGACGTGGCA | G-Box | light responsiveness,  light responsive element |
|  | CCGAAA | LTR | low-temperature responsiveness |
|  | CAACTG | MBS | MYB drought-inducibility,  drought-inducibility element |
|  | CTAACGTATT/GACACGTATG | ACE | light responsiveness |
|  | ATTAAT | Box 4 | light responsive module |
|  | AACCTAA | MRE | MYB light responsiveness |
|  | CAACGG | CCAAT-box | MYBHv1 binding site |
|  | GATAGGA/ AAGGATAAGG  AAGATAAGATT | GATA-motif | light responsive element |
|  | AATTATTTTTTATT | AT1-motif | light responsive module |
|  | cCATATCCAAT/ TGATAATGTgGATAAGGTG/ ccttatcct | I-box | light responsive element |
|  | AATTACAGCCATT | ACA-motif | gapA light responsiveness |
|  | TGCTATCCA | ATC-motif | light responsive module |
|  | TGGTAATAA | Box II | light responsive element |
|  | CAATCAAAACCT | AAAC-motif | light responsive element |
|  | AAATTTCCT | WUN-motif | wound-responsive element |
|  | ATAGATAA | GA-motif | light responsive element |
|  | CCCCCG | GC-motif | anoxic specific inducibility element |
|  | AAACCA | ARE | anaerobic induction element |
|  | TCTTAC | TCT-motif | light responsive element |
|  | AGAAACAA/AGAAACTT | AE-box | light responsive module |
|  | TTACTTAA | chs-CMA1a | light responsive element |
|  | TCACTTGA | chs-CMA2a | light responsive element |
|  | ATTCTCTAAC/GTTTTCTTAC | TC-rich repeats | defense and stress responsiveness |
|  | TAAGAGAGGAA | 3-AF1 binding site | light responsive element |
|  | TCTCCCT | TCCC-motif | light responsive element |
|  | CTTTATCA/CCTTATCCA | LAMP-element | light responsive element |
|  | GGGCGG | Sp1 | light responsive element |
|  | AATCTAATCC | ATCT-motif | light responsive module |
| **Hormonal Regulation** | ACGTG/CGTACGTGCA/TACGTGTC/TACGGTCCACGTG/GACACGTGGCAACCCGG/GCAACGTGTC | ABRE | abscisic acid responsive element |
|  | TCAGAAGAGG/CCATCTTTTT | TCA-element | salicylic acid responsiveness |
|  | CAACAAACCCCTT/CCTTTTG | P-box | gibberellin responsive element |
|  | TGACG | TGACG-motif | MeJA-responsive element |
|  | AACGAC | TGA-element | auxin responsive element |
|  | GATGATGTGG/GATGACATGG/GTTGACGTGA/GATGA(C/T)(A/G)TG(A/G) | O2-site | zein metabolism regulation element |
|  | aaaAaaC(G/C)GTTA/TTTTTACGGTTA | MBSI | MYB flavonoid biosynthetic genes regulation |
|  | CGTCA | CGTCA-motif | MeJA-responsive element |
|  | GGTCCAT | AuxRR-core | auxin responsive element |
|  | TAAAATACT | AT-rich sequence | maximal elicitor-mediated activation element |
|  | TCTGTTG | GARE-motif | gibberellin responsive element |
|  | TATCCCA | TATC-box | gibberellin responsive element |
|  | TTCGACCATCTT | SARE | salicylic acid responsive element |
| **Cellular Development** | GCCACT | CAT-box | meristem expression |
|  | TGAGTCA | GCN4_motif | endosperm expression |
|  | CAAAGATATC | circadian | circadian control element |
