# Supplementary figures and images for: Genome wide identification and characterization of nodulation related genes in Arachis hypogaea
Source: PLoS One. 2022 Sep 9;17(9):e0273768. doi: 10.1371/journal.pone.0273768 (PMC9462762; doi:10.1371/journal.pone.0273768)

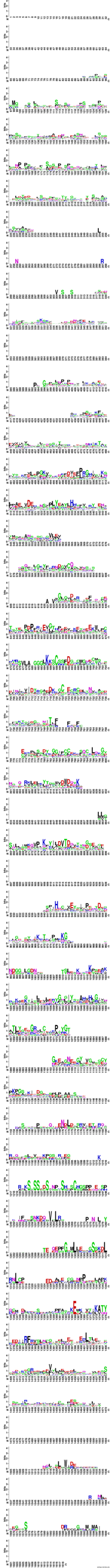

Supplement: S1 Fig — The C-terminal and N-terminal pf NIG gene domain are represented by using ‘N’ and ‘C’. (TIF) [file pone.0273768.s005.tif]
